# Supplementary material for: Bicarbonate-binding catalysis for the enantioselective desymmetrization of keto sulfonium salts
Source: Nat Commun. 2024 Jun 3;15:4727. doi: 10.1038/s41467-024-48832-x (PMC11148132; doi:10.1038/s41467-024-48832-x)
Supplement: Supplementary file 1 — Suplementary Information [file 41467_2024_48832_MOESM1_ESM.pdf]

Supplementary Information

Bicarbonate-Binding Catalysis for the Enantioselective Desymmetrization of Ketosulfonium Salts

José Alemán,[1,2,3]* Jorge Humbrías-Martín,[1] Roberto del Río-Rodríguez,[1] Fernando Aguilar-Galindo,[2,4] Sergio Díaz-Tendero[2,4,5] and Jose A. Fernández-Salas[1,2]*

[1] Departamento de Química Orgánica (módulo 1), Universidad Autónoma de Madrid, Cantoblanco, 28049-Madrid, Spain.

[2] Institute for Advanced Research in Chemical Sciences (IAdChem), Universidad Autónoma de Madrid, 28049-Madrid, Spain.

[3] Center for Innovation in Advanced Chemistry (ORFEO-CINQA), Universidad Autónoma de Madrid, 28049 Madrid, Spain

[4] Departamento de Química, Universidad Autónoma de Madrid, Cantoblanco, 28049-Madrid, Spain.

[5] Condensed Matter Physics Center (IFIMAC), Universidad Autónoma de Madrid, 28049-Madrid, Spain.

e-mail: jose.aleman@uam.es; j.fernandez@uam.es

1. General methods and Starting materials 3

2. Synthesis and characterization data of sulfonium salts (1a-l) 3

2.1. General procedure A: synthesis and characterization data of dienones 4

2.2. General procedure B: cyclization reaction and formation of salts (1a-l) 6

3. Synthesis and characterization data of sulfonium salt 4 10

3.1. General procedure C for the synthesis of sulfonium salt 4 10

3.2. Characterization data of sulfonium salt 4 11

4. Screening of reaction conditions 12

5. General Procedures for the Enantioselective Desymmetrization 14

5.1. General procedure C and D for the Enantioselective Desymmetrization of sulfonium salts 1a-l and 4 14

5.2. Characterization data of 3a-l and 5 14

6. General procedure E: derivatization of 3a to 6 19

7. General procedure F: derivatization of (*R*)-3a to 7 20

8. General procedure F: derivatization of (*R*)-3a to 8 21

9. General procedure F: derivatization of (*R*)-3d to 9 22

10. NMR studies. 23

10.1. Titration experiments. Procedure for the titration experiments 23

10.2. Titration experiments. Determination of the binding constant 24

10.3. ^{13}C-NMR. 25

11. Computational studies 27

11.1. Mechanism of the reaction: anion-binding process 27

11.2. Study of the equilibrium between sulfonium salt and HCO_3^- 30

11.3. Computational study of non-covalent interactions and energy decomposition análisis 31

12. X-Ray crystal structure analysis of 3a 39

13. Spectra Data. 41

13.1. (1E,4E)-1,5-bis(4-bromophenyl)penta-1,4-dien-3-one: NMR 41

13.2. Sulfonium salts: NMR 42

13.3. Products: NMR and SFC chromatograms 58

13.4. Derivatizations: NMR and chromatograms 86

14. Coordinates 94

15. Supplementary References 117

1. General methods and Starting materials

- Catalysts **2f and 2j** were acquired from commercial sources and catalysts **2a-e** and **2g-i** were synthesized following procedures described in the literature.[1,2,3] $TBAHCO_3$ was synthesized following a procedure described in the literature.[4]
- For thin layer chromatography, (TLC) silica gel plates with fluorescence indicator 254 nm were used and compounds were visualized by irradiation with UV light and/or by treatment with a solution of potassium permanganate in water followed by heating. Flash column chromatography was performed using Geduran® Si 60 (silica gel). Starting materials are purified either by manual flash chromatography or on Biotage Isolera Prime® using SNAP cartridge KP-SIL 50g or KP-SIL 10g. As eluent mixtures of cyclohexane: ethylacetate were used in all cases, except when indicated.
- Optical rotation was recorded in cells with 10 cm path length; the specific solvents and concentrations (in g/100 mL) are indicated.
- NMR spectra were acquired on a *Bruker Avance 300 MHz spectrometer*, running at 300, 76 MHz for ^1H, ^{13}C, and the ^{19}F were adquired on a Bruker Avance 500 MHz spectrometer running at 471 MHz. Chemical shifts (δ) are reported in ppm relative to residual solvent signals ($CDCl_3$, 7.26 ppm; acetone-d_6, 2.05 ppm in ^1H-NMR and $CDCl_3$, 77.2 ppm; acetone-d_6, 206.3 ppm for ^{13}C-NMR). ^{13}C and ^{19}F NMR spectra were adquired on a broad band decoupled mode. The following abbreviations are used to describe peak patterns when appropriate: s (singlet), d (doublet), t (triplet), q (quartet), m (multiplet), bs (broad singlet), app (apparent).
- For measuring the exact mass (indicated for each case): MS (ESI) (Electrospray ionization mass spectroscopy) was acquired with an *Agilent Technologies 6120 Quadrupole LC/MS*. In this technique, *MassWorks* software ver. 4.0.0.0. (*Cerno Bioscience*) was used for the formula identification. *MassWorks* is a MS calibration software that calibrates for isotope profile as well as for mass accuracy allowing highly accurate comparisons between calibrated and theoretical spectra.[5,6,7,8]
- Enantiomeric excesses were determined in a Supercritical Fluid Chromatography (SFC) with chiral columns. The chromatograms were acquired with *an Agilent Technologies 1260 Infinity* with a *SFC module* and a UV-vis detector. The chiral columns used were: Chiralpak IA, IB-3, IC, ID-3, IG-3 (see in each case).

2. Synthesis and characterization data of sulfonium salts (1a-l)

The total synthesis of sulfonium salts **1a-l** is a sequential synthesis that consists in 3 steps. The following scheme shows the global synthesis and intermediates prepared.

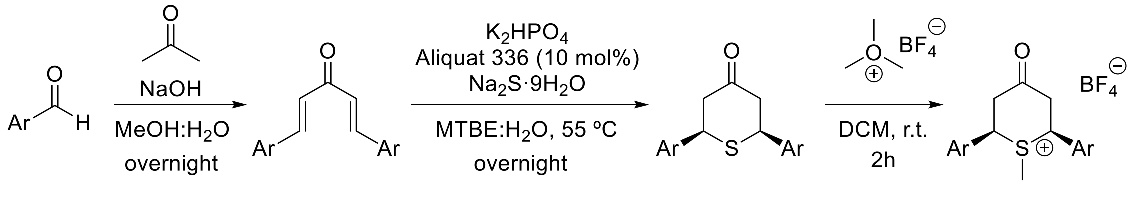

1a-l

2.1. General procedure A: synthesis and characterization data of dienones[9,10]

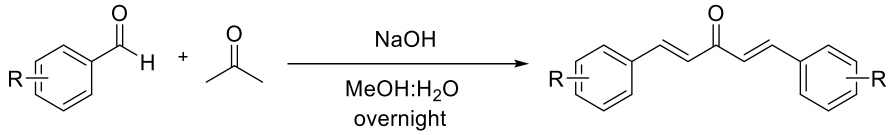

The corresponding dienones were prepared following a procedure described in the literature.[5-8] corresponding aldehyde (2.0 equiv., 20.0 mmol) and acetone (1.0 equiv., 10 mmol, 0.74 mL) were combined in a mixture methanol (18 mL) and stirred for 15 min at room temperature. A solution of sodium hydroxide (5 equiv., 2.0 g, 50.0 mmol) in water (18 ml) was added and the solution stirred overnight at room temperature. The resulting precipitate was filtered and wash with cold methanol. The final product was used in the next step without further purifications required.

(1*E*,4*E*)-1,5-diphenylpenta-1,4-dien-3-one[5]

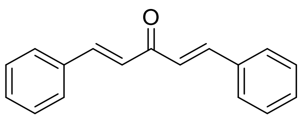

Following general procedure A, benzaldehyde (20 mmol, 2.0 mL) gave (1*E*,4*E*)-1,5-diphenylpenta-1,4-dien-3-one in 84% yield (2.0 g). The ^{1}H-NMR is in accordance with the literature. **^{1}H NMR** (300 MHz, $CDCl_3$) δ 7.07 (d, *J*=15.9 Hz, 2H), 7.40 (m, 8H), 7.61 (m, 2H), 7.73 (d, *J* = 15.9 Hz, 2H).

Dimethyl 4,4'-((1*E*,4*E*)-3-oxopenta-1,4-diene-1,5-diyl)dibenzoate[5]

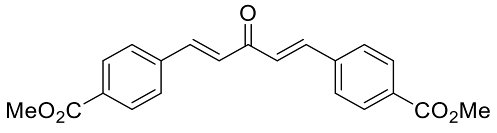

Following general procedure A, methyl 4-formylbenzoate (20 mmol, 3.4 g) gave dimethyl 4,4'-((1*E*,4*E*)-3-oxopenta-1,4-diene-1,5-diyl)dibenzoate in 71% yield (2.5 g). The ^{1}H-NMR is in accordance with the literature. **^{1}H NMR** (300 MHz, $CDCl_3$) δ 8.06 (d, 4H, *J* = 8.1 Hz), 7.73 (d, 2H, *J* = 16.0 Hz), 7.65 (d, *J* = 8.1 Hz, 4H), 7.12 (d, *J* = 16.0 Hz, 2H), 3.92 (s, 6H).

(1*E*,4*E*)-1,5-bis(4-(trifluoromethyl)phenyl)penta-1,4-dien-3-one[5]

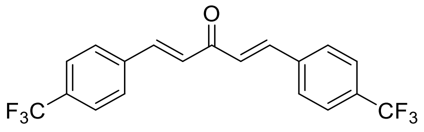

Following general procedure A, 4-(trifluoromethyl) benzaldehyde (20 mmol, 2.7 mL) gave (1*E*,4E)-1,5-bis(4-(trifluoromethyl)phenyl)penta-1,4-dien-3-one in 87% yield (3.2 g). The ^{1}H-NMR is in accordance with the literature. **^{1}H NMR** (300 MHz, $CDCl_3$) δ 7.73 (d, *J* = 15.9 Hz, 2H), 7.69 (m, 8H), 7.12 (d, *J* = 15.9 Hz, 2H).

(1*E*,4*E*)-1,5-bis(4-fluorophenyl)penta-1,4-dien-3-one[5]

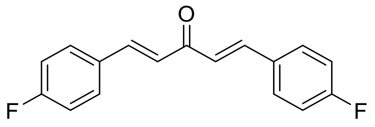

Following general procedure A, 4-fluorobenzaldehyde (20 mmol, 2.2 mL) gave (1*E*,4*E*)-1,5-bis(4-fluorophenyl)penta-1,4-dien-3-one in 85% yield (2.3 g). The ^{1}H-NMR is in accordance with the literature. **^{1}H NMR** (300 MHz, $CDCl_3$) δ 7.68 (d, *J* = 15.9 Hz, 2H), 7.58 (m, 4H), 7.09 (m, 4H), 6.97 (d, *J* = 15.9 Hz, 2H).

(1*E*,4*E*)-1,5-bis(4-chlorophenyl)penta-1,4-dien-3-one[5]

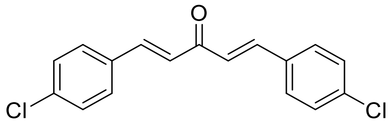

Following general procedure A, 4-chlorobenzaldehyde (20 mmol, 2.8 g) gave (1*E*,4*E*)-1,5-bis(4-chlorophenyl)penta-1,4-dien-3-one in 69% yield (2.1 g). The ^{1}H-NMR is in accordance with the literature. **^{1}H NMR** (300 MHz, $CDCl_3$) δ 7.66 (d, *J* = 15.9 Hz, 2H), 7.52 (d, *J* = 8.5 Hz, 4H), 7.37 (d, *J* = 8.5 Hz, 4H), 7.00 (d, *J* = 15.9 Hz, 2H).

(1*E*,4*E*)-1,5-bis(4-bromophenyl)penta-1,4-dien-3-one

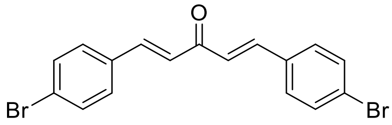

Following general procedure A, 4-bromobenzaldehyde (20 mmol, 3.7 g) gave (1*E*,4*E*)-1,5-bis(4-bromophenyl)penta-1,4-dien-3-one in 84% yield (3.3 g). **^{1}H NMR** (300 MHz, $CDCl_3$) δ 7.67 (d, *J* = 16.0 Hz, 2H), 7.55 (d, *J* = 8.2 Hz, 4H), 7.47 (d, *J* = 8.2 Hz, 4H), 7.05 (d, *J* = 16.0 Hz, 2H).**^{13}C NMR** (76 MHz, $CDCl_3$) δ 188.5, 142.3, 133.8, 132.4, 129.9, 125.9, 125.0. **HRMS** (ESI): *m/z* calculated for $C_{17}H_{13}Br_2O$ $[M+H]^+$: 389.9255; found 389.9259.

(1*E*,4*E*)-1,5-bis(4-methoxyphenyl)penta-1,4-dien-3-one[5]

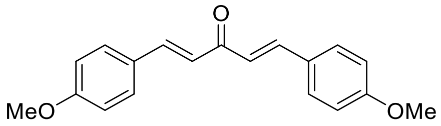

Following general procedure A, 4-methoxy benzaldehyde (20 mmol, 2.4 mL) gave (1*E*,4*E*)-1,5-bis(4-methoxyphenyl)penta-1,4-dien-3-one in 68% yield (2.0 g). The ^{1}H-NMR is in accordance with the literature. **^{1}H NMR** (300 MHz, $CDCl_3$) δ 7.68 (d, *J* = 15.9 Hz, 2H), 7.54 (d, *J* = 8.5 Hz, 4H), 6.93 (d, *J* = 15.9 Hz, 2H), 6.90 (d, *J* = 8.5 Hz, 4H), 3.82 (s, 6H).

(1*E*,4*E*)-1,5-bis(4-methylphenyl)penta-1,4-dien-3-one[5]

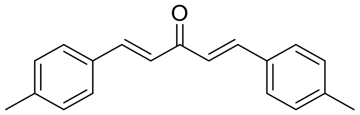

Following general procedure A, 4-methylbenzaldehyde (20 mmol, 2.4 mL) gave (1*E*,4*E*)-1,5-bis(4-methylphenyl)penta-1,4-dien-3-one in 76% yield (2.0 g). The ^{1}H-NMR is in accordance with the literature. **^{1}H NMR** (300 MHz, $CDCl_3$) δ 7.70 (d, *J* = 15.9 Hz, 2H), 7.50 (d, *J* = 7.9 Hz, 4H), 7.20 (d, *J* = 7.9 Hz, 4H), 7.02 (d, *J* = 15.9 Hz, 2H), 2.37 (s, 6H).

(1*E*,4*E*)-1,5-bis(3-methylphenyl)penta-1,4-dien-3-one[5]

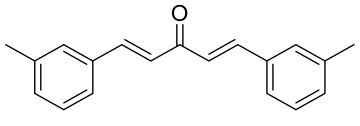

Following a modified procedure A,[11] 3-methylbenzaldehyde (20 mmol, 2.4 mL) gave (1*E*,4*E*)-1,5-bis(3-methylphenyl)penta-1,4-dien-3-one in 68% yield (1.8 g). The ^{1}H-NMR is in accordance with the literature. **^{1}H NMR** (300 MHz, $CDCl_3$) δ 7.70 (d, *J* = 15.9 Hz, 2H), 7.40 (m, 4H), 7.26 (m, 4H), 7.06 (d, *J* = 15.9 Hz, 2H), 2.38 (s, 6H).

(1*E*,4*E*)-1,5-bis(2-methylphenyl)penta-1,4-dien-3-one[5]

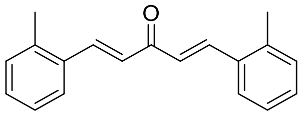

Following a modified general procedure A,[9-10] 2-methylbenzaldehyde (20 mmol, 2.3 mL) gave (1*E*,4*E*)-1,5-bis(2-methylphenyl)penta-1,4-dien-3-one in 73% yield (1.9 g). The ^{1}H-NMR is in accordance with the literature. **^{1}H NMR** (300 MHz, $CDCl_3$) δ 8.03 (d, *J* = 15.9 Hz, 2H), 7.64 (d, *J* = 7.2 Hz, 2H), 7.24 (m, 6H), 6.98 (d, J=15.9 Hz, 2H), 2.47 (s, 6H).

(1*E*,4*E*)-1,5-di(naphthalen-2-yl)penta-1,4-dien-3-one[5]

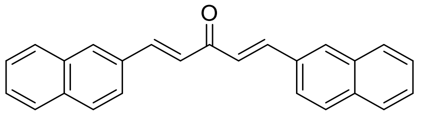

Following a modified general procedure A,[11] 2-naphthalaldehyde (20 mmol, 3.2 mL) gave (1*E*,4*E*)-1,5-di(naphthalen-2-yl)penta-1,4-dien-3-one in 72% yield (2.4 g). The ^{1}H-NMR is in accordance with the literature. **^{1}H NMR** (300 MHz, $CDCl_3$) δ 8.03 (d, *J* = 15.9 Hz, 2H), 7.64 (d, *J* = 7.2 Hz, 2H), 7.24 (m, 6H), 6.98 (d, J=15.9 Hz, 2H), 2.47 (s, 6H).

(1*E*,4*E*)-1,5-di([1,1'-biphenyl]-4-yl)penta-1,4-dien-3-one[6]

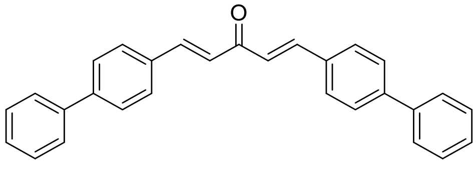

Following a modified general procedure A,[11] [1,1'-biphenyl]-4-carbaldehyde (20 mmol, 3.6 mL) gave (1*E*, 4*E*)-1,5-di([1,1'-biphenyl]-4-yl)penta-1,4-dien-3-one in 72% yield (2.8 g). **^{1}H NMR** (300 MHz, $CDCl_3$) δ 7.75-7.58 (m, 10H), 7.56 – 7.35 (m, 8H), 6.79 (d, *J* = 16.2 Hz, 2H).

2.2. General procedure B: cyclization reaction and formation of salts (1a-l)

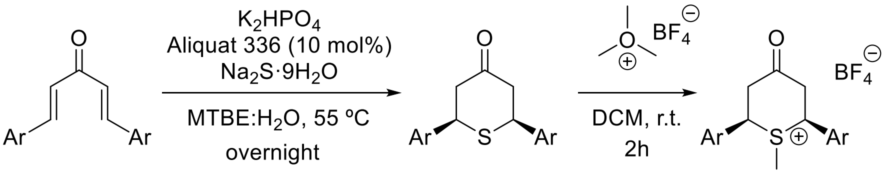

1a-l

The cyclization reaction was carried out following a general procedure described in the literature.[12] Dienone starting material (1.0 equiv., 1 mmol) and Aliquat® 336 (0.1 equiv., 0.1 mmol, 40 mg) were dissolved in MTBE (10 mL) under argon. Then, a solution of potassium phosphate dibasic (4.0 equiv., 4 mmol, 697 mg) in water (3.5 mL) was added to the reaction mixture. To this vigorously stirred diphasic solution sodium sulfide nonahydrate (2.0 equiv., 2 mmol, 480 mg) was added in one portion and the reaction was heated at 55 °C under argon and vigorous stirred overnight. After being cooled to room temperature, the mixture was quenched by addition of cold 1 M hydrochloric acid (5 mL) and saturated ammonium chloride (5 mL). The organic phase was separated and the aqueous was extracted with chloroform (3 x 10 mL). Combined organic phases were washed with brine, dried over $MgSO_4$, filtered, and concentrated under reduced pressure. The residue was used in the next step without further purification.

In a round-bottomed flask trimethyloxonium tetrafluoroborate (1.1 equiv., 1.1 mmol, 162.7 mg) was suspended in DCM (4 mL) under Argon atmosphere, and the suspension was flushed with Argon for 10 min. Then, a solution of the corresponding sulfide (1.0 equiv., 1 mmol; assumed from the previous step) in DCM (6 mL) was added to the reaction crude. The reaction was stirred for 2 hours at room temperature. After the indicated time, diethyl ether was added to the reaction crude observing the appearance of turbidity. Once the turbidity was persistent, the reaction crude was dropped into a beaker with 20 mL of diethyl ether to precipitate the sulfonium salt. After filtration, the corresponding sulfonium salt **1a-l** was dried under vacuum and kept in the fridge. The salts were obtained pure without any further purification.

(2*R*,6*S*)-1-methyl-4-oxo-2,6-diphenylhexahydrothiopyrylium tetrafluoroborate (1a)

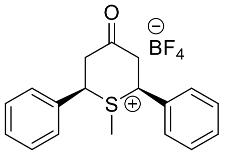

Following general procedure B, (1*E*,4*E*)-1,5-diphenylpenta-1,4-dien-3-one (1 mmol, 234 mg) gave sulfonium salt **1a** as a light yellowish solid in 76% yield (282 mg).

^{1}H NMR (300 MHz, acetone-d_6) δ 7.80 – 7.65 (m, 4H), 7.64 – 7.50 (m, 6H), 5.47 (dd, *J* = 13.6, 3.5 Hz, 2H), 4.01 – 3.85 (m, 2H), 3.27 – 3.11 (m, 2H), 2.80 (s, 3H). **^{13}C NMR** (76 MHz, acetone-d_6) δ 199.6, 133.0, 131.7, 131.0, 129.7, 59.6, 46.3, 21.5. **^{19}F NMR** (471 MHz, acetone-d_6) δ -149.26. **HRMS** (ESI): *m/z* calculated for $C_{18}H_{19}OS$ $[M-BF_4]^+$: 283.1151; found 283.1145.

(2*R*,6*S*)-2,6-bis(4-(methoxycarbonyl)phenyl)-1-methyl-4-oxohexahydrothiopyrylium tetrafluoroborate (1b)

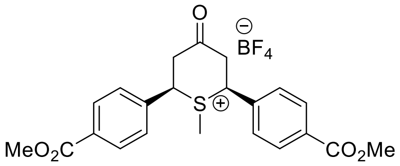

Following general procedure B, dimethyl 4,4'-((1*E*,4*E*)-3-oxopenta-1,4-diene-1,5-diyl)dibenzoate (1 mmol, 350 mg) gave sulfonium salt **1b** as an orange solid in 80% yield (390 mg).

^{1}H NMR (300 MHz, acetone-d_6) δ 8.10 (d, *J* = 8.2 Hz, 2H), 7.88 (d, *J* = 8.4 Hz, 2H), 5.63 – 5.46 (m, 2H), 4.07 – 3.88 (m, 2H), 3.88 (s, 6H), 3.35 – 3.09 (m, 2H), 2.87 (s, 3H). **^{13}C NMR** (76 MHz, acetone-d_6) δ 198.4, 165.6, 136.6, 132.3, 130.8, 129.3, 58.3, 52.0, 45.0, 20.7. **^{19}F NMR** (471 MHz, acetone-d_6) δ -149.94. **HRMS** (ESI): *m/z* calculated for $C_{22}H_{23}O_5S$ $[M-BF_4]^+$: 399.1261; found 399.1254.

(2*R*,6*S*)-1-methyl-4-oxo-2,6-bis(4-(trifluoromethyl)phenyl)hexahydrothiopyrylium tetrafluoroborate (1c)

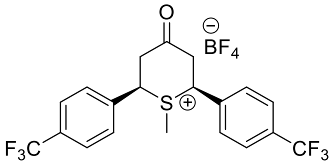

Following general procedure B, (1*E*,4*E*)-1,5-bis(4-(trifluoromethyl)phenyl)penta-1,4-dien-3-one (1 mmol, 370 mg) gave sulfonium salt **1c** as a brownish solid in 57% yield (286 mg).

^{1}H NMR (300 MHz, acetone-d_6) δ 8.00 (d, *J* = 8.2 Hz, 4H), 7.93 (d, *J* = 8.2 Hz, 4H), 5.68 (dd, *J* = 13.4, 3.1 Hz, 2H), 3.98 (dd, *J* = 16.1, 13.4 Hz, 2H), 3.32 (dd, *J* = 16.1, 3.1 Hz, 2H), 2.97 (s, 3H). **^{13}C NMR** (76 MHz, acetone-d_6) δ 198.9, 137.3, 132.9 (q, *J* = 32.6 Hz), 130.9, 127.8 (q, *J* = 3.9 Hz), 124.9 (q, *J* = 271.7 Hz), 59.0, 45.9, 21.7. **^{19}F NMR** (471 MHz, acetone-d_6) δ -63.48, -148.74. **HRMS** (ESI): *m/z* calculated for $C_{20}H_{17}F_6OS$ $[M-BF_4]^+$: 419.0899; found 419.0900.

(2*R*,6*S*)-2,6-bis(4-fluorophenyl)-1-methyl-4-oxohexahydrothiopyrylium tetrafluoroborate (1d)

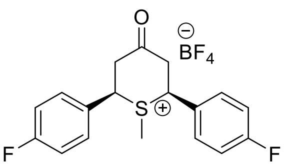

Following general procedure B, (1*E*,4*E*)-1,5-bis(4-fluorophenyl)penta-1,4-dien-3-one (1 mmol, 270 mg) gave sulfonium salt **1d** as a brownish solid in 64% yield (260 mg).

^{1}H NMR (300 MHz, acetone-d_6) δ 7.86 – 7.74 (m, 4H), 7.44 – 7.24 (m, 4H), 5.45 (dd, *J* = 13.6, 3.3 Hz, 2H), 3.93 (td, *J* = 15.5, 13.6, 2.7 Hz, 2H), 3.18 (dd, *J* = 15.5, 3.3 Hz, 2H), 2.83 (s, 3H). **^{13}C NMR** (76 MHz, acetone-d_6) δ 199.4, 164.8 (d, *J* = 248.9 Hz), 132.3 (d, *J* = 8.9 Hz), 129.2 (d, *J* = 3.2 Hz), 117.9 (d, *J* = 22.3 Hz), 58.7, 46.3, 21.3. **^{19}F NMR** (471 MHz, acetone-d_6) δ -111.26, -149.09. **HRMS** (ESI): *m/z* calculated for $C_{18}H_{17}F_2OS$ $[M-BF_4]^+$: 319.0963; found 399.0963.

(2*R*,6*S*)-2,6-bis(4-chlorophenyl)-1-methyl-4-oxohexahydrothiopyrylium tetrafluoroborate (1e)

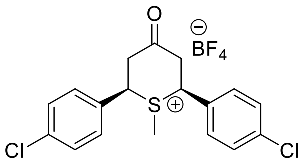

Following general procedure B, (1*E*,4*E*)-1,5-bis(4-chlorophenyl)penta-1,4-dien-3-one (1 mmol, 303 mg) gave sulfonium salt **1e** as a pale grey solid in 71% yield (312 mg).

^{1}H NMR (300 MHz, acetone-*d_6*) δ 7.75 (d, *J* = 8.6 Hz, 4H), 7.60 (d, *J* = 8.6 Hz, 4H), 5.51 (dd, *J* = 13.6, 3.1 Hz, 2H), 3.91 (dd, *J* = 16.0, 13.6 Hz, 2H), 3.23 (dd, *J* = 16.0, 3.1 Hz, 2H), 2.92 (s, 3H). **^{13}C NMR** (76 MHz, acetone-*d_6*) δ 199.2, 137.3, 131.8, 131.7, 131.1, 59.0, 46.1, 21.6. **^{19}F NMR** (471 MHz, acetone-*d_6*) δ -149.10. **HRMS** (ESI): *m/z* calculated for $C_{18}H_{17}Cl_2OS$ $[M-BF_4]^+$: 351.0372; found 351.0367.

(2*R*,6*S*)-2,6-bis(4-bromophenyl)-1-methyl-4-oxohexahydrothiopyrylium tetrafluoroborate (1f)

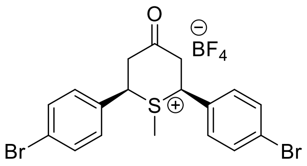

Following general procedure B, (1*E*,4*E*)-1,5-bis(4-bromophenyl)penta-1,4-dien-3-one (1 mmol, 392 mg) gave sulfonium salt **1f** as a white solid in 83% yield (438 mg).

^{1}H NMR (300 MHz, acetone-*d_6*) δ 7.73 (d, *J* = 8.5 Hz, 1H), 7.65 (d, *J* = 8.5 Hz, 1H), 5.47 (dd, *J* = 13.6, 3.2 Hz, 2H), 3.88 (dd, *J* = 16.2, 13.6 Hz, 2H), 3.21 (dd, *J* = 16.2, 3.2 Hz, 2H), 2.90 (s, 3H). **^{13}C NMR** (76 MHz, acetone-*d_6*) δ 199.1, 134.0, 132.2, 131.8, 125.5, 58.9, 46.0, 21.5. **^{19}F NMR** (471 MHz, acetone-*d_6*) δ -149.36. **HRMS** (ESI): *m/z* calculated for $C_{18}H_{17}Br_2OS$ $[M-BF_4]^+$: 438.9361; found 438.9362.

(2*R*,6*S*)-2,6-bis(4-methoxyphenyl)-1-methyl-4-oxohexahydrothiopyrylium tetrafluoroborate (1g)

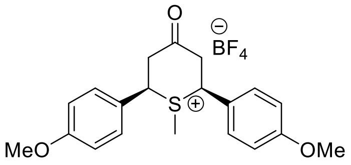

Following general procedure B, (1*E*,4*E*)-1,5-bis(4-methoxyphenyl)penta-1,4-dien-3-one (1 mmol, 294 mg) gave sulfonium salt **1g** as a reddish solid in 58% yield (251 mg).

^{1}H NMR (300 MHz, acetone-*d_6*) δ 7.64 (d, *J* = 8.7 Hz, 4H), 7.08 (d, *J* = 8.7 Hz, 4H), 5.35 (dd, *J* = 13.5, 3.0 Hz, 1H), 3.92 (dd, *J* = 15.6, 13.5 Hz, 2H), 3.86 (s, 6H), 3.12 (dd, *J* = 15.6, 3.0 Hz, 2H), 2.78 (s, 3H). **^{13}C NMR** (76 MHz, acetone-*d_6*) δ 200.0, 162.4, 131.2, 124.6, 116.2, 59.2, 56.0, 46.5, 21.2. **^{19}F NMR** (471 MHz, acetone-*d_6*) δ -150.17. **HRMS** (ESI): *m/z* calculated for $C_{20}H_{23}O_3S$ $[M-BF_4]^+$: 343.1362; found 343.1357.

(2*R*,6*S*)-1-methyl-4-oxo-2,6-di-*p*-tolylhexahydrothiopyrylium tetrafluoroborate (1h)

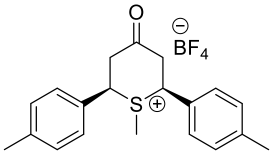

Following general procedure B, (1*E*,4*E*)-1,5-di-*p*-tolylpenta-1,4-dien-3-one (1 mmol, 262 mg) gave sulfonium salt **1h** as an orange solid in 69% yield (274 mg).

^{1}H NMR (300 MHz, acetone-*d_6*) δ 7.60 (d, *J* = 8.2 Hz, 4H), 7.37 (d, *J* = 8.2 Hz, 4H), 5.40 (dd, *J* = 13.6, 3.0 Hz, 2H), 3.90 (dd, *J* = 16.1, 13.7 Hz, 2H), 3.15 (dd, *J* = 16.2, 3.0 Hz, 2H), 2.78 (s, 3H), 2.38 (s, 6H). **^{13}C NMR** (76 MHz, acetone-*d_6*) δ 199.8, 141.9, 131.5, 130.0, 129.6, 59.4, 46.4, 21.4, 21.3. **^{19}F NMR** (471 MHz, acetone-*d_6*) δ -149.54. **HRMS** (ESI): *m/z* calculated for $C_{20}H_{23}OS$ $[M-BF_4]^+$: 311.1464; found 311.1461.

(2*R*,6*S*)-1-methyl-4-oxo-2,6-di-*m*-tolylhexahydrothiopyrylium tetrafluoroborate (1i)

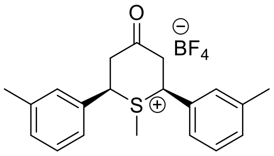

Following general procedure B, (1*E*,4*E*)-1,5-di-*m*-tolylpenta-1,4-dien-3-one (1 mmol, 262 mg) gave sulfonium salt **1i** as an orange solid in 73% yield (291 mg).

^{1}H NMR (500 MHz, acetone-d_6) δ 7.56 (s, 2H), 7.50 (d, *J* = 7.6 Hz, 2H), 7.44 (t, *J* = 7.6 Hz, 2H), 7.37 (d, *J* = 7.6 Hz, 2H), 5.40 (dd, *J* = 13.6, 3.3 Hz, 1H), 3.90 (dd, *J* = 16.1, 13.3 Hz, 1H), 3.18 (dd, *J* = 16.1, 3.3 Hz, 1H), 2.80 (s, 3H), 2.39 (s, 6H). **^{13}C NMR** (76 MHz, acetone-d_6) δ 199.6, 140.7, 132.8, 132.1, 130.5, 129.9, 126.5, 59.1, 46.1, 26.9, 21.2. **^{19}F NMR** (471 MHz, acetone-d_6) δ -149.27. **HRMS** (ESI): *m/z* calculated for $C_{20}H_{23}OS$ $[M-BF_4]^+$: 311.1464; found 311.1459.

(2*R*,6*S*)-1-methyl-4-oxo-2,6-di-*o*-tolylhexahydrothiopyrylium tetrafluoroborate (1j)

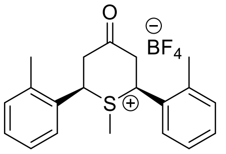

Following general procedure B, (1*E*,4*E*)-1,5-di-*o*-tolylpenta-1,4-dien-3-one (1 mmol, 262 mg) gave sulfonium salt **1j** as an orange solid in 52% yield (207 mg).

^{1}H NMR (300 MHz, acetone-d_6) δ 7.93 – 7.84 (m, 2H), 7.53 – 7.28 (m, 6H), 5.87 (dd, *J* = 13.3, 3.4 Hz, 2H), 3.78 (dd, *J* = 16.3, 13.4 Hz, 2H), 3.15 (dd, *J* = 16.3, 3.4 Hz, 2H), 2.83 (s, 3H), 2.53 (s, 6H). **^{13}C NMR** (76 MHz, acetone-d_6) δ 199.6, 138.4, 132.6, 131.7, 131.0, 128.8, 128.6, 55.6, 46.5, 20.9, 19.6. **^{19}F NMR** (471 MHz, acetone-d_6) δ -149.98. **HRMS** (ESI): *m/z* calculated for $C_{20}H_{23}OS$ $[M-BF_4]^+$: 311.1464; found 311.1460.

(2*R*,6*S*)-1-methyl-2,6-di(naphthalen-2-yl)-4-oxohexahydrothiopyrylium tetrafluoroborate (1k)

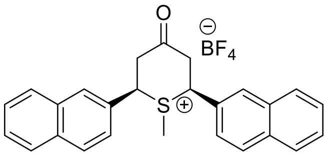

Following general procedure B, (1*E*,4*E*)-1,5-di(naphthalen-2-yl)penta-1,4-dien-3-one (1 mmol, 334 mg) gave sulfonium salt **1k** as a white solid in 66% yield (311 mg).

^{1}H NMR (300 MHz, acetone-d_6) δ 8.32 (d, *J* = 2.0 Hz, 2H), 8.11 (d, *J* = 8.6 Hz, 2H), 8.03 – 7.96 (m, 4H), 7.86 (dd, *J* = 8.6, 2.0 Hz, 2H), 7.66 – 7.58 (m, 4H), 5.71 (dd, *J* = 13.6, 3.2 Hz, 2H), 4.12 (dd, *J* = 16.0, 13.6 Hz, 2H), 3.31 (dd, *J* = 15.9, 3.2 Hz, 2H), 2.90 (s, 3H). **^{13}C NMR** (76 MHz, acetone-d_6) δ 199.7, 135.1, 134.3, 131.1, 130.3, 130.3, 129.4, 128.9, 128.7, 128.2, 125.6, 60.1, 46.5, 21.7. **^{19}F NMR** (471 MHz, acetone-d_6) δ -149.33. **HRMS** (ESI): *m/z* calculated for $C_{26}H_{23}OS$ $[M-BF_4]^+$: 383.1464; found 383.1455.

(2*R*,6*S*)-2,6-di([1,1'-biphenyl]-4-yl)-1-methyl-4-oxohexahydrothiopyrylium tetrafluoroborate (1l)

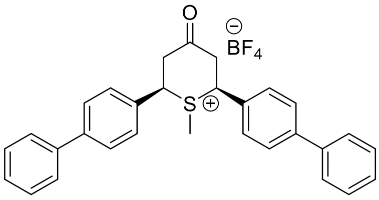

Following general procedure B, (1*E*,4*E*)-1,5-di([1,1'-biphenyl]-4-yl)penta-1,4-dien-3-one (1 mmol, 386 mg) gave sulfonium salt **1l** as a white solid in 74% yield (387 mg).

^{1}H NMR (300 MHz, acetone-d_6) δ 7.96 – 7.80 (m, 8H), 7.80 – 7.63 (m, 4H), 7.56 – 7.44 (m, 4H), 7.47 – 7.35 (m, 2H), 5.55 (dd, *J* = 13.6, 3.4 Hz, 2H), 4.19 – 3.81 (dd, *J* = 16.7, 13.6 Hz, 2H), 3.25 (dd, *J* = 16.7, 3.4 Hz, 2H), 2.90 (s, 3H). **^{13}C NMR** (76 MHz, acetone-d_6) δ 199.7, 144.2, 140.4, 131.9, 130.3, 130.0, 129.2, 129.1, 127.9, 59.4, 46.3, 21.6. **^{19}F NMR** (471 MHz, acetone-d_6) δ -149.46. **HRMS** (ESI): *m/z* calculated for $C_{30}H_{27}OS$ $[M-BF_4]^+$: 435.1777; found 435.1785.

3. Synthesis and characterization data of sulfonium salt 4

3.1. General procedure C for the synthesis of sulfonium salt 4

The total synthesis of sulfonium salts **5** is a sequential synthesis that consists in 3 steps. The following scheme shows the global synthesis and intermediates prepared.

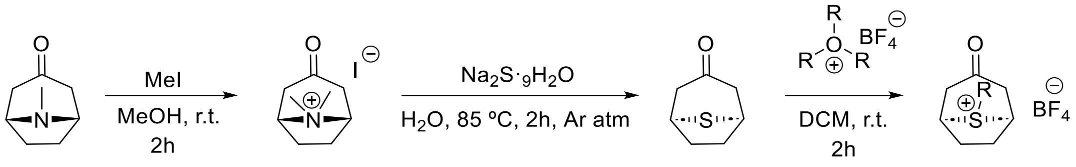

Following a described procedure in the literature,[13] to a solution of tropinone (1.0 equiv., 10 mmol, 1.4 g) in methanol (8.5 mL), methyl iodide (1.3 equiv, 13 mmol, 0.8 mL) was added in one portion. The reaction was stirred at room temperature for 2h. Then, the solid was filtered, washed with methanol and recrystallized in methanol:water to obtain 8,8-dimethyl-3-oxo-8-azoniabicyclo[3.2.1]octane iodide as a white solid in 87% yield (8.7 mmol, 2.4 g).

Then, 8,8-dimethyl-3-oxo-8-azoniabicyclo[3.2.1]octane iodide (1.0 equiv., 3.8 mmol, 1.1 g) was added to a solution of sodium sulfide nonahydrate (1.2 equiv., 4.6 mmol, 1.1 g) in water (80 mL) under argon atmosphere. The reaction was stirred for 2h at 85 °C. Next, the crude was extracted with diethyl ether (2x20 mL). The combination of organic phases was treated with HCl 0.1M and then washed with brine until neutrality (pH=7). The organic phase was dried over $MgSO_4$, filtered, and concentrated under reduced pressure. Then, the solid was passed through a short flash column (stationary phase: 2 cm of basic alumina; eluent: diethyl ether) to obtain (1*R*,5*S*)-8-thiabicyclo[3.2.1]octan-3-one as a white solid in 83% yield (3.2 mmol, 455 mg).

Finally, in a round-bottomed flask trialkyloxonium tetrafluoroborate (1.1 equiv., 1.1 mmol) was suspended in DCM (4 mL) under Argon atmosphere, and the suspension was flushed with Argon for 10 min. Then, a solution of (1*R*,5*S*)-8-thiabicyclo[3.2.1]octan-3-one (1.0 equiv., 1 mmol, 142 mg) in DCM (6 mL) was added to the reaction crude. The reaction was stirred for 2 hours at room temperature. After the indicated time, diethyl ether was added to the reaction crude observing the appearance of turbidity. Once the turbidity was persistent, the reaction crude was dropped into a beaker with 20 mL of diethyl ether to precipitate the sulfonium salt. After filtration, (the corresponding salt **5** was dried under vacuum and kept in the fridge. The salt was obtained pure without any further purification.

Finally, in a round-bottomed flask trimethyloxonium tetrafluoroborate (1.1 equiv., 1.1 mmol, 162.7 mg) was suspended in DCM (4 mL) under Argon atmosphere, and the suspension was flushed with Argon for 10 min. Then, a solution of (1*R*,5*S*)-8-thiabicyclo[3.2.1]octan-3-one (1.0 equiv., 1 mmol, 142 mg) in DCM (6 mL) was added to the reaction crude. The reaction was stirred for 2 hours at room temperature. After the indicated time, diethyl ether was added to the reaction crude observing the appearance of turbidity. Once the turbidity was persistent, the reaction crude was dropped into a beaker with 20 mL of diethyl ether to precipitate the sulfonium salt. After filtration, (1*R*,5*S*)-8-methyl-3-oxo-8-thiabicyclo[3.2.1]octan-8-ium tetrafluoroborate **4** was dried under vacuum and kept in the fridge. The salt was obtained pure without any further purification.

3.2. Characterization data of sulfonium salt 4

(1*R*,5*S*)-8-methyl-3-oxo-8-thiabicyclo[3.2.1]octan-8-ium tetrafluoroborate (4a)

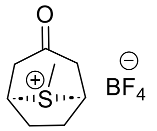

Following general procedure C, trimethyloxonium tetrafluoroborate (1.1 equiv., 1.1 mmol, 162.7 mg) gave sulfonium salt **4a** as a white solid in 70% yield (170 mg). **^{1}H NMR** (300 MHz, acetone-*d$_6$*) δ 4.89 – 4.75 (m, 2H), 3.24 (dd, *J* = 18.6, 1.6 Hz, 2H), 3.04 (s, 3H), 2.99 (dd, *J* = 18.6, 3.8 Hz, 2H), 2.91 – 2.86 (m, 2H), 2.62 – 2.47 (m, 2H). **^{13}C NMR** (76 MHz, acetone-*d$_6$*) δ 201.7, 56.8, 48.1, 29.9, 19.7. **^{19}F NMR** (471 MHz, acetone-*d$_6$*) δ -151.24. **HRMS** (ESI): *m/z* calculated for $C_8H_{13}OS$ $[M-BF_4]^+$: 157.0682; found 157.0681.

(1*R*,5*S*)-8-ethyl-3-oxo-8-thiabicyclo[3.2.1]octan-8-ium tetrafluoroborate (4b)

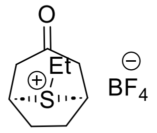

Following general procedure C, triethyloxonium tetrafluoroborate (1.1 equiv., 1.1 mmol, 162.7 mg) gave sulfonium salt **4b** as a white solid in 73% yield (188 mg). **^{1}H NMR** (300 MHz, acetone-*d$_6$*) δ 4.87 (s, 2H), 3.44 (q, *J* = 7.5 Hz, 2H), 3.24 (d, *J* = 18.1 Hz, 2H), 2.99 (d, *J* = 18.1 Hz, 2H), 2.88 – 2.71 (m, 2H), 2.60 – 2.32 (m, 2H), 1.59 (t, *J* = 7.5 Hz, 3H). **^{13}C NMR** (76 MHz, acetone-*d$_6$*) δ 201.86, 55.58 (2C), 48.05 (2C), 31.96, 30.28 (2C), 9.97. **^{19}F NMR** (471 MHz, acetone-*d$_6$*) δ -151.31. **HRMS** (ESI): **HRMS** (ESI): *m/z* calculated for $C_9H_{15}OS$ $[M-BF_4]^+$: 171.0838; found 171.0843.

4. Screening of reaction conditions

Supplementary Table 1: Optimization table

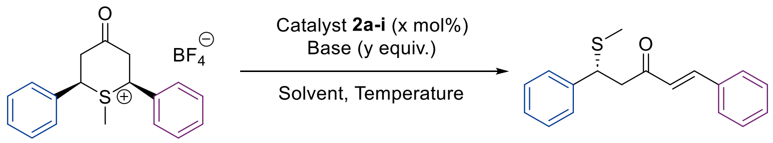

1a **3a**

Catalysts

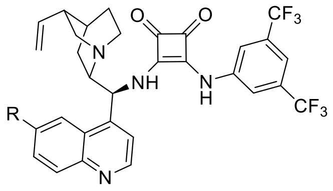

2a R = OMe
2b R = H

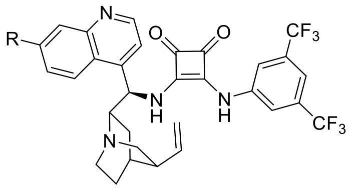

2c R = OMe
2d R = H

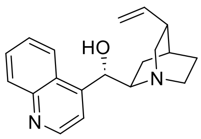

2f

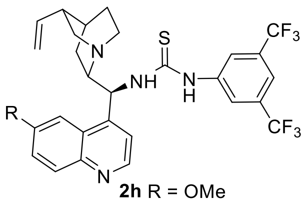

2h R = OMe
2i R = H

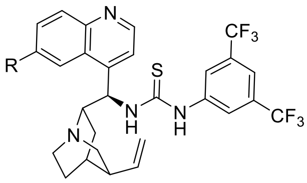

2e R = OMe
2g R = H

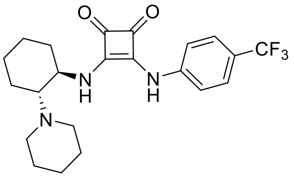

2j

Entry	Catalyst (x mol%)	Base (y equiv.)	Solvent	Temperature	Time	Yield	*er*
**1**	-	TMP (1.2 equiv.)	DCM (0.2 M)	r.t.	16h	95%	-
**2**	-	Na_2CO_3 (1.2 equiv.)	DCM (0.2 M)	r.t.	16h	93%	-
**3**	-	$NaHCO_3$ (1.2 equiv.)	DCM (0.2 M)	r.t.	16h	n.r.	-
**4**	-	NaOAc (1.2 equiv)	DCM (0.2 M)	r.t.	16h	91%	-
**5**	-	$NaH_2PO_4 \cdot 2H_2O$ (1.2 equiv)	DCM (0.2 M)	r.t.	16h	n.r.	-
**6**	**2a** (20 mol%)	$NaHCO_3$ (1.2 equiv.)	DCM (0.2 M)	r.t.	16h	87%	15:85
**7**	**2a** (20 mol%)	$NaH_2PO_4 \cdot 2H_2O$ (1.2 equiv)	DCM (0.2 M)	r.t.	16h	69%	31.3:68.7
**8**	**2b** (20 mol%)	$NaHCO_3$ (1.2 equiv.)	DCM (0.2 M)	r.t.	16h	92%	35:65
**9**	**2c** (20 mol%)	$NaHCO_3$ (1.2 equiv.)	DCM (0.2 M)	r.t.	16h	93%	90.3:9.7
**10**	**2d** (20 mol%)	$NaHCO_3$ (1.2 equiv.)	DCM (0.2 M)	r.t.	16h	88%	91.4:8.6
**11**	**2e** (20 mol%)	$NaHCO_3$ (1.2 equiv.)	DCM (0.2 M)	r.t.	16h	76%	90.3:9.7
**12**	**2f** (20 mol%)	$NaHCO_3$ (1.2 equiv.)	DCM (0.2 M)	r.t.	16h	84%	64.3:35:7
**13**	**2g** (20 mol%)	$NaHCO_3$ (1.2 equiv.)	DCM (0.2 M)	r.t.	16h	78%	71:29

**14**	**2h** (20 mol%)	$NaHCO_3$ (1.2 equiv.)	DCM (0.2 M)	r.t.	16h	85%	40:60
**15**	**2i** (20 mol%)	$NaHCO_3$ (1.2 equiv.)	DCM (0.2 M)	r.t.	16h	82%	12:88
**16**	**2j** (20 mol%)	$NaHCO_3$ (1.2 equiv.)	DCM (0.2 M)	r.t.	16h	87%	50:50
**17**	**2d** (10 mol%)	$NaHCO_3$ (1.2 equiv.)	DCM (0.2 M)	r.t.	16h	78%	90:10
**18**	**2d** (5 mol%)	$NaHCO_3$ (1.2 equiv.)	DCM (0.2 M)	r.t.	16h	75%	90:10
**19**	**2d** (1 mol%)	$NaHCO_3$ (1.2 equiv.)	DCM (0.2 M)	r.t.	16h	69%	87:13
**20**	**2d** (20 mol%)	$NaHCO_3$ (1.2 equiv.)	$CHCl_3$ (0.2 M)	r.t.	16h	90%	95:5
**21**	**2d** (20 mol%)	$NaHCO_3$ (1.2 equiv.)	THF (0.2 M)	r.t.	16h	87%	94.4:5.6
**22**	**2d** (20 mol%)	$NaHCO_3$ (1.2 equiv.)	Toluene (0.2 M)	r.t.	16h	79%	94:6
**23**	**2d** (20 mol%)	$NaHCO_3$ (1.2 equiv.)	C_6F_6 (0.2 M)	r.t.	16h	82%	89:11
**24**	**2d** (20 mol%)	$NaHCO_3$ (1.2 equiv.)	$CHCl_3$ (0.2 M) H2O (0.2M)	r.t.	16h	88%	95:5
**25**	**2d** (20 mol%)	$NaHCO_3$ (1.2 equiv.)	$CHCl_3$ (0.2 M)	-20 °C	16h	65%	92.7:7.3
**26**	**2d** (20 mol%)	$NaHCO_3$ (1.2 equiv.)	$CHCl_3$ (0.2 M)	-10 °C	16h	72%	96:4
**27**	**2d** (20 mol%)	$NaHCO_3$ (1.2 equiv.)	$CHCl_3$ (0.2 M)	0 °C	16h	77%	96:4
**28**	**2d** (20 mol%)	$NaHCO_3$ (1.2 equiv.)	$CHCl_3$ (0.2 M)	5 °C	16h	83%	95.4:4.6
**29**	**2d** (20 mol%)	$NaHCO_3$ (1.2 equiv.)	$CHCl_3$ (0.2 M)	10 °C	16h	87%	95.6:4.4
**30**	**2d** (20 mol%)	$NaHCO_3$ (1.2 equiv.)	$CHCl_3$ (0.2 M)	40 °C	16h	96%	71:29
**31**	**2d** (20 mol%)	$NaHCO_3$ (1 equiv.)	$CHCl_3$ (0.2 M)	r.t.	16h	84%	94:6
**32**	**2d** (20 mol%)	$NaHCO_3$ (0.8 equiv.)	$CHCl_3$ (0.2 M)	r.t.	16h	84%	89:11

5. General Procedures for the Enantioselective Desymmetrization

5.1. General procedure C and D for the Enantioselective Desymmetrization of sulfonium salts 1a-l and 4

Procedure C

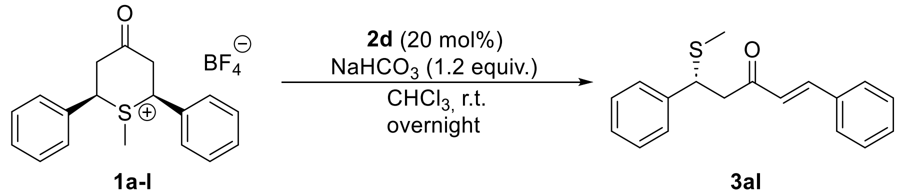

1a-l **3al**

Procedure D

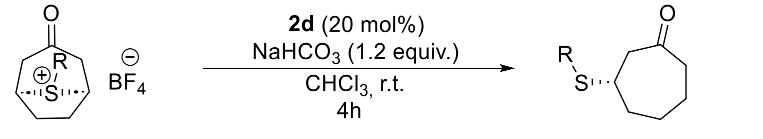

4 **5**

Procedure D: enantioselective desymmetrization of sulfonium salts 1a-l

The corresponding sulfonium salt (1 equiv., 0.05 mmol), sodium bicarbonate (1.2 equiv., 0.06 mmol, 5 mg) and **2d** (0.2 equiv., 0.01 mmol, 6 mg) were dissolved in chloroform (0.25 mL) and stir at room temperature overnight. Then, diethyl ether was added (2 mL) and the reaction crude was filtered.[14] The filtrate was concentrated under reduced pressure and purified by flash chromatography.

Procedure E: enantioselective desymmetrization of sulfonium salts 4

Sulfonium salt **4** (1 equiv., 0.05 mmol, 12.2 mg), sodium bicarbonate (1.2 equiv., 0.06 mmol, 5 mg) and **2d** (0.2 equiv., 0.01 mmol, 6 mg) were dissolved in chloroform (0.25 mL) and stir at room temperature for 4 hours. Then, diethyl ether was added (2 mL) and the reaction crude was filtered.[7] The filtrate was concentrated under reduced pressure and purified by flash chromatography.

5.2. Characterization data of 3a-l and 5

(*R*,*E*)-5-(methylthio)-1,5-diphenylpent-1-en-3-one (3a)

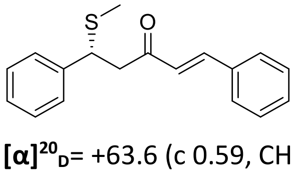

Following general procedure D, sulfonium salt **1a** (1 equiv., 0.05 mmol, 18 mg) gave **3a** as a white solid in 90% yield (13 mg).

$[\alpha]^{20}_{D}$= +63.6 (c 0.59, $CHCl_3$). **^{1}H NMR** (300 MHz, acetone-*d_6*) δ 7.68 (dt, *J* = 6.1, 2.0 Hz, 2H), 7.47 – 7.37 (m, 5H), 7.37 – 7.26 (m, 3H), 7.27 – 7.16 (m, 1H), 6.86 (d, *J* = 16.3 Hz, 1H), 4.39 (t, *J* = 7.5 Hz, 1H), 3.40 (dt, *J* = 16.6, 7.5 Hz, 1H), 3.33 (dd, *J* = 16.6, 7.0 Hz, 1H), 1.91 (s, 3H). **^{13}C NMR** (76 MHz, acetone-*d_6*) δ 197.2, 143.4, 143.1, 135.9, 131.4, 130.0, 129.4, 129.3, 129.0, 128.0, 127.4, 47.2, 46.8, 14.5. **HRMS** (ESI): *m/z* calculated for $C_{18}H_{19}OS$ $[M+H]^+$: 283.1151; found 283.1158. The enantiomeric ratio was determined by SFC using a Chiralpak IB column [CO_2/MeOH 98:2 in 10 min, flow rate 2.0 mL/min], τ_{major} = 4.67 min, τ_{minor} = 4.29 min (**e.r.** = 95:5).

The reaction was scaled up to 0.2 mmol. Procedure D was followed using a 72 mg of sulfonium salt **1a**. After workup and purification as described above, **3a** (54mg, 92% yield) was obtained as a white solid. The enantiomeric ratio was determined by SFC using a Chiralpak IB column [CO_2/MeOH 98:2 in 10 min, flow rate 2.0 mL/min], τ_{major} = 4.67 min, τ_{minor} = 4.29 min (**e.r.** = 92:8).

The reaction was scaled up to 1.0 mmol. Procedure D was followed using a 360 mg of sulfonium salt **1a**. After workup and purification as described above, **3a** (265mg, 93% yield) was obtained as a white solid. The enantiomeric ratio was determined by SFC using a Chiralpak IB column [CO_2/MeOH 98:2 in 10 min, flow rate 2.0 mL/min], τ_{major} = 4.67 min, τ_{minor} = 4.29 min (**e.r.** = 89:11).

Dimethyl 4,4'-(5-(methylthio)-3-oxopent-1-ene-1,5-diyl)(*R*,*E*)-dibenzoate (3b)

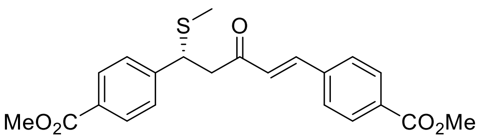

Following general procedure D, sulfonium salt **1a** (1 equiv., 0.05 mmol, 24 mg) gave **3a** as an orange solid in 90% yield (18 mg).

[α]$^{20}_{D}$= +13.1 (c 1.49, $CHCl_3$). **^{1}H NMR** (300 MHz, acetone-*d$_6$*) δ 8.03 (d, *J* = 8.4 Hz, 2H), 7.96 (d, *J* = 8.4 Hz, 2H), 7.81 (d, *J* = 8.4 Hz, 2H), 7.70 (d, *J* = 16.3 Hz, 1H), 7.56 (d, *J* = 8.4 Hz, 2H), 6.99 (d, *J* = 16.3 Hz, 1H), 4.47 (dd, *J* = 8.0, 6.7 Hz, 1H), 3.90 (s, 3H), 3.86 (s, 3H), 3.48 (dd, *J* = 16.8, 8.0 Hz, 1H), 3.38 (dd, *J* = 16.8, 6.7 Hz, 1H), 1.93 (s, 3H).**^{13}C NMR** (76 MHz, acetone-*d$_6$*) δ 196.9, 167.1, 166.8, 148.6, 142.1, 140.2, 132.5, 130.8, 130.4, 130.1, 129.4, 129.3, 129.2, 52.7, 52.4, 46.9, 46.3, 14.4. **HRMS** (ESI): *m/z* calculated for $C_{22}H_{23}O_5S$ $[M+H]^+$: 399.1261; found 399.1246. The enantiomeric ratio was determined by SFC using a Chiralpak IB column [CO_2/MeOH 95:5 in 15 min, flow rate 2.0 mL/min], τ_{major} = 6.60 min, τ_{minor} = 7.16 min (**e.r.** = 92.1:7.9).

(*R*,*E*)-5-(methylthio)-1,5-bis(4-(trifluoromethyl)phenyl)pent-1-en-3-one (3c)

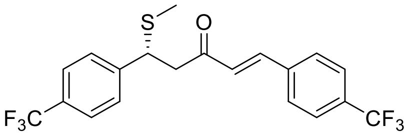

Following general procedure D, sulfonium salt **1c** (1 equiv., 0.05 mmol, 25 mg) gave **3c** as a white solid in 90% yield (19 mg).

[α]$^{20}_{D}$= +26.6 (c 1.18, $CHCl_3$). **^{1}H NMR** (300 MHz, $CDCl_3$) δ 7.69-7.57 (m, 4H), 7.60 – 7.55 (m, 3H), 7.52 (d, *J* = 8.4 Hz, 2H), 6.73 (d, *J* = 16.2 Hz, 1H), 4.41 (t, *J* = 7.2 Hz, 1H), 3.30 (dd, *J* = 16.8, 6.6 Hz, 1H), 3.22 (dd, *J* = 16.8, 7.8 Hz, 1H), 1.94 (s, 3H).**^{13}C NMR** (76 MHz, $CDCl_3$) δ 195.9, 145.7, 141.4, 137.6, 132.2 (q, *J* = 32.8 Hz), 129.4 (q, *J* = 32.1 Hz), 128.5, 128.2, 127.7, 125.9 (q, *J* = 3.8 Hz), 125.6 (q, *J* = 3.7 Hz), 124.0 (q, *J* = 272.0 Hz), 123.7 (q, *J* = 271.2 Hz), 47.2, 45.5, 14.8. **HRMS** (ESI): *m/z* calculated for $C_{20}H_{17}F_6OS$ $[M+H]^+$: 419.0899; found 419.0937. The enantiomeric ratio was determined by SFC using a Chiralpak IG column [CO_2/MeOH from 95:5 to 60:40 in 8 min, flow rate 2.0 mL/min], τ_{major} = 3.72 min, τ_{minor} = 3.46 min (**e.r.** = 88.7:11.3).

(*R*,*E*)- 1,5-bis(4-fluorophenyl)-5-(methylthio)pent-1-en-3-one (3d)

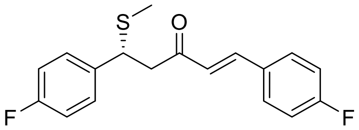

Following general procedure D, sulfonium salt **1d** (1 equiv., 0.05 mmol, 20 mg) gave **3d** as a white solid in 82% yield (13 mg).

[α]$^{20}_{D}$= -22.7 (c 0.26, $CHCl_3$). **^{1}H NMR** (300 MHz, acetone-*d$_6$*) δ 7.75 (dd, *J* = 8.6, 5.6 Hz, 2H), 7.64 (d, *J* = 16.3 Hz, 1H), 7.45 (dd, *J* = 8.6, 5.6 Hz, 2H), 7.21 (t, *J* = 8.8 Hz, 2H), 7.07 (t, *J* = 8.8 Hz, 2H), 6.82 (d, *J* = 16.3 Hz, 1H), 4.40 (dd, *J* = 7.8, 6.6 Hz, 1H), 3.35 (dd, *J* = 16.4, 7.8 Hz, 1H), 3.26 (td, *J* =

16.4, 6.6 Hz, 1H), 1.91 (s, 3H). ^{13}**C NMR** (76 MHz, acetone-d_6) δ 196.9, 164.9 (d, J = 249.3 Hz), 162.7 (d, J = 243.4 Hz), 142.3, 142.2, 139.2, 132.4 (d, J = 3.3 Hz), 131.6 (d, J = 8.2 Hz), 130.8 (d, J = 8.0 Hz), 127.2, 126.6 (d, J = 2.7 Hz), 116.9 (d, J = 22.1 Hz), 115.9 (d, J = 21.4 Hz), 47.2, 46.0, 14.5. **HRMS** (ESI): *m/z* calculated for $C_{18}H_{17}F_2OS$ $[M+H]^+$: 319.0963; found 399.0972. The enantiomeric ratio was determined by SFC using a Chiralpak ID column [CO_2/MeOH from 95:5 to 60:40 in 8 min, flow rate 2.0 mL/min], τ_{major} = 1.60 min, τ_{minor} = 1.41 min (**e.r.** = 89.9:10.1).

(*R*,*E*)- 1,5-bis(4-chlorophenyl)-5-(methylthio)pent-1-en-3-one (3e)

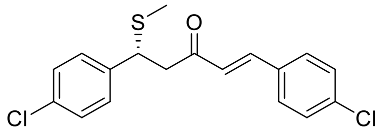

Following general procedure D, sulfonium salt **1e** (1 equiv., 0.05 mmol, 22 mg) gave **3e** as a white solid in 95% yield (17 mg).

[α]$^{20}_{D}$= +42.1 (c 0.98, $CHCl_3$). ^{1}**H NMR** (300 MHz, acetone-d_6) δ 7.74 – 7.70 (m, 2H), 7.65 (d, J = 16.3 Hz, 1H), 7.50 – 7.47 (m, 2H), 7.47 – 7.44 (m, 2H), 7.39 – 7.34 (m, 2H), 6.89 (d, J = 16.3 Hz, 1H), 4.41 (dd, J = 8.2, 6.5 Hz, 1H), 3.39 (dd, J = 16.7, 8.2 Hz, 1H), 3.32 (dd, J = 16.7, 6.5 Hz, 1H), 1.95 (s, 3H). ^{13}**C NMR** (76 MHz, acetone-d_6) δ 196.9, 142.2, 142.1, 136.7, 134.7, 133.2, 131.0, 130.9, 130.7, 130.1, 130.1, 129.3, 127.9, 47.1, 46.1, 14.5. **HRMS** (ESI): *m/z* calculated for $C_{18}H_{17}Cl_2OS$ $[M+H]^+$: 351.0372; found 351.0381. The enantiomeric ratio was determined by SFC using a Chiralpak ID column [CO_2/MeOH from 95:5 to 60:40 in 8 min, flow rate 2.0 mL/min], τ_{major} = 2.98 min, τ_{minor} = 2.48 min (**e.r.** = 93.6:6.4).

(*R*,*E*)- 1,5-bis(4-bromophenyl)-5-(methylthio)pent-1-en-3-one (3f)

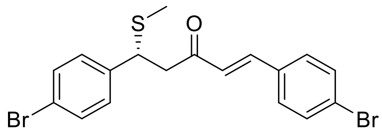

Following general procedure D, sulfonium salt **1f** (1 equiv., 0.05 mmol, 26 mg) gave **3f** as a white solid in 97% yield (21 mg).

[α]$^{20}_{D}$= +79.3 (c 1.15, $CHCl_3$). ^{1}**H NMR** (300 MHz, acetone-d_6) δ 7.67 – 7.57 (m, 5H), 7.52 – 7.46 (m, 2H), 7.42 – 7.33 (m, 2H), 6.89 (d, J = 16.3 Hz, 1H), 4.37 (t, J = 7.4 Hz, 1H), 3.39 (dd, J = 16.7, 8.0 Hz, 1H), 3.28 (dd, J = 16.7, 6.8 Hz, 1H), 1.92 (s, 3H). ^{13}**C NMR** (76 MHz, acetone-d_6) δ 196.9, 142.6, 142.1, 135.1, 133.1, 133.1, 132.3, 131.2, 131.1, 131.0, 128.0, 125.0, 121.2, 47.0, 46.1, 14.4. **HRMS** (ESI): *m/z* calculated for $C_{18}H_{17}Br_2OS$ $[M+H]^+$: 438.9361; found 438.9372. The enantiomeric ratio was determined by SFC using a Chiralpak ID column [CO_2/MeOH from 95:5 to 60:40 in 8 min, flow rate 2.0 mL/min], τ_{major} = 4.16 min, τ_{minor} = 3.38 min (**e.r.** = 92.6:7.4).

(*R*,*E*)- 1,5-bis(4-methoxyphenyl)-5-(methylthio)pent-1-en-3-one (3g)

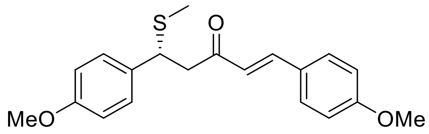

Following a modified procedure D,[15] sulfonium salt **1g** (1 equiv., 0.05 mmol, 21 mg) gave **3g** as an orange solid in 92% yield (16 mg).

[α]$^{20}_{D}$= +36.4 (c 1.3, $CHCl_3$). ^{1}**H NMR** (300 MHz, acetone-d_6) δ 7.66 – 7.62 (m, 2H), 7.59 (d, J = 16.3 Hz, 1H), 7.36 – 7.30 (m, 2H), 7.02 – 6.96 (m, 2H), 6.89 – 6.85 (m, 2H), 6.72 (d, J = 16.3 Hz, 1H), 4.35 (dd, J = 8.2, 6.5 Hz, 1H), 3.86 (s, 3H), 3.77 (s, 3H), 3.28 (dd, J = 16.2, 8.2 Hz, 1H), 3.21 (dd, J = 16.2, 6.5 Hz, 1H), 1.90 (s, 3H). ^{13}**C NMR** (76 MHz, acetone-d_6) δ 197.1, 162.8, 159.9, 143.3, 134.9, 131.1, 130.0, 128.4, 125.2, 115.4, 114.6, 55.9, 55.6, 47.3, 46.4, 14.5. **HRMS** (ESI): *m/z* calculated for $C_{20}H_{23}O_3S$ $[M+H]^+$: 343.1362; found 343.1389. The enantiomeric ratio was

determined by SFC using a Chiralpak ID column [CO_2/MeOH from 95:5 to 60:40 in 8 min, flow rate 2.0 mL/min], τ_{major} = 4.64 min, τ_{minor} = 3.83 min (**e.r.** = 88.7:11.3).

(*R*,*E*)-5-(methylthio)-1,5-di-*p*-tolylpent-1-en-3-one (3h)

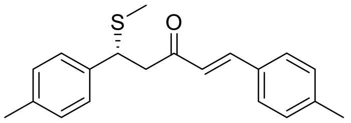

Following general procedure D, sulfonium salt **1h** (1 equiv., 0.05 mmol, 20 mg) gave **3h** as a yellowish solid in 91% yield (14 mg).

[α]$^{20}_{D}$= +24.5 (c 1.0, $CHCl_3$). **^{1}H NMR** (300 MHz, acetone-*d$_6$*) δ 7.65 – 7.52 (m, 3H), 7.33 – 7.20 (m, 4H), 7.12 (d, *J* = 7.9 Hz, 2H), 6.80 (d, *J* = 16.3 Hz, 1H), 4.35 (t, *J* = 7.3 Hz, 1H), 3.32 (dd, *J* = 16.5, 7.8 Hz, 1H), 3.22 (dd, *J* = 16.5, 6.8 Hz, 1H), 2.35 (s, 3H), 2.27 (s, 3H), 1.90 (s, 3H). **^{13}C NMR** (76 MHz, acetone-*d$_6$*) δ 197.2, 143.4, 141.8, 140.0, 137.4, 133.1, 130.6, 129.9, 129.4, 128.8, 126.4, 47.2, 46.6, 21.5, 21.1, 14.5. **HRMS** (ESI): *m/z* calculated for $C_{20}H_{23}OS$ $[M+H]^+$: 311.1464; found 311.1471. The enantiomeric ratio was determined by SFC using a Chiralpak ID column [CO_2/MeOH 90:10 in 15 min, flow rate 2.0 mL/min], τ_{major} = 2.17 min, τ_{minor} = 1.19 min (**e.r.** = 87:13).

(*R*,*E*)-5-(methylthio)-1,5-di-*m*-tolylpent-1-en-3-one (3i)

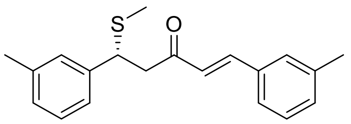

Following general procedure D, sulfonium salt **1i** (1 equiv., 0.05 mmol, 20 mg) gave **3i** as a white solid in 86% yield (13 mg).

[α]$^{20}_{D}$= +16.6 (*c* 0.67, $CHCl_3$). **^{1}H NMR** (300 MHz, acetone-*d$_6$*) δ 7.59 (d, *J* = 16.2 Hz, 1H), 7.49 (s, 1H), 7.46 (d, *J* = 7.6 Hz, 1H), 7.31 (t, *J* = 7.6 Hz, 1H), 7.25 (d, *J* = 5.7 Hz, 2H), 7.22 – 7.16 (m, 2H), 7.07 – 7.00 (m, 1H), 6.84 (d, *J* = 16.2 Hz, 1H), 4.34 (dd, *J* = 7.8, 6.8 Hz, 1H), 3.33 (dd, *J* = 16.4, 7.8 Hz, 1H), 3.27 (dd, *J* = 16.4, 6.8 Hz, 1H), 2.35 (s, 3H), 2.31 (s, 3H), 1.91 (s, 3H). **^{13}C NMR** (76 MHz, acetone-*d$_6$*) δ 197.2, 143.6, 143.0, 139.6, 138.7, 135.9, 132.2, 129.9, 129.9, 129.6, 129.2, 128.8, 127.3, 126.6, 126.0, 47.2, 46.9, 21.6, 21.4, 14.6 **HRMS** (ESI): *m/z* calculated for $C_{20}H_{23}OS$ $[M+H]^+$: 311.1464; found 311.1473. The enantiomeric ratio was determined by SFC using a Chiralpak IG column [CO_2/MeOH from 95:5 to 60:40 in 8 min, flow rate 2.0 mL/min], τ_{major} = 5.42 min, τ_{minor} = 6.63 min (**e.r.** = 91.5:8.5).

(*R*,*E*)-5-(methylthio)-1,5-di-*o*-tolylpent-1-en-3-one (3j)

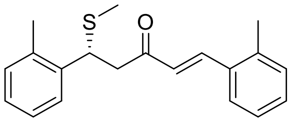

Following general procedure D, sulfonium salt **1j** (1 equiv., 0.05 mmol, 20 mg) gave **3j** as a yellowish solid in 85% yield (13 mg).

^{1}H NMR (300 MHz, acetone-*d$_6$*) δ 7.89 (d, *J* = 16.0 Hz, 1H), 7.66 (d, *J* = 7.5 Hz, 1H), 7.44 (d, *J* = 7.5 Hz, 1H), 7.34 – 7.22 (m, 3H), 7.21 – 7.08 (m, 3H), 6.77 (d, *J* = 16.0 Hz, 1H), 4.68 (dd, *J* = 8.2, 6.3 Hz, 1H), 3.48 (dd, *J* = 16.7, 8.2 Hz, 1H), 3.37 (dd, *J* = 16.7, 6.3 Hz, 1H), 2.46 (s, 3H), 2.44 (s, 3H), 1.96 (s, 3H).**^{13}C NMR** (76 MHz, acetone-*d$_6$*) δ 197.6, 140.7, 140.6, 138.9, 137.1, 134.5, 131.7, 131.2, 131.1, 128.3, 127.7, 127.5, 127.3, 127.3, 127.0, 47.0, 41.8, 19.8, 19.8, 14.0. **HRMS** (ESI): *m/z* calculated for $C_{20}H_{23}OS$ $[M+H]^+$: 311.1464; found 311.1471. The enantiomeric ratio was determined by SFC using a Chiralpak IB column [CO_2/MeOH from 95:5 to 70:30 in 8 min, flow rate 2.0 mL/min], τ_{major} = 1.93 min, τ_{minor} = 1.79 min (**e.r.** = 50:50).

(*R*,*E*)-5-(methylthio)-1,5-di(naphthalen-2-yl)pent-1-en-3-one (3k)

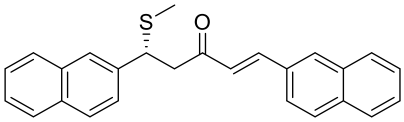

Following general procedure D, sulfonium salt **1k** (1 equiv., 0.05 mmol, 23 mg) gave **3k** as a white solid in 85% yield (16 mg).

[α]$^{20}_{D}$= +58.7 (c 1.20, $CHCl_3$). **^{1}H NMR** (300 MHz, acetone-*d$_6$*) δ 8.14 (s, 1H), 7.97 – 7.83 (m, 8H), 7.82 – 7.79 (m, 1H), 7.66 (dd, *J* = 8.6, 1.8 Hz, 1H), 7.59 – 7.53 (m, 2H), 7.50 – 7.45 (m, 2H), 7.01 (d, *J* = 16.2 Hz, 1H), 4.60 (dd, *J* = 8.0, 6.7 Hz, 1H), 3.54 (dd, *J* = 16.5, 8.0 Hz, 1H), 3.43 (dd, *J* = 16.5, 6.7 Hz, 1H), 1.94 (s, 3H). **^{13}C NMR** (76 MHz, acetone-*d$_6$*) δ 197.1, 143.5, 140.3, 135.4, 134.5, 134.2, 133.8, 133.4, 131.3, 131.3, 129.7, 129.6, 129.2, 128.7, 128.6, 128.3, 127.8, 127.6, 127.1, 127.1, 127.0, 126.8, 124.7, 47.1, 47.0, 14.4. **HRMS** (ESI): *m/z* calculated for $C_{26}H_{23}OS$ [M+H]$^+$: 383.1464; found 383.1465. The enantiomeric ratio was determined by SFC using a Chiralpak ID column [CO_2/MeOH 90:10 in 15 min, flow rate 2.0 mL/min], τ_{major} = 10.51 min, τ_{minor} = 9.68 min (**e.r.** = 82:18).

(*R*,*E*)-1,5-di([1,1'-biphenyl]-4-yl)-5-(methylthio)pent-1-en-3-one (3l)

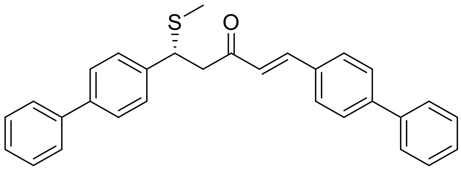

Following general procedure D, sulfonium salt **1l** (1 equiv., 0.05 mmol, 26 mg) gave **3l** as a white solid in 93% yield (20 mg).

[α]$^{20}_{D}$= +46.3 (c 0.63, $CHCl_3$). **^{1}H NMR** (300 MHz, acetone-*d$_6$*) δ 7.80 – 7.77 (m, 2H), 7.77 – 7.67 (m, 5H), 7.68 – 7.60 (m, 4H), 7.56 – 7.50 (m, 2H), 7.52 – 7.45 (m, 2H), 7.44 (d, *J* = 6.3 Hz, 2H), 7.43 – 7.36 (m, 1H), 7.37 – 7.30 (m, 1H), 6.93 (d, *J* = 16.2 Hz, 1H), 4.46 (dd, *J* = 8.2, 6.6 Hz, 1H), 3.44 (dd, *J* = 16.4, 8.2 Hz, 1H), 3.36 (dd, *J* = 16.4, 6.6 Hz, 1H), 1.97 (s, 3H). **^{13}C NMR** (76 MHz, acetone-*d$_6$*) δ 197.1, 143.9, 143.0, 142.3, 141.6, 141.0, 140.8, 135.0, 130.1, 130.0, 130.0, 129.8, 129.5, 128.9, 128.4, 128.4, 128.3, 127.9, 127.9, 127.8, 127.3, 47.2, 46.6, 14.6. **HRMS** (ESI): *m/z* calculated for $C_{30}H_{27}OS$ [M+H]$^+$: 435.1777; found 435.1769. The enantiomeric ratio was determined by SFC using a Chiralpak IB column [CO_2/MeOH from 95:5 to 60:40 in 15 min, flow rate 2.0 mL/min], τ_{major} = 6.28 min, τ_{minor} = 6.55 min (**e.r.** = 92.2:7.8).

(*S*)-6-(methylthio)cyclohept-2-en-1-one (5a)

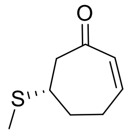

Following general procedure E, sulfonium salt **4a** (1 equiv., 0.05 mmol, 12 mg) gave **5a** as a white solid in 94% yield (7 mg).

[α]$^{20}_{D}$= -28.5 (c 0.38, $CHCl_3$). **^{1}H NMR** (300 MHz, acetone-*d$_6$*) δ 6.70 (dt, *J* = 11.6, 5.4 Hz, 1H), 6.08 (d, *J* = 11.8 Hz, 1H), 3.20 – 3.10 (m, 1H), 3.00 (dd, *J* = 14.7, 4.6 Hz, 1H), 2.81 (dd, *J* = 14.6, 9.1 Hz, 1H), 2.74 – 2.60 (m, 1H), 2.59 – 2.40 (m, 1H), 2.28 – 2.19 (m, 1H), 2.17 (s, 3H), 2.06 – 1.93 (m, 1H). **^{13}C NMR** (76 MHz, acetone-*d$_6$*) δ 200.8, 147.1, 132.9, 49.3, 40.5, 33.3, 27.9, 14.6. **HRMS** (ESI): *m/z* calculated for $C_8H_{13}OS$ [M+H]$^+$: 157.0682; found 157.0680. The enantiomeric ratio was determined by SFC using a Chiralpak ID column [CO_2/MeOH 95:5 in 15 min, flow rate 2. 0 mL/min], τ_{major} = 1.36 min, τ_{minor} = 1.49 min (**e.r.** = 74.6:25.4).

(*S*)-6-(ethylthio)cyclohept-2-en-1-one (5b)

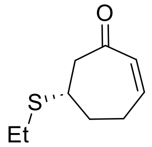

Following general procedure E, sulfonium salt **4b** (1 equiv., 0.05 mmol, 12 mg) gave **5b** as a white solid in 87% yield (7 mg).

[α]$^{20}_{D}$= -14.8 (c 0.24, $CHCl_3$). **^{1}H NMR** (300 MHz, $CDCl_3$) δ 6.66 (ddd, *J* = 12.0, 6.1, 4.7 Hz, 1H), 6.04 (ddt, *J* = 12.0, 2.2, 1.1 Hz, 1H), 3.23 (ddt, *J* = 9.2, 6.7, 4.7 Hz, 1H), 2.95 (dd, *J* = 14.7, 4.7 Hz, 1H), 2.77 (dd, *J* = 14.7, 9.2 Hz, 1H), 2.71 – 2.62 (m, 1H), 2.58 (q, *J* = 7.4 Hz, 2H), 2.52 – 2.36 (m, 1H), 2.24 – 2.07 (m, 1H), 2.05 – 1.85 (m, 1H), 1.26 (t, *J* = 7.4 Hz, 3H). **^{13}C NMR** (76 MHz, $CDCl_3$) δ 200.9, 147.2, 133.0, 49.8, 38.4, 33.7, 28.0, 25.2, 14.8. **HRMS** (ESI): *m/z* calculated for $C_8H_{15}OS$ $[M+H]^+$: 171.0838; found 171.0831. The enantiomeric ratio was determined by SFC using a Chiralpak ID column [CO_2/MeOH 95:5 in 15 min, flow rate 2. 0 mL/min], τ_{major} = 2.25 min, τ_{minor} = 2.51 min (**e.r.** = 71:29).

6. General procedure E: derivatization of 3a to 6

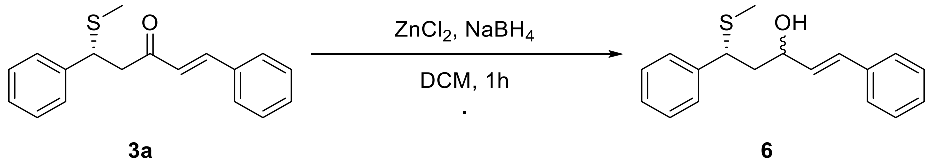

(*R*)-3a-[95:5 e.r.] (1. equiv., 0.05 mmol, 14 mg), anhydrous zinc chloride (1.2 equiv., 0.06 mmol, 8 mg) and sodium borohydride (1.2 equiv., 0.06, 2 mg) were weighted in a vial and dissolved in dichloromethane (0.4 mL). The reaction was stirred at room temperature for 1h. Then, HCl 1M (2 mL) was added to the reaction crude and it was extracted with diethyl ether (3x1mL). The combination of organic phases was dried over $MgSO_4$, filtered, and concentrated under reduced pressure. The residue was purified by flash chromatography (eluent: cyclohexane: ethyl acetate, 10:1), obtaining **6** as a mixture of two diastereoisomers (d.r. 4.1:1). **6** was obtained as a white oil in 75% yield (11 mg).

^{1}H NMR (300 MHz, $CDCl_3$) δ 7.39 – 7.29 (m, 8H, major and minor), 7.29 – 7.22 (m, 2H, major and minor), 6.54 (d, *J* = 16.0 Hz, 1H, major and minor), 6.18 (dd, *J* = 16.0, 6.8 Hz, 1H, major and minor), 4.52 – 4.38 (m, 1H, minor diastereoisomer), 4.30 – 4.14 (m, 1H, major diastereoisomer), 3.95 (dd, *J* = 9.0, 6.3 Hz, 1H, major and minor), 2.31 – 2.14 (m, 1H, major and minor), 2.10 (ddd, *J* = 13.8, 9.0, 4.2 Hz, 1H, major and minor), 1.90 (s, 3H, major diastereoisomer), 1.87 (s, 3H, minor diastereoisomer). **^{13}C NMR** (76 MHz, $CDCl_3$) δ 142.3, 141.9, 136.7, 131.9, 131.6, 131.2, 130.6, 128.8, 128.7, 128.1, 128.0, 127.9, 127.4, 127.3, 126.7, 126.6, 71.0, 71.0, 48.0, 47.7, 43.2, 14.4, 14.2. **HRMS** (ESI): *m/z* calculated for $C_{18}H_{21}OS$ $[M+H]^+$: 285.1308; found 285.1301. The diastereomeric ratio and the enantiomeric ratio were determined by SFC using a Chiralpak IB column [CO_2/MeOH 95:5 in 10 min, flow rate 2. 0 mL/min]; **d.r.** 4.1:1; major diastereoisomer: τ_{major} = 9.43 min, τ_{minor} = 5.78 min (**e.r.** = 94.7:5.3); minor diastereoisomer: τ_{major} = 8.76 min, τ_{minor} = 7.75 min (**e.r.** = 94.7:5.3).

7. General procedure F: derivatization of (*R*)-3a to 7

(*R*)-3-(2-(methylthio)-2-phenylethyl)-5-phenyl-1-tosyl-1*H*-pyrazole (7)

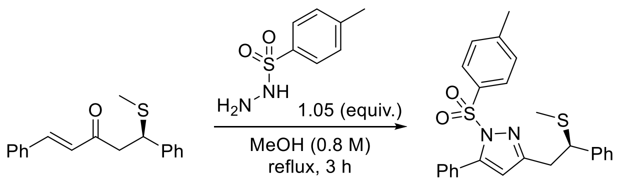

(*R*)-3a-[90:10 e.r.] (0.05 mmol, 1.0 equiv.) and 4-methylbenzenesulfonohydrazide (1.05 equiv.) were dissolved in MeOH (0.8 M) and refluxed for 3 hours. After that time, the mixture was concentrated, and the residue was dissolved in DCM. The organic layer was washed with saturated aqueous $NaHCO_3$ and brine. The organic layer was dried with Na_2SO_4, filtered, and evaporated to give a solid residue, which was further purified by flash column chromatography (Cy:EtOAc gradient from 100:0 to 90:10) to yield the final product (92% yield, 90:10 e.r.).
*The final product was characterized along with an inseparable 30% of its reduced analogue 3-((*R*)-2-(methylthio)-2-phenylethyl)-5-phenyl-1-tosyl-4,5-dihydro-1*H*-pyrazole (**7'**).

^{1}H NMR (300 MHz, $CDCl_3$) δ 7.73 (d, *J* = 8.4 Hz, 2H), 7.44 – 7.27 (m, 12H), 6.75 (s, 1H), 3.84 (t, *J* = 7.1 Hz, 1H), 3.12 (dd, *J* = 14.3, 7.1 Hz, 1H), 2.96 (dd, *J* = 14.3, 7.1 Hz, 1H), 2.43 (s, 3H), 1.84 (s, 3H). **^{13}C NMR** (126 MHz, $CDCl_3$) δ154.7, 144.1, 141.0, 135.9, 135.4, 134.2, 129.5 (2C), 129.4 (2C), 129.0 (2C), 128.6, 128.5, 128.4 (2C), 127.4 (2C), 127.2 (2C), 116.4, 49.2, 34.1, 21.8, 15.4. **HRMS** (ESI): *m/z* calculated for $C_{25}H_{25}N_2O_2S_2$ $[M+H]^+$: 449.1352; found 449.1356. The enantiomeric ratio was determined by SFC using a Chiralpak IG column [CO_2/MeOH 75:25 in 20 min, flow rate 2.0 mL/min]; t_{major} = 14.76 min, t_{minor} = 17.65 min (e.r. = 90:10).

3-((*R*)-2-(methylthio)-2-phenylethyl)-5-phenyl-1-tosyl-4,5-dihydro-1*H*-pyrazole (**7'**)

^{1}H NMR (300 MHz, $CDCl_3$) δ 7.84 (d, *J* = 8.3 Hz, 2H), 7.45 – 7.28 (m, 8H), 7.22 – 7.13 (m, 4H), 6.86 (d, *J* = 16.4 Hz, 1H), 6.76 – 6.74 (m, 1H), 6.71 (d, *J* = 16.4 Hz, 1H), 4.04 (t, *J* = 7.5 Hz, 1H), 3.06 – 3.01 (m, 2H), 2.44 (s, 3H), 1.80 (s, 3H). **^{13}C NMR** (126 MHz, $CDCl_3$) δ 144.2, 141.5, 138.8, 135.6, 135.2, 129.7 (2C), 129.1 (2C), 129.0 (2C), 128.7, 128.2, 128.0 (2C), 127.7 (2C), 127.6 (2C), 49.0, 47.2, 46.3, 40.2, 30.5, 29.8. **HRMS** (ESI): *m/z* calculated for $C_{25}H_{27}N_2O_2S_2$ $[M+H]^+$: 451.1508; found 451.1499.

8. General procedure F: derivatization of (*R*)-3a to 8

(*R*)-3-(methylthio)-3-phenyl-1-(4-phenyl-1*H*-pyrrol-3-yl)propan-1-one (8)

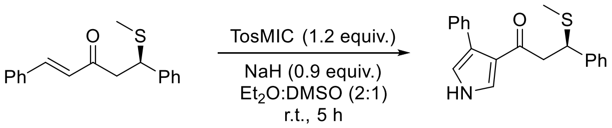

(*R*)-3a-[85:15 e.r.] (0.05 mmol, 1.0 equiv.) and TosMIC (1.2 equiv.) were dissolved in Et_2O:DMSO (2:1, 0.3 mL) and added dropwise to a vial containing NaH (0.9 equiv.) in Et_2O (0.1 mL). After that time, water was added to the crude and the aqueous layer was extracted with Et_2O (3 x 0.3 mL). The combined organic layer was dried over anhydrous Na_2SO_4, filtered, and evaporated to give a solid residue, which was further purified by flash column chromatography (Cy:EtOAc gradient from 90:10 to 75:25) to yield the final product (88% yield, 84:16 e.r.).

$[\alpha]^{20}_D$= +34.1 (c 0.20, $CHCl_3$). **^{1}H NMR** (300 MHz, $CDCl_3$) δ 7.42 – 7.27 (m, 10H), 7.23 – 7.13 (m, 1H), 6.74 (t, *J* = 2.3 Hz, 1H), 4.39 (t, *J* = 7.2 Hz, 1H), 3.25 (dd, *J* = 16.1, 7.1 Hz, 1H), 3.19 (dd, *J* = 16.1, 7.4 Hz, 1H), 1.87 (s, 3H). **^{13}C NMR** (126 MHz, $CDCl_3$) δ 193.0, 142.1, 135.1, 129.4 (2C), 128.6 (2C), 128.1 (2C), 128.0 (2C), 127.3, 126.9, 126.4, 125.2, 123.6, 119.3, 47.0, 46.5, 14.9. **HRMS** (ESI): *m/z* calculated for $C_{20}H_{20}NOS$ $[M+H]^+$: 322.1260; found 322.1253. The enantiomeric ratio was determined by SFC using a Chiralpak IA column [CO_2/MeOH from 95:5 to 60:40 in 8 min, flow rate 3.0 mL/min], t_{minor} = 6.14min, t_{major} = 6.87 min (e.r. = 17:83).

9. General procedure F: derivatization of (*R*)-3d to 9

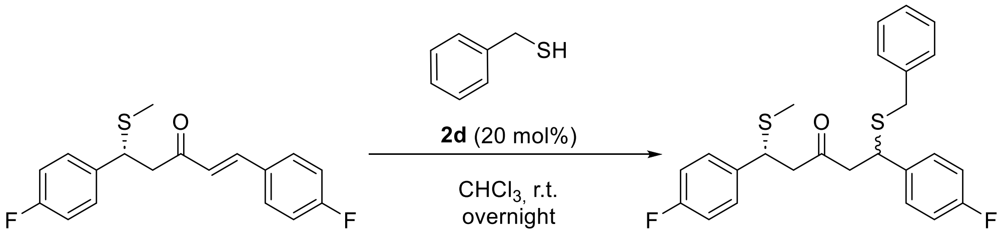

(*R*)-3d **7**

Isolation of single enantiomer of (*R*)-3d: the two enantiomers were separated from 60 mg of a racemic mixture of **3d** in a Chiralflash IA (30 mm ø x 100 mmL, particle size 20 µm) column using a Biotage Isolera Prime® (eluent: Hexane:*i*PrOH, 95:5; flow 1 ml/min). The e.r. of each enantiomer was determined by SFC using the conditions described for **3d** and it was determined as >99%.

(*R*)-3d (1.0 equiv., 0.05 mmol, 16 mg), **2d** (0.2 equiv., 0.01 mmol, 6 mg) and benzyl mercaptan (2 equiv., 0.1 mmol, 11.7 µL) were dissolved in chloroform (0.3 mL). The reaction was stirred at room temperature overnight. Then, the reaction crude was concentrated under reduced pressure. The residue was purified by flash chromatography (eluent: cyclohexane: ethyl acetate, 20:1), obtaining **7** as a mixture of two diastereoisomers (d.r. 9:1). **9** was obtained as a white oil in 87% yield (18 mg).

^{1}H NMR (300 MHz, $CDCl_3$) δ 7.37 – 7.30 (m, 3H, major and minor), 7.30 – 7.24 (m, 4H, major and minor), 7.22 – 7.16 (m, 2H, major and minor), 7.06 – 7.03 (m, 2H, major and minor), 7.04 – 7.00 (m, 2H, major and minor), 4.25 – 4.07 (m, 2H, minor and minor), 3.54 (d, *J* = 13.4 Hz, 1H, major and minor), 3.44 (d, *J* = 13.6 Hz, 1H, major and minor), 2.93 (ddd, *J* = 17.0, 8.4, 1.6 Hz, 2H, major and minor), 2.82 (ddd, *J* = 17.1, 6.2, 3.6 Hz, 2H, major and minor), 1.90 (s, 3H, minor diastereoisomer), 1.87 (s, 3H, major diastereoisomer). **^{13}C NMR** (76 MHz, $CDCl_3$) δ 203.7 (major), 203.5 (minor), 162.9 (d, *J* = 3.6 Hz), 160.9 (d, *J* = 3.5 Hz), 137.6 (minor), 137.5, 137.0 (d, *J* = 3.2 Hz), 136.95 (d, *J* = 3.2 Hz), 129.52 (d, *J* = 8.2 Hz, major), 129.4 (d, *J* = 8.1 Hz, minor), 129.29 (d, *J* = 8.2 Hz, major), 129.16 (d, *J* = 8.1 Hz, minor), 128.9 (minor), 128.9 (major), 128.6 (minor), 128.5 (major), 127.1 (major), 127.1 (minor), 115.49 (d, *J* = 3.4 Hz, major), 115.44 (d, *J* = 2.9 Hz, minor), 115.32 (d, *J* = 3.4 Hz, major), 115.27 (d, *J* = 2.9 Hz), 50.0, 49.9 (major), 49.8 (minor), 49.6 (major), 44.8 (minor), 44.7 (major), 42.9 (major), 42.8 (minor), 35.7 (major), 35.7 (minor), 14.6 (major), 14.5 (minor). **^{19}F NMR** (471 MHz, acetone-*d_6*) δ -114.82 (major), -114.86 (minor), -114.95 (major), -115.01 (minor). **HRMS** (ESI): *m/z* calculated for $C_{25}H_{27}OS_2$ $[M+H]^+$: 407.1498; found 407.1502. The diastereomeric ratio and the enantiomeric ratio were determined by SFC using a Chiralpak IG column [CO_2/MeOH 98:2 in 30 min, flow rate 2.0 mL/min]; **d.r.** 9:1; major diastereoisomer: τ_{major} = 9.71 min, τ_{minor} = 5.78 min (**e.r.** = 94.7:5.3); minor diastereoisomer: τ_{major} = 9.10 min, τ_{minor} = 7.75 min (**e.r.** = 94.7:5.3).

10. NMR studies.

10.1. Titration experiments. Procedure for the titration experiments

Two standard solutions of catalyst (6 mM in $CDCl_3$) and $TBAHCO_3$ (tetrabutylammonium bicarbonate) (30 mM in $CDCl_3$) were prepared the same day that the experiments were run out. The ^{1}H NMR spectra were obtained using a *Bruker Avance 300 MHz spectrometer* with an accumulation of 100 scans in each experiment. In supplementary table 2 there are summed up the quantities used in each NMR tube to ensure a final amount of 0.6 mL so that the concentration of the catalyst was maintained constant, and it was fixed at 2 mM. The volume was measured using micropipettes or Hamilton syringes.

Supplementary Table 2: amounts of volumes used in each tube.

entry	Equiv. $TBAHCO_3$	V (µL) catalyst standard solution	V (µL) $TBAHCO_3$ standard solution	V (µL) $CDCl_3$	Total volume (µL)
**1**	0	200	0	400	600
**2**	0.1	200	4	396	600
**3**	0.3	200	12	388	600
**4**	0.5	200	20	380	600
**5**	0.7	200	28	372	600
**6**	0.9	200	36	364	600
**7**	1	200	40	360	600
**8**	2	200	80	320	600
**9**	5	200	200	200	600
**10**	10	200	400	0	600

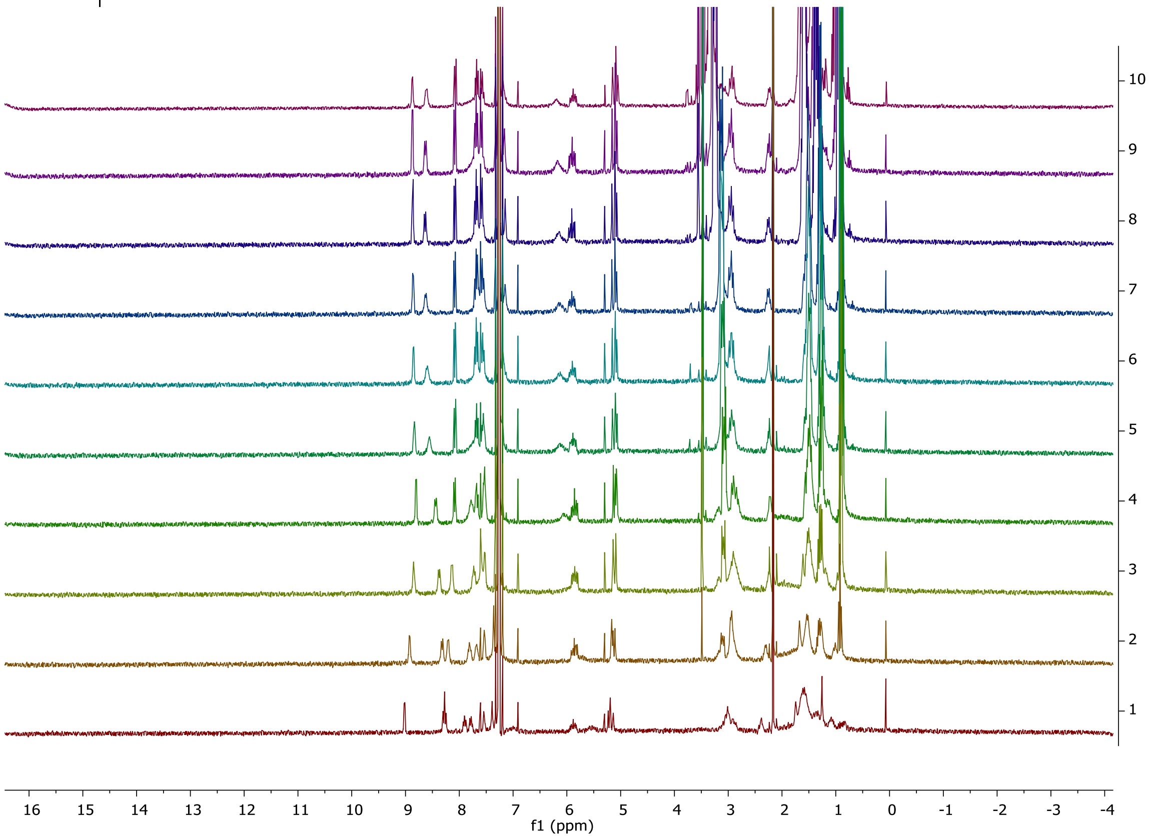

10.2. Titration experiments. Determination of the binding constant

Since the anion recognition of the squaramide unit occurs through the N-H bonds, the identification of these signals is essential for determining the association constant. The N-H signals were easily identified adding some drops of D_2O to an NMR tube containing only the catalyst as the signals disappear (7.00 and 5.56 ppm).

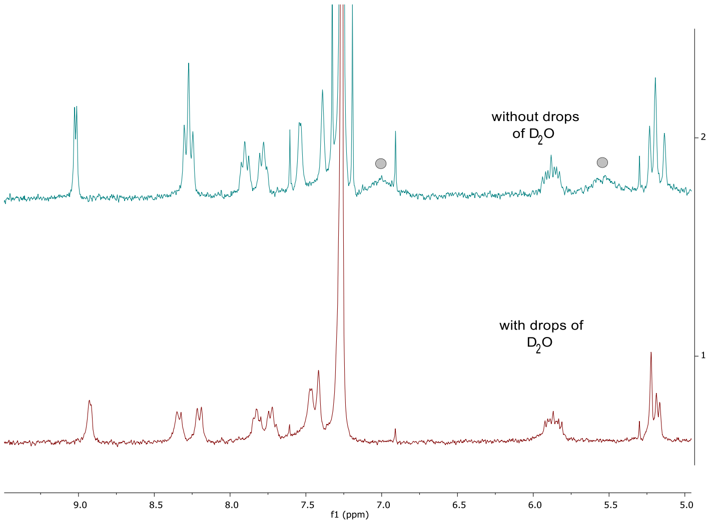

A downfield shift of the signals of the N-H of the squaramide moiety was clearly appreciated even when low quantities of the $TBAHCO_3$ were added. Unfortunately, one of the signals of the N-H overlapped with other signals of the spectra and it could not be used for the analysis of the association constant. The determination of the association constant was carried out with one of the NH of the squaramide core using BindFit.[16,17] Calculations were performed using model Nelder-Mead and a stoichiometry catalyst:bicarbonate 1:1. However, the result could not be considered a good approximation since the analysis was just made with one signal and the error was considerable. We can only estimate that the association constant is high (K_a=126098±992M^{-1}) what is consistent to the fact that even when low quantities of $TBAHCO_3$ were used (0.1 equiv.) there was a noticeable shift in the NH signals. Nevertheless, these results confirmed that an anion-binding process was taking place and that the squaramide formed a host-guest complex with the bicarbonate.

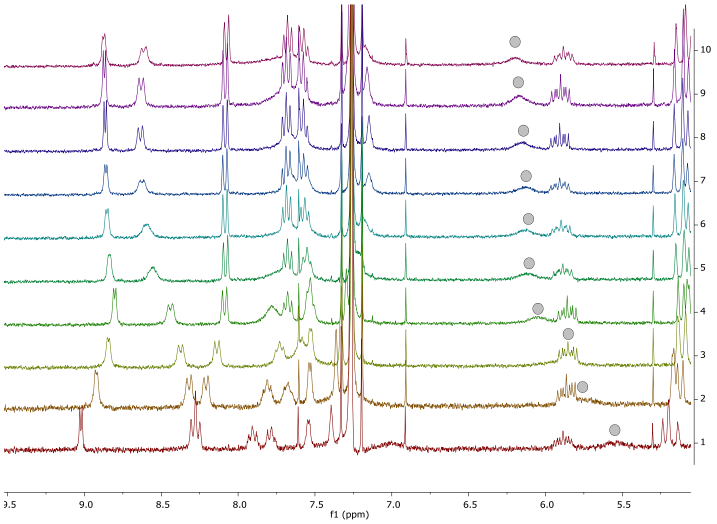

[Cat]	[TBAHCO$_3$]	ΔH_{NH}(ppm)
0,002	0	0
0,002	0,0002	0,03
0,002	0,0006	0,09
0,002	0,001	0,16
0,002	0,0014	0,28
0,002	0,0018	0,32
0,002	0,002	0,35
0,002	0,004	0,35
0,002	0,01	0,35
0,002	0,02	0,35

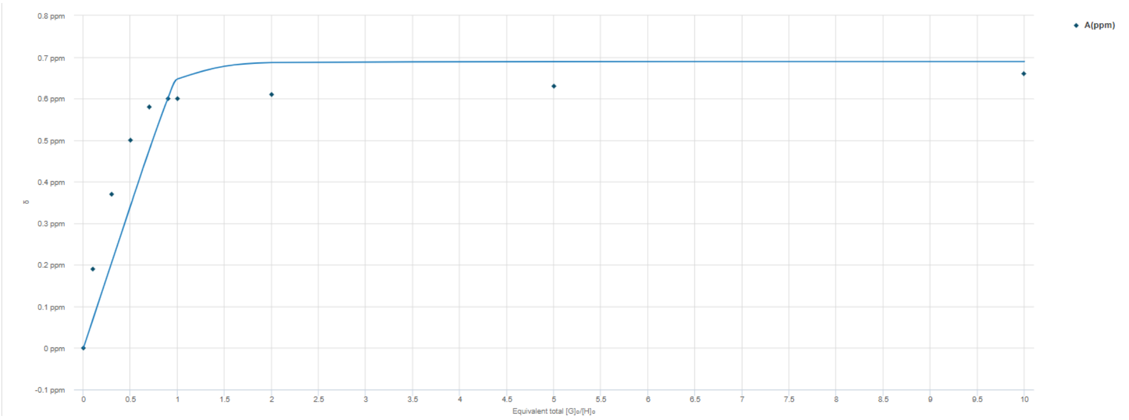

In addition, we have identified that protons at 8.3 ppm are allocated at carbon 5 and 8 of the quinoline core. Those protons are equivalents and therefore appear as a sole triplet for two protons. However, and in presence of the anion, that signal split in two doublets clearly differentiated. This may support our hypothesis corroborating the fact that the catalyst environment is clearly modified when bonded to the anion. In TS shown in Figure 4 of the manuscript, we can see how proton 5 and 8 of the quinoline are not equivalent anymore due to the disposition of the catalyst with respect to the bicarbonate. Supplementary Figure 7 shows an interaction between hydrogen of C5 of the quinoline and the quinoline ring. This interaction together with the rigidity acquired might help to differentiate them.

10.3. ^{13}C-NMR.

The ^{13}C NMR spectra of a mixture 1:1 of the catalyst **2d** and TBAHCO$_3$ (tetrabutylammonium bicarbonate) in CDCl$_3$ (final amount of 0.6 mL) were obtained using a *Bruker Avance 300 MHz spectrometer*. The volume was measured using micropipettes or Hamilton syringes.

As observed in figure below, the carbon signal of the HCO_3^- was shifted downfield in the presence of 1.0 equiv. of TBAHCO$_3$.

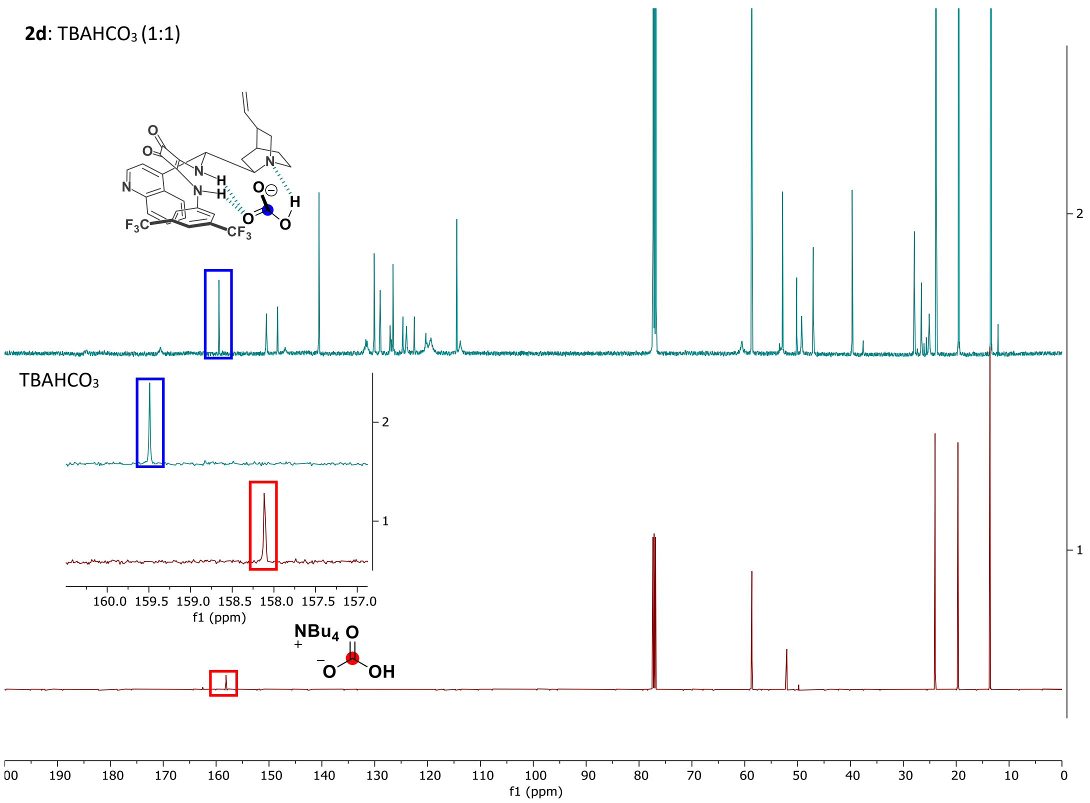

Moreover, the NMR studies showed a great solubility effect (see below) that might emphasize the interaction between the catalyst and the HCO_3^-.

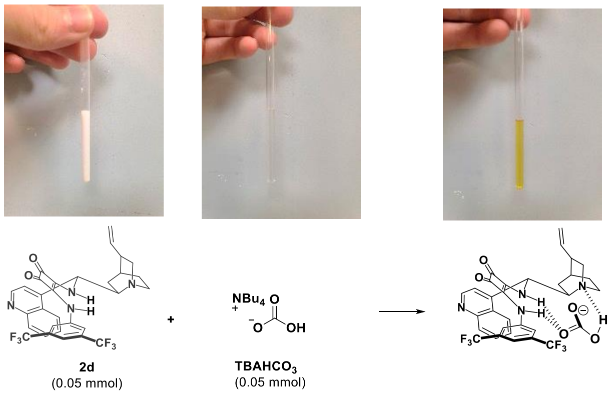

11. Computational studies

11.1. Mechanism of the reaction: anion-binding process

Based on experimental evidence, we decided to gain further information regarding the mechanism of the reaction and the origin of the enantioselectivity.

Firstly, we considered the possibility of two different modes of coordination of the sulfonium salt and bicarbonate to the catalyst, as shown in Supplementary Figure 1. In order to corroborate or to discard this hypothesis, we have carried out an exhaustive exploration of the potential energy surface of the anion-substrate-catalyst system using the CREST software. Considering an energy window of 15 kcal·mol^{-1}, 3041 different structures were found. Among these huge amount of conformers, only in two of them a change in the coordination site was found (hydrogen bond formed with the carbonyl group of the substrate instead with the bicarbonate). The most stable of these structures was found 7.9 kcal·mol^{-1} above the energy of the global minimum. Hence, this binding mode does not play a significant role in the chemical process studied in this work.

Supplementary Figure 1: General structures for the two possible modes of coordination.

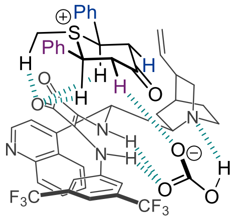

I

Anion-binding mode of coordination

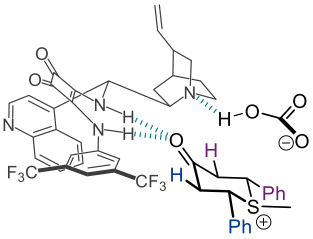

Bifunctional mode of coordination

Our proposal for the mechanism of the reaction starts with the formation of a host-guest complex between the organocatalyst and the bicarbonate anion (**II$_{eq}$**). Then, the sulfonium cation interacts with one of the carbonyl moieties of the squaramide by hydrogen bonding, forming the chiral contact ion pair (**I**). At this point, the bicarbonate is capable of deprotonate one of the enantiotopic protons of the sulfonium cation and leading to the formation of **II**. The transition state for this step implies the elongation of the $C_{benzylic}$-S bond (from 1.86 Å to 1.90 Å) $C_{methylenic}$-H bond (from 1.10 Å to 1.45 Å) and a shortening in $C_{methylenic}$-$C_{benzylic}$ bond (from 1.53 to 1.49 Å) (see supplementary figures below and supplementary table 3). An enolate intermediate was not found during the exploration of the potential energy surface, thus we can say that the reaction follows an E2 mechanism pathway. The catalytic cycle is closed by protonation of a bicarbonate forming carbon dioxide and water.

Supplementary Figure 2: proposal for the mechanism of the reaction in the anion-binding process.

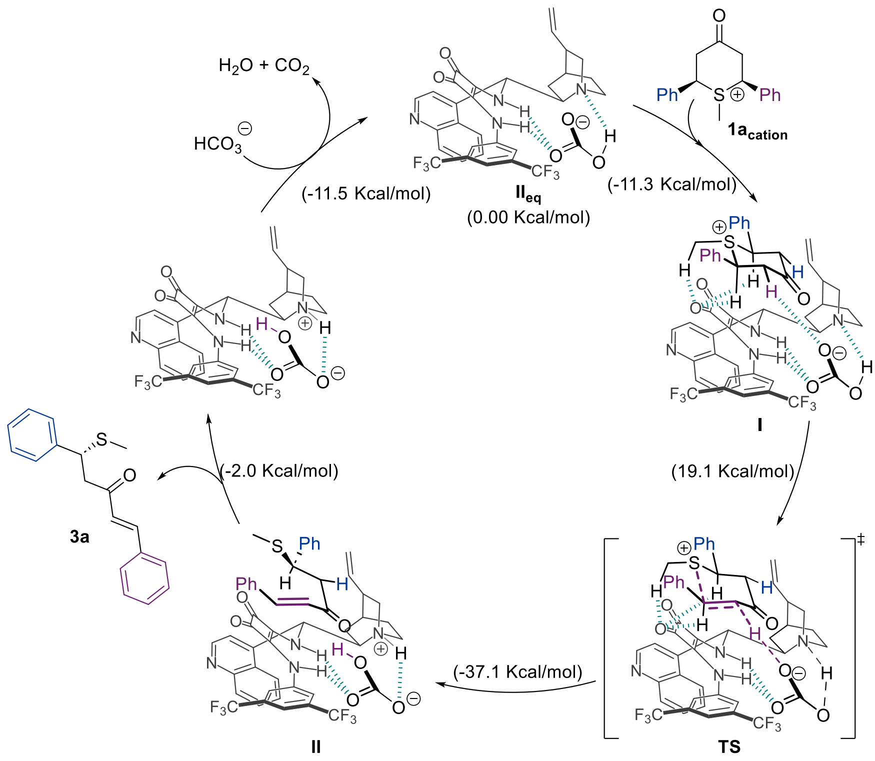

Regarding the origin the enantioselectivity, we decided to study the chemical course for the other enantiomer. In order to determine the starting point for the other enantiomer we optimized 2 different starting structures that gave 2 different energy minimums (**I', I''** see the computational data for a picture of the structures). It turned out to be that the most stable structure was the one that consisted in another conformer of the catalyst (**I'**). The energy profile for the minor enantiomer followed similar energy barriers (see energy profile down below). However, there are clear structural differences between both transition states (see Supplementary Table 3 and Supplementary Table 4). The main difference is the higher elongation of the $C_{benzylic}$-S bond (1.899 in **TS** vs 2.337 in **TS'**).

Supplementary Table 3: distances in **I** and in **TS**

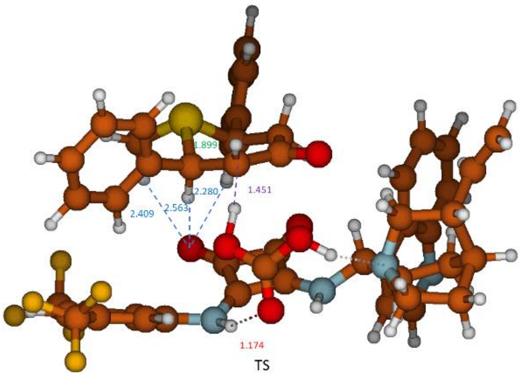

TS

Entry	Bond	Distance in I (Å)	Distance in TS (Å)
**1**	$C_{benzylic}$-S bond	1.855	1.899
**2**	$C_{methylenic}$-$C_{benzylic}$	1.533	1.495

**3**	$C_{methylenic}$-H	1.093	1.451
**4**	H-$O_{bicarbonate}$	2.418	1.174

Supplementary Table 4: distances in **I'** and in **TS'**

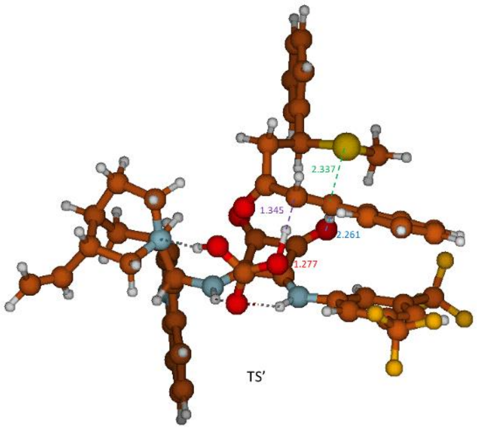

TS'

Entry	Bond	Distance in I' (Å)	Distance in TS' (Å)
**1**	$C_{benzylic}$-S bond	1.861	2.337
**2**	$C_{methylenic}$-$C_{benzylic}$	1.529	1.433
**3**	$C_{methylenic}$-H	1.098	1.345
**4**	H-$O_{bicarbonate}$	2.326	1.277

The high elongation of the $C_{benzylic}$-S bond could lead to the formation of a carbocation in the $C_{benzylic}$ which is slightly stabilized by resonance by the aromatic ring. Aiming to prove this hypothesis, we performed an NBO analysis of both TS in order to analyze the change in the natural charge at the S atom and $C_{benzylic}$ (see comparison in Supplementary Table 5)

Supplementary Table 5: Natural charges obtained by NBO analysis

Entry	Bond	Natural charge in TS	Natural charge in TS'
**1**	$C_{benzylic}$	-0.343	-0.083
**2**	S	0.844	0.500

As it can be seen, there is a significant change in the charge distribution between both transition states. While in **TS** almost all the positive charge is at the sulfur atom, in **TS'** the $C_{benzylic}$ has lost an important part of its electron density (~0.3 electrons). In the view of these results, we believe that contrary to the major enantiomer where the mechanism was very similar to a E2, for the minor enantiomer we believe that the mechanism resembles more of a E1 mechanism. However, we could not find the minimum corresponding to the carbocation, and therefore we assume that the mechanism is not a pure E1 mechanism.

The relative energies of all the structures found are illustrate in Supplementary Figure 3.

Supplementary Figure 3: DFT M06-2X/6-31+G(d,p)//M06-2X/6-31G(d,p) (PCM=chloroform) Reaction Energy profile for both enantiomer (major in purple, minor in blue).

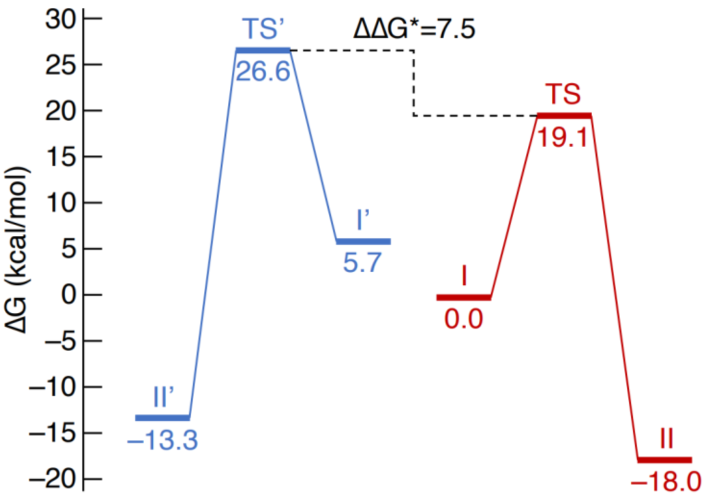

11.2. Study of the equilibrium between sulfonium salt and HCO_3^-

Since bicarbonate has an important role in the enantioselectivity of the process (see the manuscript), we decided to estimate the tendency of the catalyst to preferably bind the bicarbonate anion instead of the sulfonium salt, so that the mechanism of a direct deprotonation could be discarded. Therefore, we determined the energy of each of the following structures at the M06-2X/6-31+G(d,p)//M06-2X/6-31G(d,p) level. Then, we obtained ΔG by the difference between the sum of the free energies of reagents and the sum of the free energies of the products (see Supplementary Figure 4).

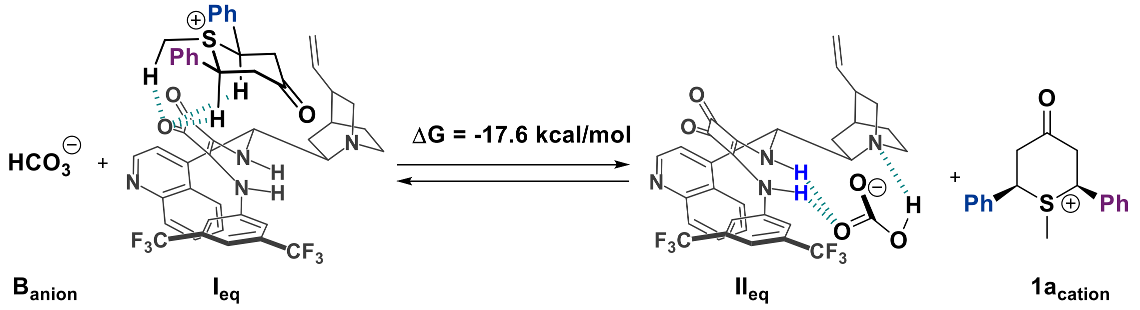

Supplementary Figure 4: study of the affinity of bicarbonate and substrate to bind the catalyst.

The energy difference is not negligible, 17.60 kcal·mol^{-1}. Therefore, there is a clear preference for the catalyst to bind the sulfonium what is supported by the anion-binding process observed by titration experiments (see section 8).

11.3. Computational study of non-covalent interactions and energy decomposition análisis

We have carried out an exhaustive analysis to characterize bonding properties in relevant structures. To this, we have computed the non-covalent interactions (NCI) using the NCIPLOT method, an energy decomposition analysis (EDA) using the EDA-NCI method, and further bonding properties were characterized with the Quantum Theory of Atoms in Molecules (QTAIM).

The bonding characterization analyses were done for the most stable structure found with CREST (see Figure 7 (c) in the main article), as well as for structures I, TS and TS' (see Figure 6 in the main article). The results are given and discussed below for each structure separately.

For the most stable structure found with CREST the EDA-NCI analysis was performed evaluating the interaction between the reactant sulfoninum cation, and the catalyst bonded to the bicarbonate anion. The main interaction energy components are:

Electrostatic = -78.2 kcal/mol

Pauli = 43.2 kcal/mol

Polarization = 2.1 kcal/mol

thus, giving a total interaction of = -32.9 kcal/mol, clearly dominated by the electrostatic term. The total deformation density plot is shown in Supplementary Figure 3. We observe that the main charge transfer is localized in the sulfonium – catalyst region.

After quantification of the interaction energy terms, a qualitative analysis of the non-covalent interactions was performed using the QTAIM and NCI-plot methods. Supplementary Figure 4 shows the QTAIM analysis with a very complex net of bonding interactions. Notice that strong bonds are shown between the bicarbonate and the catalyst. In Supplementary Figure 5 the analysis with the NCI-plot method shows that not only electrostatic interactions and hydrogen bonds stabilizes the complex, but also other NCI interactions.

The EDA-NCI analysis for structure I gives the following interaction energy components:

Electrostatic = -90.9 kcal/mol

Pauli = 49.6 kcal/mol

Polarization = 2.5 kcal/mol

With a total interaction energy of -38.8 kcal/mol and dominated by the electrostatic term. The corresponding total deformation density plot is shown in Supplementary Figure 6. Similarly to the analysis in the most stable structure found with CREST, we observe for **I** that the main charge transfer is localized in the sulfonium – catalyst region.

We have also analyzed the interactions in the complex formed in structure **I**, using the QTAIM and NCI-plot methods. The results are shown in Supplementary Figures 7 and 8. We also observe a complex net of bond stabilizing the structure with the QTAIM, with strong interactions between the three moieties – catalyst, bicarbonate and sulfonium. The NCI plot also shows further non-covalent interactions stabilizing the complex.

In the case of TS, we have performed the QTAIM and the NCI analyses (see Supplementary Figures 9 and 10, respectively). We have also performed a QTAIM analysis on the TS' structure (see Supplementary Figure 11).

For structures **I**, **TS** and **TS'** we have analyzed the density in some relevant bond critical points (BCP) (see Supplementary Table 6) and the charge on some relevant atoms (see Supplementary Table 7). In the reaction that occurs through **TS**, the density in the BCP S-C is very similar to that of structure **I**. The same holds for the atomic charges in the atoms forming such bond (C and S). This reflects that in the TS the S-C bond is not broken yet. The charges in the C-H bond corresponding to the proton being transferred show differences in I and in TS, indicating that the bicarbonate is taking the proton. This is precisely the reaction that determine the energy barrier, the H transfer.

However, if we now focus on structure TS', we can see that the density in the S-C bond, and the atomic charges are very different from TS. In the path occurring through TS', the C-S bond is almost broken while the proton is transferred simultaneously. In this case, the proton transfer is not as advanced as in TS. Comparison between the geometry of TS and TS' is given in Supplementary Figure 12.

Therefore, we can conclude that in TS, proton abstraction by bicarbonate is more favorable than C-S cleavage; although once the proton has been transferred, the breaking of the C-S bond is more prone than initially.

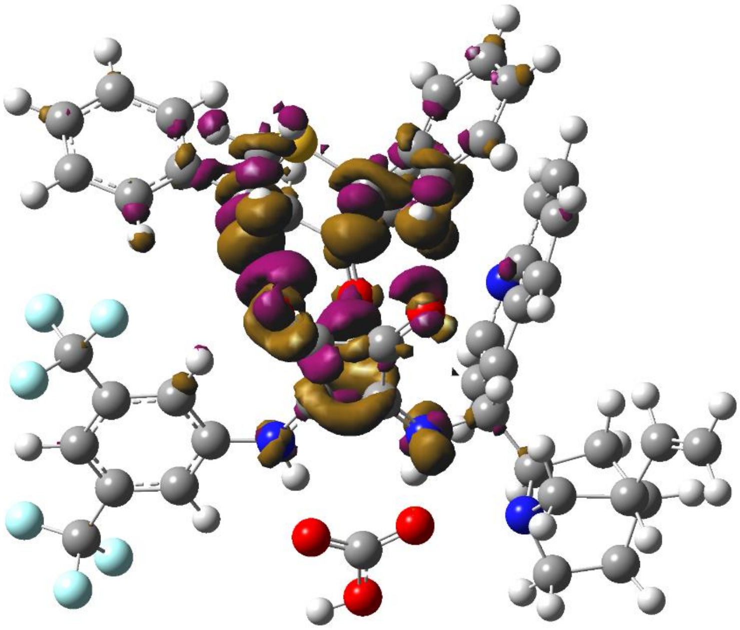

Supplementary Figure 5: Total deformation density plot in the most stable structure found with CREST. The isosurface value is 0.001 au.

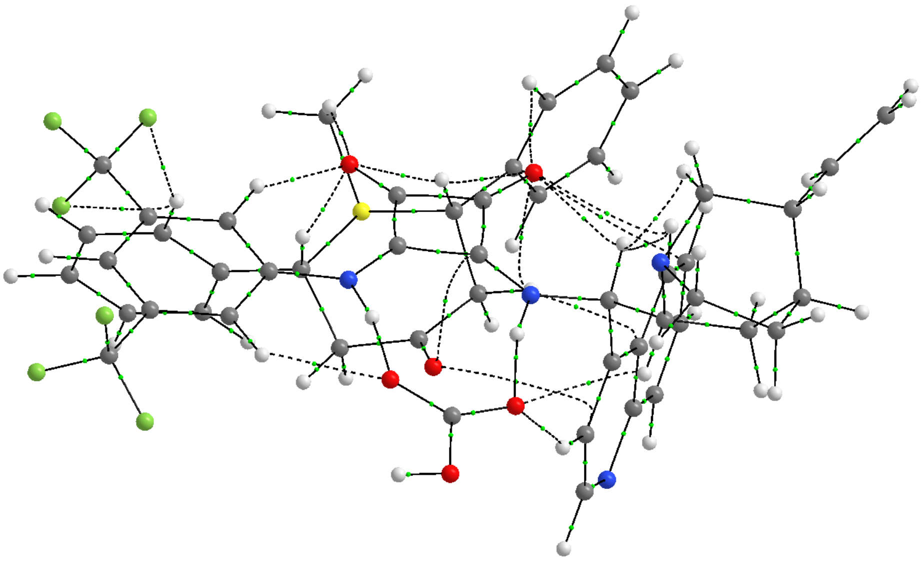

Supplementary Figure 6: QTAIM analysis in the most stable structure found with CREST. Bond critical points are represented with small green point.

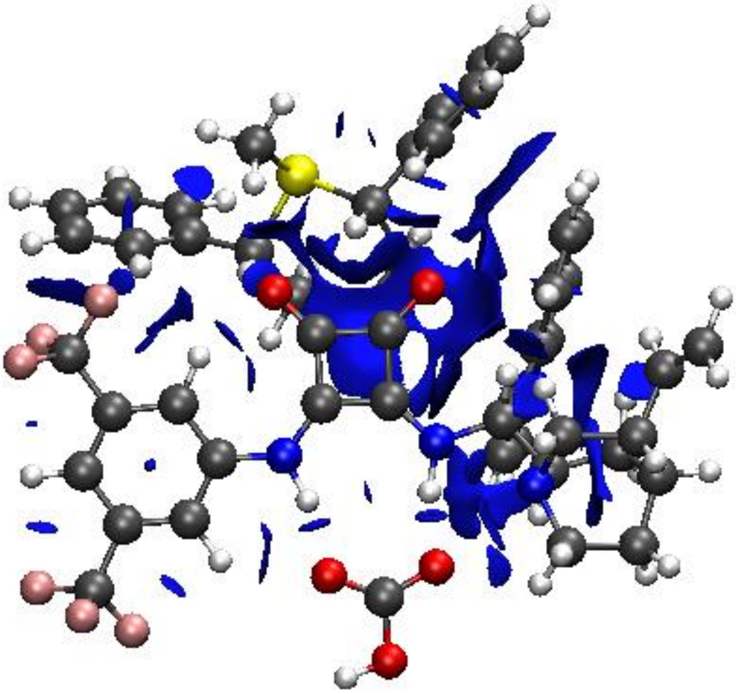

Supplementary Figure 7: NCI-plot analysis in the most stable structure found with CREST.

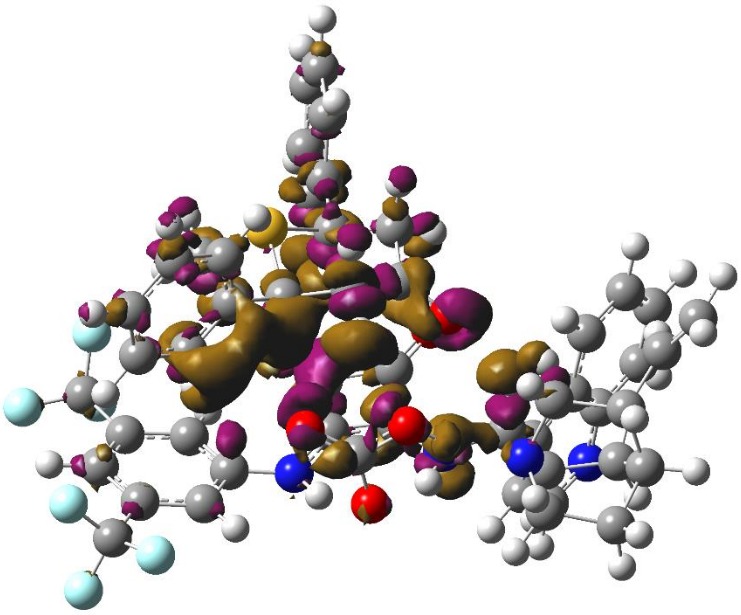

Supplementary Figure 8: Total deformation density plot for structure **I**. The isosurface value is 0.001 au.

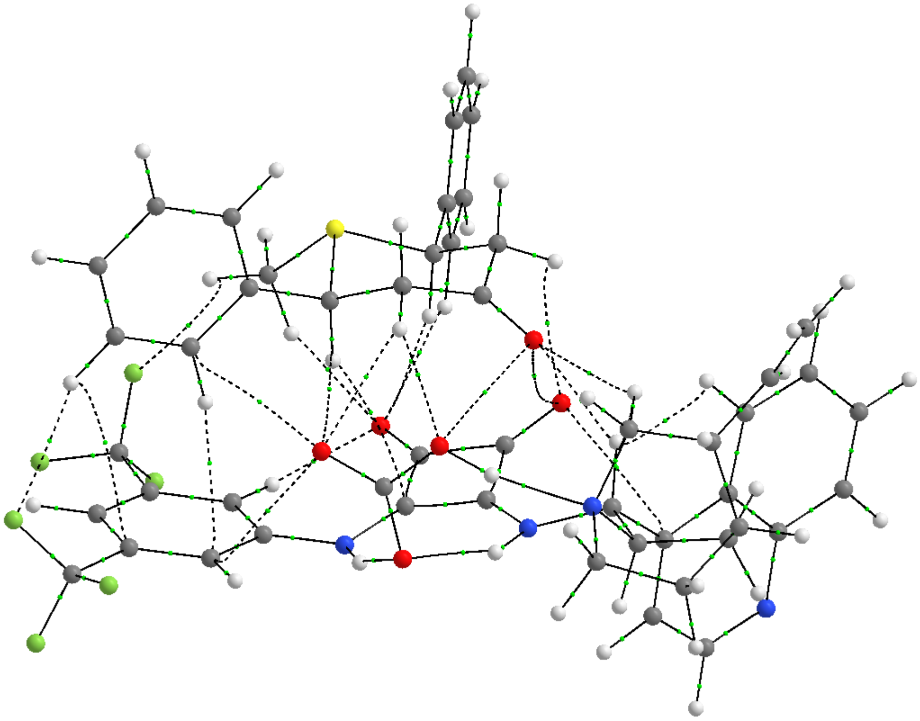

Supplementary Figure 9: QTAIM analysis in structure **I**. Bond critical points are represented with small green point.

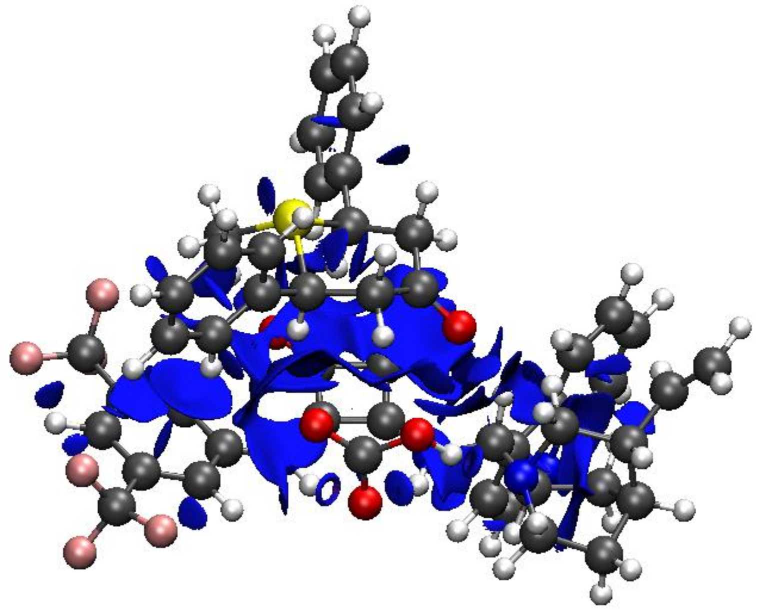

Supplementary Figure 10: NCI-plot analysis in structure **I**.

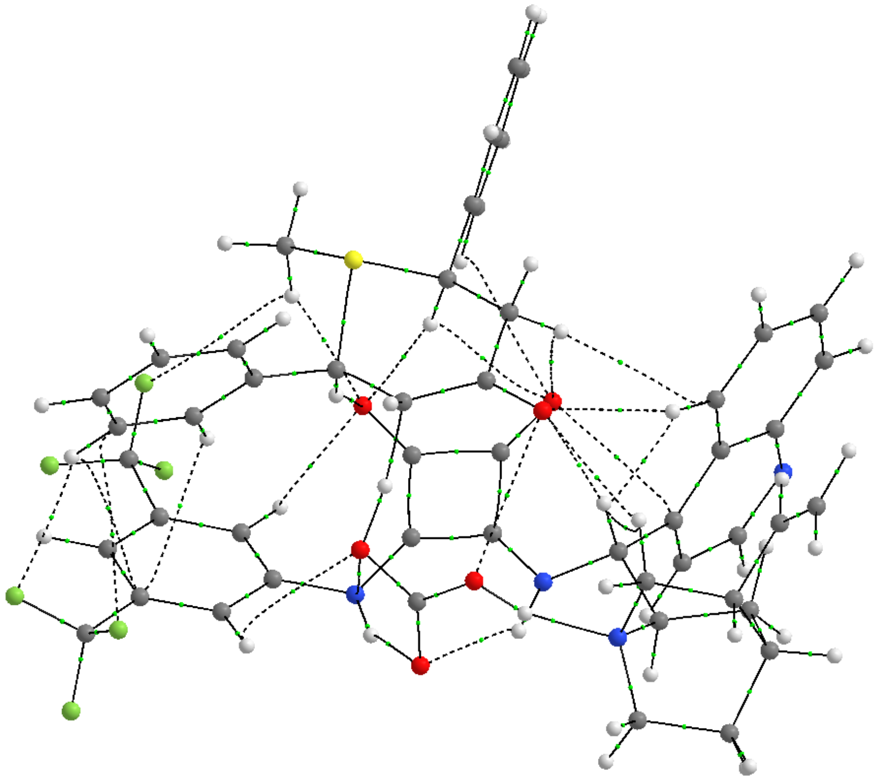

Supplementary Figure 11: QTAIM analysis in structure **TS**. Bond critical points are represented with small green point.

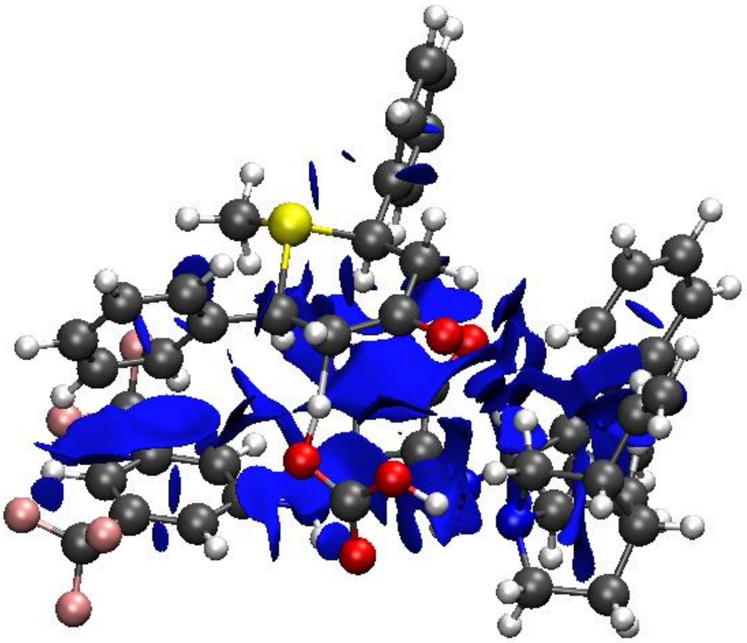

Supplementary Figure 12: NCI-plot analysis in structure **TS**.

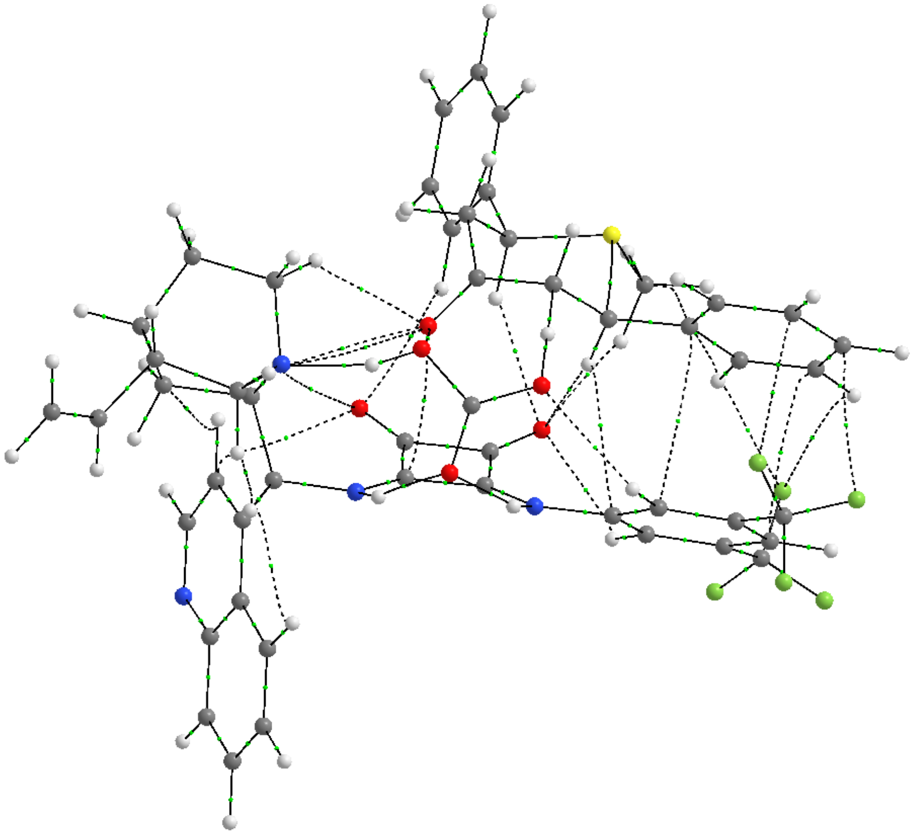

Supplementary Figure 13: QTAIM analysis in structure **TS'**. Bond critical points are represented with small green point.

Supplementary Table 6: Density (ρ) in the Bond Critical Points (BCP) found in the QTAIM analyses for relevant structures **I**, **TS** and **TS'**. H-C corresponds to the bond of the H being transferred.

	**TS**	**TS'**	**I**
$\rho_{S\text{-}C(benzylic)}$	0.1528	0.0626	0.1694
$\rho_{H\text{-}C(methylenic)}$	0.1122	0.1456	0.2823
$\rho_{O\text{-}H}$	0.1895	0.1418	0.0121

Supplementary Table 7: Atomic charges (q) computed in the QTAIM analyses for relevant structures **I**, **TS** and **TS'**. C(-H) is the carbon atom holding the H being transferred.

	**TS**	**TS'**	**I**
q_S	0.25	0.08	0.26
$q_{C(benzylic)}$	-0.02	0.02	-0.03
$q_{C(methylenic)}$	-0.23	-0.18	0.02
q_O	-1.27	-1.27	-1.34
q_H	0.53	0.47	0.13

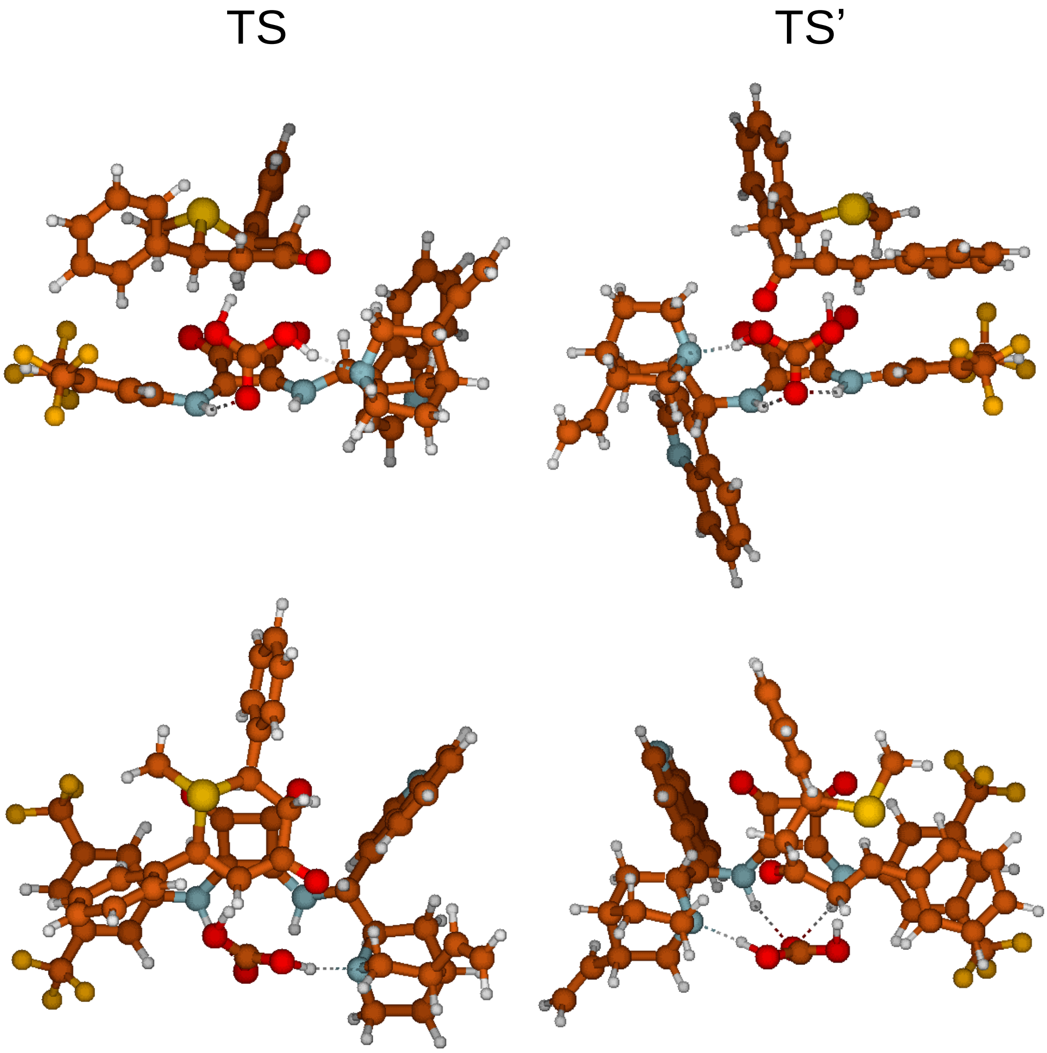

Supplementary Figure 14: Top and lateral views of TS and TS’.

12. X-Ray crystal structure analysis of 3a

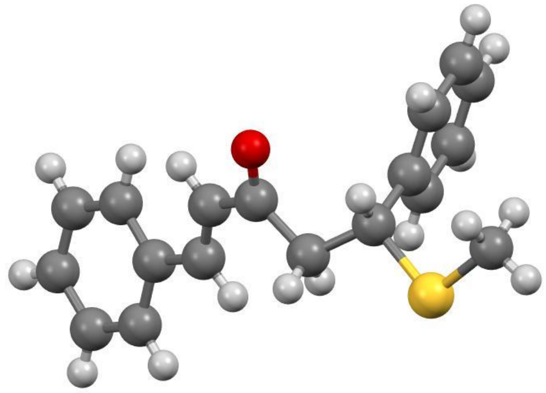

CCDC Nr.: 2160424

A clear colourless needle-like specimen of $C_{18}H_{18}OS$, approximate dimensions 0.020 mm x 0.020 mm x 0.189 mm, was used for the X-ray crystallographic analysis. The X-ray intensity data were measured.

The total exposure time was 16.60 hours. The frames were integrated with the Bruker SAINT software package using a narrow-frame algorithm. The integration of the data using a monoclinic unit cell yielded a total of 12138 reflections to a maximum θ angle of 25.35° (0.83 Å resolution), of which 2747 were independent (average redundancy 4.419, completeness = 99.7%, R_{int} = 5.45%, R_{sig} = 5.48%) and 2155 (78.45%) were greater than $2\sigma(F^2)$. The final cell constants of a = 11.4072(12) Å, b = 5.5576(7) Å, c = 11.9984(12) Å, β = 100.282(5)°, volume = 748.44(14) $Å^3$, are based upon the refinement of the XYZ-centroids of 2362 reflections above 20 σ(I) with 7.203° < 2θ < 44.35°. Data were corrected for absorption effects using the multi-scan method (SADABS). The ratio of minimum to maximum apparent transmission was 0.867.

The final anisotropic full-matrix least-squares refinement on F^2 with 182 variables converged at R1 = 3.68%, for the observed data and wR2 = 7.33% for all data. The goodness-of-fit was 1.009. The largest peak in the final difference electron density synthesis was 0.166 $e^-/Å^3$ and the largest hole was -0.169 $e^-/Å^3$ with an RMS deviation of 0.035 $e^-/Å^3$. On the basis of the final model, the calculated density was 1.253 g/cm^3 and F(000), 300 e^-.

**Chemical formula**	$C_{18}H_{18}OS$
**Formula weight**	282.38 g/mol
**Temperature**	200(2) K
**Wavelength**	0.71073 Å
**Crystal size**	0.020 x 0.020 x 0.189 mm
**Crystal habit**	clear colourless needle
**Crystal system**	monoclinic
**Space group**	P 1 21 1
**Unit cell dimensions**	a = 11.4072(12) Å α = 90°
	b = 5.5576(7) Å β = 100.282(5)°
	c = 11.9984(12) Å γ = 90°
**Volume**	748.44(14) $Å^3$
**Z**	2
**Density (calculated)**	1.253 g/cm^3
**Absorption coefficient**	0.209 mm^{-1}
**F(000)**	300

13. Spectra Data.

13.1. (1E,4E)-1,5-bis(4-bromophenyl)penta-1,4-dien-3-one: NMR

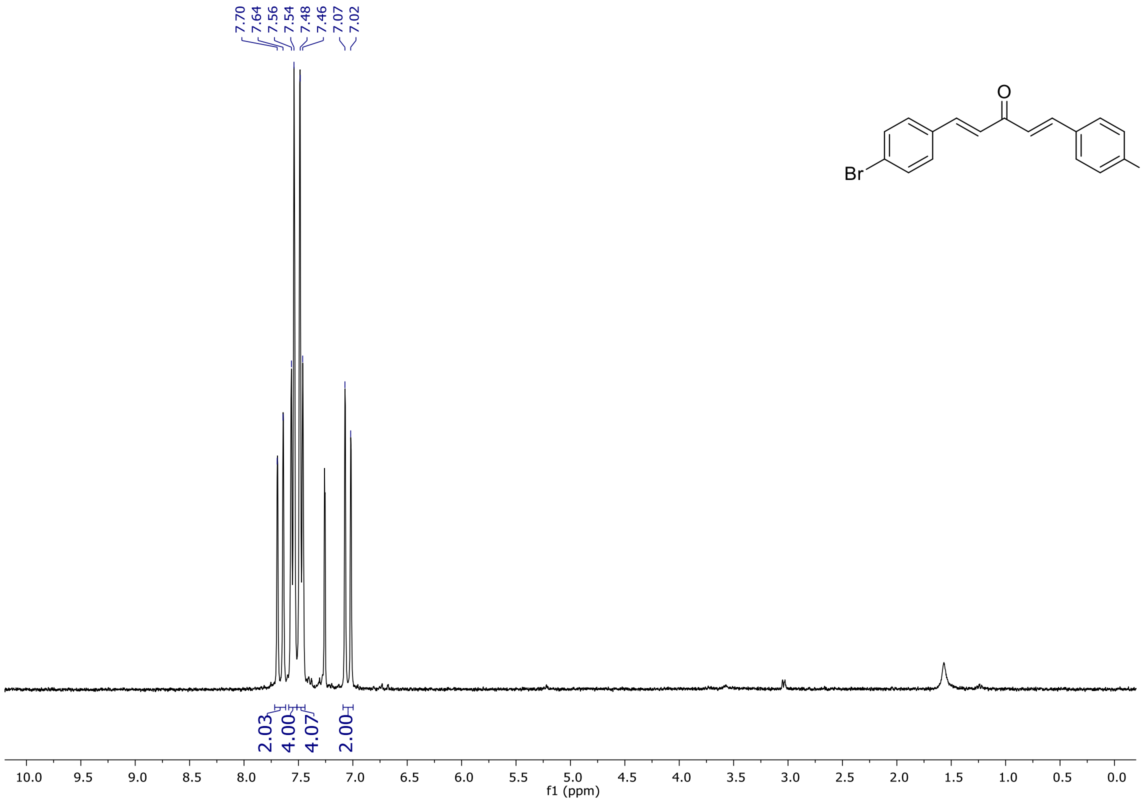

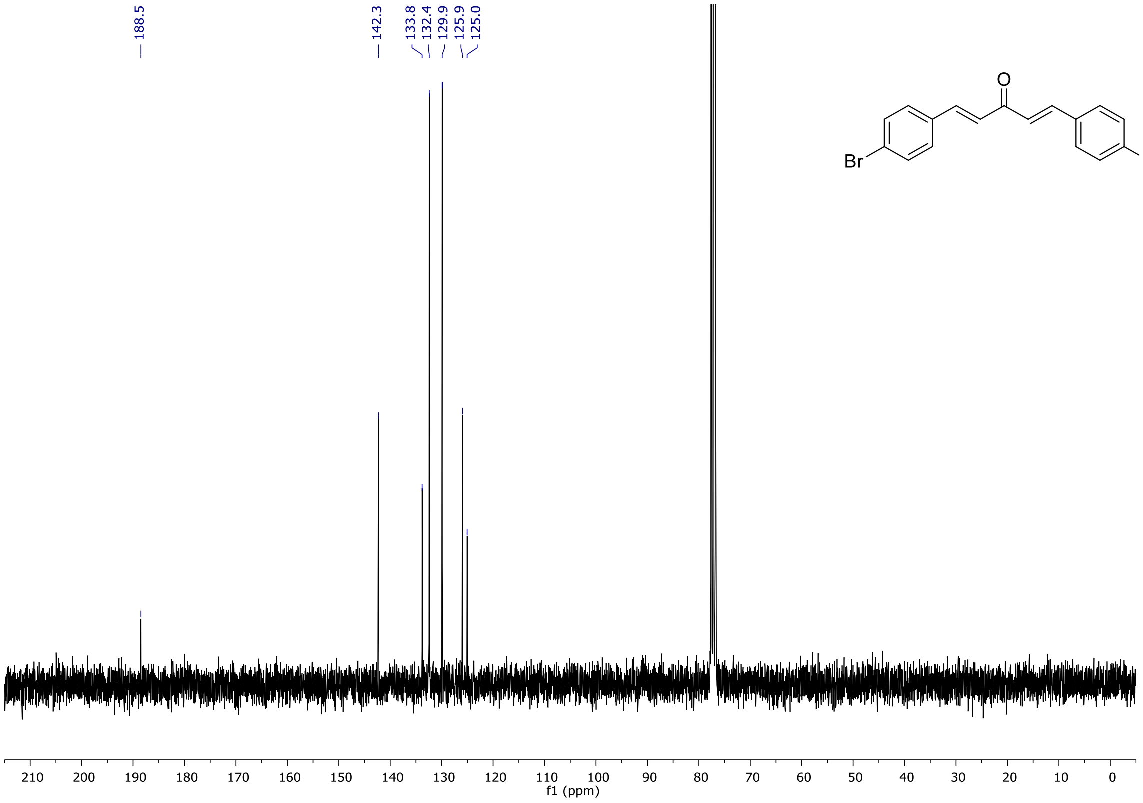

13.2. Sulfonium salts: NMR

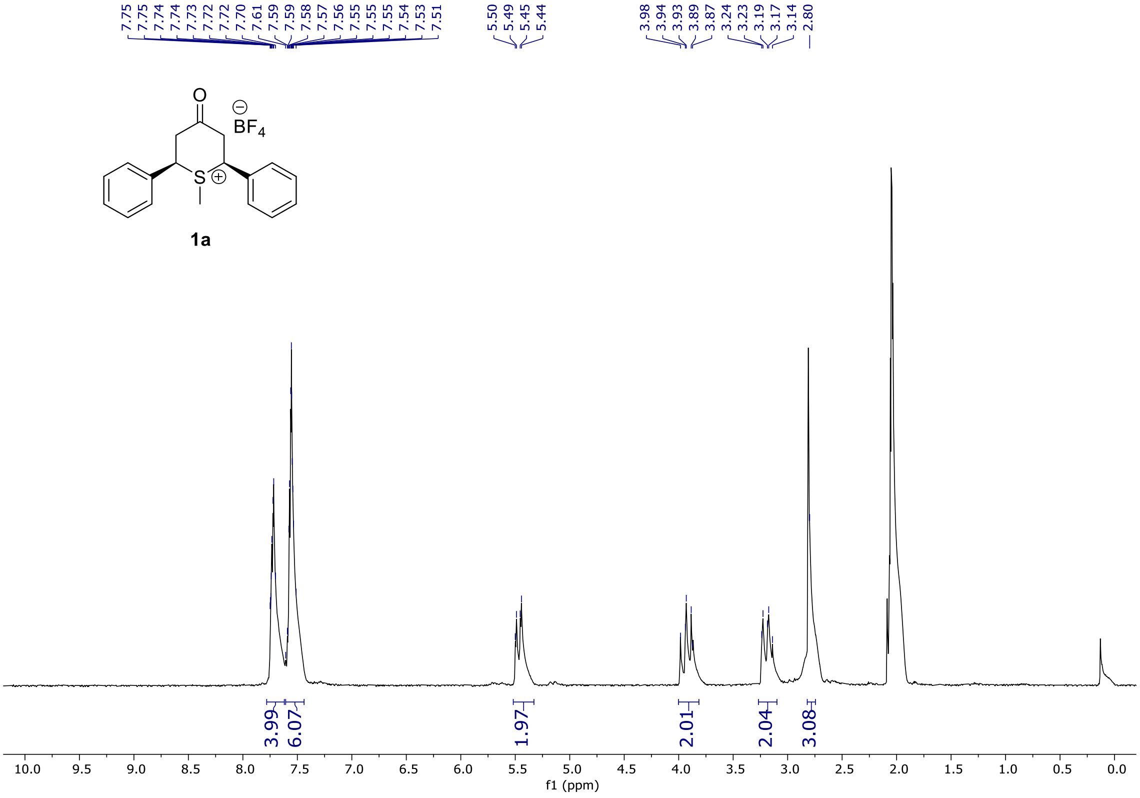

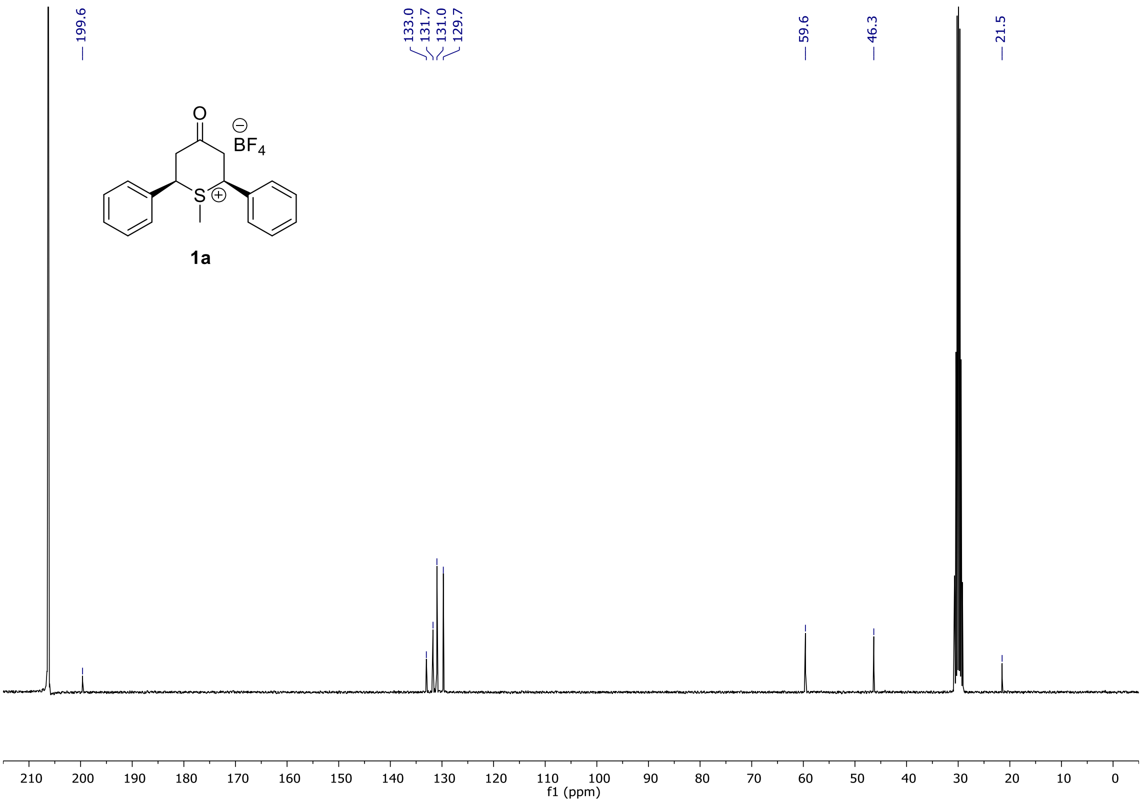

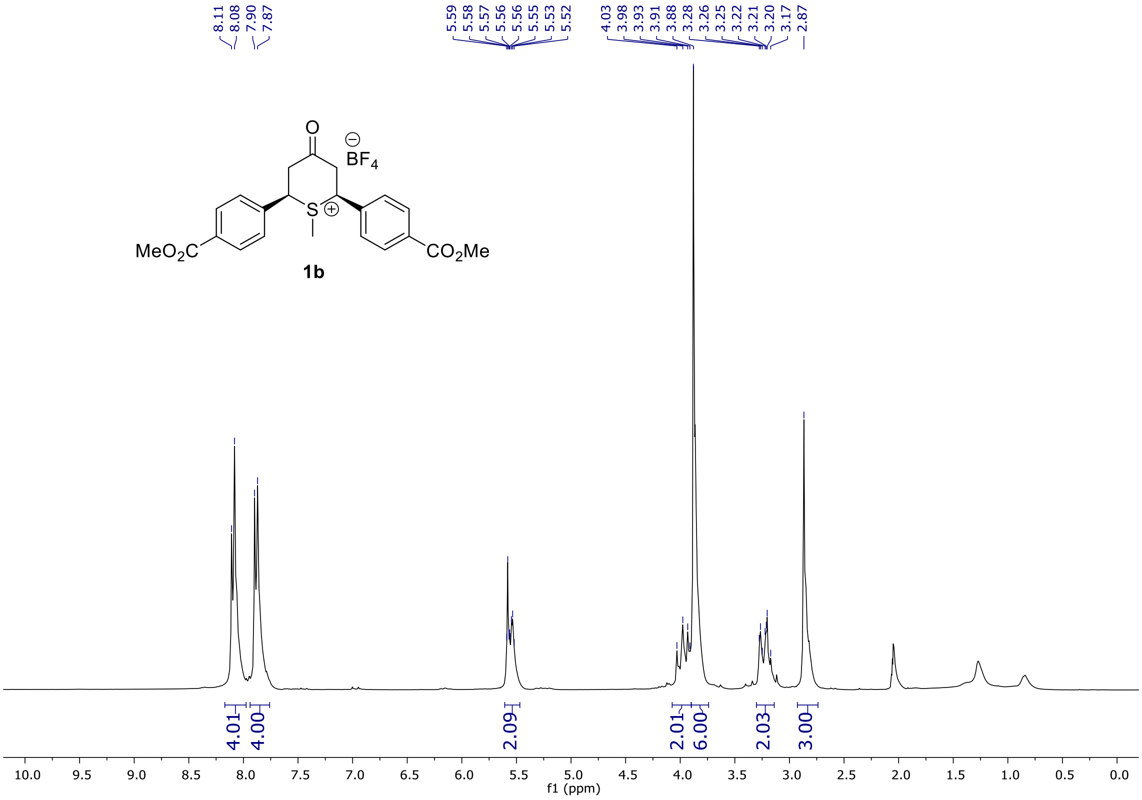
8.11
8.08
7.90
7.87
5.59
5.58
5.57
5.56
5.56
5.55
5.53
5.52
4.03
3.98
3.93
3.91
3.88
3.28
3.26
3.25
3.22
3.21
3.20
3.17
2.87
BF4
S
MeO2C
CO2Me
1b
4.01
4.00
2.09
2.01
6.00
2.03
3.00
f1 (ppm)

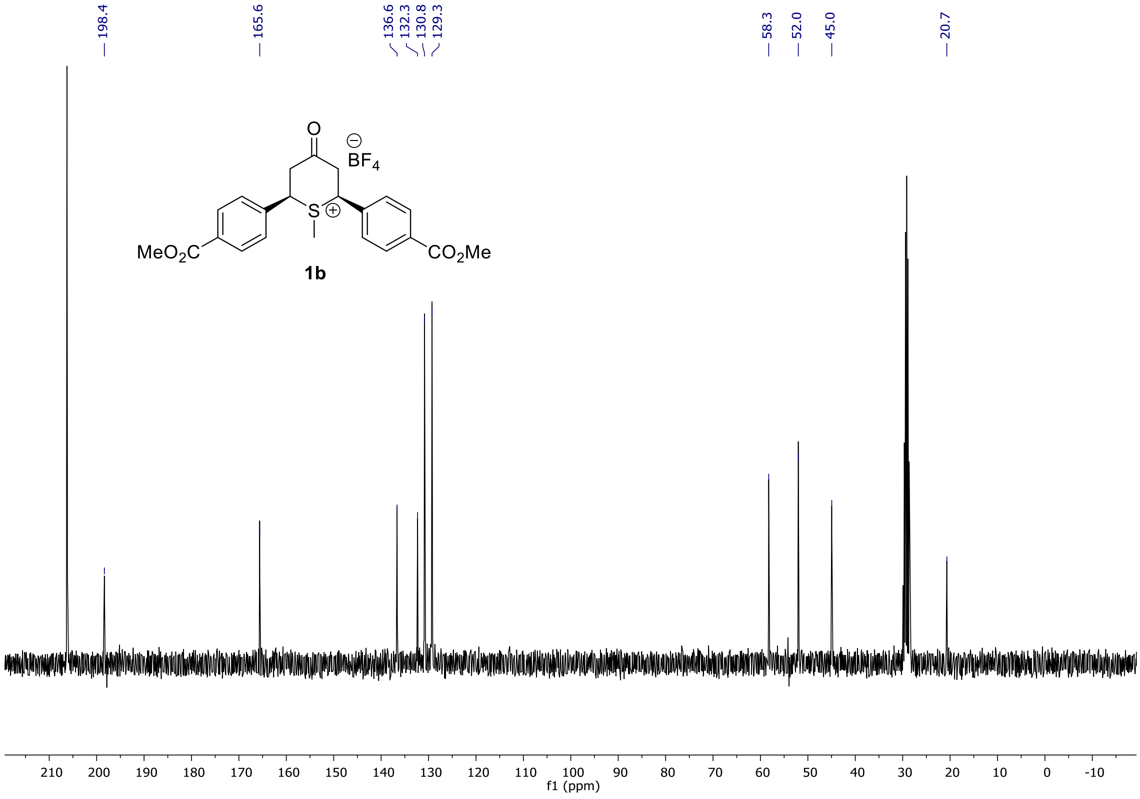
198.4
165.6
136.6
132.3
130.8
129.3
58.3
52.0
45.0
20.7
BF4
S
MeO2C
CO2Me
1b
f1 (ppm)

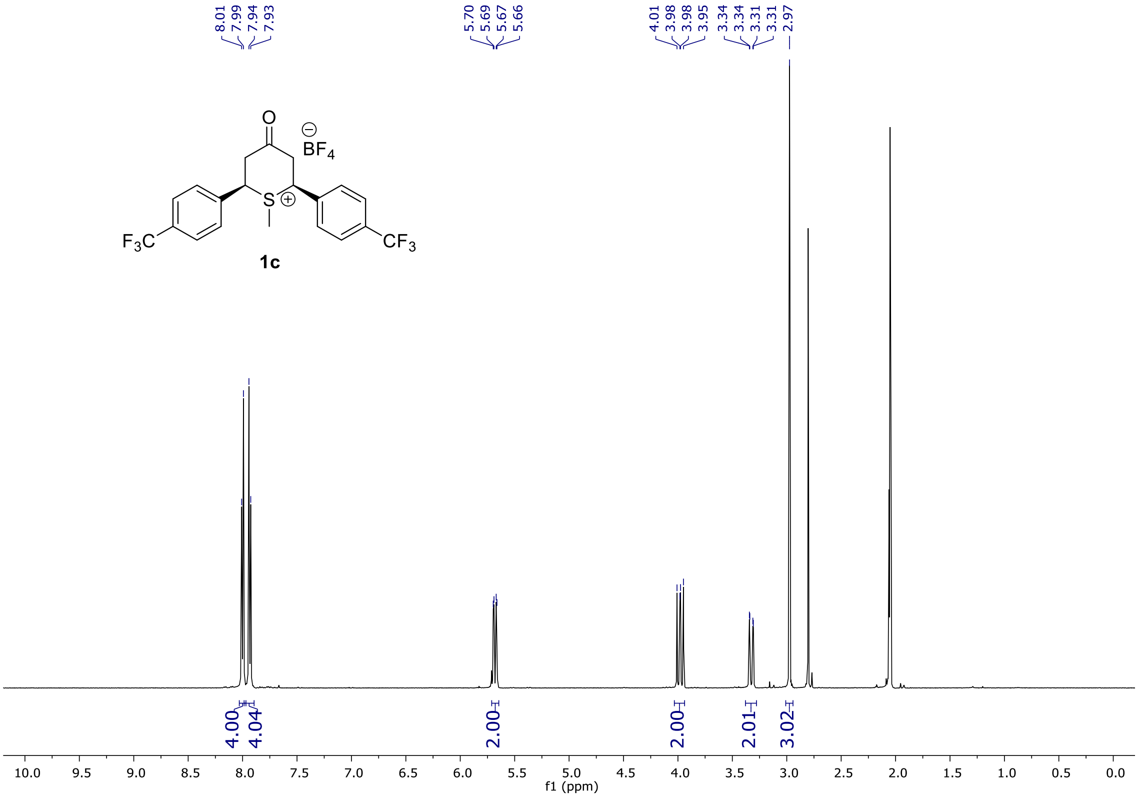
8.01
7.99
7.94
7.93
5.70
5.69
5.67
5.66
4.01
3.98
3.98
3.95
3.34
3.34
3.31
3.31
2.97
O
BF4
S
F3C
CF3
1c
4.00
4.04
2.00
2.00
2.01
3.02
10.0 9.5 9.0 8.5 8.0 7.5 7.0 6.5 6.0 5.5 5.0 4.5 4.0 3.5 3.0 2.5 2.0 1.5 1.0 0.5 0.0
f1 (ppm)

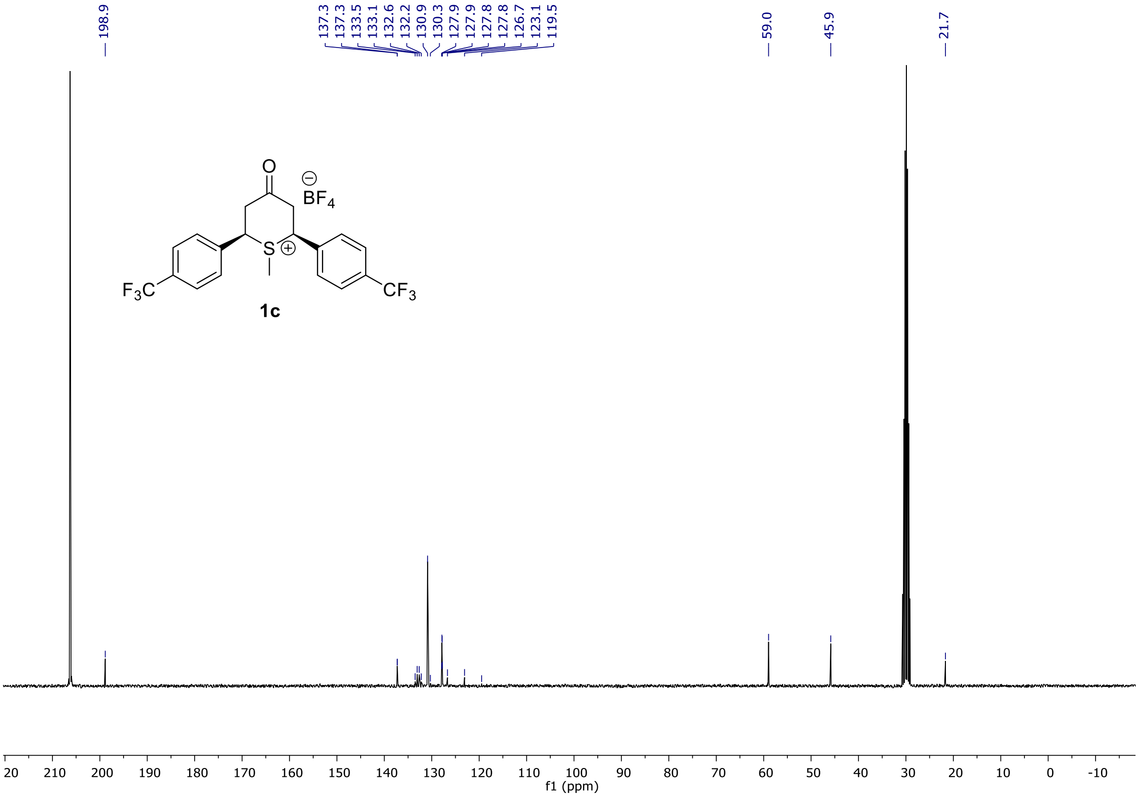
198.9
137.3
137.3
133.5
133.1
132.6
132.2
130.9
130.3
127.9
127.9
127.8
127.8
126.7
123.1
119.5
59.0
45.9
21.7
O
BF4
S
F3C
CF3
1c
20 210 200 190 180 170 160 150 140 130 120 110 100 90 80 70 60 50 40 30 20 10 0 -10
f1 (ppm)

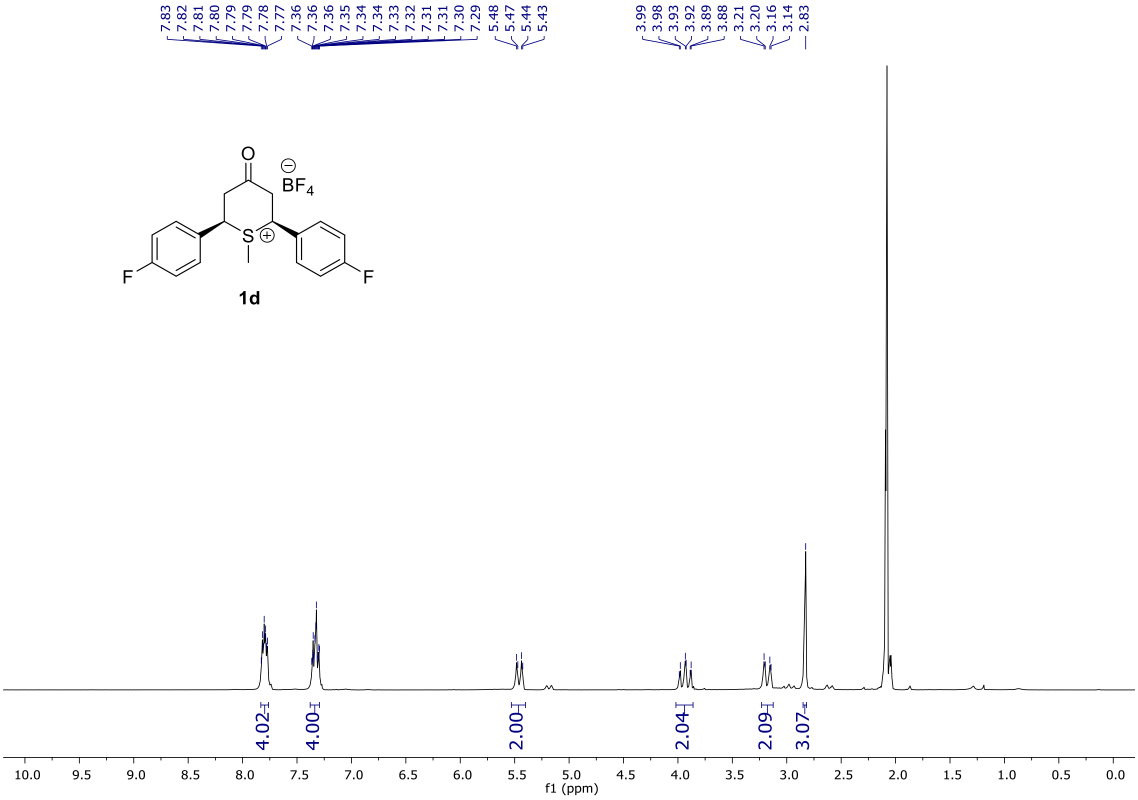
7.83 7.82 7.81 7.80 7.79 7.79 7.78 7.77 7.36 7.36 7.35 7.34 7.34 7.33 7.32 7.31 7.31 7.30 7.29 5.48 5.47 5.44 5.43
3.99 3.98 3.93 3.92 3.89 3.88 3.21 3.20 3.16 3.14 2.83
O
BF4
S
F
F
1d
4.02
4.00
2.00
2.04
2.09
3.07
f1 (ppm)

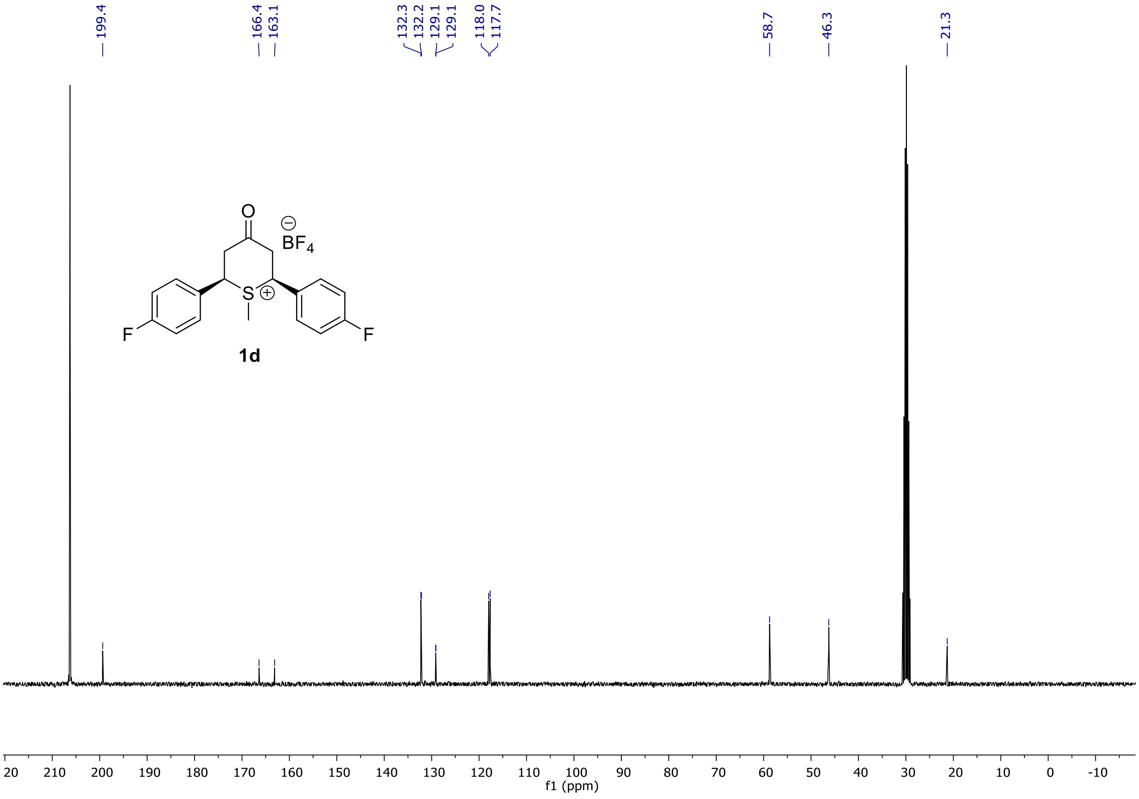
199.4
166.4 163.1
132.3 132.2 129.1 129.1 118.0 117.7
58.7
46.3
21.3
O
BF4
S
F
F
1d
f1 (ppm)

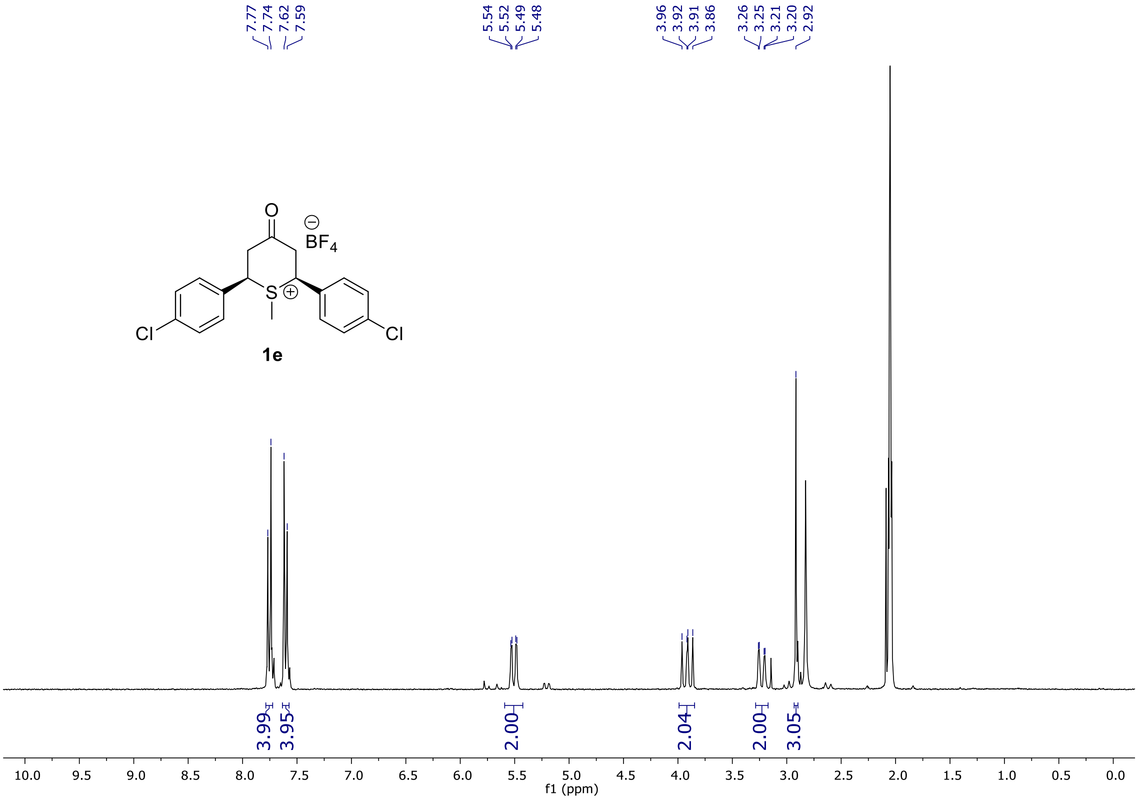
7.77
7.74
7.62
7.59
5.54
5.52
5.49
5.48
3.96
3.92
3.91
3.86
3.26
3.25
3.21
3.20
2.92
O
BF4
S
Cl
Cl
1e
3.99
3.95
2.00
2.04
2.00
3.05
10.0 9.5 9.0 8.5 8.0 7.5 7.0 6.5 6.0 5.5 5.0 4.5 4.0 3.5 3.0 2.5 2.0 1.5 1.0 0.5 0.0
f1 (ppm)

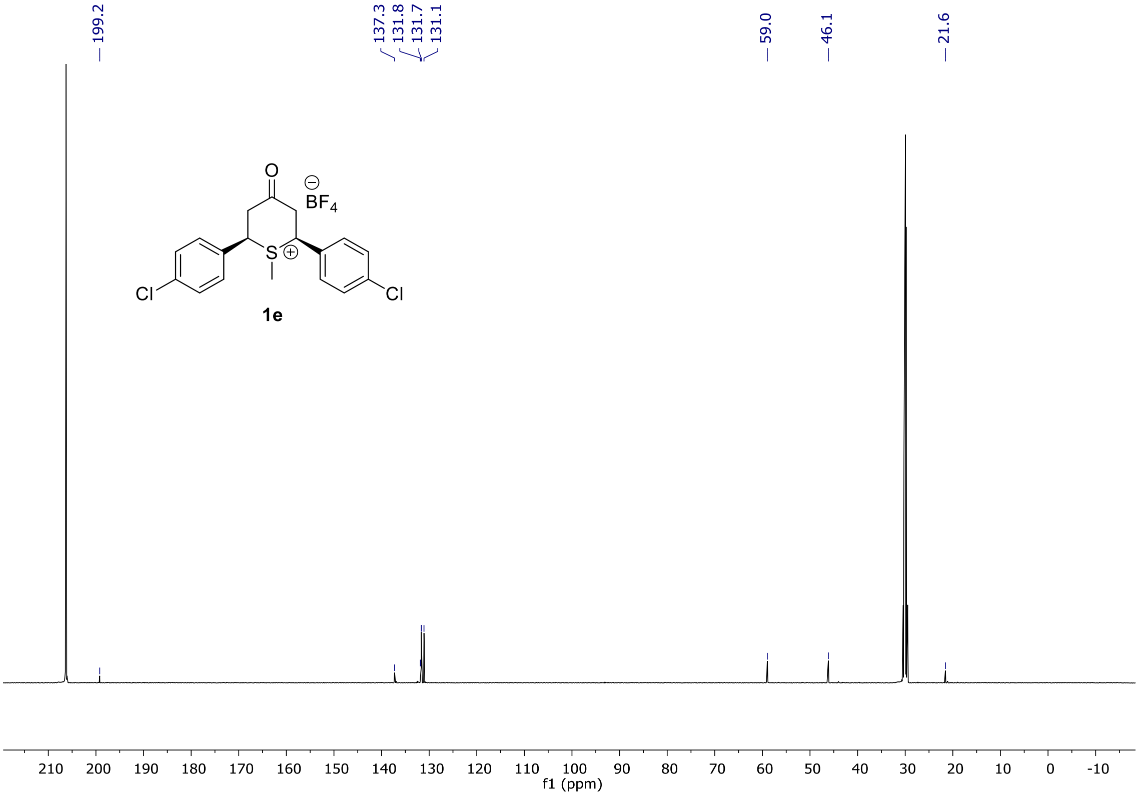
199.2
137.3
131.8
131.7
131.1
59.0
46.1
21.6
O
BF4
S
Cl
Cl
1e
210 200 190 180 170 160 150 140 130 120 110 100 90 80 70 60 50 40 30 20 10 0 -10
f1 (ppm)

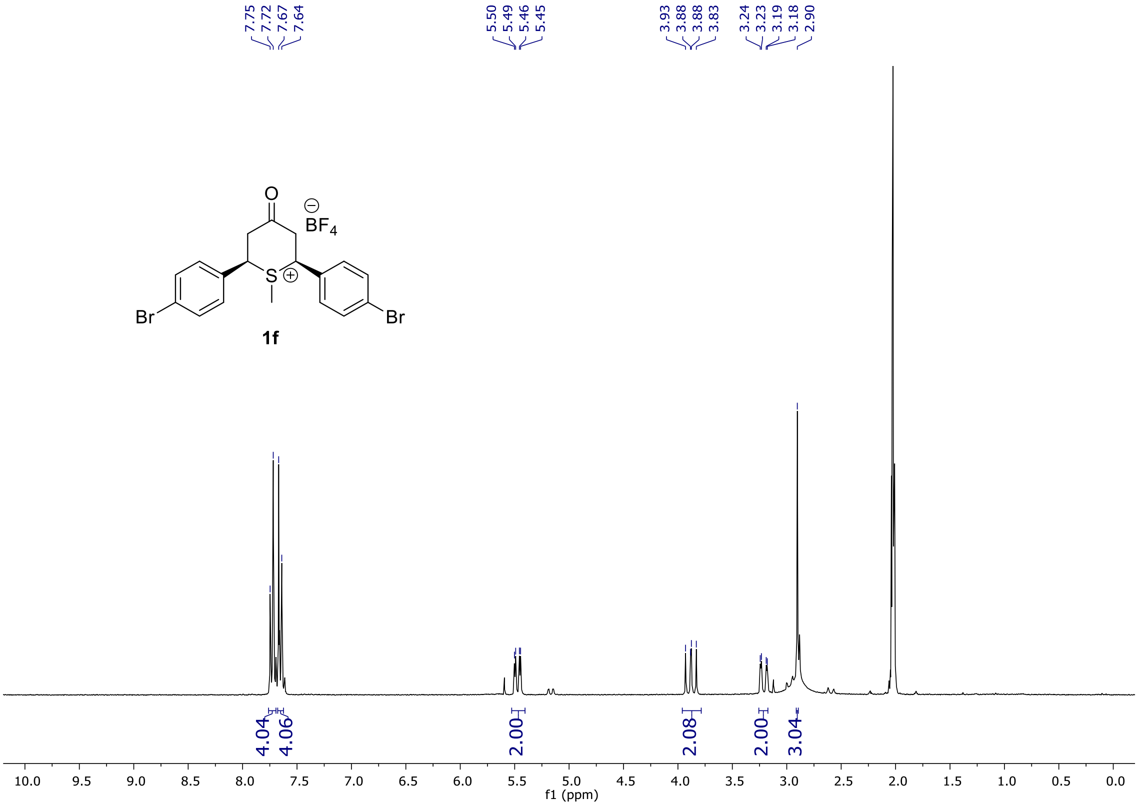
7.75
7.72
7.67
7.64
5.50
5.49
5.46
5.45
3.93
3.88
3.88
3.83
3.24
3.23
3.19
3.18
2.90
1f
4.04
4.06
2.00
2.08
2.00
3.04
f1 (ppm)

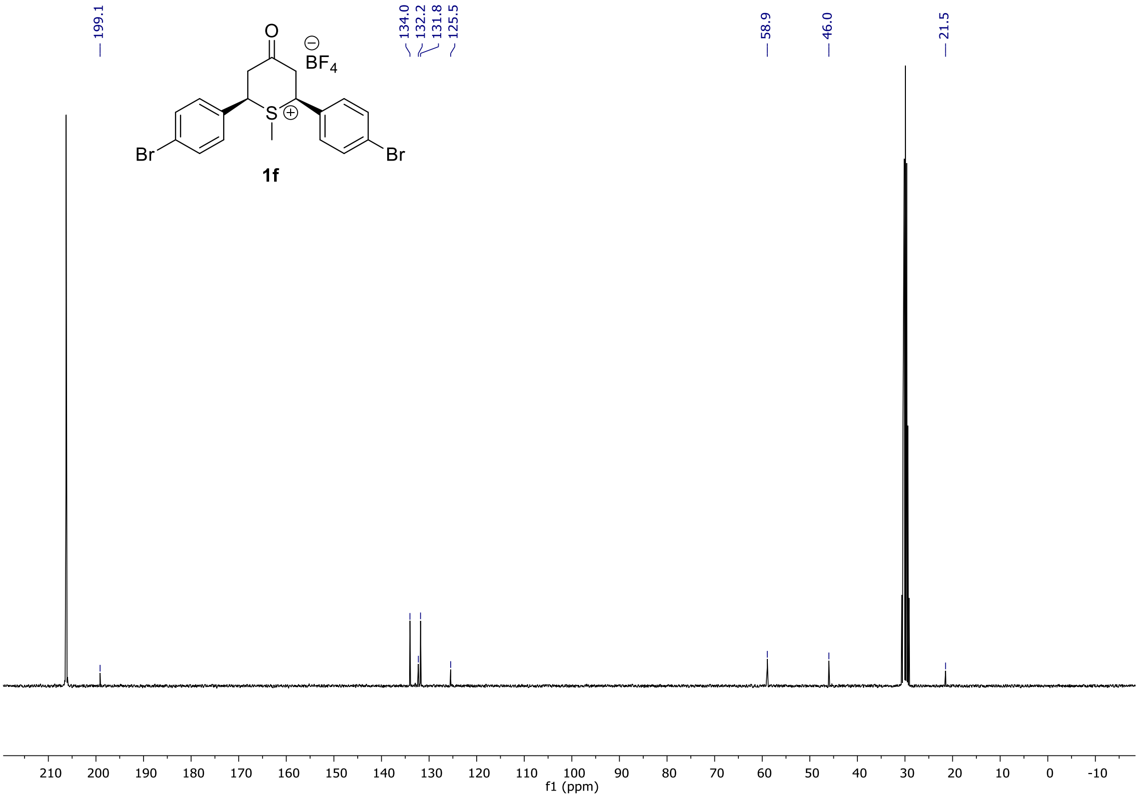
199.1
134.0
132.2
131.8
125.5
58.9
46.0
21.5
1f
f1 (ppm)

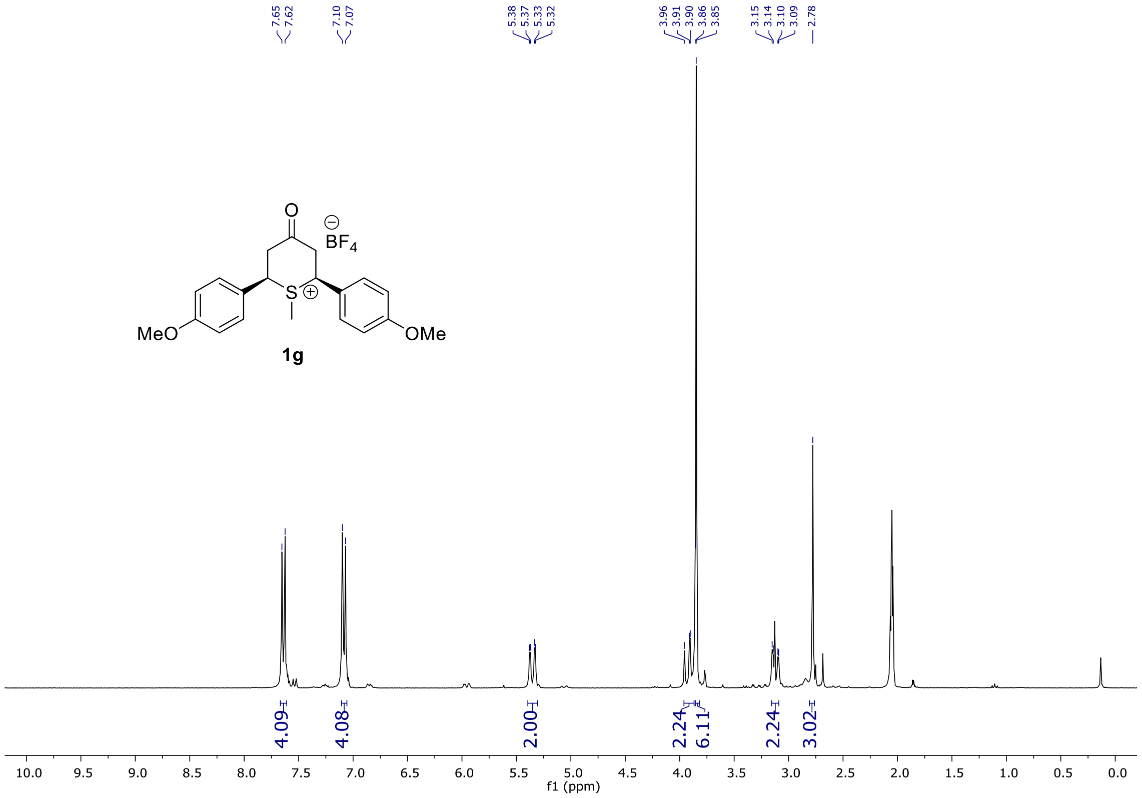
1g
BF4
MeO
OMe
f1 (ppm)
4.09
4.08
2.00
2.24
6.11
2.24
3.02

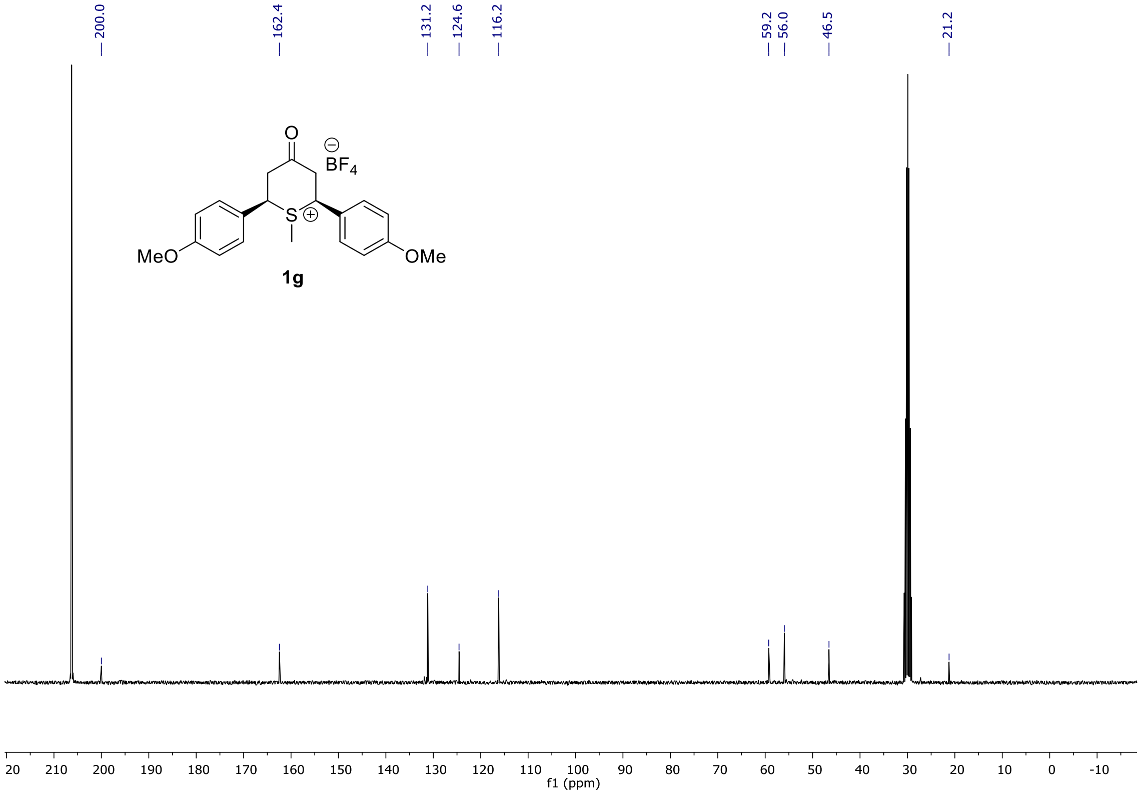
1g
BF4
MeO
OMe
f1 (ppm)
200.0
162.4
131.2
124.6
116.2
59.2
56.0
46.5
21.2

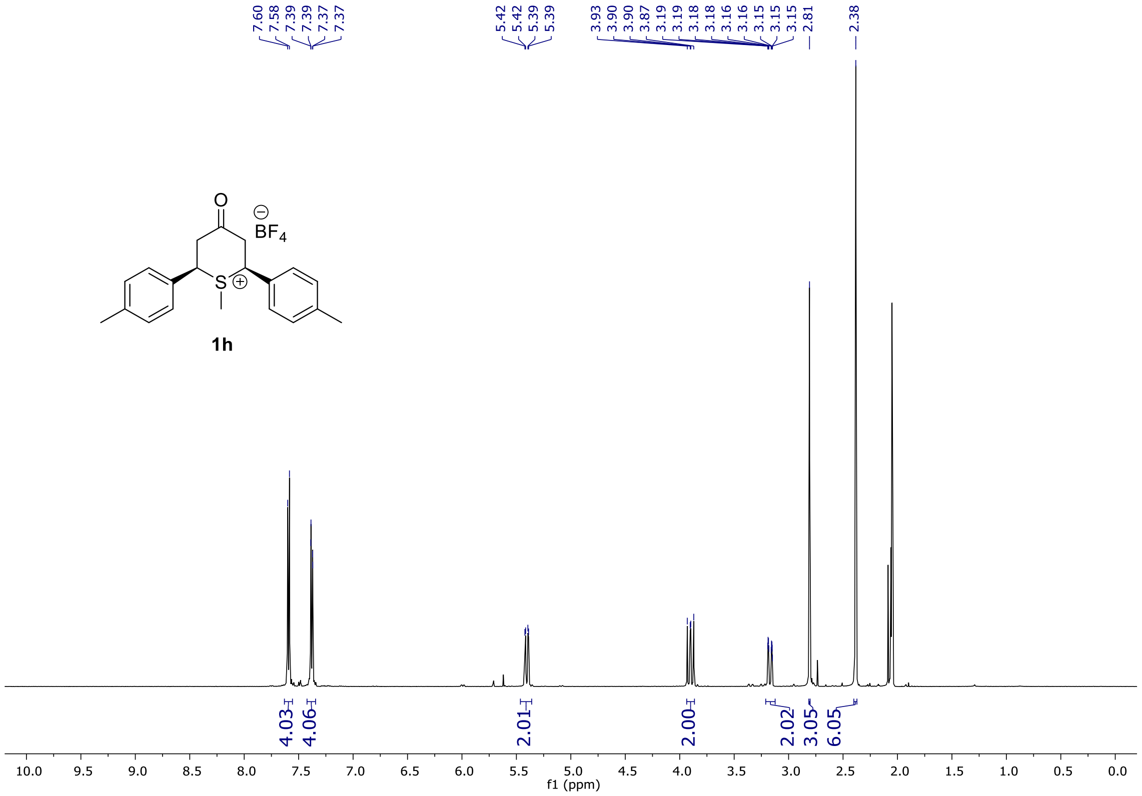
7.60
7.58
7.39
7.39
7.37
7.37
5.42
5.42
5.39
5.39
3.93
3.90
3.90
3.87
3.19
3.19
3.18
3.18
3.16
3.15
3.15
3.15
2.81
2.38
BF4
S
1h
4.03
4.06
2.01
2.00
2.02
3.05
6.05
f1 (ppm)

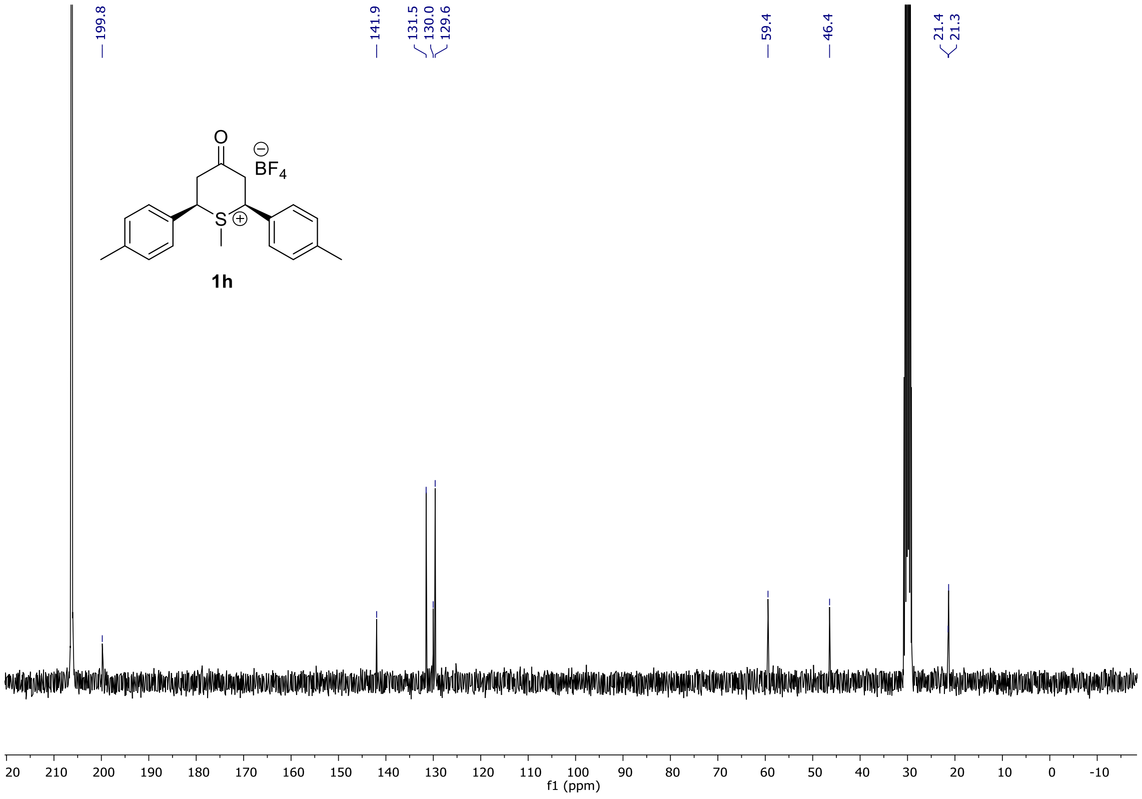
199.8
141.9
131.5
130.0
129.6
59.4
46.4
21.4
21.3
BF4
S
1h
f1 (ppm)

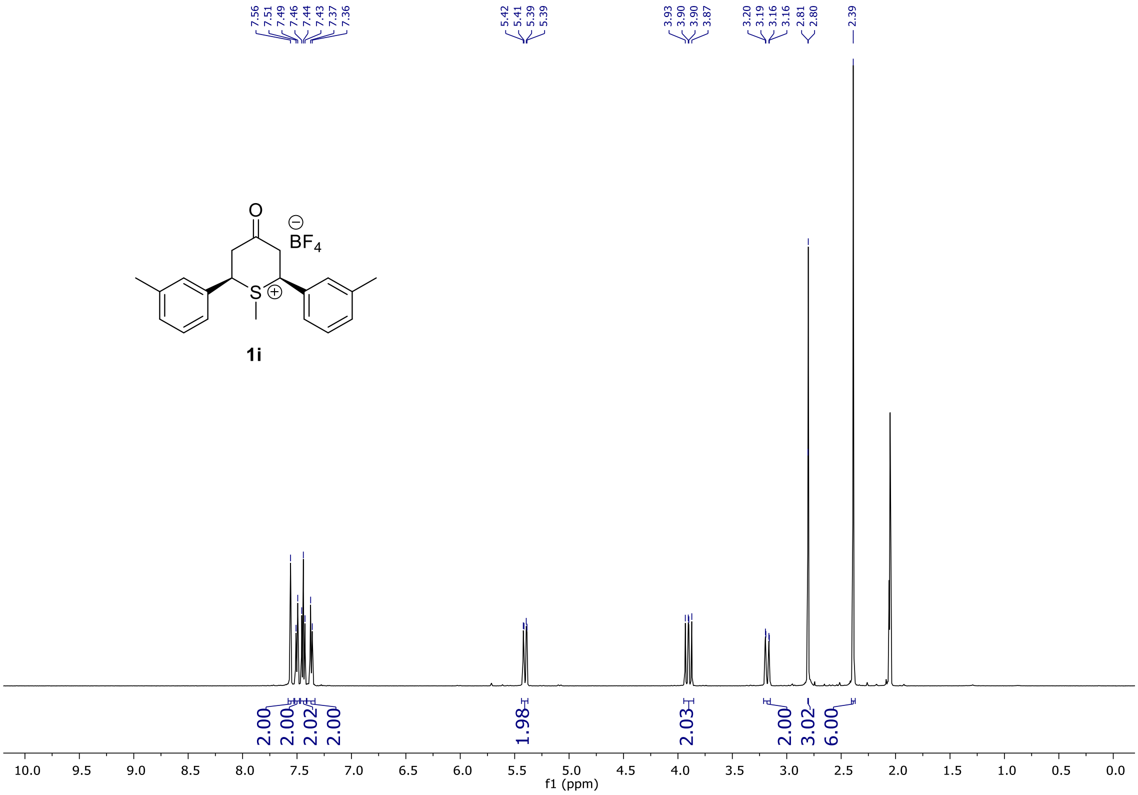
7.56 7.51 7.49 7.46 7.44 7.43 7.37 7.36
5.42 5.41 5.39 5.39
3.93 3.90 3.90 3.87
3.20 3.19 3.16 3.16 2.81 2.80
2.39
O
BF4
S
1i
2.00 2.00 2.02 2.00
1.98
2.03
2.00 3.02 6.00
f1 (ppm)

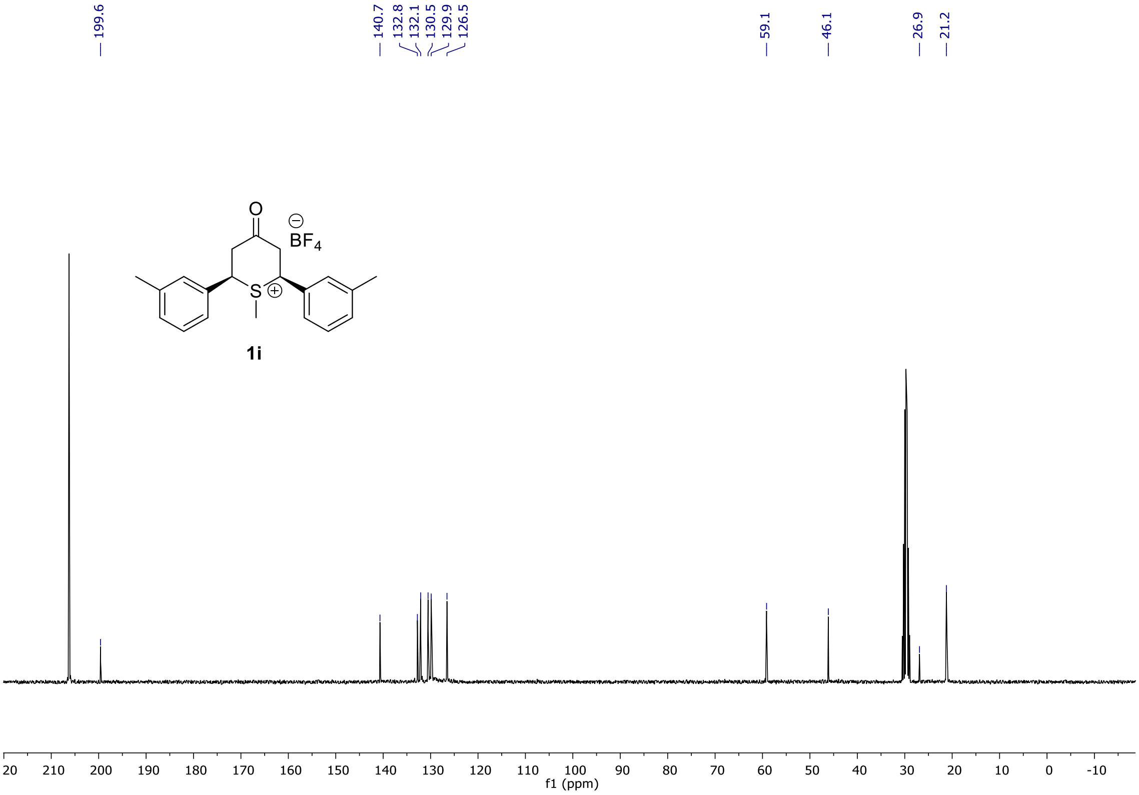
199.6
140.7 132.8 132.1 130.5 129.9 126.5
59.1
46.1
26.9
21.2
O
BF4
S
1i
f1 (ppm)

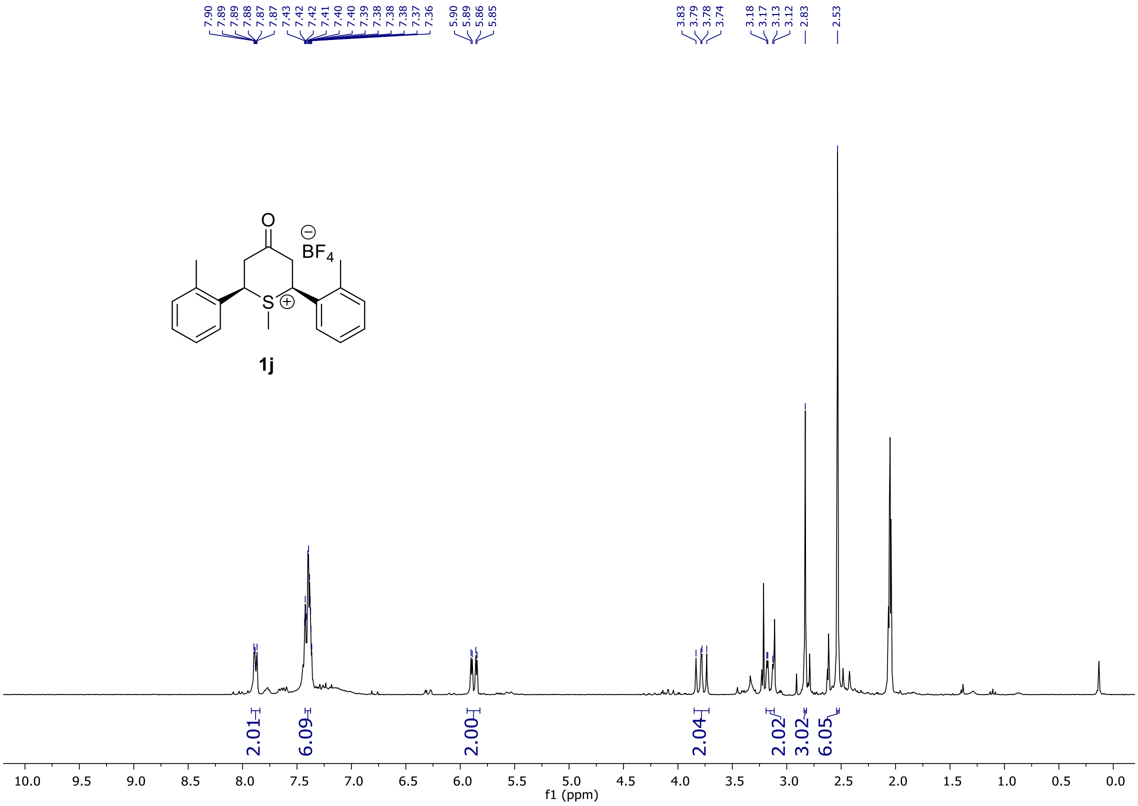
7.90 7.89 7.89 7.88 7.87 7.87 7.43 7.42 7.42 7.41 7.40 7.40 7.39 7.38 7.38 7.38 7.37 7.36 5.90 5.89 5.86 5.85
3.83 3.79 3.78 3.74 3.18 3.17 3.13 3.12 2.83 2.53
O
BF4
S
1j
2.01
6.09
2.00
2.04
2.02
3.02
6.05
f1 (ppm)

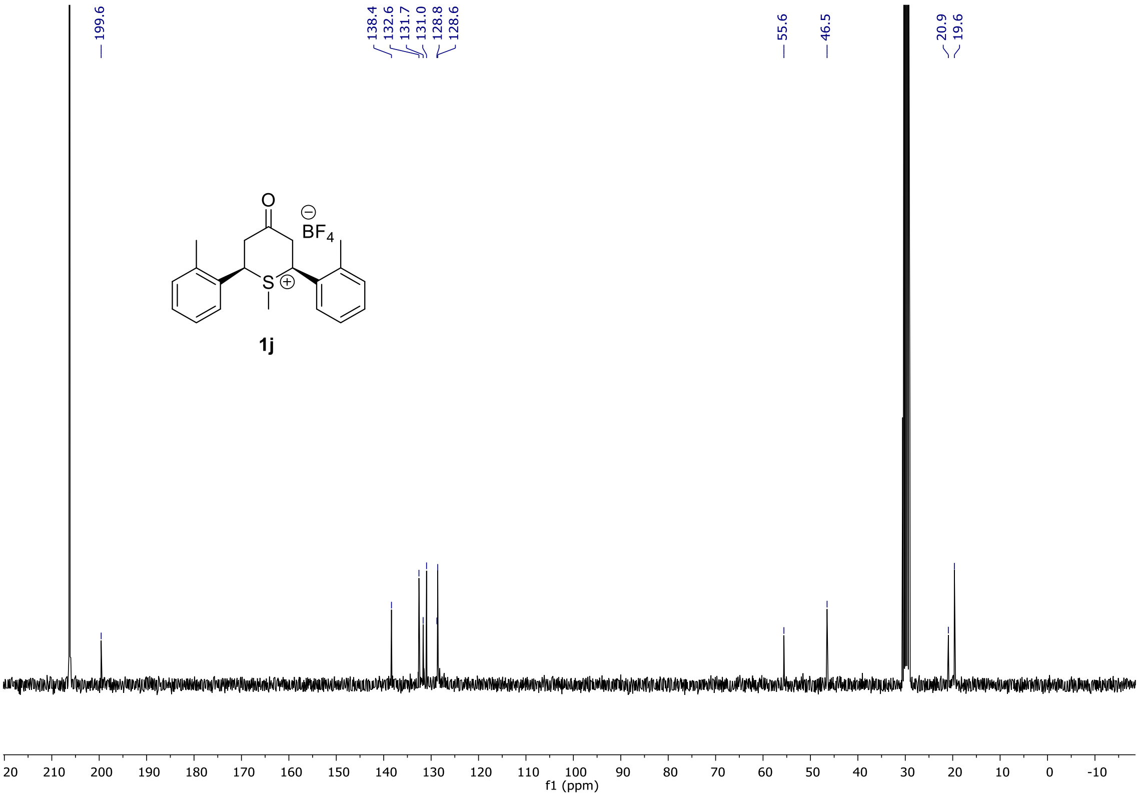
199.6
138.4 132.6 131.7 131.0 128.8 128.6
55.6
46.5
20.9 19.6
O
BF4
S
1j
f1 (ppm)

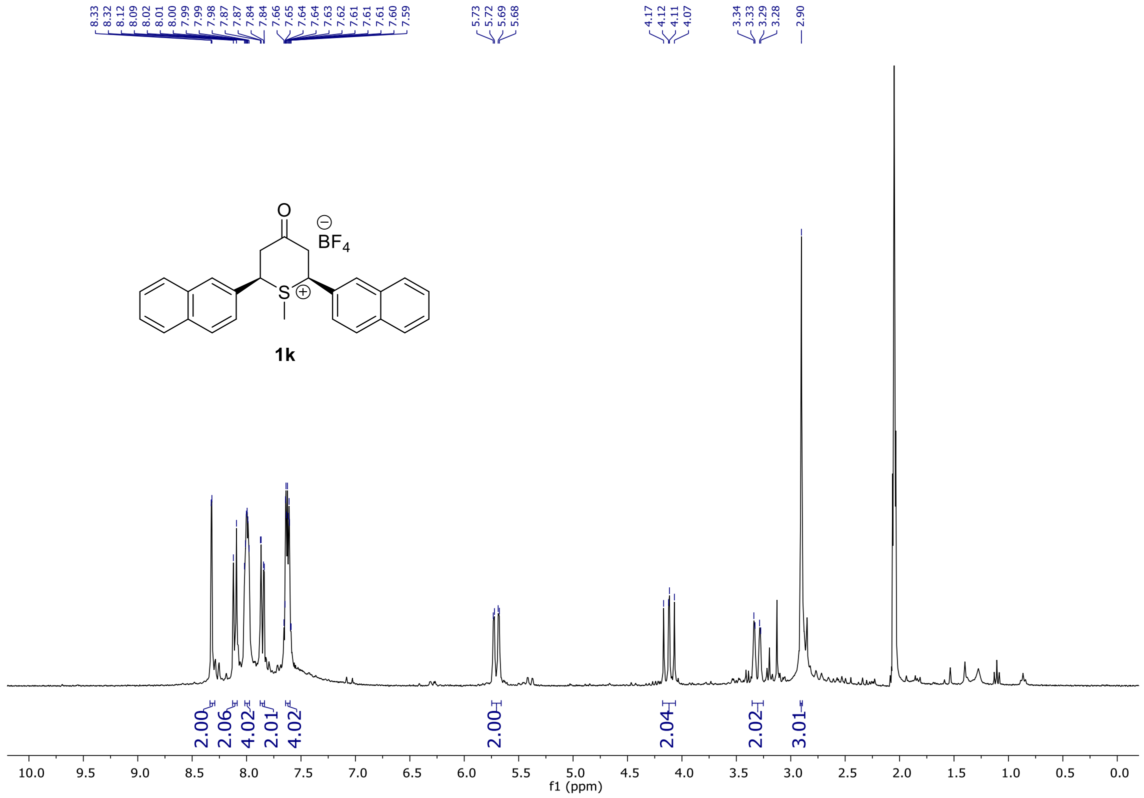
8.33 8.32 8.12 8.09 8.02 8.01 8.00 7.99 7.99 7.98 7.87 7.87 7.84 7.84 7.66 7.65 7.64 7.64 7.63 7.62 7.61 7.61 7.61 7.60 7.59
5.73 5.72 5.69 5.68
4.17 4.12 4.11 4.07
3.34 3.33 3.29 3.28
2.90
BF4
S
O
1k
2.00 2.06 4.02 2.01 4.02 2.00 2.04 2.02 3.01
10.0 9.5 9.0 8.5 8.0 7.5 7.0 6.5 6.0 5.5 5.0 4.5 4.0 3.5 3.0 2.5 2.0 1.5 1.0 0.5 0.0
f1 (ppm)

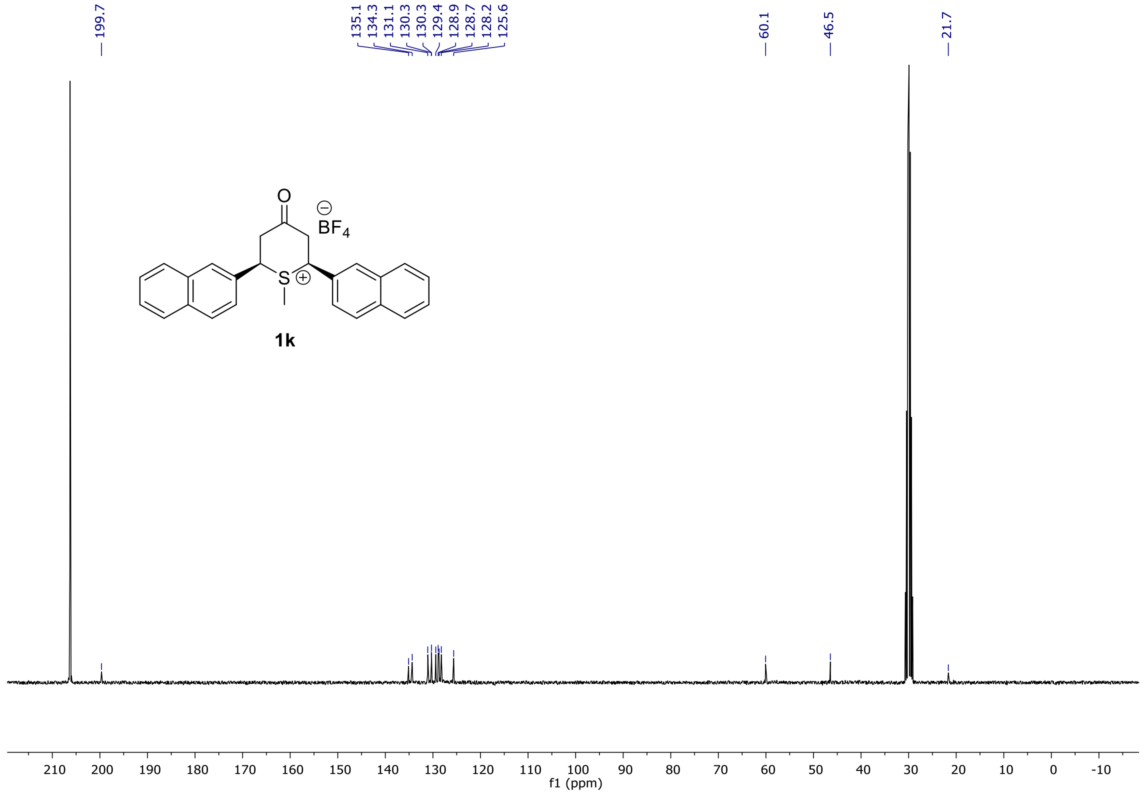
199.7
135.1 134.3 131.1 130.3 130.3 129.4 128.9 128.7 128.2 125.6
60.1
46.5
21.7
BF4
S
O
1k
210 200 190 180 170 160 150 140 130 120 110 100 90 80 70 60 50 40 30 20 10 0 -10
f1 (ppm)

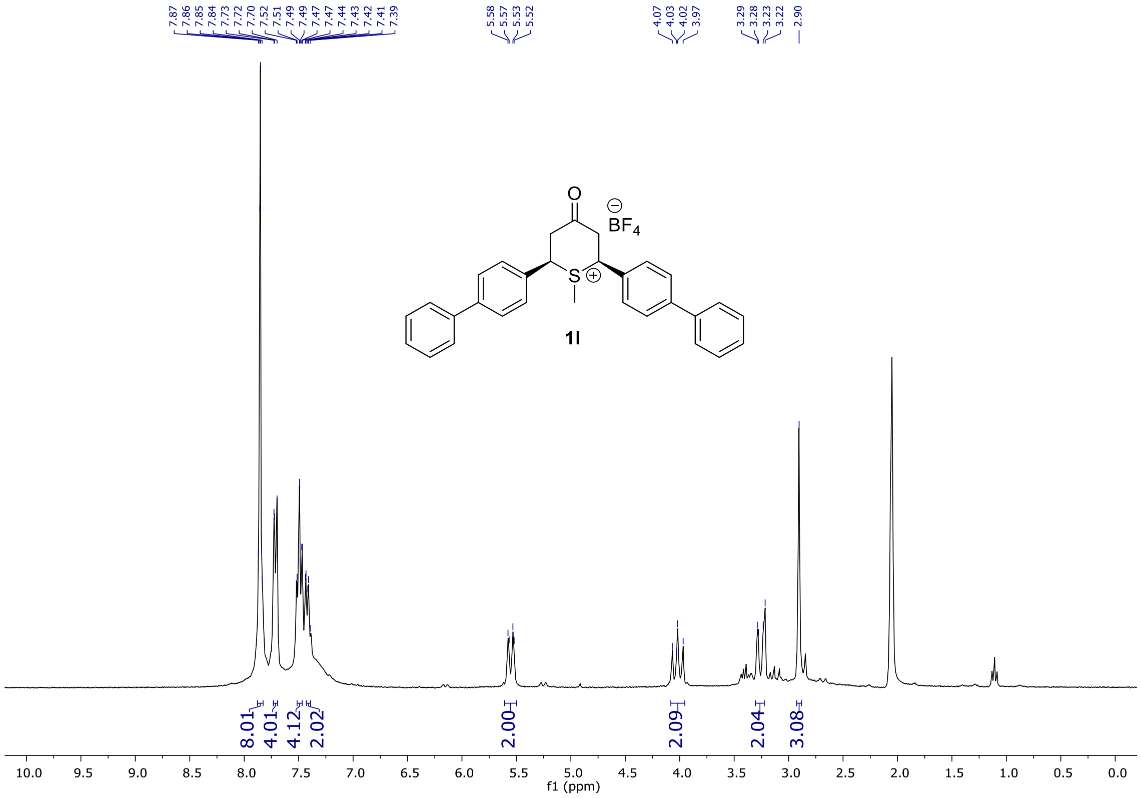
7.87 7.86 7.85 7.84 7.73 7.72 7.70 7.52 7.51 7.49 7.49 7.47 7.47 7.44 7.43 7.42 7.41 7.39
5.58 5.57 5.53 5.52
4.07 4.03 4.02 3.97
3.29 3.28 3.23 3.22
2.90
O
BF4
S
1l
8.01
4.01
4.12
2.02
2.00
2.09
2.04
3.08
10.0 9.5 9.0 8.5 8.0 7.5 7.0 6.5 6.0 5.5 5.0 4.5 4.0 3.5 3.0 2.5 2.0 1.5 1.0 0.5 0.0
f1 (ppm)

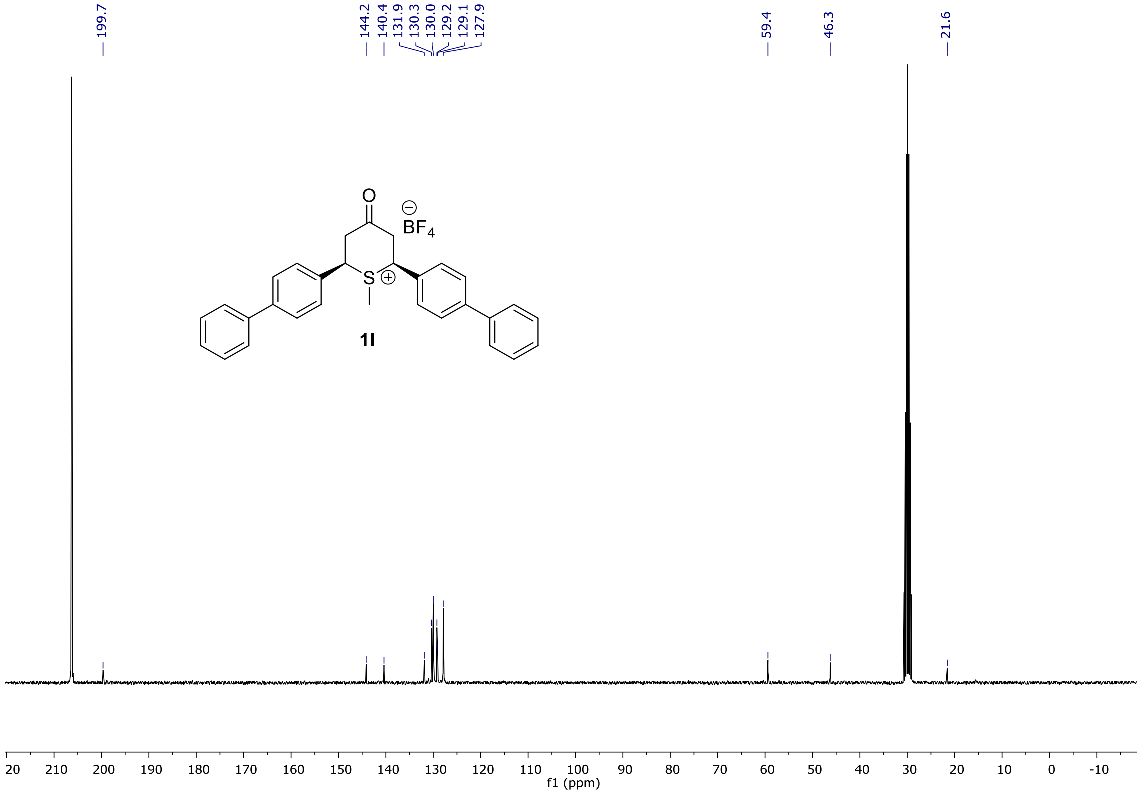
199.7
144.2 140.4 131.9 130.3 130.0 129.2 129.1 127.9
59.4
46.3
21.6
O
BF4
S
1l
20 210 200 190 180 170 160 150 140 130 120 110 100 90 80 70 60 50 40 30 20 10 0 -10
f1 (ppm)

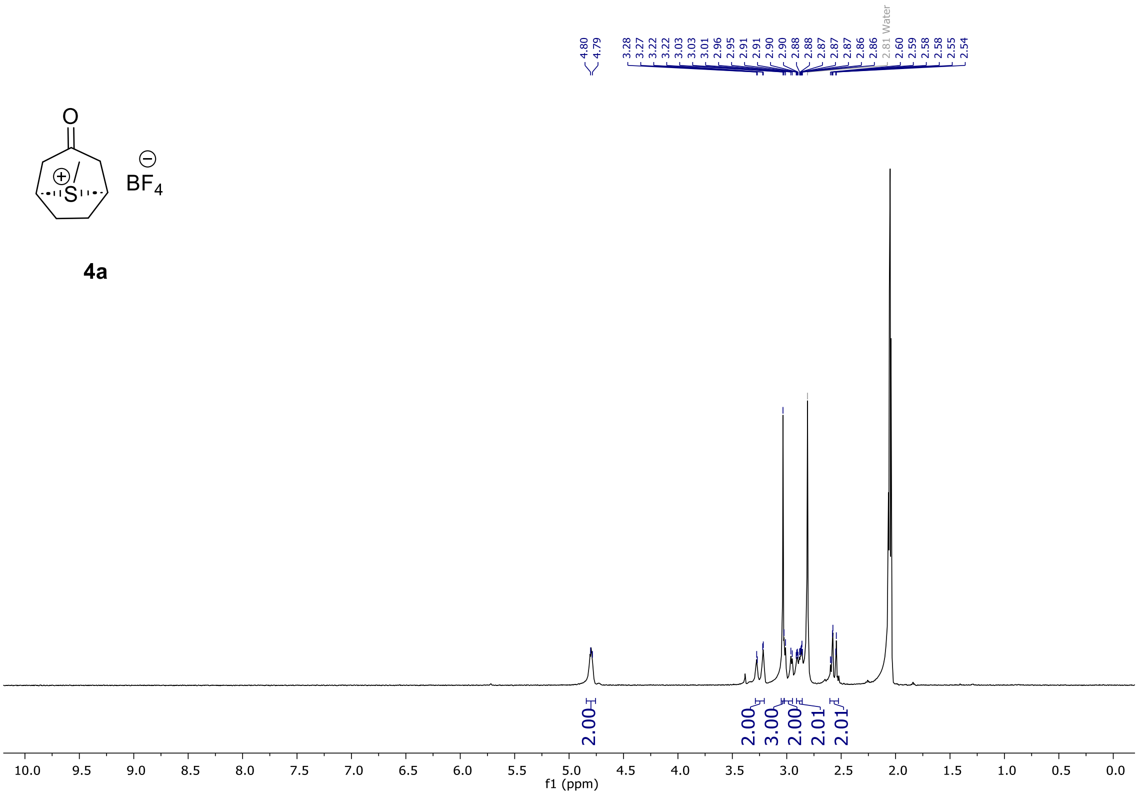
4.80
4.79
3.28
3.27
3.22
3.22
3.03
3.03
3.01
2.96
2.95
2.91
2.91
2.90
2.90
2.88
2.88
2.87
2.87
2.87
2.86
2.81 Water
2.60
2.59
2.58
2.58
2.55
2.54
BF4
4a
2.00
2.00
3.00
2.00
2.01
2.01
f1 (ppm)

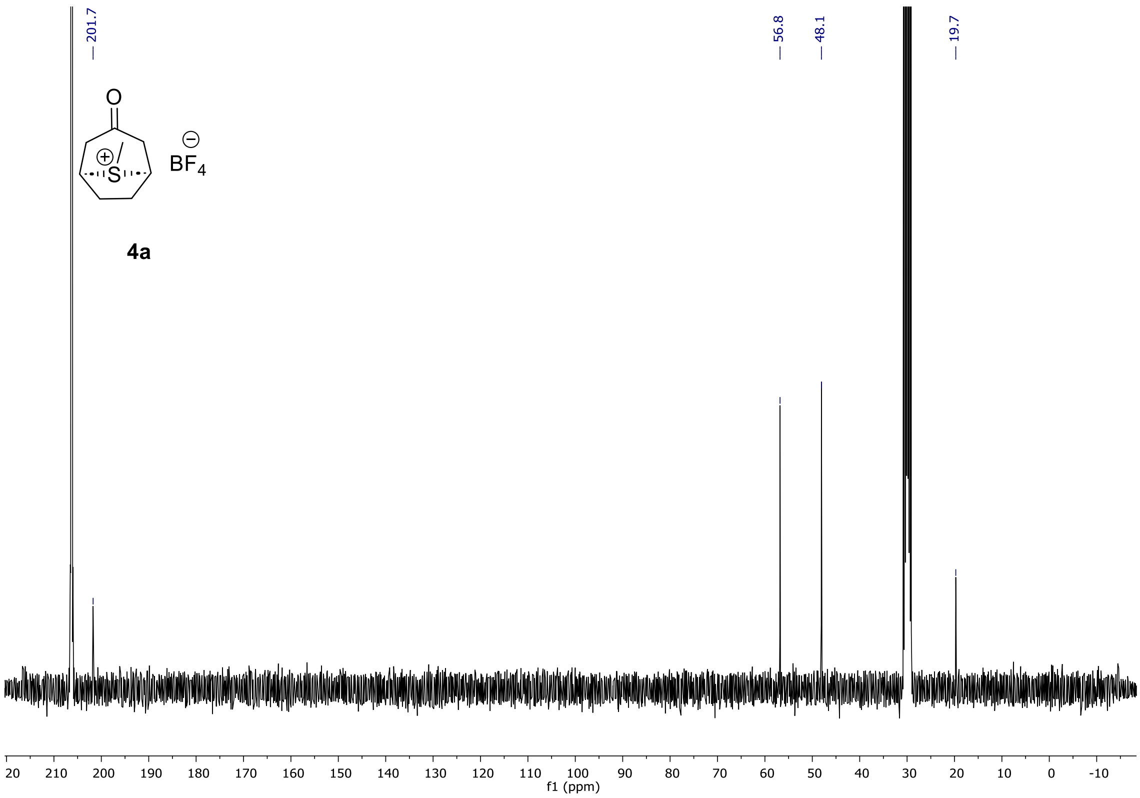
201.7
56.8
48.1
19.7
BF4
4a
f1 (ppm)

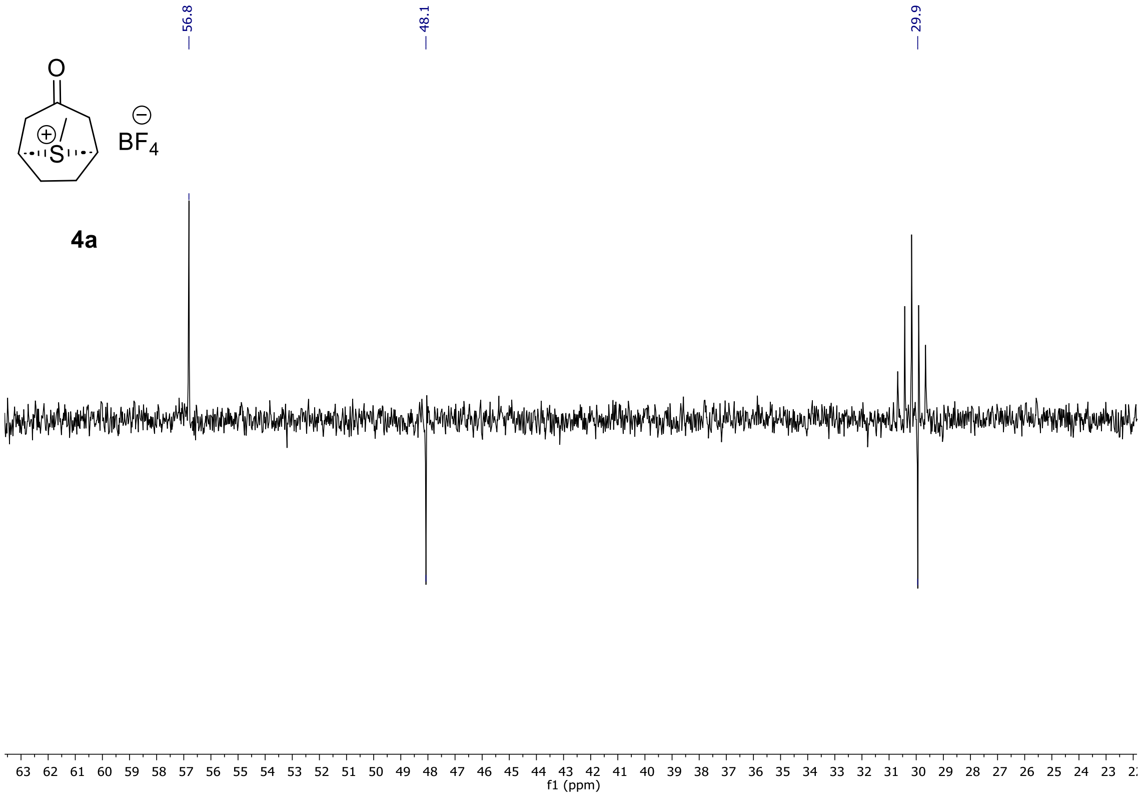

56.8
48.1
29.9
O
BF4
S
63 62 61 60 59 58 57 56 55 54 53 52 51 50 49 48 47 46 45 44 43 42 41 40 39 38 37 36 35 34 33 32 31 30 29 28 27 26 25 24 23
f1 (ppm)

4a

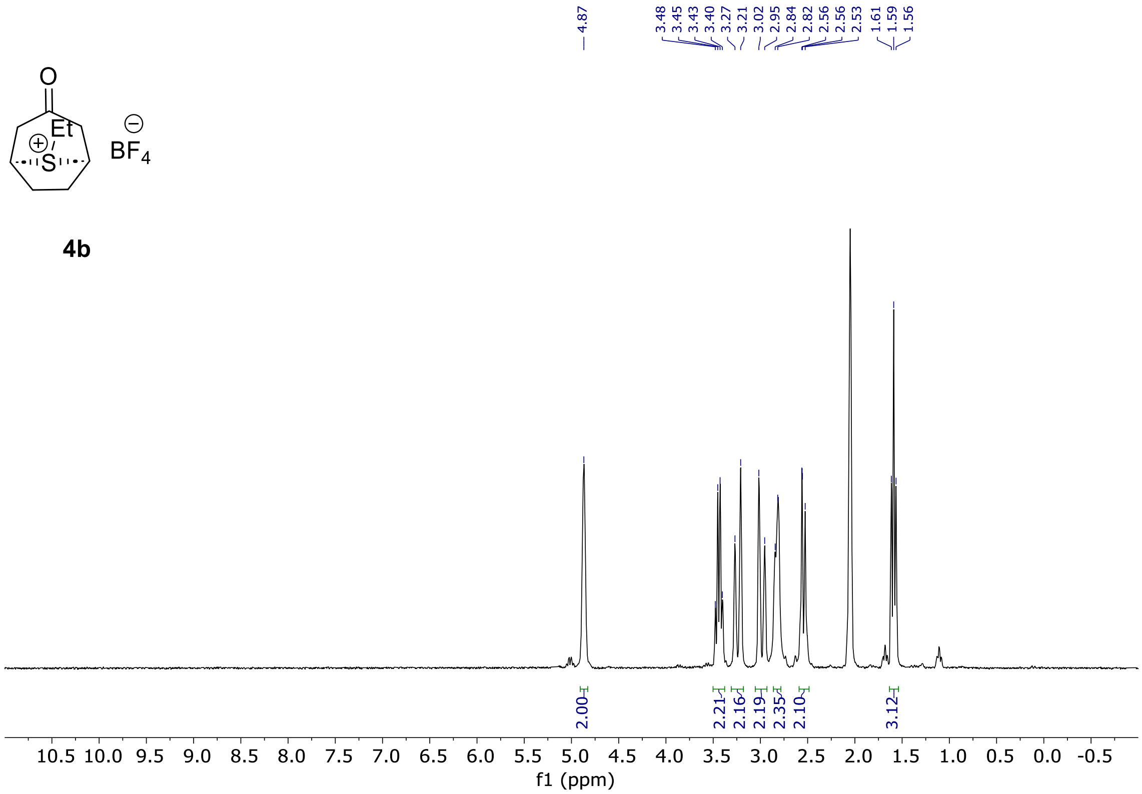
O
Et
S
BF4
4b
4.87
3.48
3.45
3.43
3.40
3.27
3.21
3.02
2.95
2.84
2.82
2.56
2.56
2.53
1.61
1.59
1.56
2.00
2.21
2.16
2.19
2.35
2.10
3.12
10.5 10.0 9.5 9.0 8.5 8.0 7.5 7.0 6.5 6.0 5.5 5.0 4.5 4.0 3.5 3.0 2.5 2.0 1.5 1.0 0.5 0.0 -0.5
f1 (ppm)

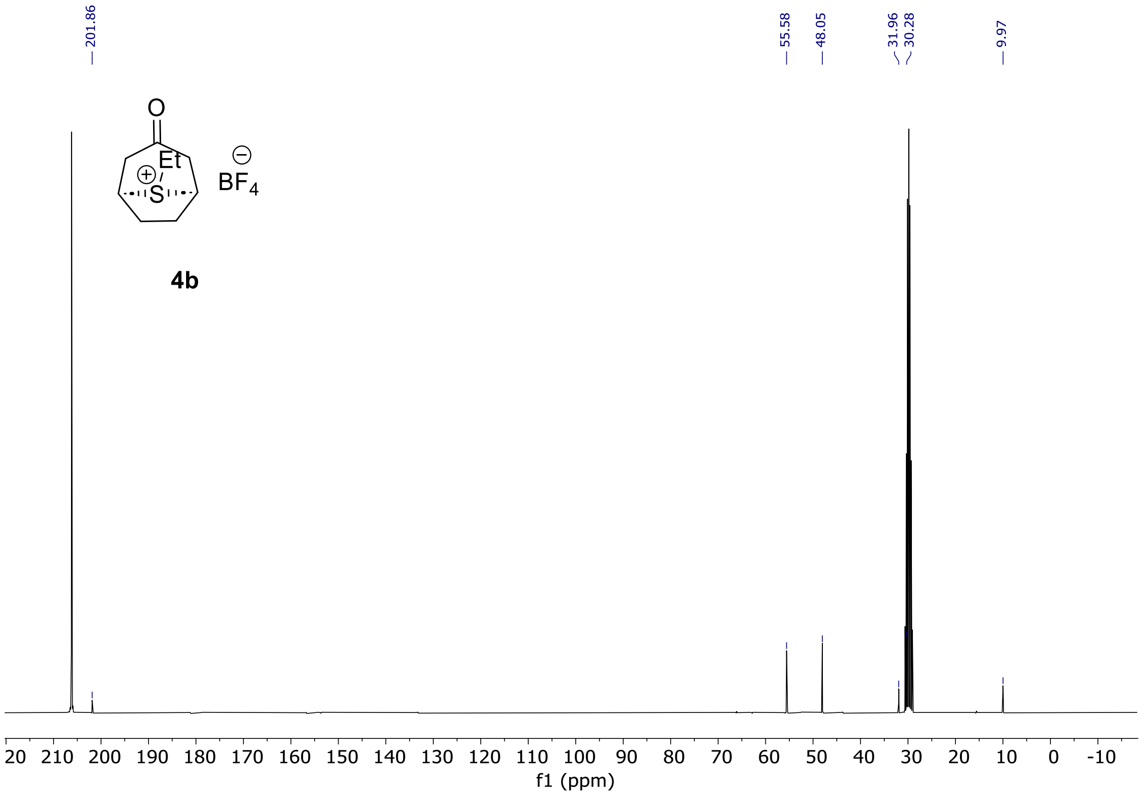
O
Et
S
BF4
4b
201.86
55.58
48.05
31.96
30.28
9.97
20 210 200 190 180 170 160 150 140 130 120 110 100 90 80 70 60 50 40 30 20 10 0 -10
f1 (ppm)

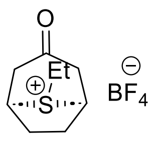

4b

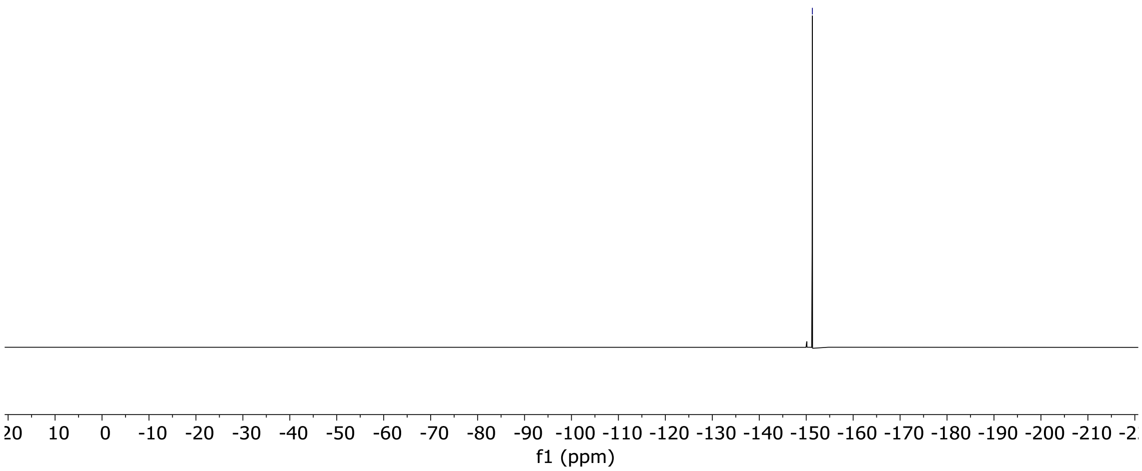
-151.31
20 10 0 -10 -20 -30 -40 -50 -60 -70 -80 -90 -100 -110 -120 -130 -140 -150 -160 -170 -180 -190 -200 -210
f1 (ppm)

13.3. Products: NMR and SFC chromatograms

3a:

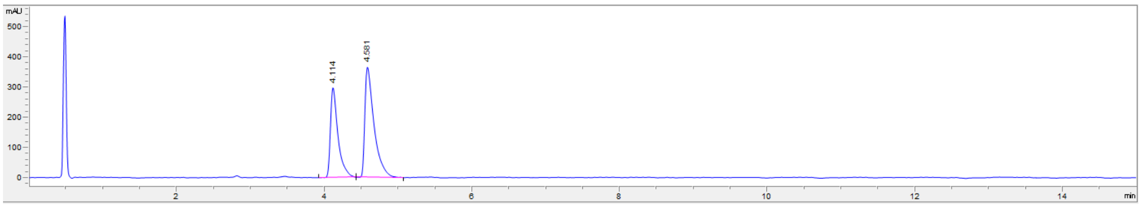

#	Time	Type	Area	Height	Width	Area%	Symmetry
1	4.114	BB	2131.6	299.3	0.1031	49.750	0.473
2	4.581	MM	2153	314.7	0.114	50.250	0.365

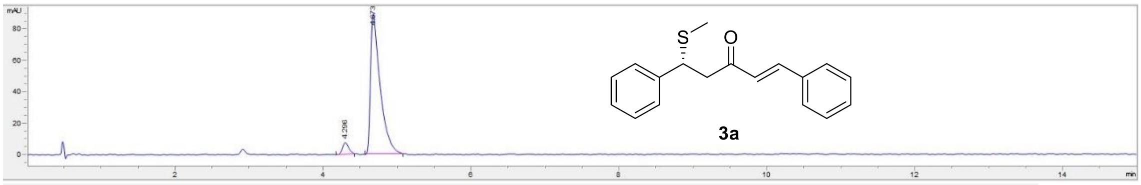

3a

#	Time	Type	Area	Height	Width	Area%	Symmetry
1	4.296	BB	43.6	7.3	0.0917	5.145	0.796
2	4.673	BB	803.7	89.3	0.131	94.855	0.347

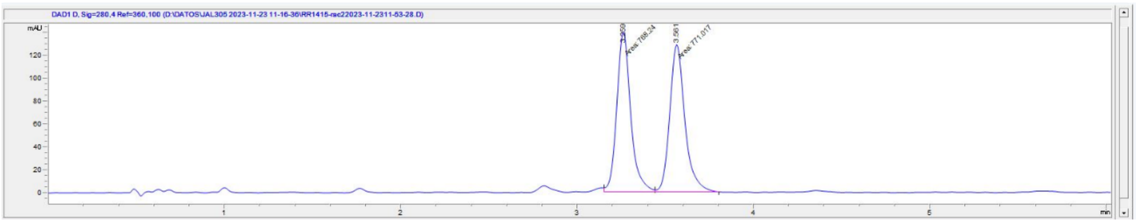

When performed at 0.2 mmol scale:

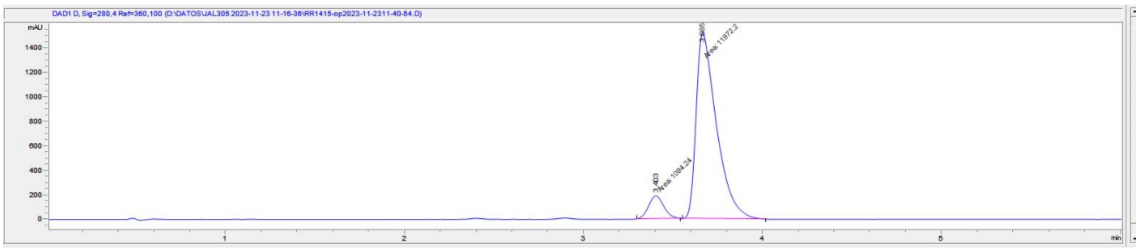

#	Time	Type	Area	Height	Width	Area%	Symmetry
1	3.403	MM	1094.2	186	0.098	8.374	0.89
2	3.665	MM	11972.2	1520	0.1313	91.626	0.459

When performed at 1.0 mmol scale:

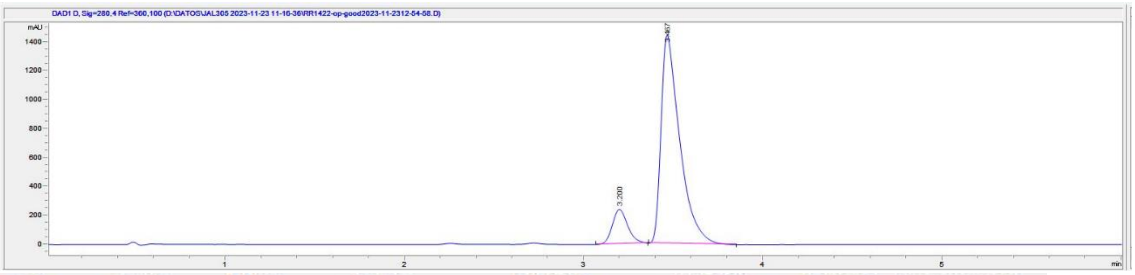

#	Time	Type	Area	Height	Width	Area%	Symmetry
1	3.2	BB	1427.4	239.9	0.0914	11.993	0.799
2	3.467	BB	10475.1	1449.2	0.1063	88.007	0.451

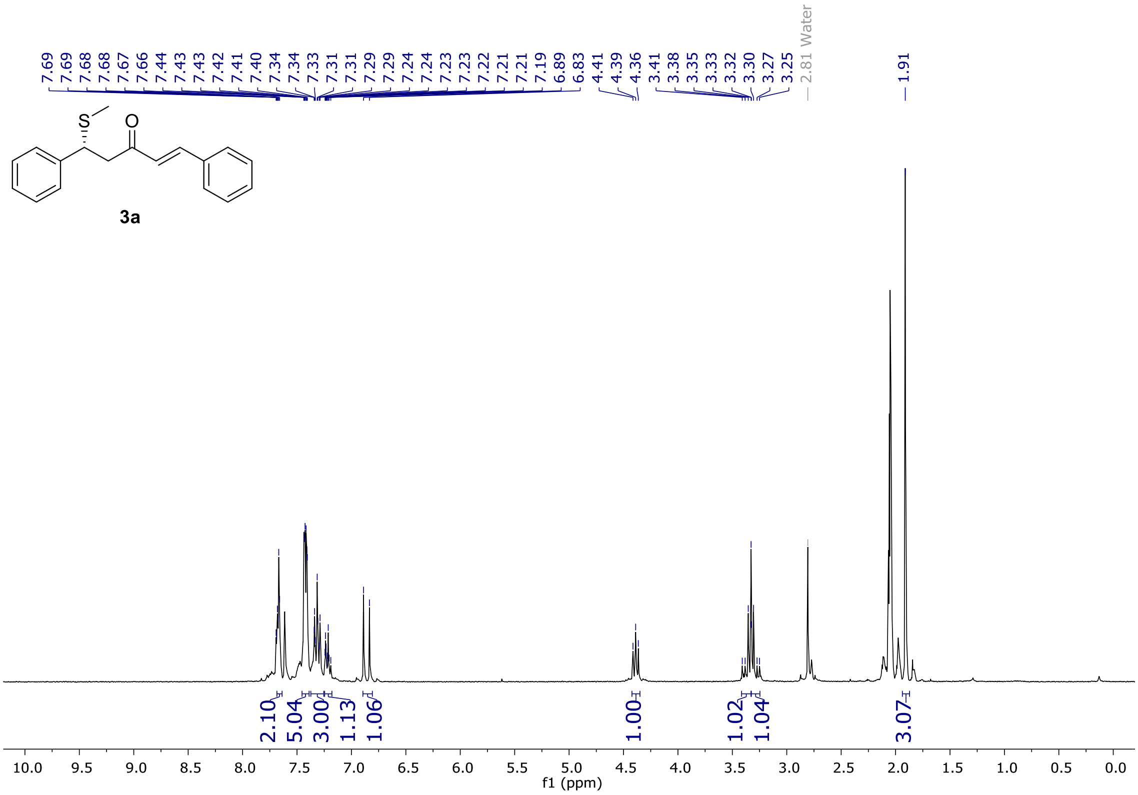
7.69
7.69
7.68
7.68
7.67
7.66
7.44
7.43
7.43
7.42
7.41
7.40
7.34
7.34
7.33
7.31
7.31
7.29
7.29
7.24
7.24
7.23
7.23
7.22
7.21
7.21
7.19
6.89
6.83
4.41
4.39
4.36
3.41
3.38
3.35
3.33
3.32
3.30
3.27
3.25
2.81 Water
1.91
3a
2.10
5.04
3.00
1.13
1.06
1.00
1.02
1.04
3.07
f1 (ppm)

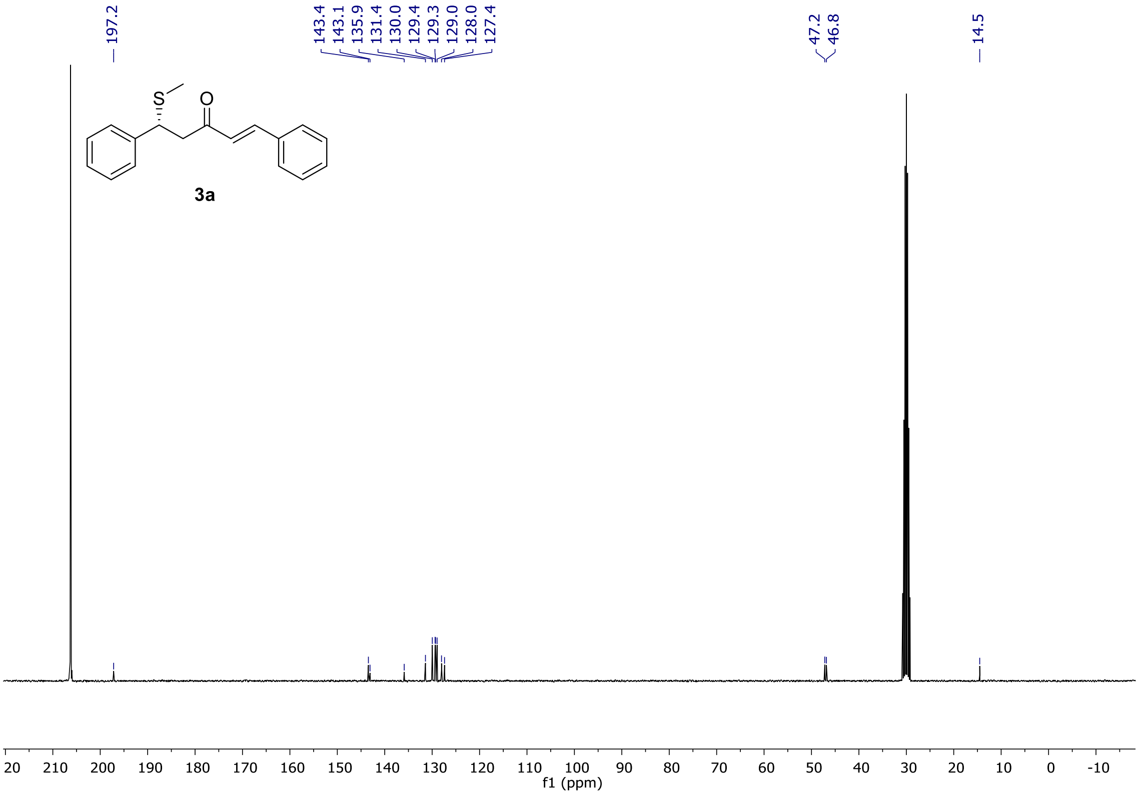
197.2
143.4
143.1
135.9
131.4
130.0
129.4
129.3
129.0
128.0
127.4
47.2
46.8
14.5
3a
f1 (ppm)

3b:

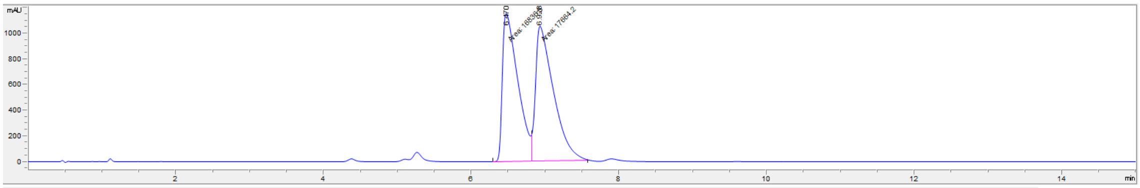

#	Time	Type	Area	Height	Width	Area%	Symmetry
1	6.47	MF	16836.5	1151.8	0.2436	48.800	0
2	6.928	FM	17664.2	1048.5	0.2808	51.200	0.321

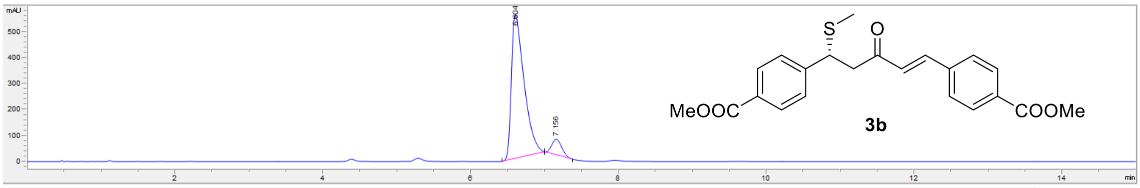

#	Time	Type	Area	Height	Width	Area%	Symmetry
1	6.604	BB	6628.2	556.3	0.177	92.094	0.423
2	7.156	BB	569	60.6	0.1495	7.906	0.76

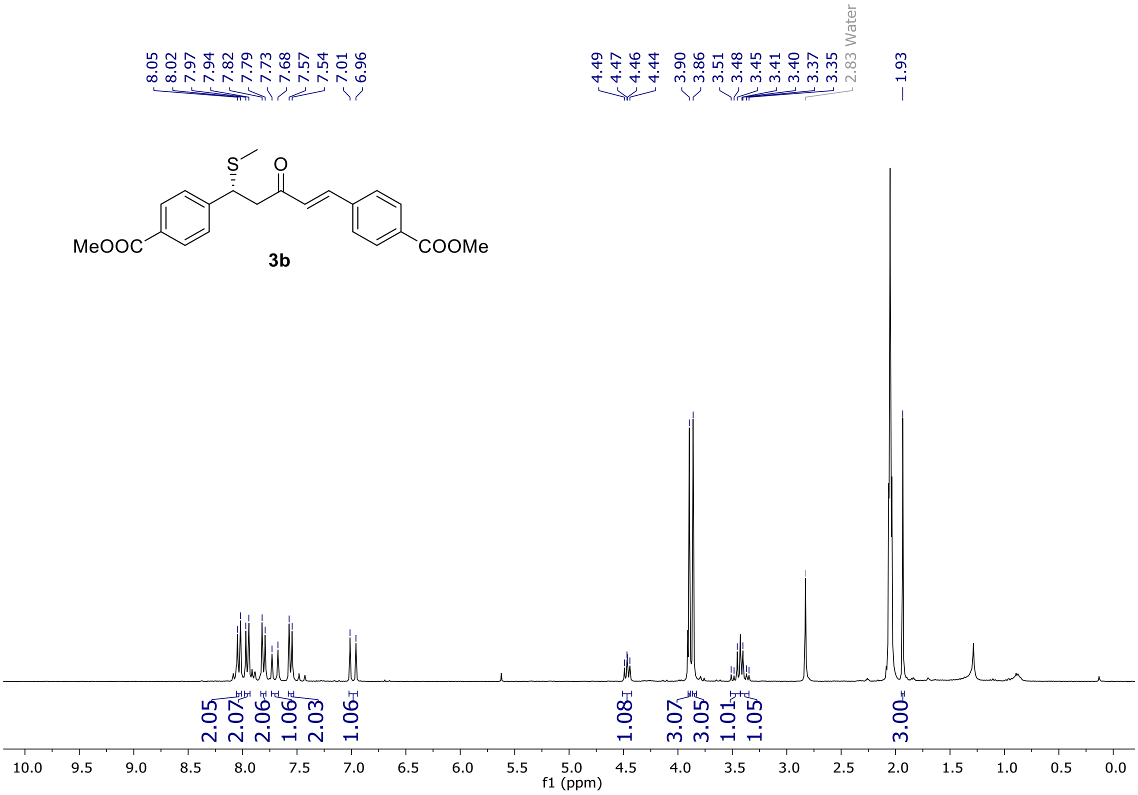

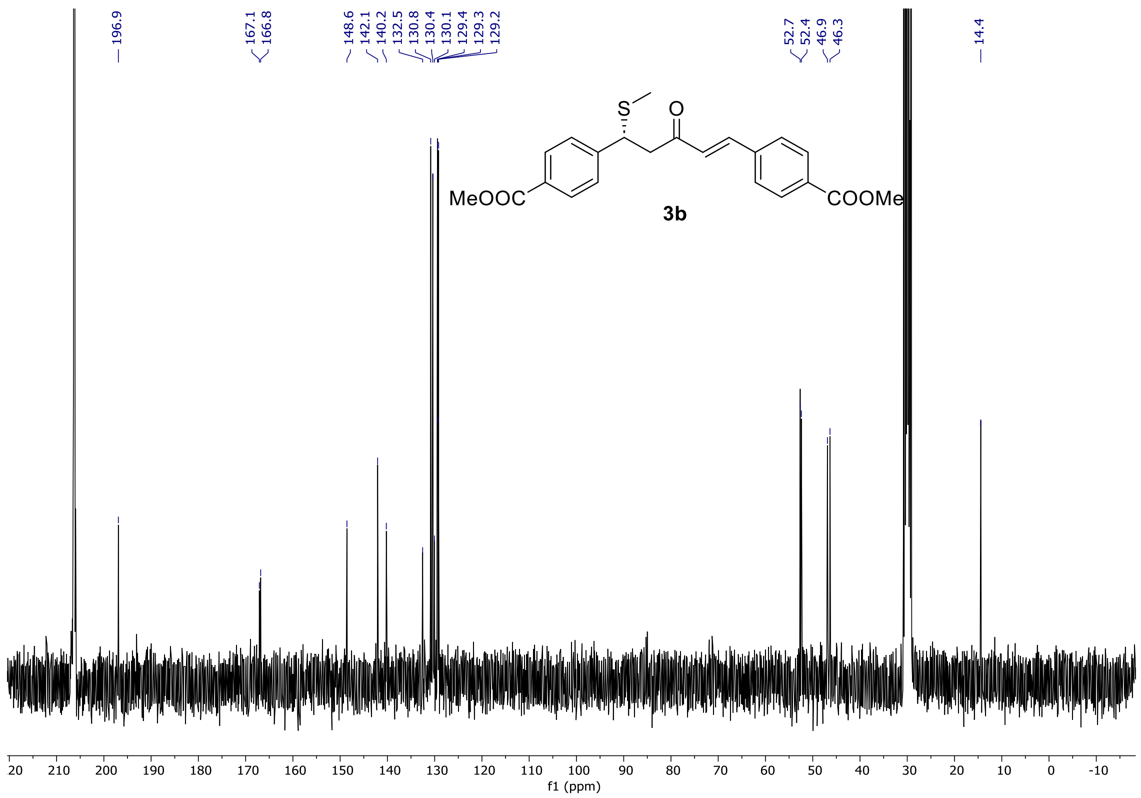

196.9
167.1
166.8
148.6
142.1
140.2
132.5
130.8
130.4
130.1
129.4
129.3
129.2
52.7
52.4
46.9
46.3
14.4
MeOOC
COOMe
3b
f1 (ppm)

3c:

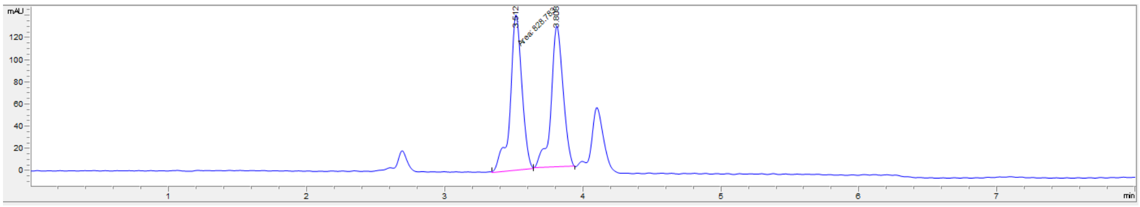

#	Time	Type	Area	Height	Width	Area%	Symmetry
1	3.512	MM	828.8	140.6	0.0983	51.668	0.978
2	3.808	BB	775.3	127.4	0.091	48.332	0.923

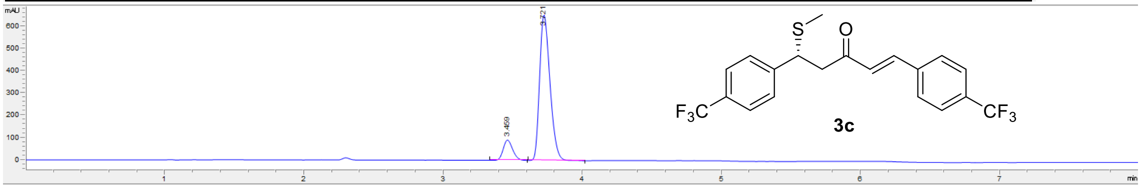

#	Time	Type	Area	Height	Width	Area%	Symmetry
1	3.459	BB	433.5	91.8	0.0731	11.291	0.792
2	3.721	BB	3405.7	657.2	0.0805	88.709	0.69

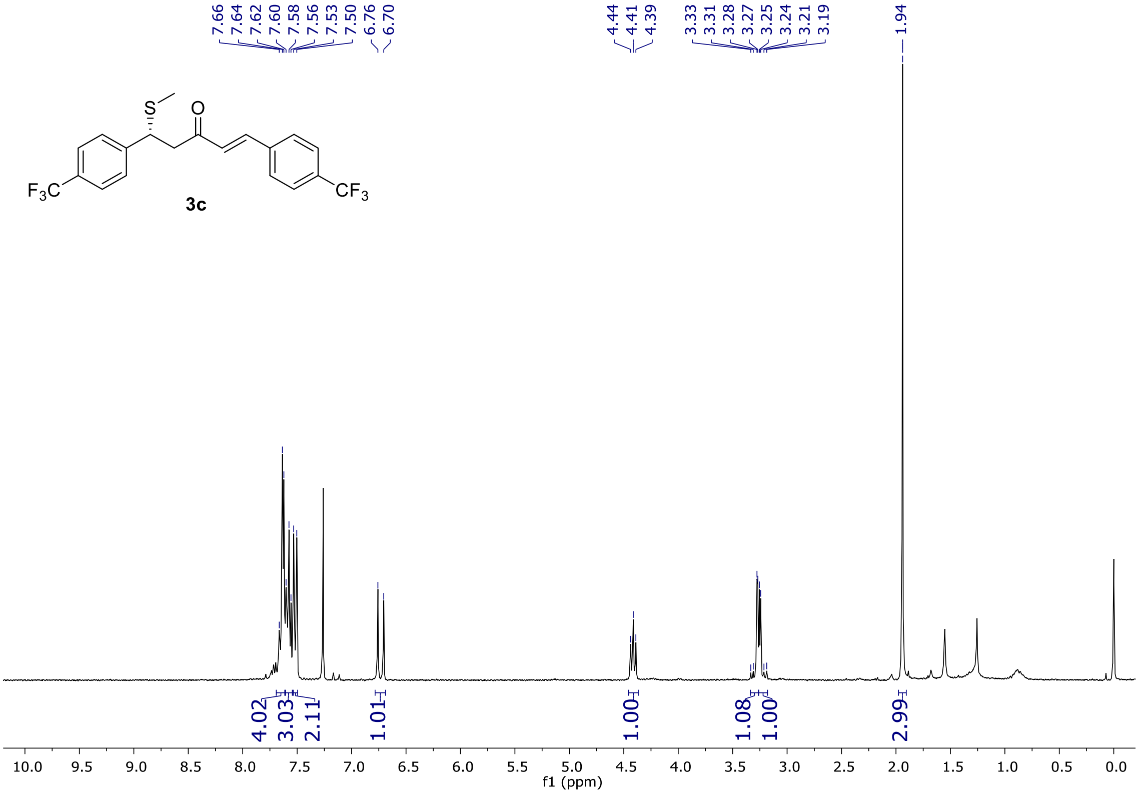

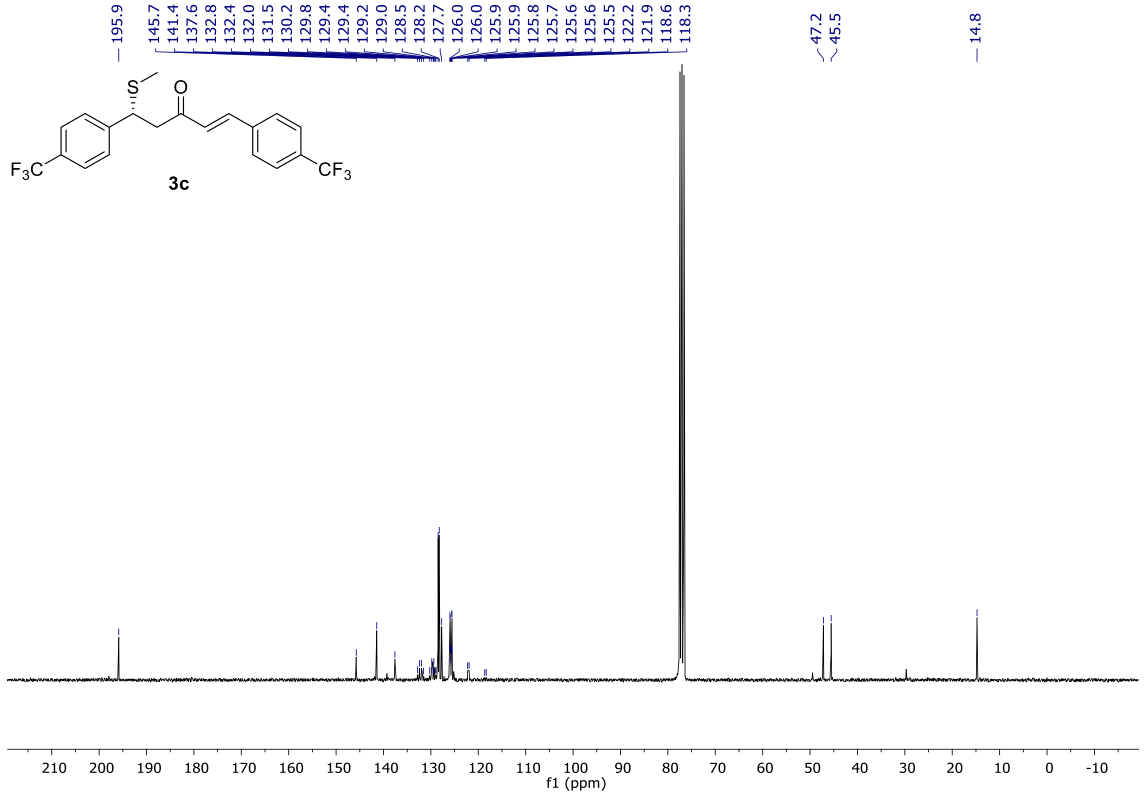

195.9
145.7
141.4
137.6
132.8
132.4
132.0
131.5
130.2
129.8
129.4
129.4
129.2
129.0
128.5
128.2
127.7
126.0
126.0
125.9
125.9
125.8
125.7
125.6
125.5
122.2
121.9
118.6
118.3
47.2
45.5
14.8
O
S
F3C
CF3
3c
210 200 190 180 170 160 150 140 130 120 110 100 90 80 70 60 50 40 30 20 10 0 -10
f1 (ppm)

3d:

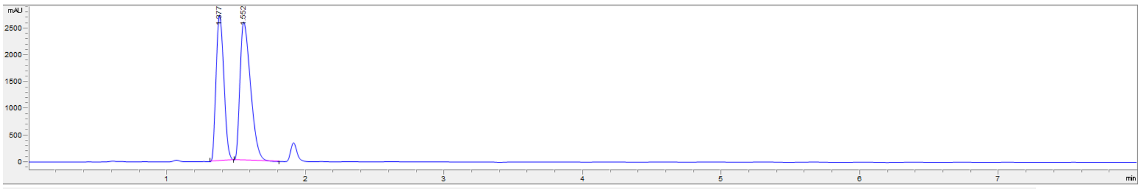

#	Time	Type	Area	Height	Width	Area%	Symmetry
1	1.377	BB	10737.3	2750.3	0.0635	49.132	0.772
2	1.552	MM	11116.9	2354	0.0787	50.868	0.57

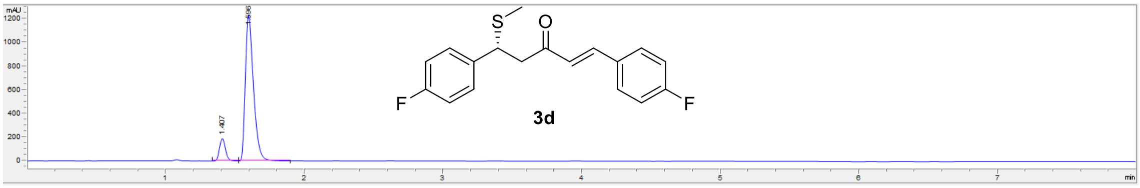

#	Time	Type	Area	Height	Width	Area%	Symmetry
1	1.407	BB	566.2	189.6	0.0468	10.112	0.874
2	1.596	BB	5032.8	1242	0.0613	89.888	0.625

Chromatograph of the major enantiomer of **3d** after separation in Chiralflash IA. *e.r.* was determined as >99:1.

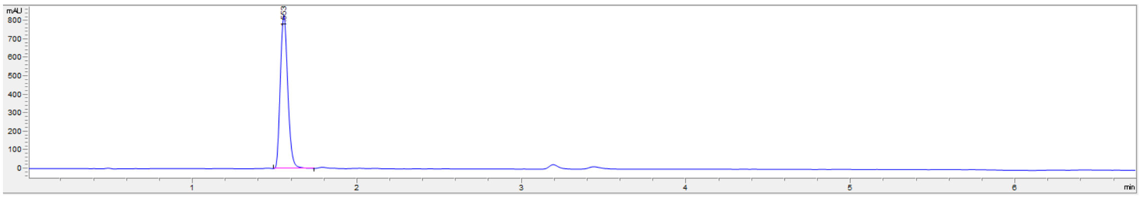

#	Time	Type	Area	Height	Width	Area%	Symmetry
1	1.553	BB	2615.6	833.1	0.0486	100.000	0.831

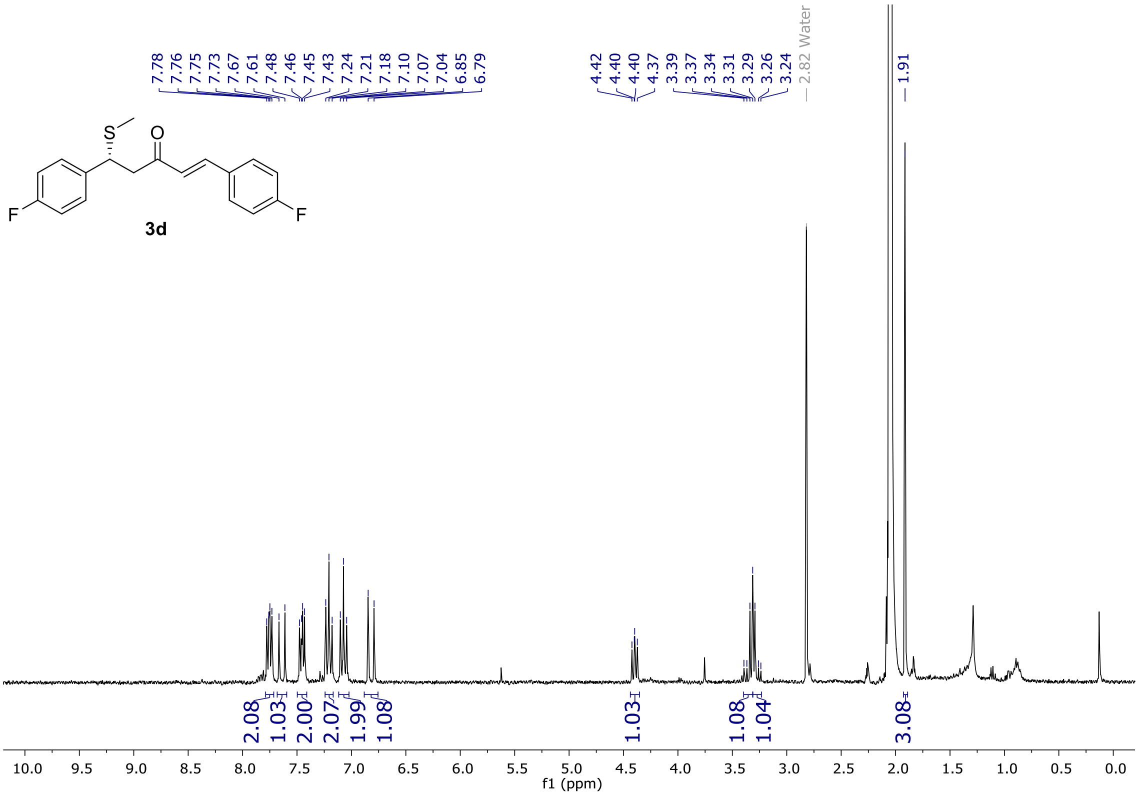
7.78
7.76
7.75
7.73
7.67
7.61
7.48
7.46
7.45
7.43
7.24
7.21
7.18
7.10
7.07
7.04
6.85
6.79
4.42
4.40
4.40
4.37
3.39
3.37
3.34
3.31
3.29
3.26
3.24
2.82 Water
1.91
S
O
F
F
3d
2.08
1.03
2.00
2.07
1.99
1.08
1.03
1.08
1.04
3.08
f1 (ppm)

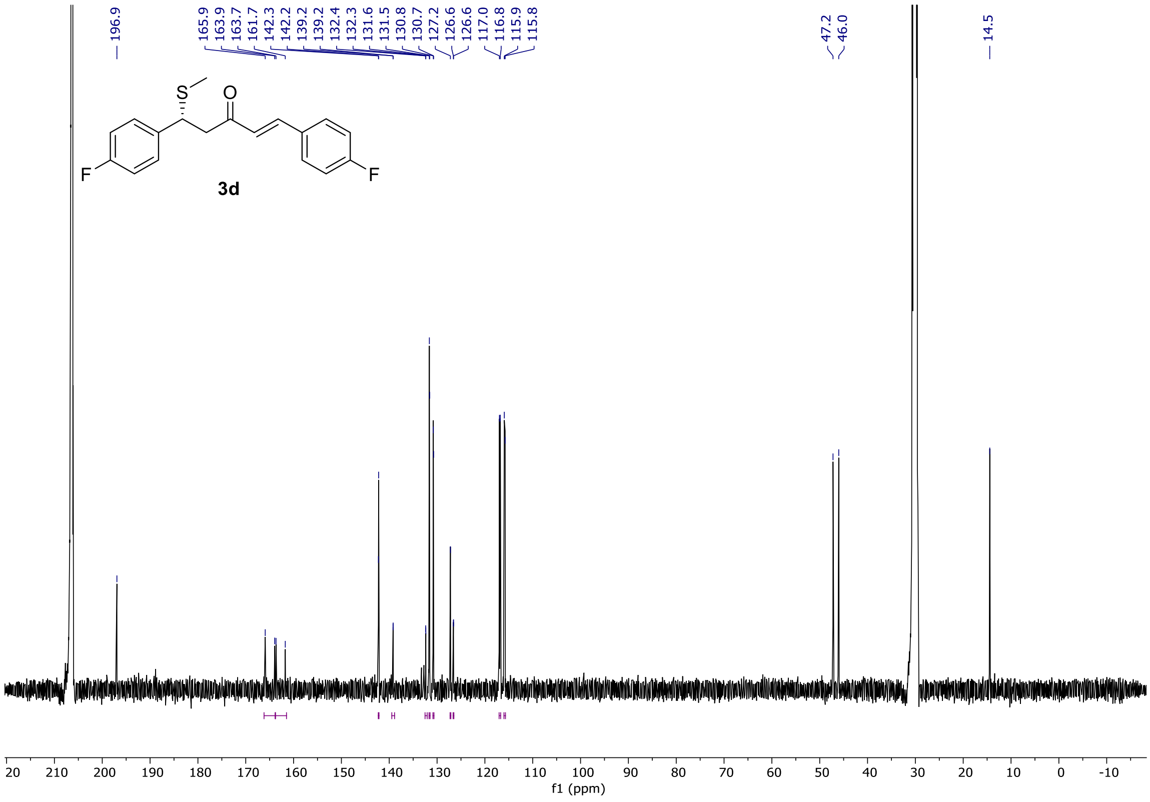
196.9
165.9
163.9
163.7
161.7
142.3
142.2
139.2
139.2
132.4
132.3
131.6
131.5
130.8
130.7
127.2
126.6
126.6
117.0
116.8
115.9
115.8
47.2
46.0
14.5
S
O
F
F
3d
f1 (ppm)

3e:

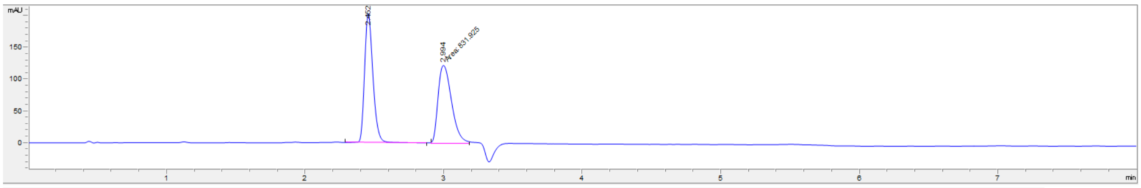

#	Time	Type	Area	Height	Width	Area%	Symmetry
1	2.452	BB	848.8	202.8	0.0648	50.503	0.734
2	2.994	MM	831.9	122.7	0.113	49.497	0.639

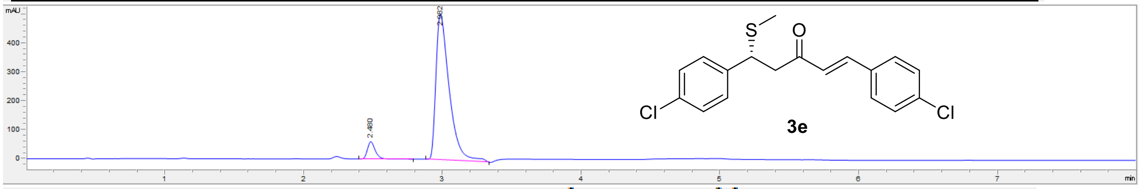

#	Time	Type	Area	Height	Width	Area%	Symmetry
1	2.48	BB	230.2	59.8	0.0589	6.397	0.731
2	2.982	BB	3369.2	504.5	0.0999	93.603	0.466

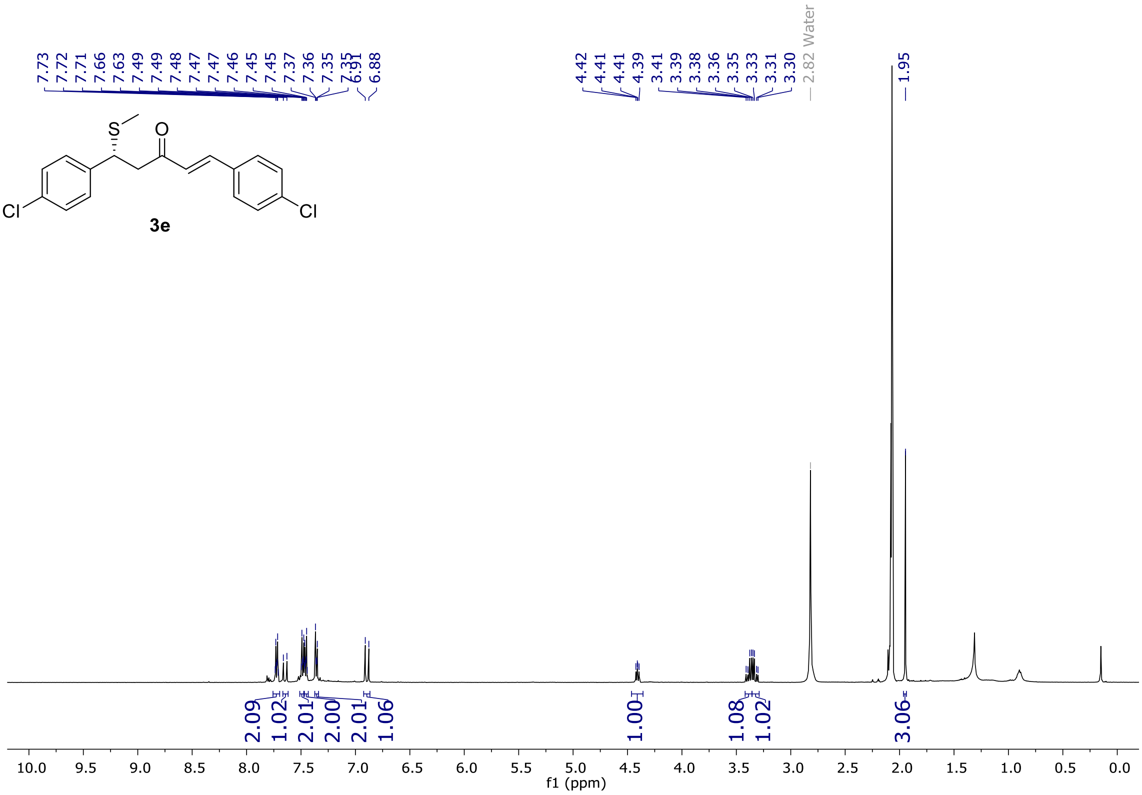

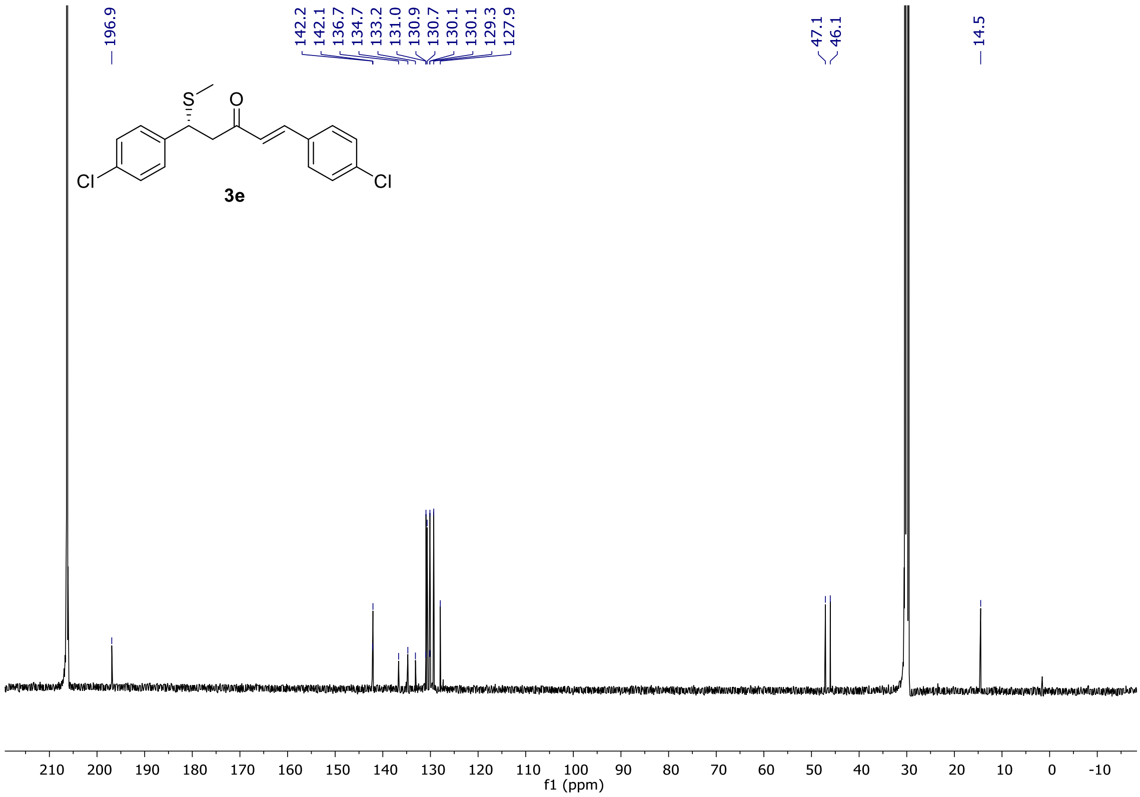
196.9
142.2
142.1
136.7
134.7
133.2
131.0
130.9
130.7
130.1
130.1
129.3
127.9
47.1
46.1
14.5
S
O
Cl
Cl
3e
210 200 190 180 170 160 150 140 130 120 110 100 90 80 70 60 50 40 30 20 10 0 -10
f1 (ppm)

3f:

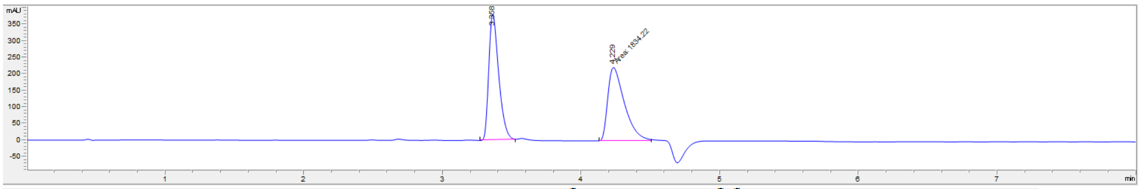

#	Time	Type	Area	Height	Width	Area%	Symmetry
1	3.358	BB	1889.9	381.2	0.0758	50.748	0.619
2	4.229	MM	1834.2	220.8	0.1385	49.252	0.501

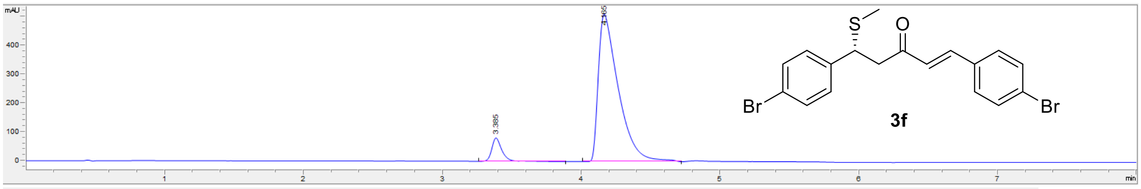

#	Time	Type	Area	Height	Width	Area%	Symmetry
1	3.385	BB	410.9	81.4	0.0749	7.402	0.66
2	4.165	BB	5139.8	512.9	0.1469	92.598	0.383

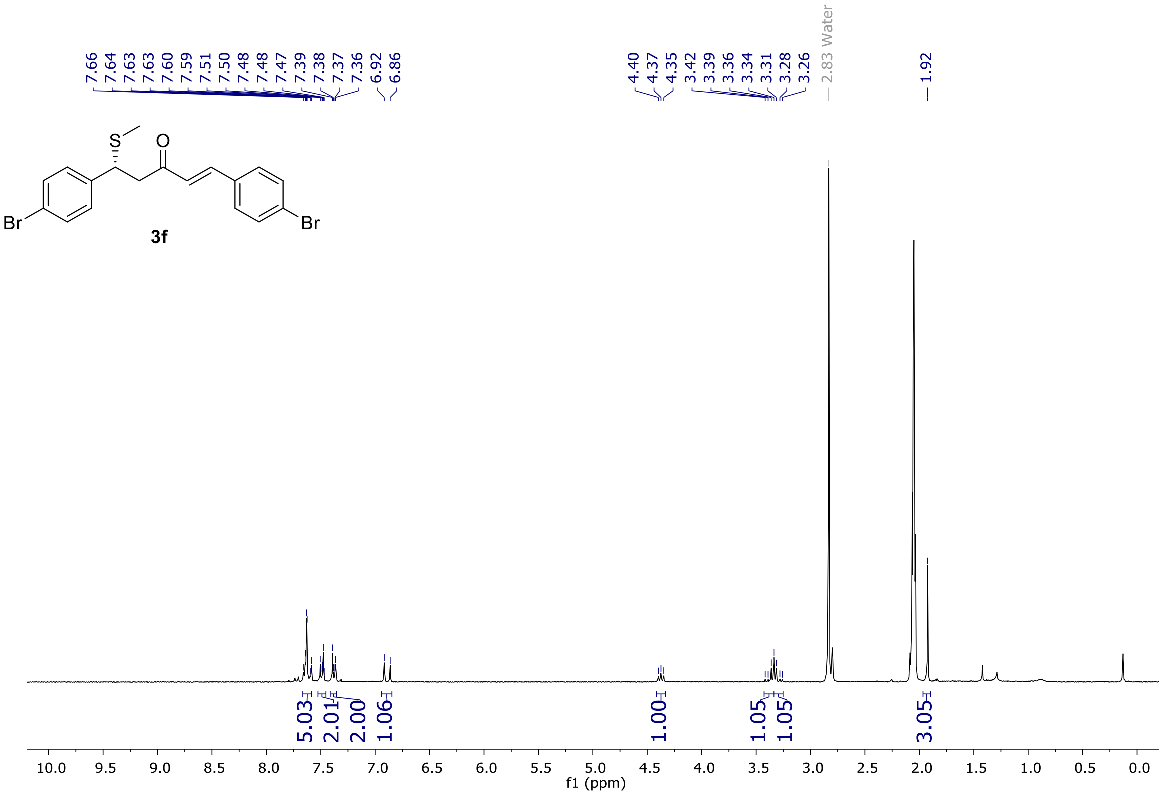

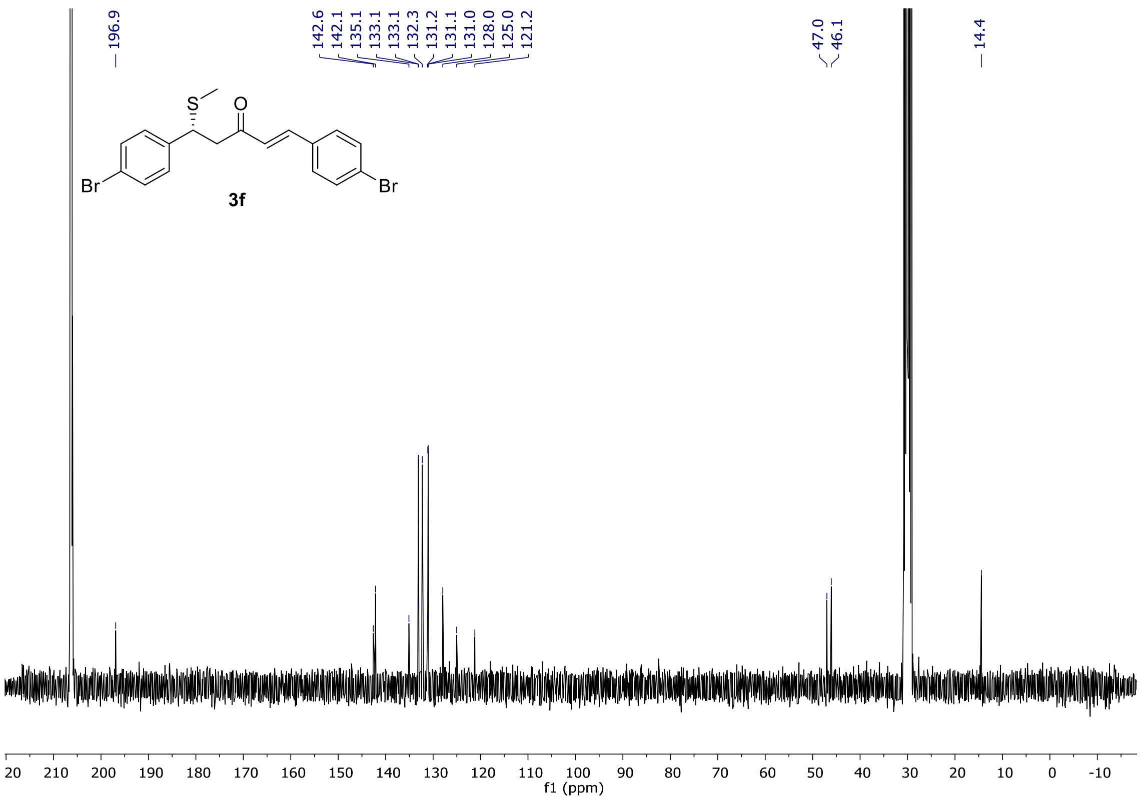
196.9
142.6
142.1
135.1
133.1
133.1
132.3
131.2
131.1
131.0
128.0
125.0
121.2
47.0
46.1
14.4
S
O
Br
Br
3f
20
210
200
190
180
170
160
150
140
130
120
110
100
90
80
70
60
50
40
30
20
10
0
-10
f1 (ppm)

3g:

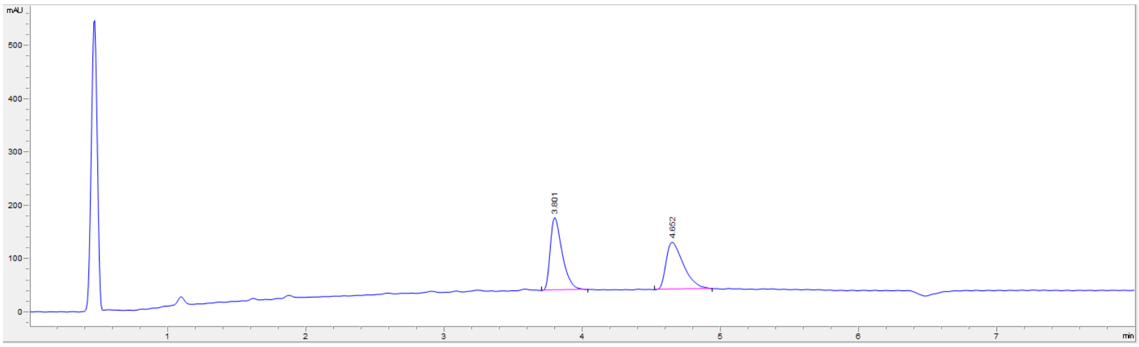

#	Time	Type	Area	Height	Width	Area%	Symmetry
1	3.801	BB	822.5	136.1	0.0905	51.408	0.552
2	4.652	BB	777.5	88.4	0.1346	48.592	0.502

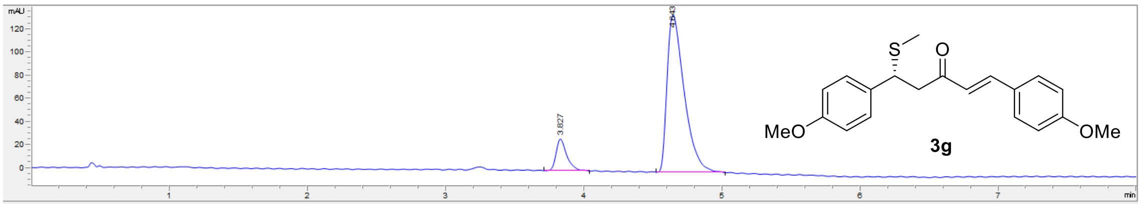

#	Time	Type	Area	Height	Width	Area%	Symmetry
1	3.827	BB	150.4	27.5	0.0818	11.335	0.62
2	4.643	BB	1176.4	136	0.1309	88.665	0.486

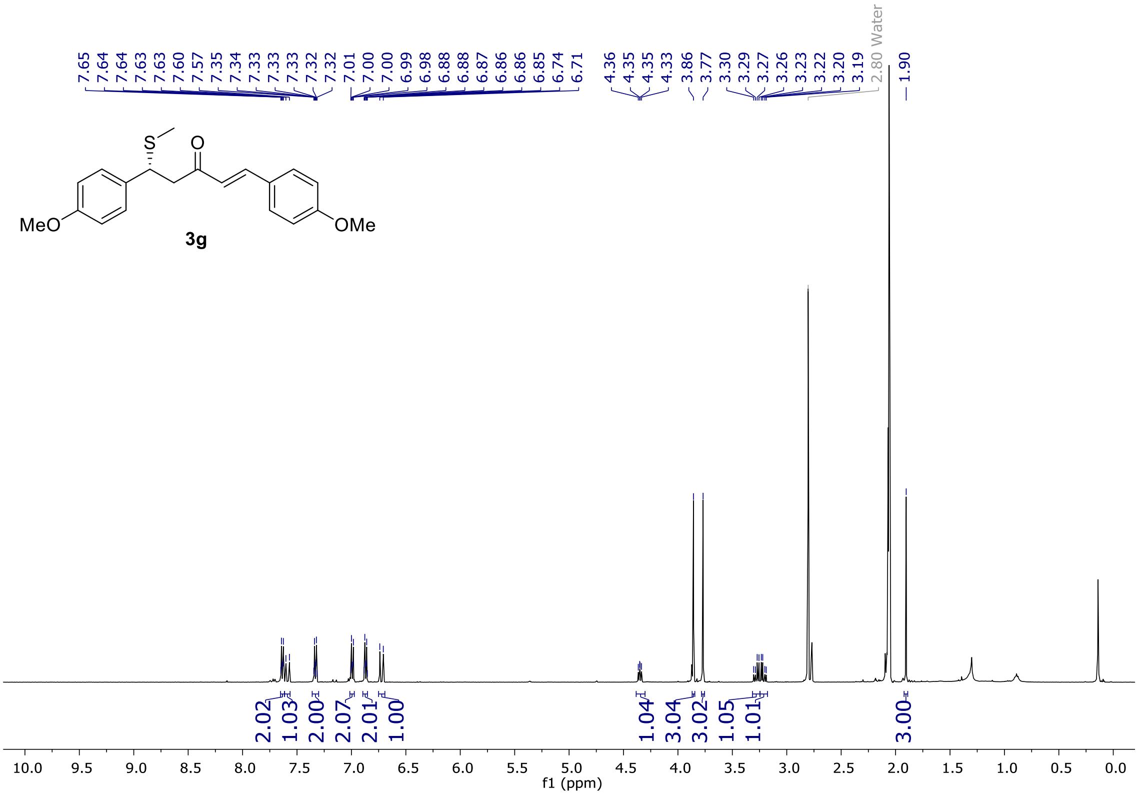

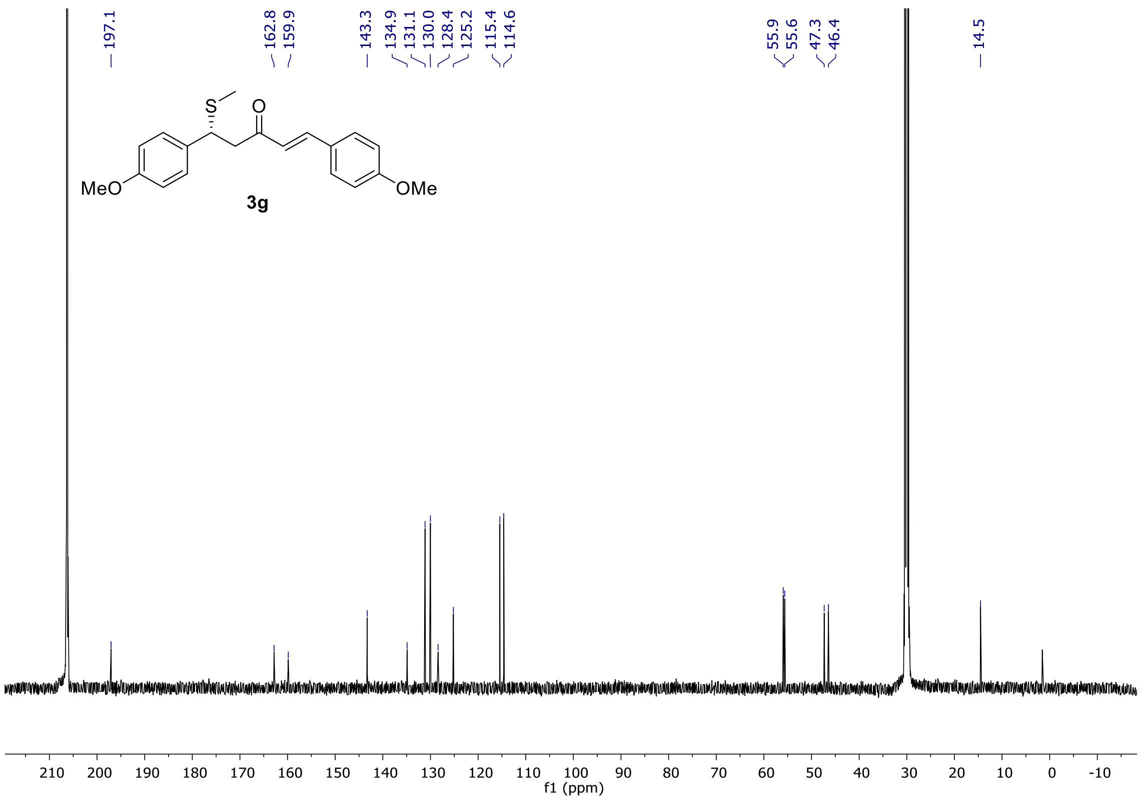

197.1
162.8
159.9
143.3
134.9
131.1
130.0
128.4
125.2
115.4
114.6
55.9
55.6
47.3
46.4
14.5
S
O
MeO
OMe
3g
210 200 190 180 170 160 150 140 130 120 110 100 90 80 70 60 50 40 30 20 10 0 -10
f1 (ppm)

3h:

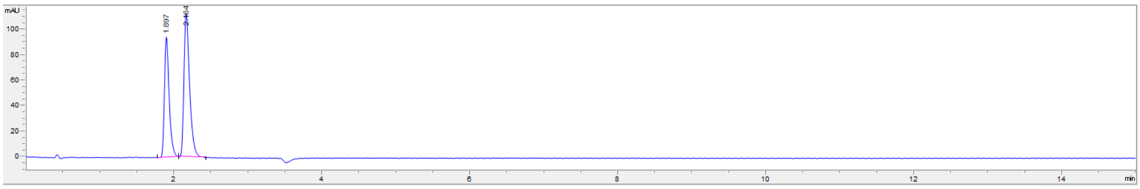

#	Time	Type	Area	Height	Width	Area%	Symmetry
1	1.897	BB	419.1	94.3	0.0658	41.354	0.672
2	2.164	BB	594.4	112.3	0.0797	58.646	0.61

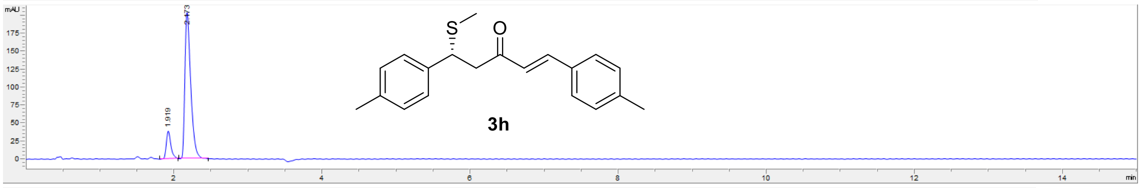

#	Time	Type	Area	Height	Width	Area%	Symmetry
1	1.919	BB	167.3	38.7	0.0664	13.049	0.702
2	2.173	BB	1114.5	203.2	0.082	86.951	0.543

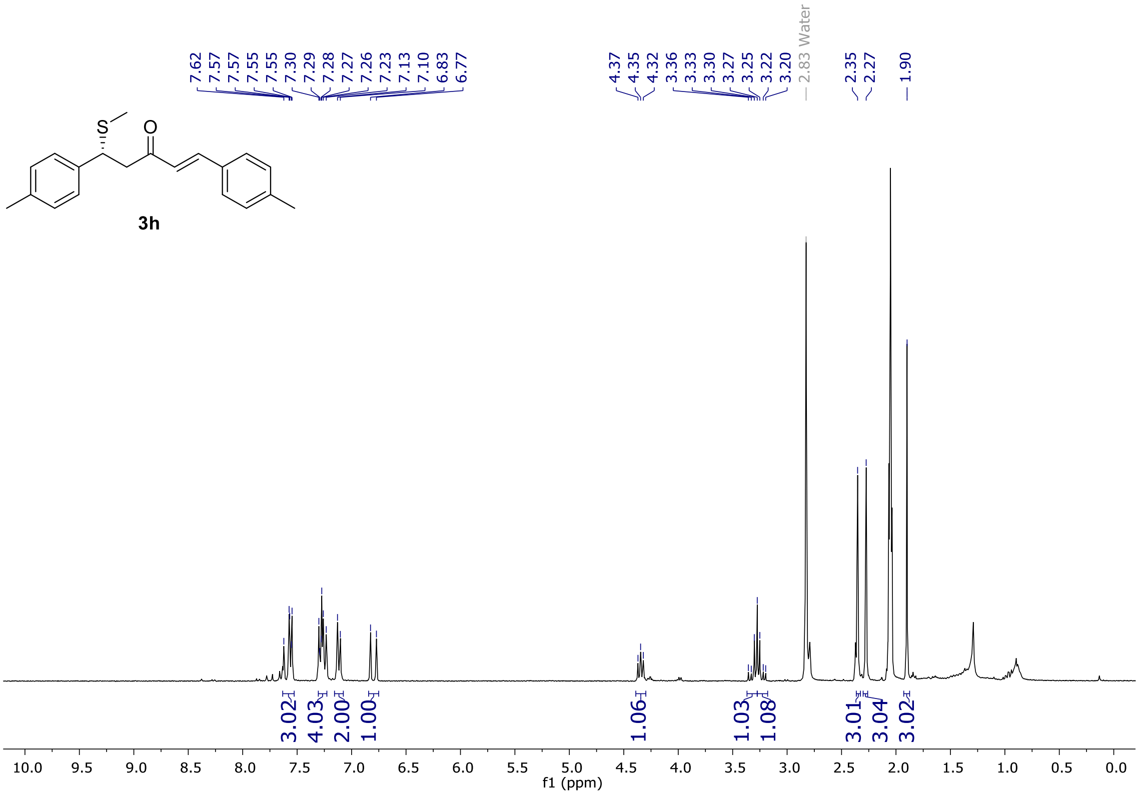

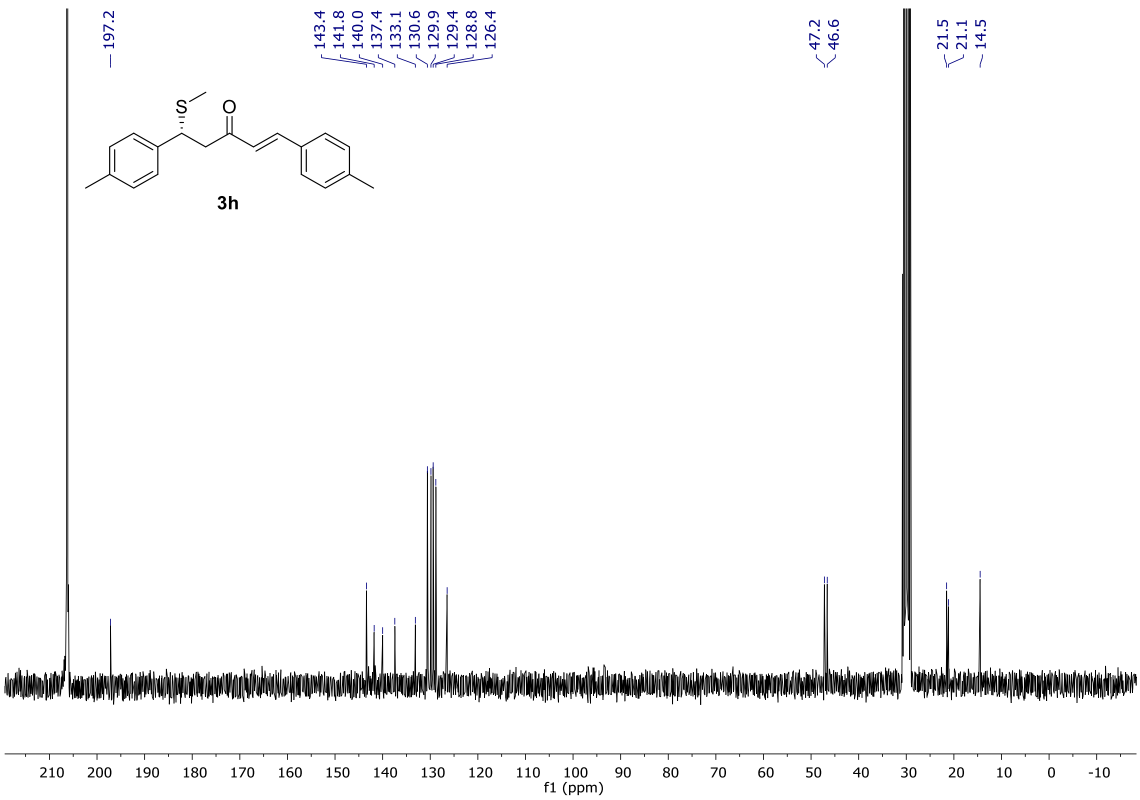
197.2
143.4
141.8
140.0
137.4
133.1
130.6
129.9
129.4
128.8
126.4
47.2
46.6
21.5
21.1
14.5
S
O
3h
210 200 190 180 170 160 150 140 130 120 110 100 90 80 70 60 50 40 30 20 10 0 -10
f1 (ppm)

3i:

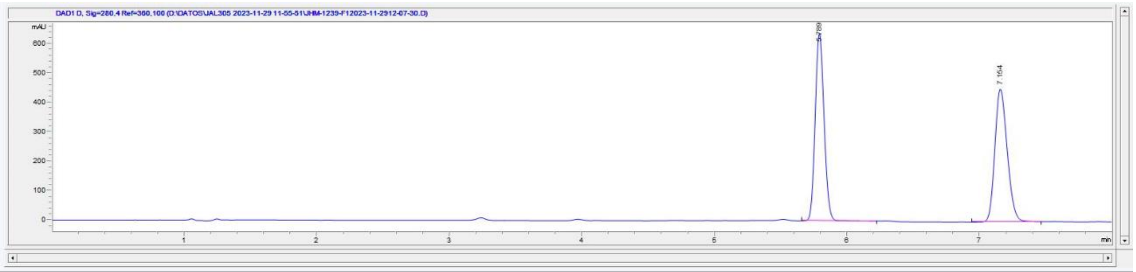

#	Time	Type	Area	Height	Width	Area%	Symmetry
1	5.789	BB	2980.8	641.9	0.0722	50.268	0.848
2	7.154	BB	2949.1	455.2	0.0996	49.732	0.765

#	Time	Type	Area	Height	Width	Area%	Symmetry
1	5.432	MM	416.8	71.4	0.0973	51.717	0.765
2	6.715	MM	389.1	29.5	0.2202	48.283	1.255

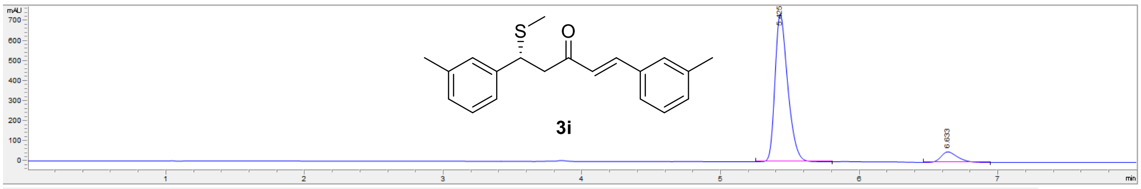

#	Time	Type	Area	Height	Width	Area%	Symmetry
1	5.425	BB	4686.8	736.8	0.0962	91.466	0.655
2	6.633	BB	437.3	52.5	0.1252	8.534	0.666

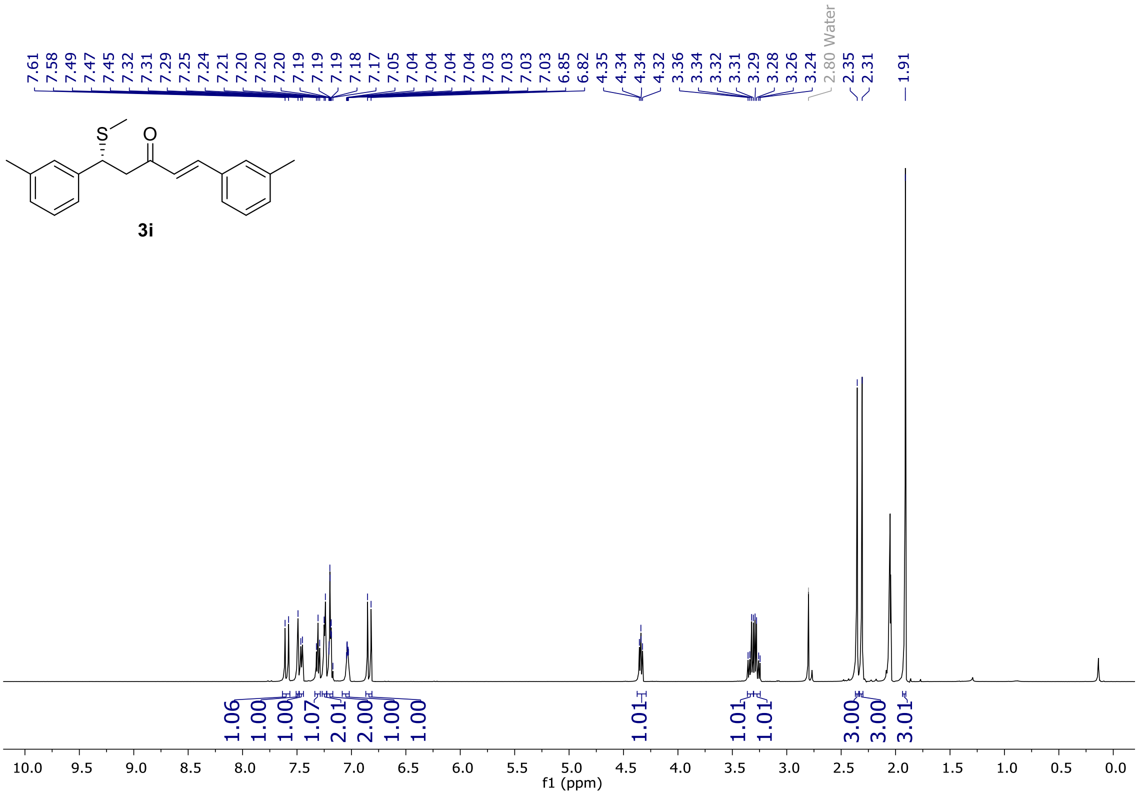

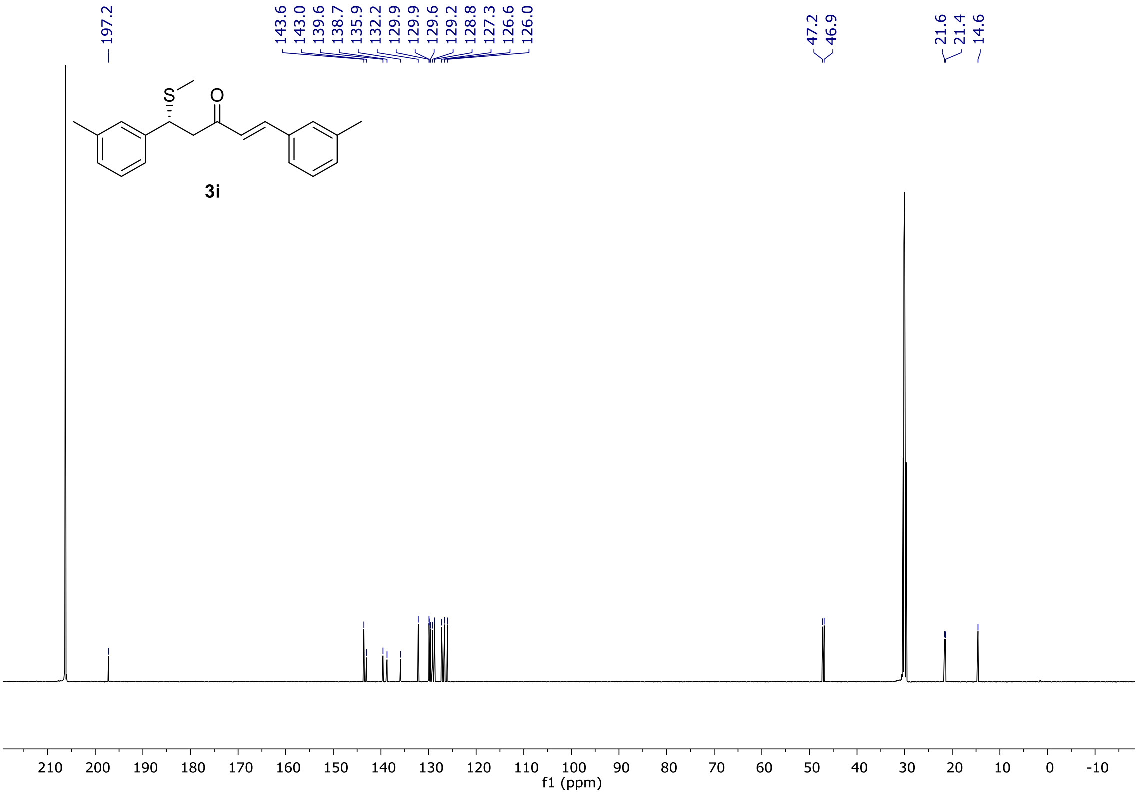
197.2
143.6
143.0
139.6
138.7
135.9
132.2
129.9
129.9
129.6
129.2
128.8
127.3
126.6
126.0
47.2
46.9
21.6
21.4
14.6
S
O
3i
210 200 190 180 170 160 150 140 130 120 110 100 90 80 70 60 50 40 30 20 10 0 -10
f1 (ppm)

3j:

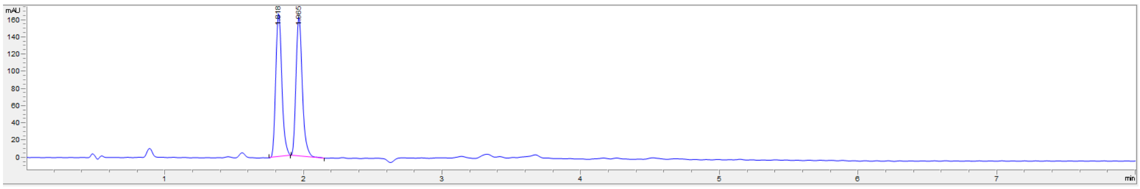

#	Time	Type	Area	Height	Width	Area%	Symmetry
1	1.818	BB	509.3	166.8	0.0476	49.999	0.838
2	1.965	BB	509.4	161.6	0.0508	50.001	0.792

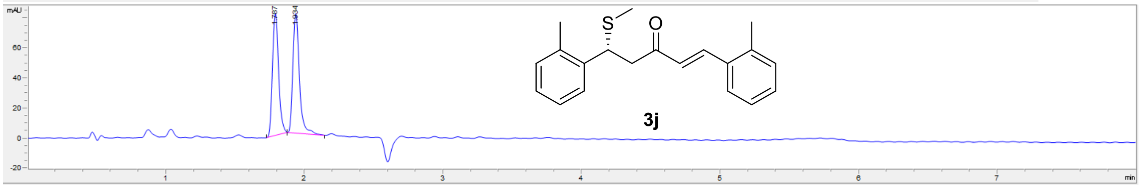

#	Time	Type	Area	Height	Width	Area%	Symmetry
1	1.787	BB	249.1	81.3	0.0498	48.863	0.84
2	1.934	BB	260.7	79.5	0.0522	51.137	0.725

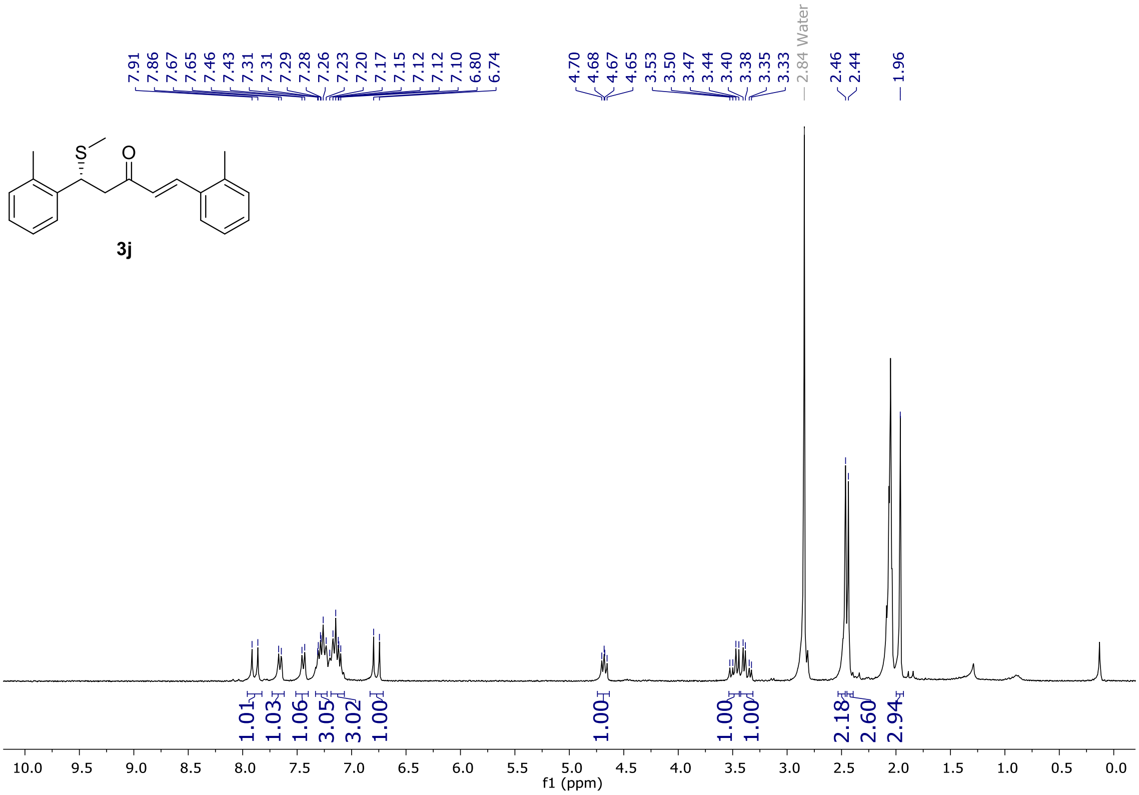

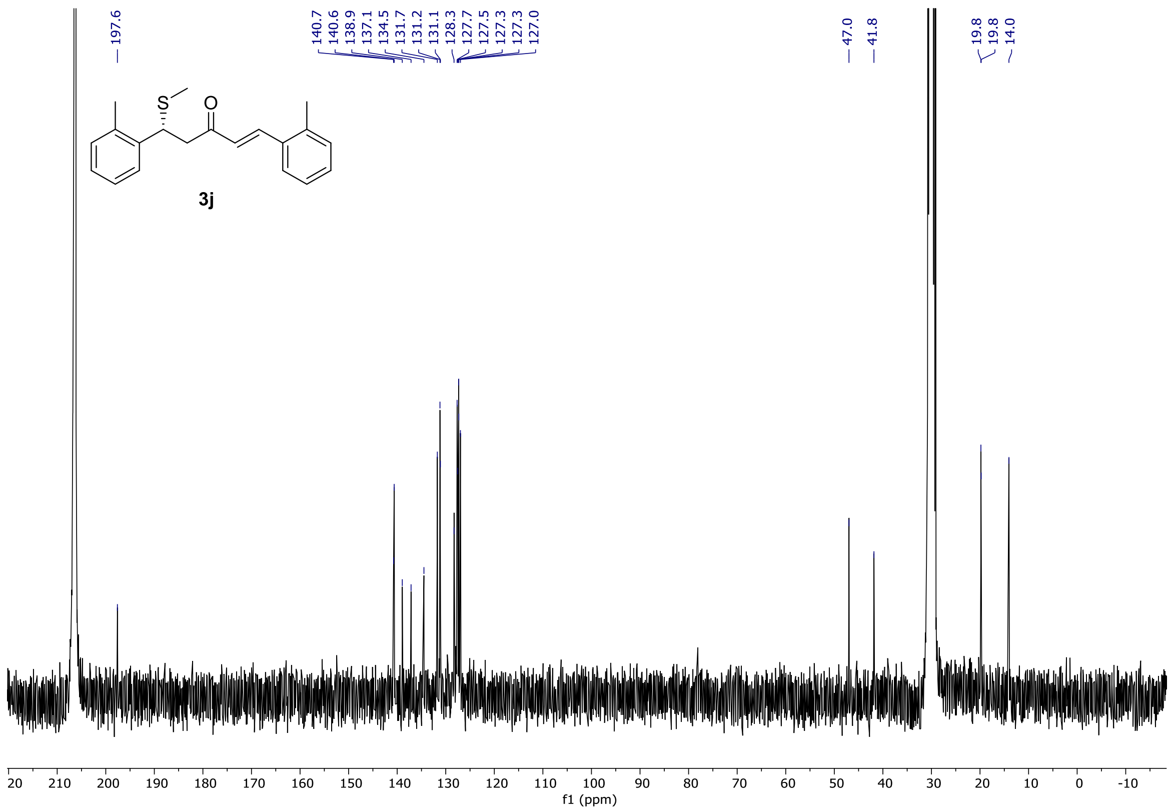

197.6
140.7
140.6
138.9
137.1
134.5
131.7
131.2
131.1
128.3
127.7
127.5
127.3
127.3
127.0
47.0
41.8
19.8
19.8
14.0
S
O
3j
20
210
200
190
180
170
160
150
140
130
120
110
100
90
80
70
60
50
40
30
20
10
0
-10
f1 (ppm)

3k:

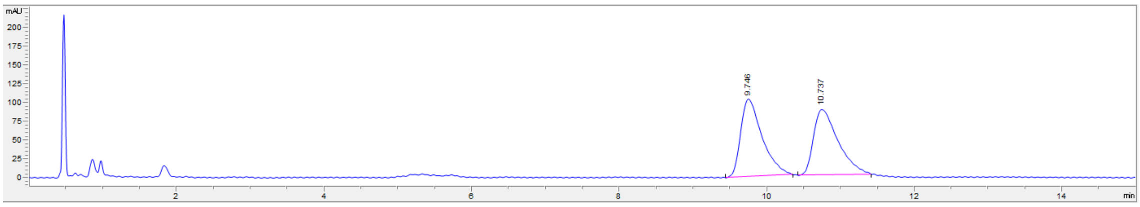

#	Time	Type	Area	Height	Width	Area%	Symmetry
1	9.746	BB	2113.4	103.7	0.2997	50.955	0.538
2	10.737	BB	2034.2	87.3	0.3377	49.045	0.477

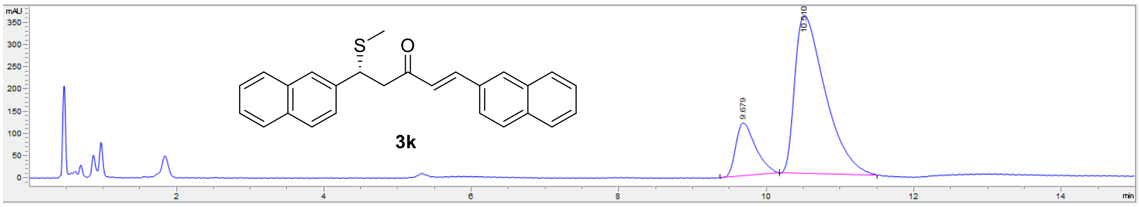

#	Time	Type	Area	Height	Width	Area%	Symmetry
1	9.679	BB	2268.9	119.9	0.2828	18.097	0.561
2	10.51	BB	10269	356.9	0.4177	81.903	0.431

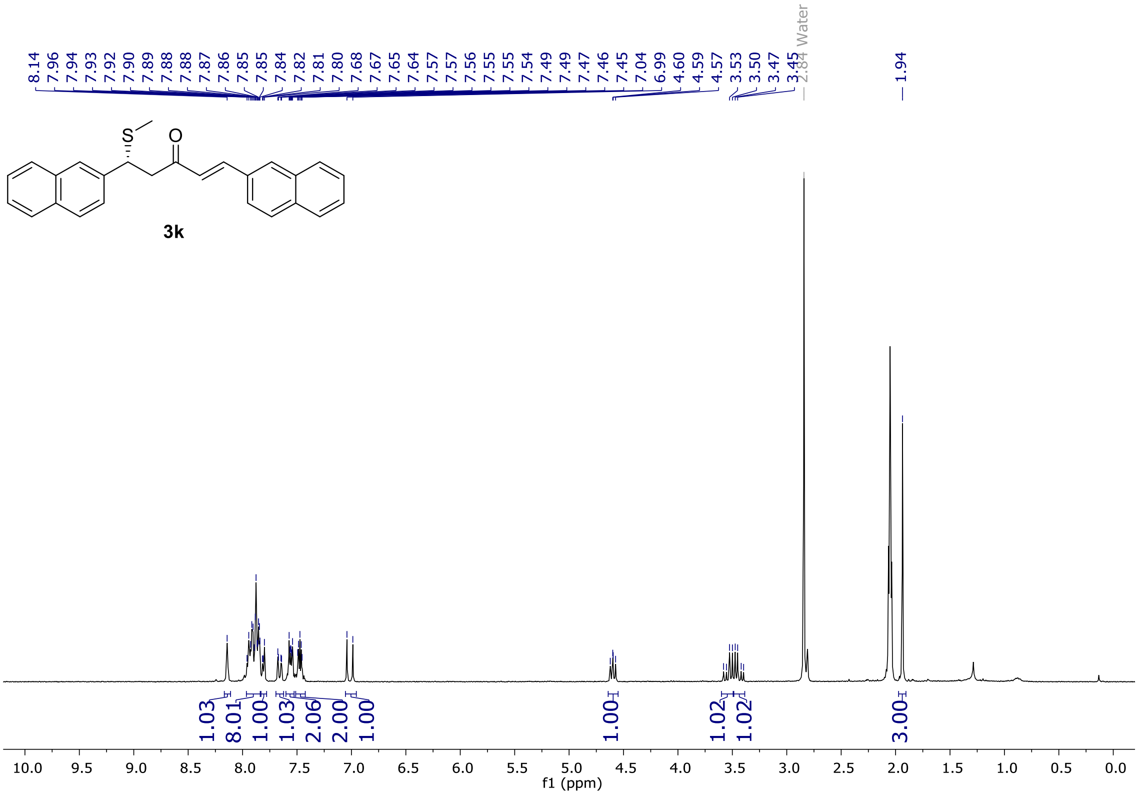

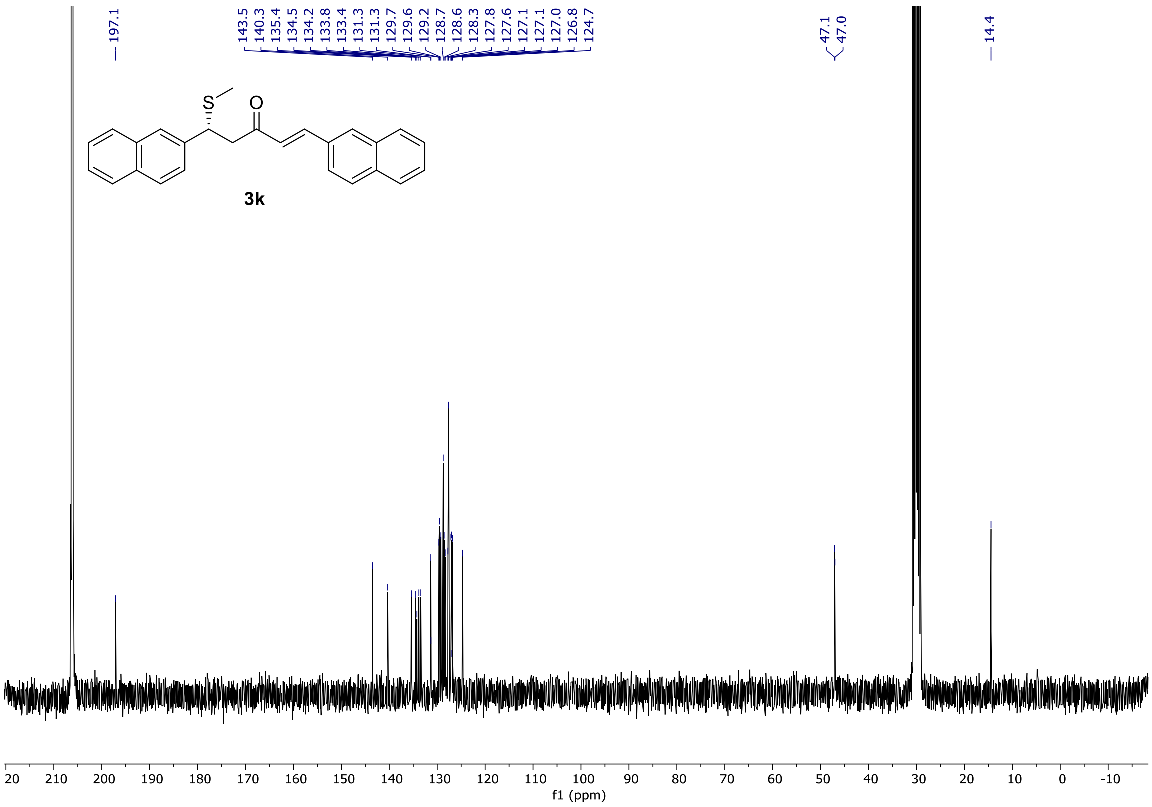

197.1
143.5 140.3 135.4 134.5 134.2 133.8 133.4 131.3 131.3 129.7 129.6 129.2 128.7 128.6 128.3 127.8 127.6 127.1 127.1 127.0 126.8 124.7
47.1 47.0
14.4
S
O
3k
20 210 200 190 180 170 160 150 140 130 120 110 100 90 80 70 60 50 40 30 20 10 0 -10
f1 (ppm)

3l:

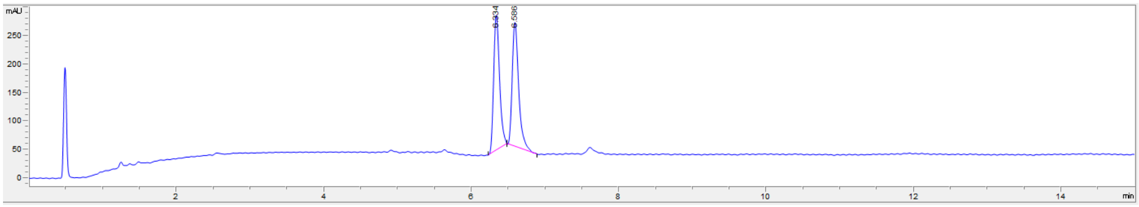

#	Time	Type	Area	Height	Width	Area%	Symmetry
1	6.334	BB	1325	238.3	0.0849	49.878	0.743
2	6.586	BB	1331.5	218.3	0.0931	50.122	0.669

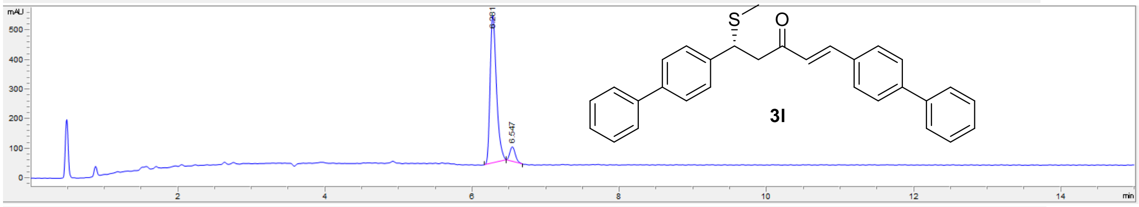

#	Time	Type	Area	Height	Width	Area%	Symmetry
1	6.281	BB	2975.5	498.2	0.0917	92.237	0.663
2	6.547	BB	250.4	48.9	0.0817	7.763	0.778

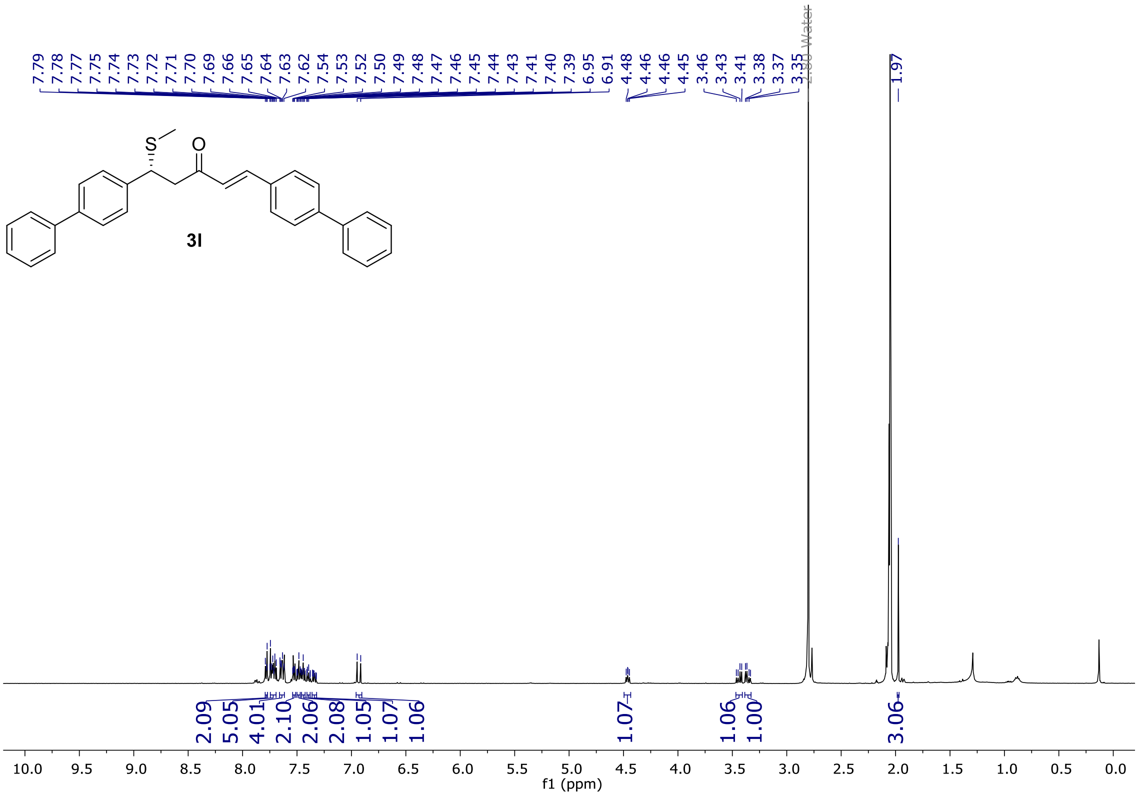

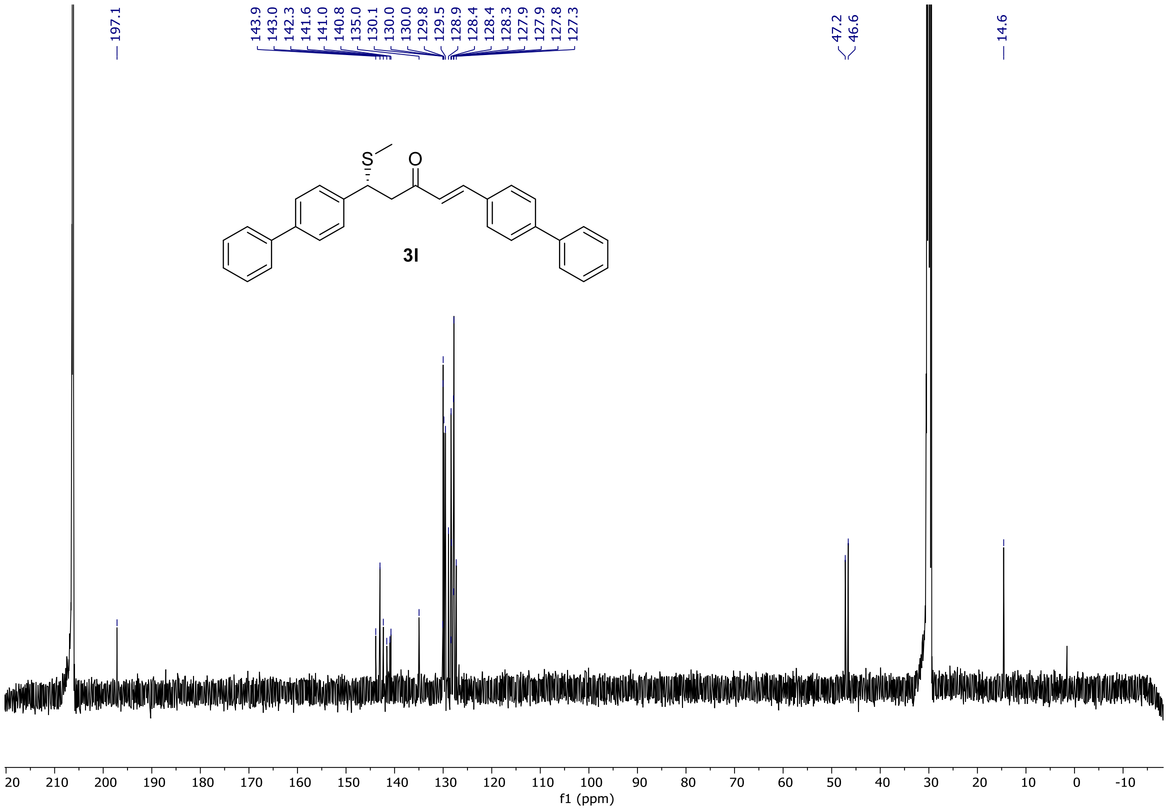
197.1
143.9
143.0
142.3
141.6
141.0
140.8
135.0
130.1
130.0
130.0
129.8
129.5
128.9
128.4
128.4
128.3
127.9
127.9
127.8
127.3
47.2
46.6
14.6
S
O
3l
f1 (ppm)

5a:

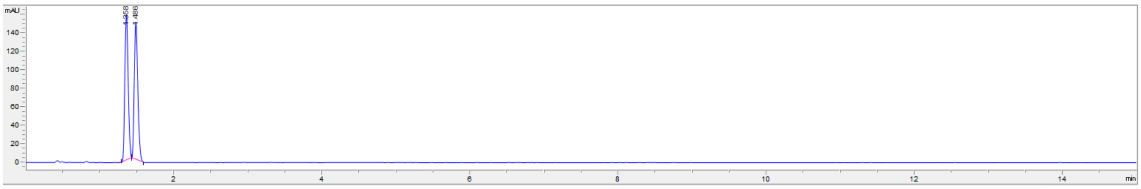

#	Time	Type	Area	Height	Width	Area%	Symmetry
1	1.358	BB	493.4	158.3	0.0484	49.995	0.872
2	1.486	BB	493.5	149.1	0.0526	50.005	0.783

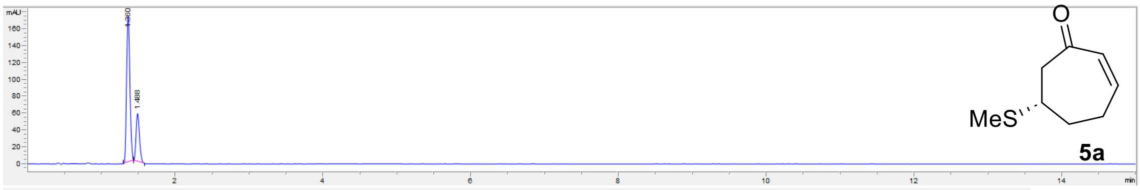

#	Time	Type	Area	Height	Width	Area%	Symmetry
1	1.36	BB	601.7	192	0.0486	74.687	0.858
2	1.488	BB	203.9	63.5	0.0515	25.313	0.778

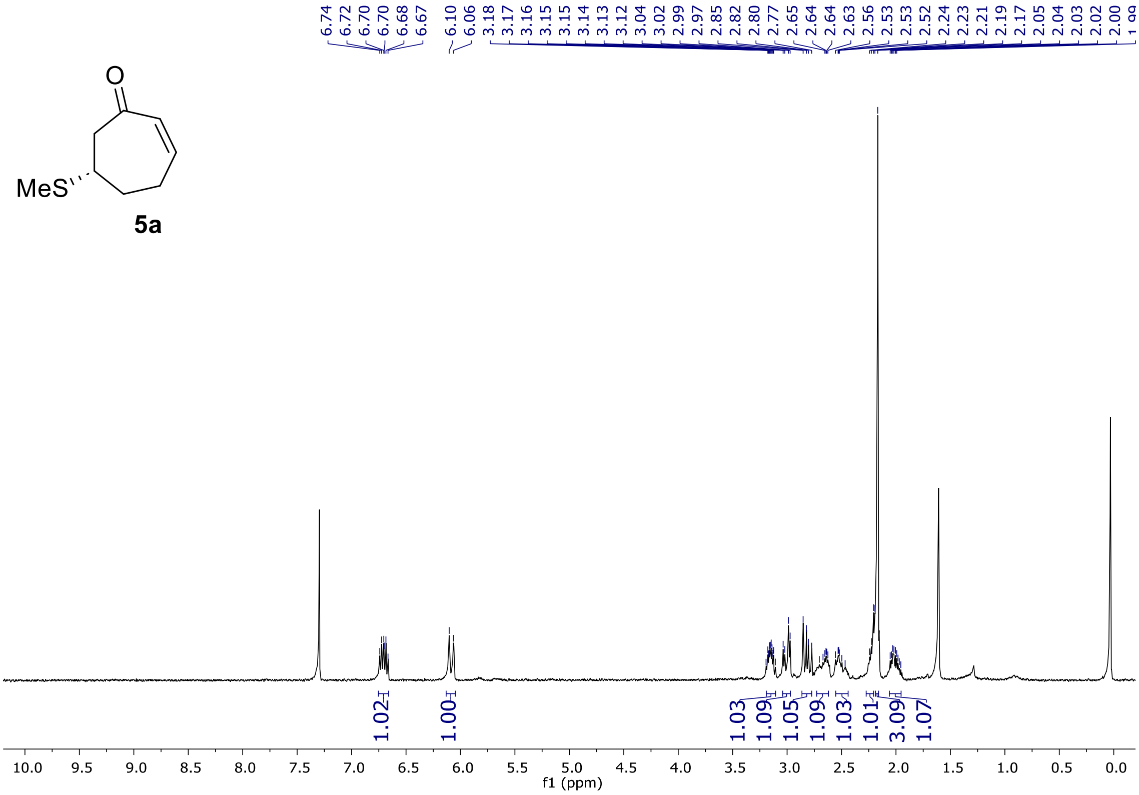

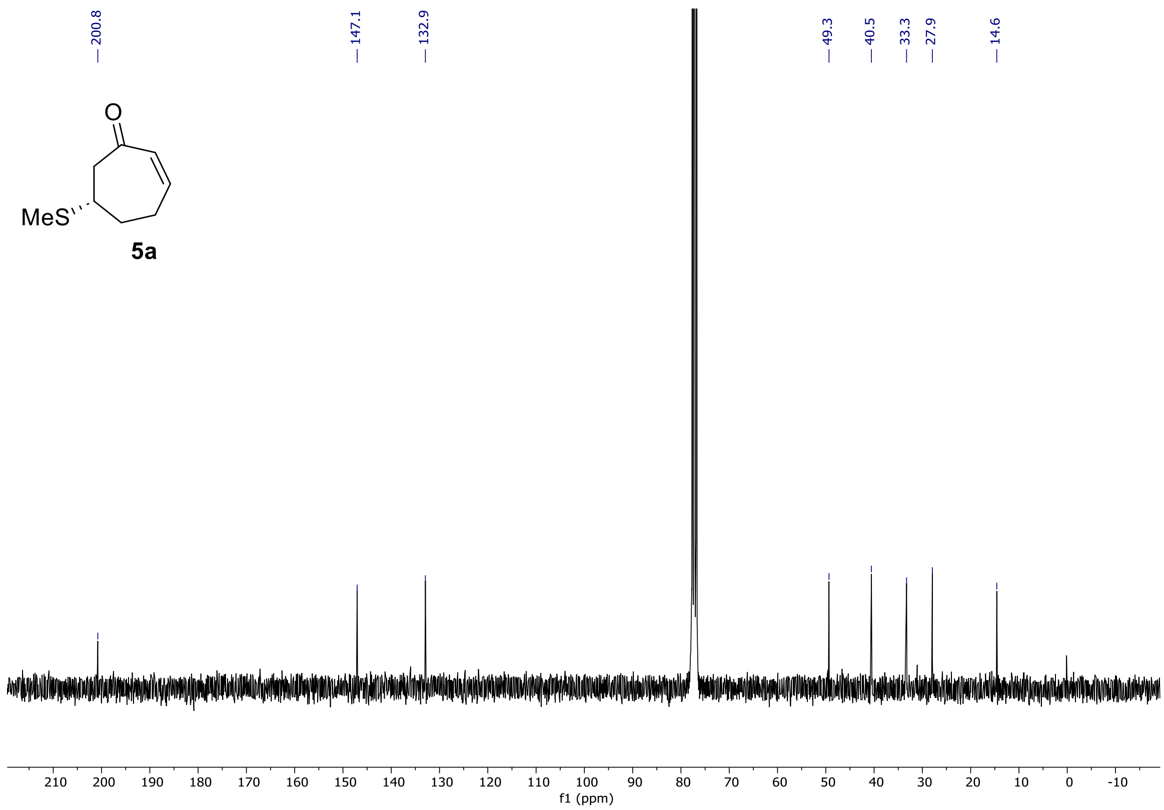

200.8
147.1
132.9
49.3
40.5
33.3
27.9
14.6
MeS
5a
210 200 190 180 170 160 150 140 130 120 110 100 90 80 70 60 50 40 30 20 10 0 -10
f1 (ppm)

5b:

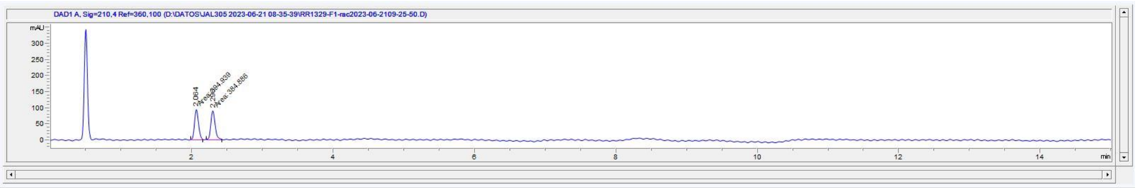

#	Time	Type	Area	Height	Width	Area%	Symmetry
1	2.064	MM	394.9	96.2	0.0684	50.645	0.824
2	2.296	MM	384.9	89.9	0.0714	49.355	0.801

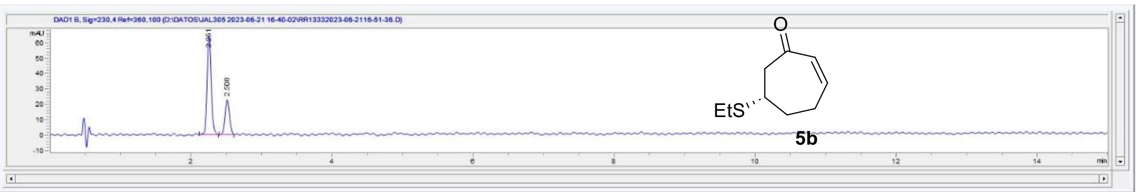

#	Time	Type	Area	Height	Width	Area%	Symmetry
1	2.251	BB	266.2	65.1	0.0637	72.323	0.863
2	2.508	BB	101.9	22.9	0.0698	27.677	0.904

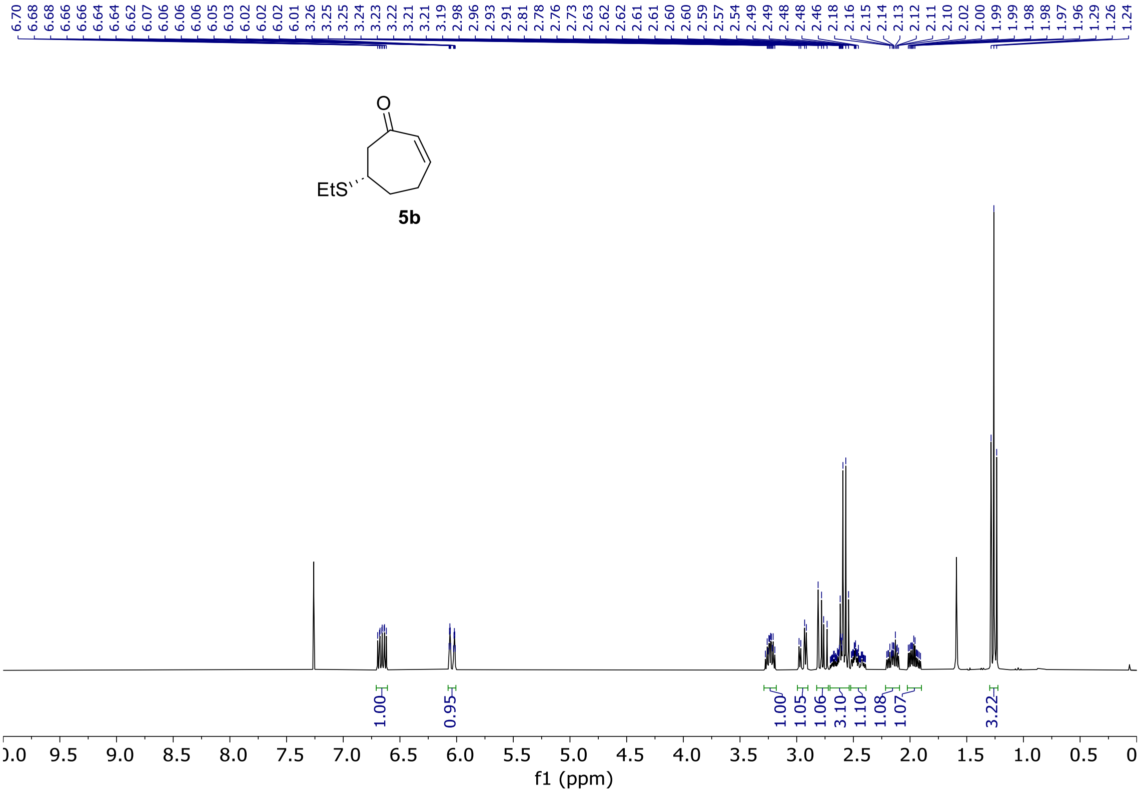

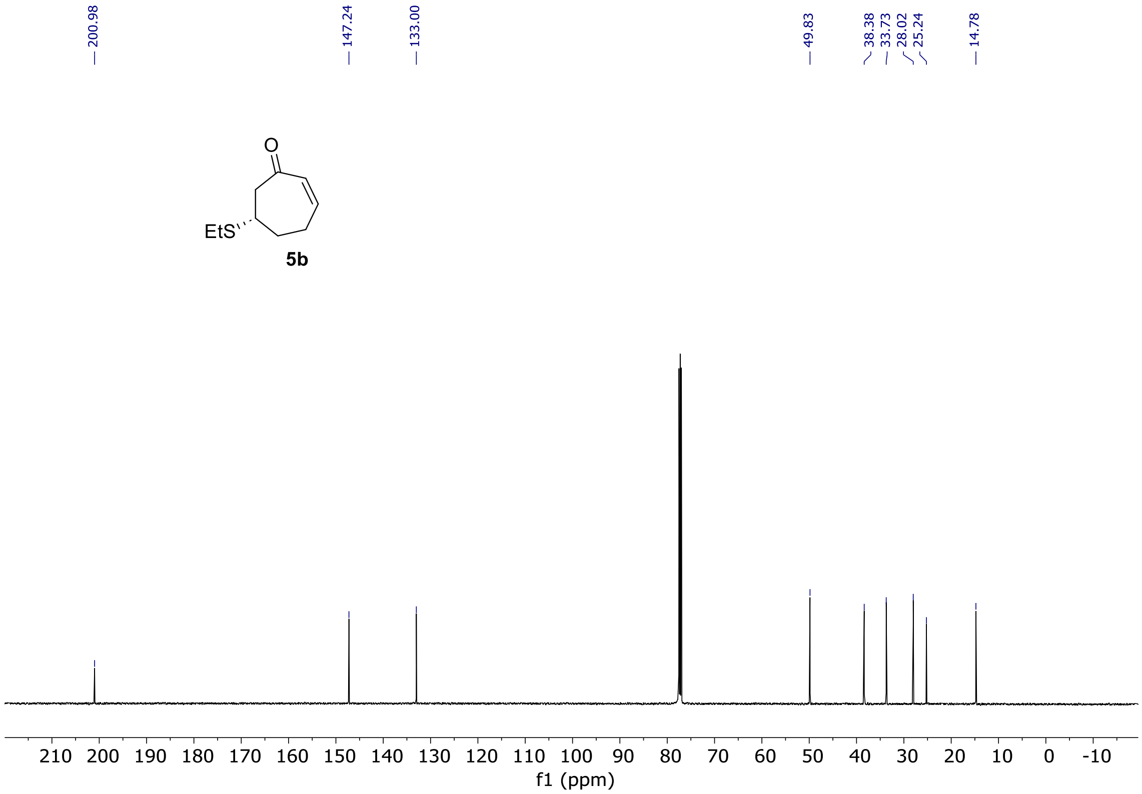
200.98
147.24
133.00
49.83
38.38
33.73
28.02
25.24
14.78
O
EtS
5b
210 200 190 180 170 160 150 140 130 120 110 100 90 80 70 60 50 40 30 20 10 0 -10
f1 (ppm)

13.4. Derivatizations: NMR and chromatograms

6:

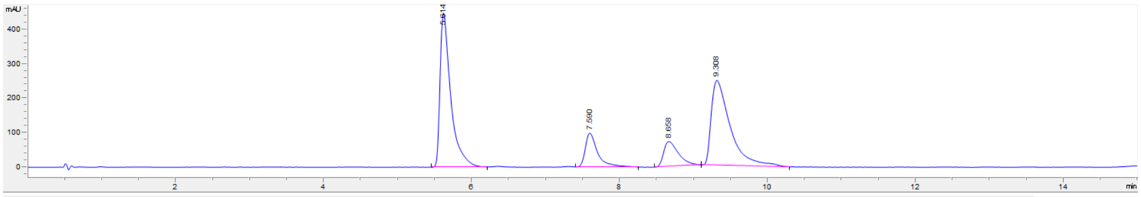

#	Time	Type	Area	Height	Width	Area%	Symmetry
1	5.614	BB	4491.8	447.3	0.1472	40.763	0.43
2	7.59	BB	1142	98.1	0.1738	10.364	0.507
3	8.658	BB	1005.3	72.4	0.208	9.123	0.504
4	9.308	BB	4380.2	246.1	0.2657	39.750	0.387

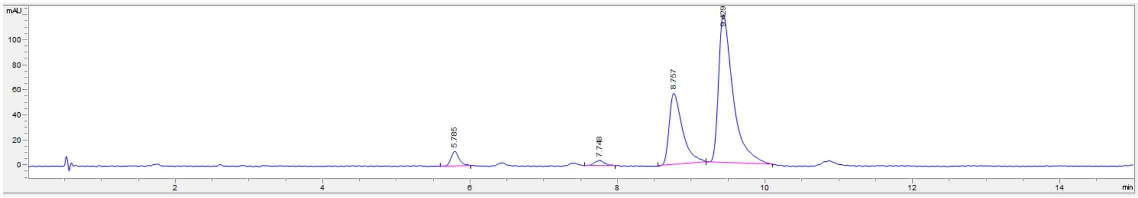

#	Time	Type	Area	Height	Width	Area%	Symmetry
1	5.785	BB	90.6	11.7	0.1145	3.663	0.796
2	7.748	BB	39.7	4.2	0.1266	1.607	0.957
3	8.757	BB	719.9	57.2	0.1829	29.119	0.502
4	9.429	BB	1622.1	118.2	0.2022	65.611	0.456

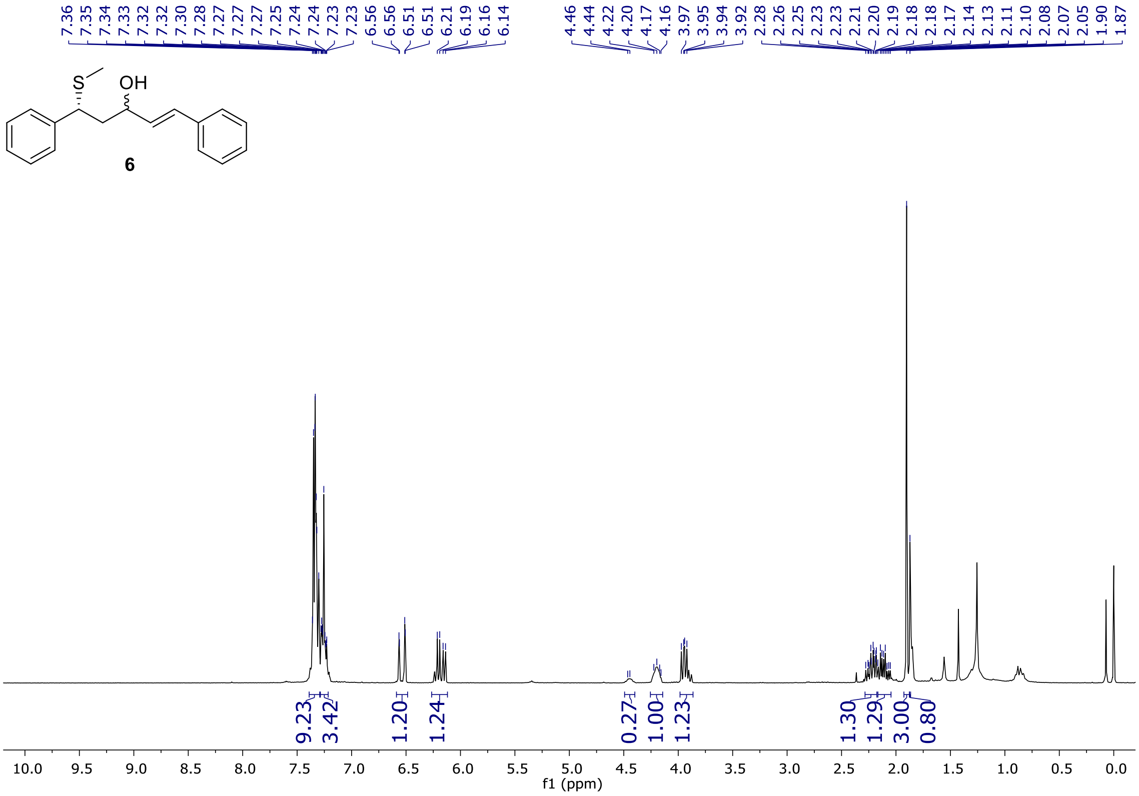

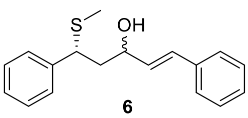

6

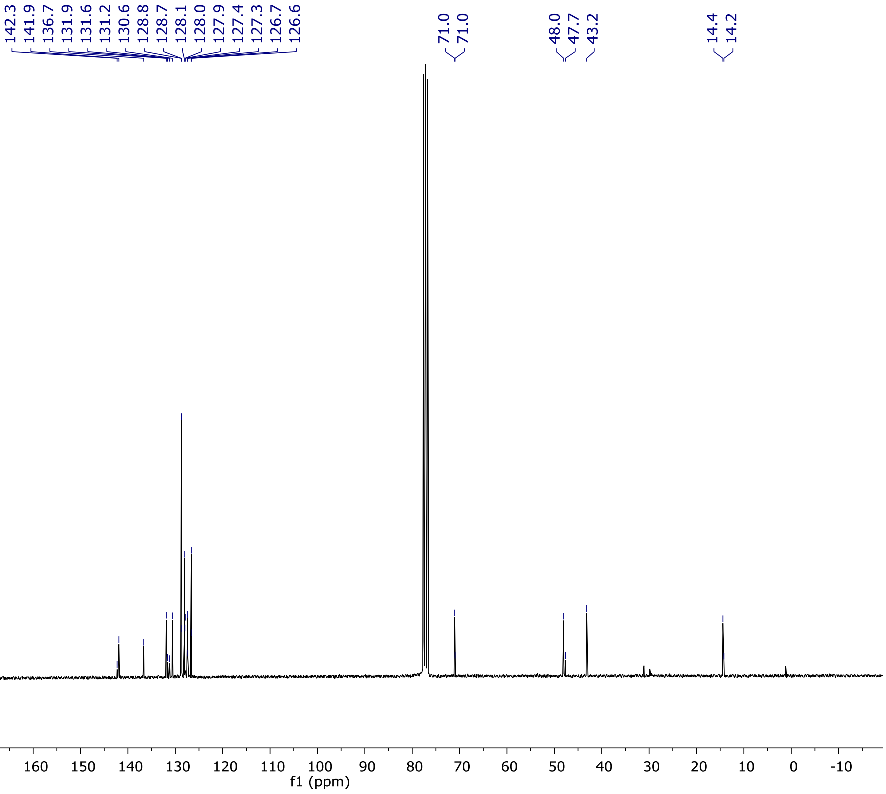
142.3
141.9
136.7
131.9
131.6
131.2
130.6
128.8
128.7
128.1
128.0
127.9
127.4
127.3
126.7
126.6
71.0
71.0
48.0
47.7
43.2
14.4
14.2
210 200 190 180 170 160 150 140 130 120 110 100 90 80 70 60 50 40 30 20 10 0 -10
f1 (ppm)

7:

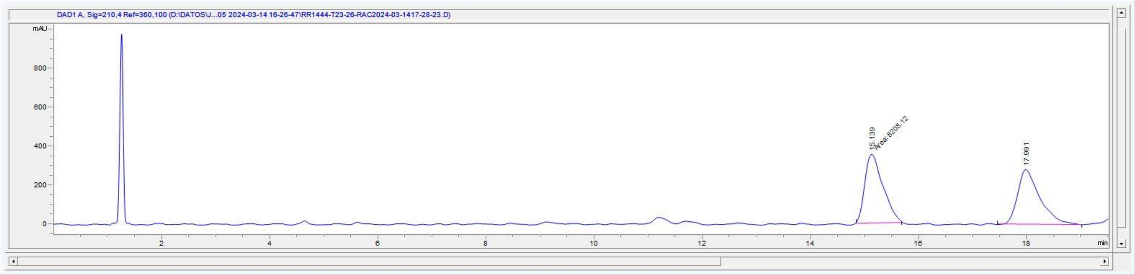

#	Time	Type	Area	Height	Width	Area%	Symmetry
1	15.139	MM	8208.1	351.5	0.3892	50.157	0.592
2	17.991	BB	8156.7	282.7	0.4007	49.843	0.561

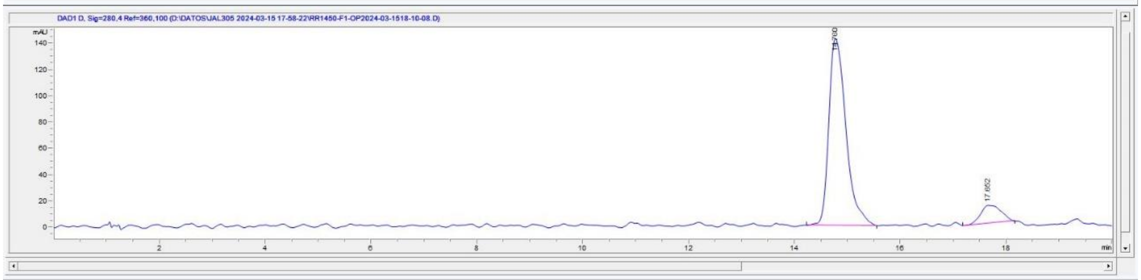

#	Time	Type	Area	Height	Width	Area%	Symmetry
1	14.76	BB	3219.4	142.8	0.351	89.498	0.593
2	17.652	BB	377.8	13.8	0.3563	10.502	0.619

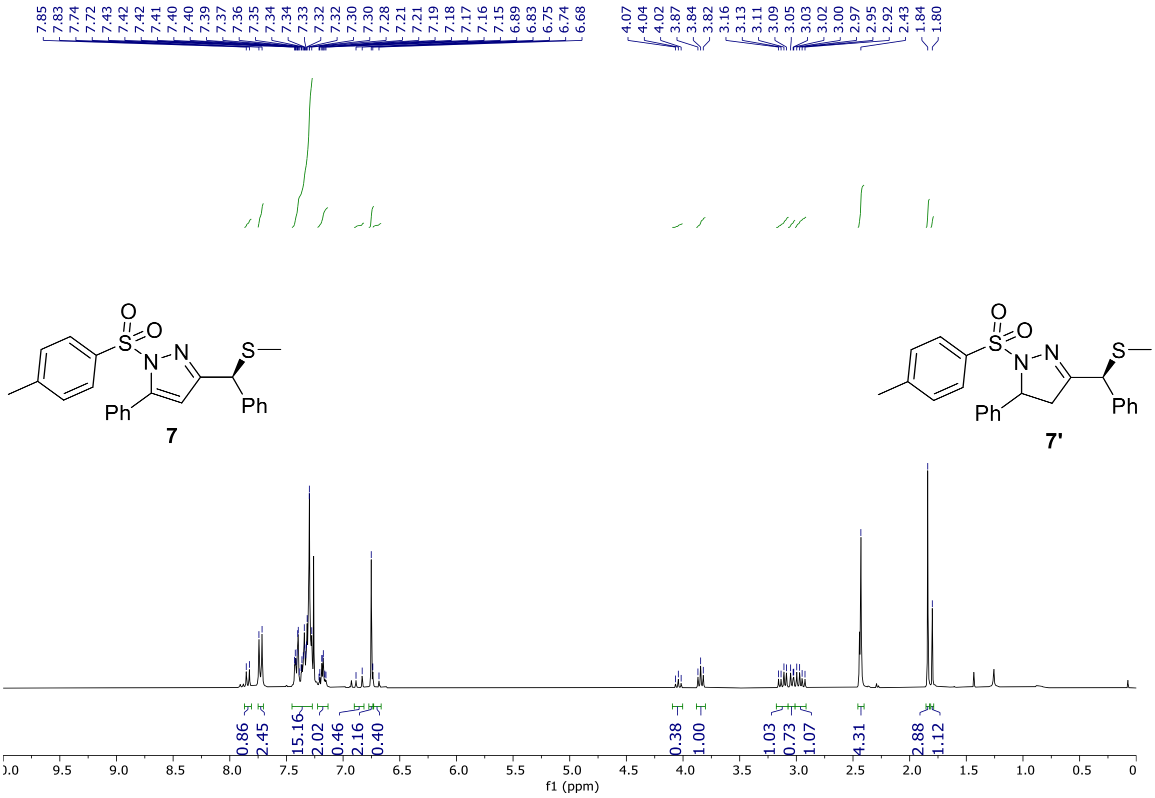

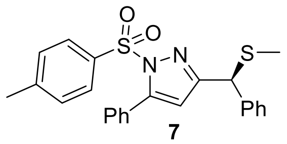

7

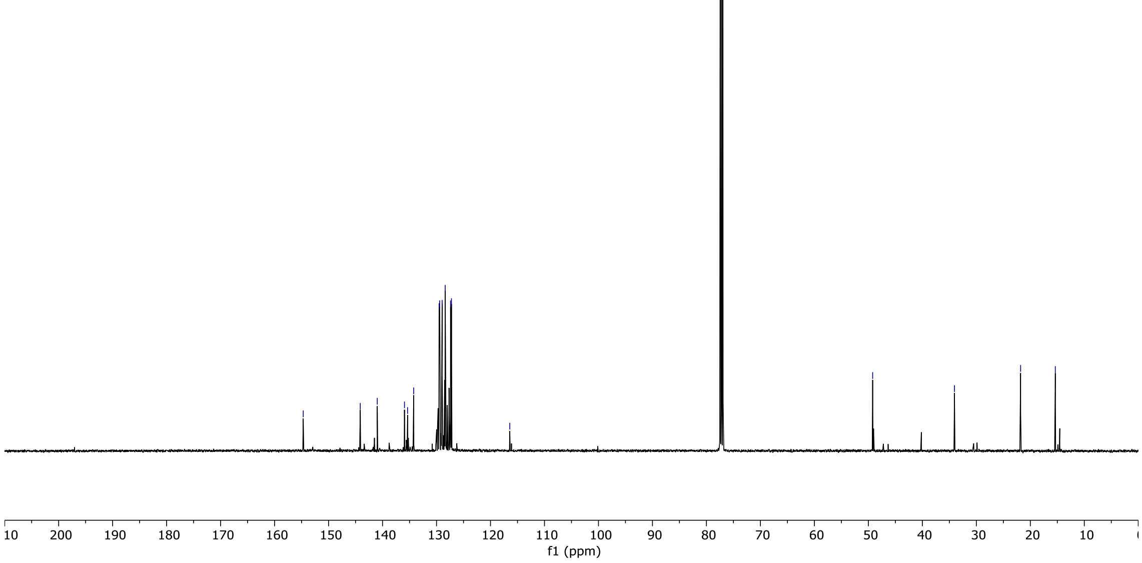
154.69
144.13
140.98
135.94
135.36
134.23
129.52
129.41
128.96
128.40
127.40
127.23
116.42
49.21
34.05
21.80
15.37
f1 (ppm)

8:

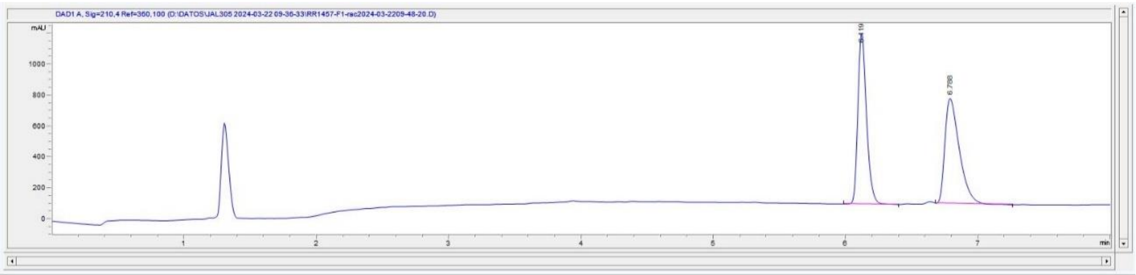

#	Time	Type	Area	Height	Width	Area%	Symmetry
1	6.119	BB	5020.2	1108.9	0.0688	49.421	0.718
2	6.788	BB	5137.8	678.6	0.1143	50.579	0.522

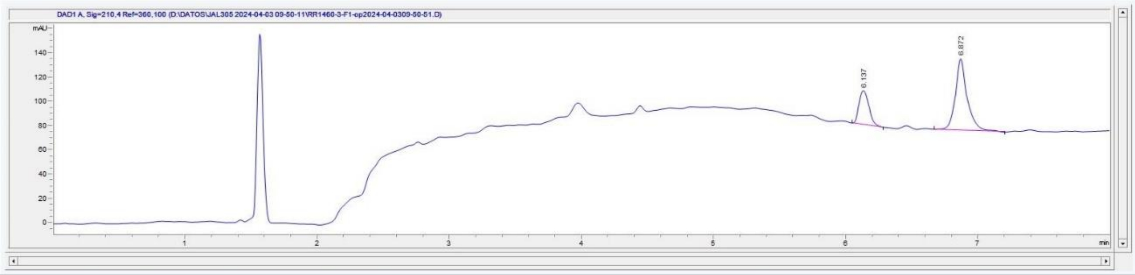

#	Time	Type	Area	Height	Width	Area%	Symmetry
1	6.137	MM	63.9	25.1	0.0423	16.208	0.942
2	6.872	BB	330.1	43.5	0.1145	83.792	0.72

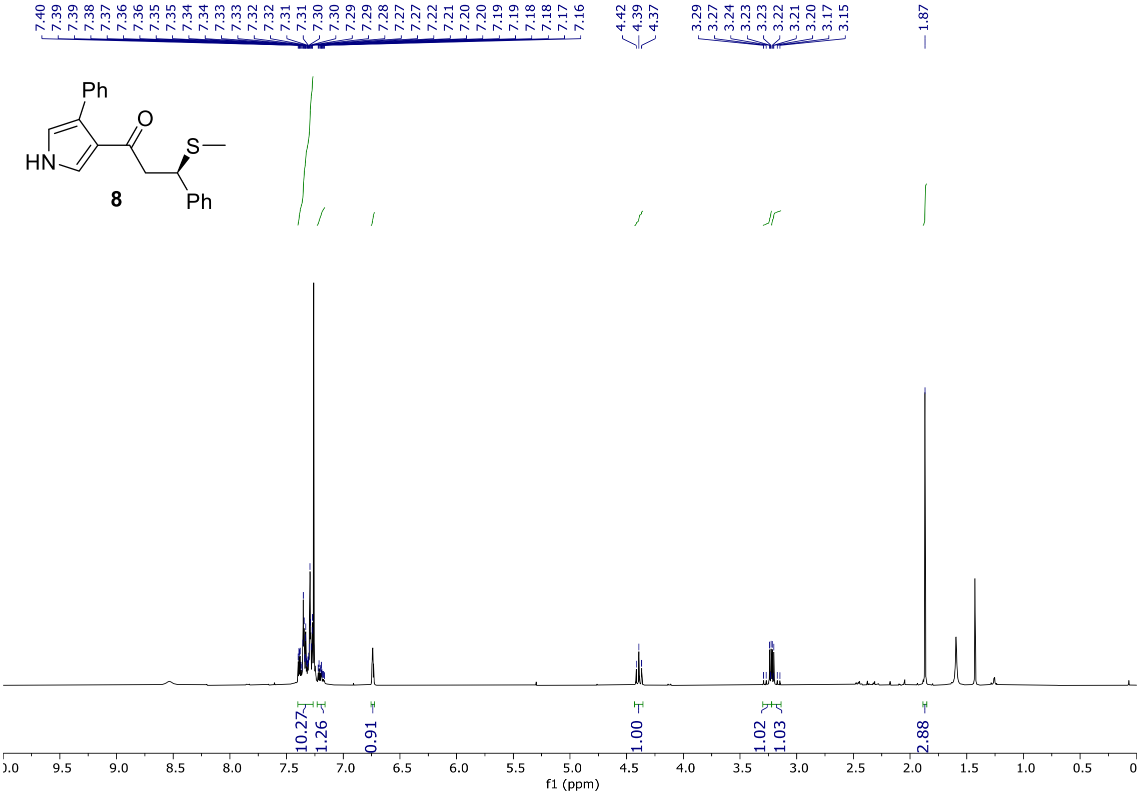

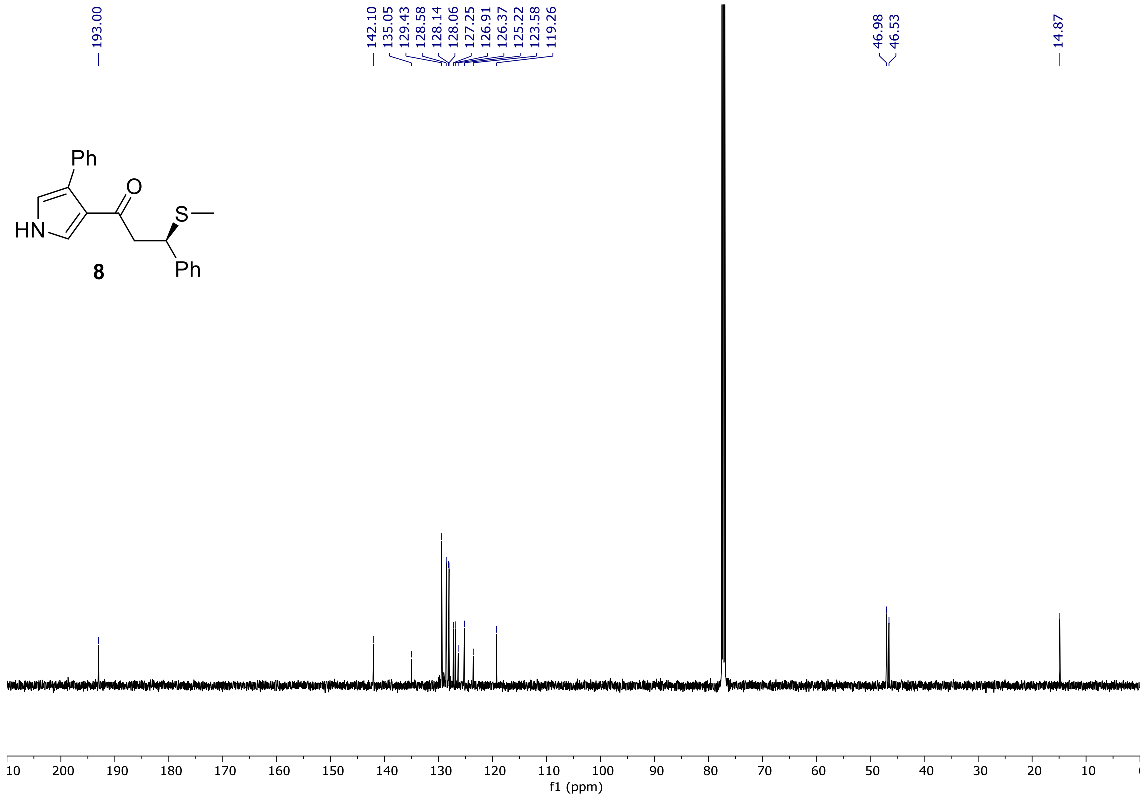
193.00
142.10
135.05
129.43
128.58
128.14
128.06
127.25
126.91
126.37
125.22
123.58
119.26
46.98
46.53
14.87
Ph
O
S
HN
Ph
8
210 200 190 180 170 160 150 140 130 120 110 100 90 80 70 60 50 40 30 20 10 0
f1 (ppm)

9:

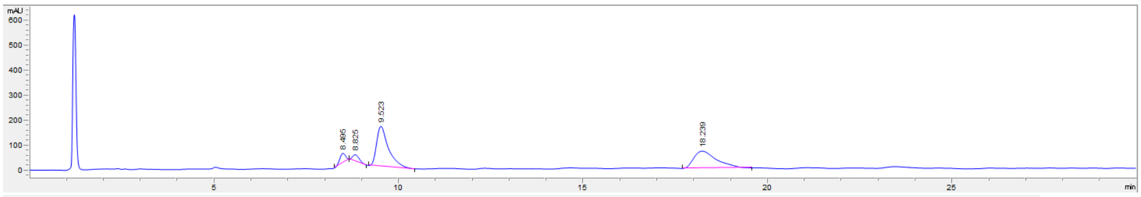

#	Time	Type	Area	Height	Width	Area%	Symmetry
1	8.495	BB	430.6	38.3	0.1791	5.885	1.3
2	8.825	BB	337.4	26.5	0.2046	4.611	0.63
3	9.523	BB	3594.8	159.8	0.3324	49.127	0.525
4	18.239	BB	2954.6	68.1	0.5859	40.378	0.54

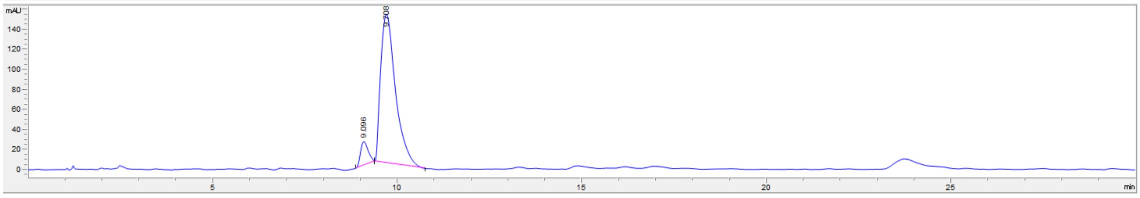

#	Time	Type	Area	Height	Width	Area%	Symmetry
1	9.096	BB	339.1	23.6	0.2174	7.553	0.751
2	9.708	BB	4150.6	148.1	0.4429	92.447	0.583

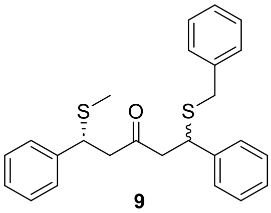

9

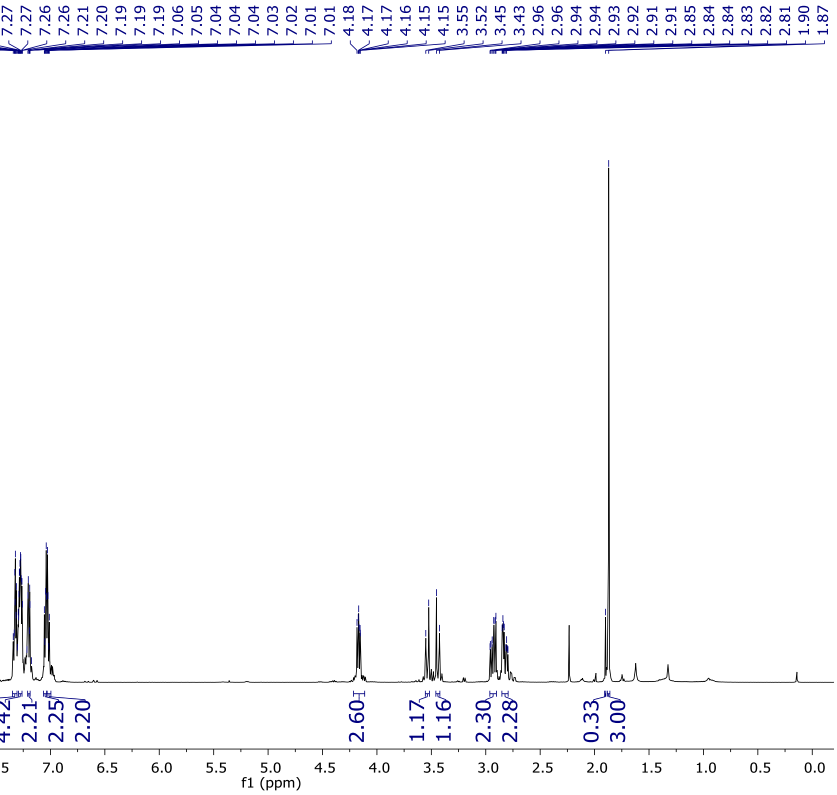

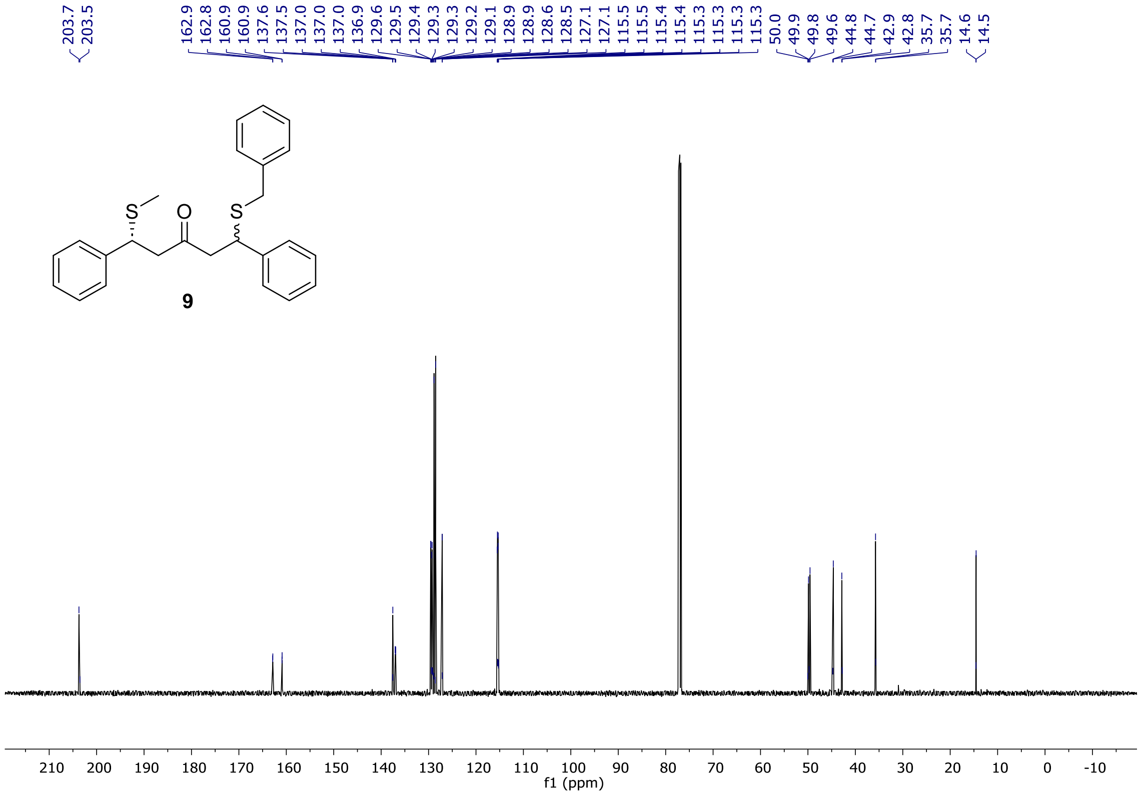

14. Coordinates

Anion-binding mechanism

(I)

N	3.32081000	-3.02076800	0.59963300
C	3.56402600	-4.47789800	0.55475700
H	3.23564200	-4.88958800	1.51410700
H	2.92378900	-4.90548200	-0.22200100
C	5.06497500	-4.76702000	0.29695500
H	5.22963300	-5.07369400	-0.74152400
H	5.41516700	-5.58193100	0.93700600
C	5.85004700	-3.47755700	0.58876200
C	3.94841100	-2.47359200	1.82036600
H	3.33429100	-2.76416000	2.67815400
H	3.90668500	-1.38208300	1.76445400
C	5.41197500	-2.96966400	1.98161900
H	5.43650200	-3.82033600	2.67480100
C	5.47689800	-2.42130200	-0.46396800
H	5.94207900	-2.64221700	-1.42895700
H	5.84267500	-1.43520400	-0.15728200
C	3.93749700	-2.42302900	-0.60573300
H	3.65852500	-3.07529400	-1.44489900
H	6.92699500	-3.66587400	0.57815400
C	6.29972300	-1.89461900	2.53714800
H	6.37080900	-0.97387500	1.95470100
C	6.97883200	-1.99261300	3.67662700
H	6.92685100	-2.88973600	4.28840900
H	7.60767200	-1.18530300	4.03718900
C	3.32336100	-1.04789300	-0.91321600
H	3.37612900	-0.41448700	-0.02674900
C	4.05187500	-0.38542700	-2.06886400
C	3.83026000	-0.81368500	-3.35417700
C	5.02123500	0.64189300	-1.85744000
C	4.58094900	-0.25847800	-4.41657900
C	5.72982500	1.11868200	-2.99766500
N	5.50431400	0.65989100	-4.26319400
H	3.08447100	-1.57481600	-3.56153200
H	4.40402400	-0.60773500	-5.43207700
C	5.31199800	1.21936500	-0.58941800
H	4.75260900	0.91414000	0.28883400
C	6.72299100	2.12096300	-2.83489300
C	6.27528600	2.18746300	-0.46458600
H	7.24531000	2.45399200	-3.72536500
H	6.48052500	2.62068500	0.50874900
C	6.99574300	2.63919800	-1.59695500

H	7.75544300	3.40492500	-1.47984500
N	1.91360200	-1.26860400	-1.22693900
H	1.48379400	-2.19303800	-1.04986900
C	1.08003400	-0.26389300	-1.41617300
C	-0.31215600	-0.16605600	-1.66452100
N	-1.22054300	-1.15804500	-1.75332000
H	-0.83726400	-2.08130800	-1.41687000
C	-2.60316700	-1.02858600	-1.67886300
C	-3.28385100	0.16495700	-1.92677300
C	-3.33019900	-2.17801300	-1.32197600
C	-4.67006800	0.20408000	-1.77678300
C	-4.70606500	-2.10842400	-1.19733200
C	-5.40082600	-0.91571500	-1.41193100
H	-2.74290400	1.05942200	-2.22087400
H	-2.79439600	-3.09159600	-1.08914900
H	-6.47736200	-0.86828500	-1.29729400
C	-5.36027300	1.52287100	-1.97677700
F	-5.15268100	2.35036200	-0.93095200
F	-4.90574000	2.16310600	-3.06183400
F	-6.68543900	1.38865400	-2.10716400
C	-5.49352100	-3.33799700	-0.84493800
F	-4.73009700	-4.30236700	-0.32337700
F	-6.46153100	-3.05768400	0.04892800
F	-6.10998400	-3.85274900	-1.92066500
C	1.21900100	1.20419300	-1.37474900
O	2.09200200	1.99356400	-1.07148000
C	-0.25003500	1.29826100	-1.71162900
O	-1.03036600	2.24068800	-1.83092500
H	1.63416200	-2.86869000	1.09029900
O	-1.31429500	-2.21588800	1.00551700
O	0.06573700	-3.11541400	-0.52753900
C	-0.22727200	-2.68543300	0.64963000
O	0.77077600	-2.72583400	1.56458300
C	-0.03253200	2.97838800	1.25070600
C	1.16141500	2.34670200	1.97300100
C	-0.13686900	0.19851500	2.68133600
C	-1.45380300	0.61553900	2.01776900
H	-0.12312400	2.57579100	0.24232300
H	2.06078500	2.62171500	1.41742900
H	1.23920900	2.73938100	2.99476500
H	-0.06196700	-0.88859400	2.59183700
H	-0.14394400	0.48117100	3.74201100
H	-1.43675100	0.34963400	0.95326500
C	1.09423200	0.82598100	2.04412700
O	2.01467600	0.14822100	1.64477300
S	-1.58202700	2.46373400	2.10790400

C	-0.00345600	4.48243300	1.15992400
C	-0.07899400	5.07250500	-0.10354000
C	0.09852100	5.28270700	2.30145400
C	-0.04166000	6.45970000	-0.22281400
C	0.13036000	6.66743700	2.17670500
C	0.06252700	7.25624800	0.91447200
H	-0.16329300	4.43656500	-0.98163800
H	0.15096900	4.82871900	3.28799800
H	-0.09462300	6.91635000	-1.20527800
H	0.20952500	7.28697100	3.06358000
H	0.09030100	8.33669500	0.81973700
C	-2.70635900	0.07023400	2.65922500
C	-3.66841600	-0.54279700	1.85521200
C	-2.93790600	0.20731700	4.03048200
C	-4.84478900	-1.02918700	2.41630600
C	-4.11608600	-0.27762700	4.59040300
C	-5.06934000	-0.89757000	3.78489400
H	-3.47229800	-0.66545700	0.79533200
H	-2.20343100	0.69270700	4.66796300
H	-5.57514200	-1.52213400	1.78279300
H	-4.28797400	-0.17255100	5.65629800
H	-5.98467300	-1.27939900	4.22485800
C	-2.83034500	2.81166600	0.85076600
H	-2.46053200	2.48537700	-0.12491700
H	-3.73164600	2.27106600	1.14178900
H	-3.01125600	3.88647900	0.85957200

(TS)

N	3.31534900	-3.25819500	0.37058700
C	3.73793700	-4.65701200	0.13986600
H	3.49544200	-5.22029100	1.04546200
H	3.13584700	-5.06646800	-0.67569900
C	5.25426800	-4.71124300	-0.17382600
H	5.42406600	-4.86675700	-1.24445500
H	5.72428600	-5.54482200	0.35542400
C	5.87724000	-3.37557400	0.26249600
C	3.91103500	-2.78310200	1.64499600
H	3.37004200	-3.26213400	2.46598700
H	3.71051400	-1.70999800	1.73395300
C	5.42829700	-3.09551000	1.71444600
H	5.58797400	-4.00950400	2.30059700
C	5.33914400	-2.25403600	-0.63805700
H	5.79825400	-2.28411700	-1.63003500
H	5.58637300	-1.27936800	-0.20647900
C	3.80806100	-2.43766800	-0.76445100
H	3.60070600	-3.01819200	-1.67354500

H	6.96818700	-3.41894300	0.20829900
C	6.18899400	-1.98197300	2.37517100
H	6.08661700	-0.99044800	1.92961600
C	6.95283500	-2.13199000	3.45342500
H	7.06907800	-3.10095800	3.93238200
H	7.48495600	-1.29612100	3.89519600
C	3.00002600	-1.12906900	-0.90534700
H	2.83663400	-0.69836300	0.08951600
C	3.74075800	-0.14896100	-1.79399300
C	3.73231500	-0.31275400	-3.15530000
C	4.51295600	0.91705600	-1.24165300
C	4.49894100	0.55749400	-3.96633800
C	5.25653300	1.72244900	-2.14943900
N	5.24276300	1.53149000	-3.50168700
H	3.14200100	-1.09974500	-3.61395900
H	4.49127100	0.42193700	-5.04627600
C	4.57244200	1.22312000	0.14740400
H	3.97672200	0.66537700	0.86617400
C	6.05703800	2.78253700	-1.64800100
C	5.35181000	2.25945900	0.59937400
H	6.61365600	3.37113700	-2.36953900
H	5.37931800	2.48591500	1.66035800
C	6.10964500	3.04272700	-0.30387200
H	6.72228200	3.85539400	0.07221700
N	1.68648400	-1.46959900	-1.47127600
H	1.36581300	-2.43122700	-1.35313300
C	0.74030200	-0.54011500	-1.62738300
C	-0.65681500	-0.57340500	-1.81572800
N	-1.52709700	-1.60019600	-1.89696300
H	-1.14648400	-2.49312100	-1.54825100
C	-2.90911500	-1.43696100	-1.72461200
C	-3.59742400	-0.35953800	-2.28431400
C	-3.60264500	-2.38777500	-0.96935000
C	-4.95789700	-0.21869400	-2.03704500
C	-4.97000800	-2.24247000	-0.77370200
C	-5.66621400	-1.15495500	-1.29310300
H	-3.07597100	0.38099800	-2.87761100
H	-3.05507900	-3.20051200	-0.50238700
H	-6.72801700	-1.03545400	-1.11461500
C	-5.63621700	1.03060800	-2.52028100
F	-5.36613100	2.06682700	-1.70722900
F	-5.22226400	1.38162200	-3.74558300
F	-6.96857700	0.89933300	-2.56282100
C	-5.69809400	-3.28584900	0.02608500
F	-4.98819800	-3.67322700	1.09318300
F	-6.88251000	-2.83377600	0.47177000

F	-5.94635900	-4.38594500	-0.70026000
C	0.75076000	0.94220800	-1.62844400
O	1.56810500	1.82862400	-1.49765600
C	-0.75401500	0.89032600	-1.73333000
O	-1.63980700	1.72930700	-1.64293100
H	1.79409200	-3.08029200	0.85723100
O	-1.06730300	-2.01604300	0.89895700
O	-0.09885900	-3.49303100	-0.47186400
C	-0.08730900	-2.78175000	0.55567100
O	0.96765700	-2.72442700	1.34408300
C	0.01357700	2.90539200	1.01974800
C	1.19141000	2.09523700	1.55202400
C	-0.35426400	0.09995900	2.25982300
C	-1.58891600	0.83749700	1.85233400
H	-0.35535900	2.48987800	0.08046900
H	1.96483900	2.10811000	0.77789700
H	1.61885700	2.59674900	2.42924500
H	-0.64829800	-1.11791300	1.52750700
H	-0.40575000	-0.36931000	3.24313200
H	-1.72733600	0.84914900	0.76229600
C	0.97674000	0.63993900	2.00753900
O	2.00919200	0.01144600	2.24693400
S	-1.41442300	2.70188700	2.17096900
C	0.30095700	4.36873700	0.80735200
C	0.15276900	4.89898500	-0.47565600
C	0.70792700	5.19653400	1.85745600
C	0.41944300	6.24589500	-0.70893400
C	0.96771900	6.54267800	1.62275100
C	0.82580900	7.06798700	0.33887100
H	-0.16166900	4.24403300	-1.28411100
H	0.81872400	4.79113600	2.86042200
H	0.30867700	6.65160000	-1.70894400
H	1.28220400	7.18197600	2.44093300
H	1.03169200	8.11771200	0.15732200
C	-2.86435600	0.36125100	2.49437200
C	-3.94423500	-0.00749800	1.68981500
C	-2.97800500	0.27343100	3.88493200
C	-5.11847200	-0.48338400	2.26930100
C	-4.15570200	-0.18812500	4.46213400
C	-5.22525800	-0.57308200	3.65436200
H	-3.85848800	0.06878500	0.60727500
H	-2.14030700	0.56482700	4.51333900
H	-5.94791800	-0.78747200	1.63897000
H	-4.23782800	-0.25392100	5.54189600
H	-6.14003200	-0.94323600	4.10526900
C	-2.74285600	3.37912300	1.15068600

H	-2.71375900	2.90297400	0.16598400
H	-3.68308900	3.17009200	1.66146500
H	-2.58631500	4.45491200	1.06646800

(II)

N	-1.74940300	-3.65155900	-0.89891800
C	-1.73833200	-5.14555200	-1.01282500
H	-1.32351500	-5.37527000	-1.99616400
H	-1.05044700	-5.52319900	-0.25355400
C	-3.17985300	-5.66386500	-0.83888100
H	-3.31298500	-6.10497500	0.15310100
H	-3.38203000	-6.44413900	-1.57598700
C	-4.14592500	-4.48216900	-1.01951100
C	-2.41790700	-3.06537500	-2.10485500
H	-1.72820900	-3.20341700	-2.94018300
H	-2.52767800	-1.99186000	-1.93267700
C	-3.77999100	-3.75681300	-2.33602800
H	-3.68013000	-4.51661500	-3.12032300
C	-3.96487700	-3.51356900	0.15765500
H	-4.39949000	-3.91881400	1.07432300
H	-4.47508900	-2.56610500	-0.04472100
C	-2.45738100	-3.27411300	0.37465100
H	-2.07035500	-3.96771100	1.13150700
H	-5.17886600	-4.83340200	-1.06724200
C	-4.81280100	-2.75726000	-2.77875800
H	-4.92620800	-1.86628100	-2.15963400
C	-5.56536400	-2.90183700	-3.86489500
H	-5.46247800	-3.76848500	-4.51303100
H	-6.30689300	-2.16110900	-4.14391000
C	-2.11760300	-1.85273400	0.83358200
H	-2.37246600	-1.14061400	0.04221100
C	-2.92723700	-1.53999500	2.08882800
C	-2.56764600	-2.12424400	3.27803700
C	-4.07649200	-0.69293700	2.06717800
C	-3.35476800	-1.90335200	4.43173800
C	-4.79245200	-0.53698900	3.29023400
N	-4.43024500	-1.15347500	4.45260000
H	-1.68062500	-2.74708600	3.34570700
H	-3.07103800	-2.37833100	5.36887200
C	-4.52280300	0.02577900	0.92216600
H	-3.97008700	-0.02354900	-0.00911600
C	-5.93406700	0.30835800	3.32871000
C	-5.61829000	0.84590900	0.99648800
H	-6.45394500	0.40088200	4.27641700
H	-5.91808100	1.42074500	0.12530400
C	-6.33665300	0.98653000	2.20910300

H	-7.19774800	1.64628900	2.24821300
N	-0.67915900	-1.77267700	1.07197200
H	-0.07595600	-2.60403200	1.03229100
C	-0.08347100	-0.58171700	1.13231100
C	1.23842800	-0.12051700	1.19226200
N	2.38169000	-0.83918600	1.25532600
H	2.25813600	-1.85438700	1.21815600
C	3.69072600	-0.37908300	1.14168600
C	4.02328700	0.97760700	1.19026700
C	4.68873300	-1.33651100	0.93041500
C	5.34909800	1.34854500	1.01350600
C	6.00277000	-0.92887800	0.75793200
C	6.35744800	0.41461200	0.80181700
H	3.25675100	1.73669200	1.30651900
H	4.42128000	-2.38588800	0.84511200
H	7.38400400	0.72524400	0.64284600
C	5.71653400	2.80540400	1.01448500
F	4.65339200	3.59729800	0.83552500
F	6.30190700	3.17478900	2.16494900
F	6.59321500	3.08311700	0.03183400
C	7.02976300	-1.94538100	0.35633200
F	7.07045000	-2.07426100	-0.98427100
F	8.26328300	-1.60105700	0.75042200
F	6.76610400	-3.16005200	0.85519900
C	-0.62612000	0.77887800	1.10504200
O	-1.73982600	1.26006800	1.05146900
C	0.79666500	1.29724700	1.09833800
O	1.29557300	2.39892300	0.99102700
H	-0.71310300	-3.33341400	-0.96101500
O	2.87729900	-3.32748300	-1.09695200
O	1.45463400	-3.45744200	0.58948400
C	1.59748700	-3.30796800	-0.64363200
O	0.69442300	-3.11713400	-1.50434300
C	-2.06164600	3.38408800	-1.27063600
C	-2.19100700	2.39788600	-2.46128600
C	-0.40637200	0.58474500	-1.96599600
C	0.60648800	1.46260100	-2.08485900
H	-1.41434900	2.94975600	-0.50244100
H	-3.22065000	2.36345100	-2.81794000
H	-1.55403100	2.70844000	-3.29452600
H	2.83890700	-3.25221000	-2.06115300
H	-0.23426400	-0.47640200	-1.78020200
H	0.36776300	2.51750600	-2.22726900
C	-1.82470700	0.97946900	-2.05931500
O	-2.70926400	0.15998300	-1.83837100
S	-1.17767400	4.88505800	-1.86961800

**C**	**-3.38652700**	**3.73770200**	**-0.62774200**
**C**	**-3.51395900**	**3.72438200**	**0.76273000**
**C**	**-4.48464300**	**4.13627600**	**-1.39981100**
**C**	**-4.71675100**	**4.08627200**	**1.36931200**
**C**	**-5.68639800**	**4.49013600**	**-0.79698500**
**C**	**-5.80693300**	**4.46487400**	**0.59341400**
**H**	**-2.67365200**	**3.39622600**	**1.36517900**
**H**	**-4.39390700**	**4.18671700**	**-2.48201400**
**H**	**-4.80159300**	**4.05592300**	**2.45109800**
**H**	**-6.52812300**	**4.79428100**	**-1.41123900**
**H**	**-6.74510700**	**4.74119800**	**1.06456400**
**C**	**2.04249800**	**1.16975400**	**-2.04725700**
**C**	**2.93534800**	**2.25171800**	**-2.06287800**
**C**	**2.55694900**	**-0.13574200**	**-2.04475300**
**C**	**4.30817700**	**2.04122700**	**-2.10842200**
**C**	**3.93174700**	**-0.34333300**	**-2.08631300**
**C**	**4.80832100**	**0.74077100**	**-2.13307800**
**H**	**2.53795300**	**3.26299200**	**-2.04275600**
**H**	**1.87949500**	**-0.98647600**	**-2.02552700**
**H**	**4.98728400**	**2.88739000**	**-2.11284100**
**H**	**4.33116500**	**-1.35241200**	**-2.06520900**
**H**	**5.87946100**	**0.56624700**	**-2.16413300**
**C**	**-0.81861500**	**5.64943700**	**-0.26632600**
**H**	**-0.22012000**	**4.96991200**	**0.34507300**
**H**	**-0.24900800**	**6.55898700**	**-0.46015600**
**H**	**-1.74411600**	**5.90553600**	**0.25310200**

(I')

**N**	**3.82569800**	**-1.66619300**	**-1.84480800**
**C**	**4.10407600**	**-1.32458700**	**-3.25818800**
**H**	**3.90761400**	**-2.22358800**	**-3.85040600**
**H**	**3.37499300**	**-0.56812600**	**-3.56219600**
**C**	**5.56406800**	**-0.84149500**	**-3.44103700**
**H**	**5.59893300**	**0.23864400**	**-3.61951500**
**H**	**6.02697200**	**-1.33030000**	**-4.30327400**
**C**	**6.33030700**	**-1.17540000**	**-2.15424900**
**C**	**4.62345400**	**-2.85107800**	**-1.48198800**
**H**	**4.18984100**	**-3.71514500**	**-1.99461900**
**H**	**4.50630400**	**-3.03637200**	**-0.40938400**
**C**	**6.12454300**	**-2.67677500**	**-1.85074900**
**H**	**6.33988500**	**-3.23543700**	**-2.77047400**
**C**	**5.73954900**	**-0.32289200**	**-1.02318900**
**H**	**6.01779100**	**0.72387300**	**-1.17012000**
**H**	**6.15727200**	**-0.61978300**	**-0.05296800**
**C**	**4.19340000**	**-0.49209700**	**-1.02274700**
**H**	**3.69723500**	**0.35660200**	**-1.50752400**

H	7.39706900	-0.96350000	-2.26945100
C	7.02818700	-3.19824100	-0.77189400
H	6.90523600	-2.75833500	0.21927700
C	7.93945400	-4.15114000	-0.94689200
H	8.08414500	-4.62140700	-1.91627100
H	8.56848000	-4.49511500	-0.13256700
C	3.64803200	-0.58922600	0.41265600
H	4.15428100	-1.44241600	0.88692300
C	3.96330900	0.62478800	1.26510600
C	4.32420700	1.83293800	0.73135700
C	3.84937900	0.53017600	2.68824800
C	4.58315800	2.93095600	1.58851200
C	4.12807200	1.69556800	3.45416300
N	4.49951200	2.88378400	2.89283800
H	4.40438500	1.98461700	-0.33843700
H	4.87257900	3.88462600	1.15090200
C	3.45764700	-0.65307600	3.37030800
H	3.21516200	-1.54704800	2.80497700
C	4.02423300	1.64365100	4.86797900
C	3.36109500	-0.67310800	4.73961800
H	4.24762600	2.55107600	5.41869400
H	3.05816700	-1.58404600	5.24506100
C	3.64897300	0.48565000	5.49848200
H	3.56775600	0.45345200	6.57988700
N	2.22011900	-0.90345000	0.43665600
H	1.93792900	-1.76552900	-0.06597500
C	1.22188000	-0.18845800	0.93211700
C	-0.16307600	-0.51908400	1.01145500
N	-0.82641500	-1.59757600	0.57935400
H	-0.23723000	-2.30789400	0.00948000
C	-2.19219500	-1.83254800	0.76290000
C	-2.90493300	-1.32900400	1.85594600
C	-2.84527500	-2.63107500	-0.18733100
C	-4.25762200	-1.62815200	1.97904600
C	-4.19427100	-2.91529700	-0.02903800
C	-4.91798300	-2.42858000	1.05519600
H	-2.41774100	-0.70181900	2.59303200
H	-2.27967800	-2.99351000	-1.04605100
H	-5.97545000	-2.64207400	1.15992100
C	-5.05056500	-0.97014100	3.06798200
F	-4.31418700	-0.71721800	4.15658600
F	-5.54635200	0.21651300	2.65186600
F	-6.10123600	-1.70970200	3.44806300
C	-4.87874500	-3.78394500	-1.04576300
F	-4.75555500	-5.08868800	-0.74964000
F	-6.19597200	-3.52724200	-1.11436400

F	-4.36970900	-3.61250300	-2.27243000
C	0.93234800	1.18683900	1.45232800
O	1.50713700	2.25049900	1.58552100
C	-0.50730800	0.78624000	1.55562400
O	-1.54645300	1.40260400	1.80435800
C	0.49944900	-2.93467600	-2.01662900
O	0.84648500	-2.96360700	-0.77660600
O	1.51361800	-2.78821300	-2.89436200
H	2.25670200	-2.39836500	-2.37200700
O	-0.65265300	-3.03401800	-2.45073400
C	-2.29752600	0.91211200	-1.31839000
C	-1.22596400	0.27526900	-2.20304300
C	0.49941900	2.22530500	-1.76149300
C	-0.50510900	3.03847100	-0.94461500
H	-2.06088200	0.81345400	-0.25595700
H	-1.25013100	-0.81587700	-2.08538500
H	-1.43749100	0.48530600	-3.26060600
H	1.46915100	2.30504400	-1.25844700
H	0.60933500	2.66788600	-2.76081600
H	-0.55992600	2.67688200	0.08452000
C	0.20446900	0.74243400	-1.96999700
O	1.12647800	-0.03455200	-2.07805200
S	-2.20381900	2.74802200	-1.60807800
C	-3.71036300	0.43215400	-1.53647300
C	-4.60959000	0.53029500	-0.46930000
C	-4.13312300	-0.13347700	-2.74160500
C	-5.92096700	0.09071500	-0.61137800
C	-5.44080700	-0.59194900	-2.87349400
C	-6.33788800	-0.47375900	-1.81440200
H	-4.26720400	0.91146100	0.49013300
H	-3.44528900	-0.24581700	-3.57284500
H	-6.60012500	0.15809100	0.23211900
H	-5.75511300	-1.05166200	-3.80424800
H	-7.35330900	-0.84037100	-1.92042800
C	-0.21369000	4.51776200	-0.92116600
C	0.15814600	5.10038700	0.29209800
C	-0.28484200	5.29817500	-2.07889300
C	0.46015500	6.45912700	0.34419400
C	0.00916100	6.65635400	-2.01943300
C	0.38218600	7.23716700	-0.80799500
H	0.23222400	4.47743200	1.17987800
H	-0.57338100	4.84992200	-3.02690500
H	0.75478300	6.90802200	1.28663400
H	-0.05171800	7.26082700	-2.91802600
H	0.61287900	8.29635600	-0.76436800
C	-3.17287100	3.44186100	-0.25173100

H	-2.85042300	2.97751300	0.68422800
H	-2.97439500	4.51449100	-0.25280500
H	-4.22414300	3.25157000	-0.46349800

(I'')

N	-3.17145800	-2.84598500	-0.23130900
C	-3.46573700	-4.29203900	-0.17192700
H	-3.46480400	-4.66715600	-1.19959800
H	-2.64614200	-4.78354700	0.35859600
C	-4.83612500	-4.52587700	0.51708900
H	-4.69960000	-4.86603300	1.54916100
H	-5.40292400	-5.29980900	-0.00825800
C	-5.60911800	-3.19458000	0.50652300
C	-4.10224700	-2.21414700	-1.18405600
H	-3.77600500	-2.44427000	-2.20323100
H	-4.01600100	-1.12923300	-1.06665800
C	-5.57073300	-2.65170400	-0.93929200
H	-5.82737200	-3.47069800	-1.62282600
C	-4.89145000	-2.19192200	1.42852600
H	-5.07302000	-2.42373400	2.48175700
H	-5.27471500	-1.18032800	1.26026100
C	-3.38052100	-2.28515700	1.12305400
H	-2.92914300	-2.99982400	1.82360900
H	-6.64265800	-3.34174100	0.83098000
C	-6.51468800	-1.51372900	-1.19781200
H	-6.42990900	-0.65126200	-0.53260800
C	-7.40784400	-1.48276800	-2.18423900
H	-7.51956700	-2.31898900	-2.86972900
H	-8.05939900	-0.62863000	-2.33653500
C	-2.55764900	-0.99222800	1.27602000
H	-2.71765400	-0.35891700	0.39553300
C	-2.94197300	-0.21681200	2.52259300
C	-2.41820800	-0.56978600	3.74052800
C	-3.87372300	0.86583600	2.47796000
C	-2.81770600	0.12956800	4.90435300
C	-4.20228000	1.50097000	3.70925800
N	-3.67258300	1.12314100	4.90912000
H	-1.69332500	-1.37362400	3.81534600
H	-2.40021000	-0.15966500	5.86677600
C	-4.48624200	1.34911100	1.28832400
H	-4.26053200	0.88566800	0.33302200
C	-5.12425100	2.58134200	3.71572700
C	-5.37559100	2.39307800	1.32424200
H	-5.34833000	3.03825300	4.67373400
H	-5.83256800	2.74428300	0.40487300
C	-5.70035900	3.01801100	2.55214200

H	-6.40491600	3.84279400	2.56576300
N	-1.16026900	-1.40604400	1.31324800
H	-0.89882100	-2.34420200	0.94452800
C	-0.17180700	-0.51836400	1.33198500
C	1.23718200	-0.63141700	1.27888000
N	1.99655900	-1.71052000	1.07337700
H	1.47088800	-2.54750000	0.74642100
C	3.38719700	-1.73347200	0.93291700
C	4.21639100	-0.81274900	1.56669300
C	3.93438600	-2.74740700	0.13653300
C	5.59413600	-0.90767700	1.38550000
C	5.30881900	-2.82788200	-0.00465200
C	6.15973900	-1.91114300	0.61415100
H	3.79813300	-0.01353400	2.16898800
H	3.26469600	-3.41255200	-0.40003800
H	7.23486200	-1.98120700	0.49509400
C	6.46087800	0.13051300	2.03741100
F	6.32457600	1.32882900	1.44749200
F	6.13944300	0.30157300	3.32992200
F	7.76180200	-0.18963800	1.98688700
C	5.92707300	-3.89428000	-0.86163700
F	6.56642600	-3.36714800	-1.91911400
F	6.84533900	-4.59840900	-0.17664200
F	5.02493100	-4.76288400	-1.33118900
C	-0.10224100	0.93928600	1.36359700
O	-0.90373000	1.85706300	1.20967100
C	1.40281100	0.83212900	1.43478900
O	2.31669500	1.63393600	1.50639600
H	-1.63396100	-2.46743800	-1.07742200
O	1.30705200	-2.64988400	-1.76797500
O	0.12160900	-3.36372200	0.00261100
C	0.25654500	-2.78138400	-1.13485500
O	-0.86680500	-2.20524800	-1.65083500
C	-0.25128400	1.10472200	-2.26196800
C	1.10833800	0.47403900	-2.57115200
C	2.46652100	2.42228000	-1.44698000
C	1.20348400	3.15960100	-1.00362400
H	-0.54699200	0.90189700	-1.22572100
H	1.01988200	-0.61677500	-2.47726800
H	1.40271000	0.70927300	-3.60220800
H	3.21376800	2.53027100	-0.65861700
H	2.86295100	2.86431100	-2.37044000
H	0.75675200	2.67673900	-0.13276100
C	2.26769200	0.92956100	-1.69001400
O	3.07202500	0.13407000	-1.26703600
S	-0.05822300	2.95175200	-2.33468300

C	-1.37984900	0.73579500	-3.19232700
C	-2.66668000	0.60687900	-2.66575600
C	-1.18569600	0.56944800	-4.56531200
C	-3.74623800	0.30269800	-3.48919400
C	-2.26404700	0.26434800	-5.39054300
C	-3.54343700	0.12870500	-4.85598100
H	-2.81445900	0.74898000	-1.59680700
H	-0.19649900	0.66882000	-4.99985000
H	-4.73884100	0.18381100	-3.06317000
H	-2.10203700	0.12781200	-6.45425100
H	-4.37970400	-0.11475900	-5.50259500
C	1.37703300	4.62326000	-0.70102600
C	0.94119800	5.09488200	0.53934800
C	1.95275800	5.50469600	-1.62015800
C	1.08938600	6.44100500	0.86264800
C	2.09446300	6.84971600	-1.29489100
C	1.66487700	7.31780000	-0.05366800
H	0.49475400	4.39645800	1.24315200
H	2.29165500	5.14532500	-2.58847700
H	0.75637600	6.80273600	1.82948300
H	2.54294200	7.53237700	-2.00864600
H	1.78029800	8.36675500	0.19806800
C	-1.54792000	3.54026700	-1.50216300
H	-1.65065200	3.02345900	-0.54384500
H	-1.42086000	4.61395700	-1.35863800
H	-2.38937900	3.34673900	-2.16718700

(TS')

N	3.61580000	0.16726000	-2.16695300
C	3.64311100	1.52276300	-2.76509600
H	3.37227700	1.41161500	-3.81982100
H	2.85943800	2.10977800	-2.28081100
C	5.03958400	2.17283200	-2.60538100
H	5.01387200	2.96680000	-1.85165100
H	5.36419700	2.62527800	-3.54689100
C	6.02052900	1.07757600	-2.16712600
C	4.49598000	-0.70963900	-2.96640200
H	4.00188600	-0.88815200	-3.92624000
H	4.56751300	-1.68229500	-2.47019900
C	5.90668000	-0.09057100	-3.17114100
H	5.97544500	0.32975000	-4.18250200
C	5.61065600	0.60702100	-0.76461900
H	5.83979600	1.38784800	-0.03518100
H	6.19079100	-0.27508300	-0.46672100
C	4.09072700	0.28345200	-0.76293200
H	3.50704400	1.10233100	-0.32227900

H	7.04636200	1.45632700	-2.15330500
C	6.99134100	-1.11763100	-3.02364200
H	7.03123300	-1.64910300	-2.07138200
C	7.87774900	-1.41708800	-3.96886200
H	7.86357700	-0.91678900	-4.93390000
H	8.64433800	-2.16898900	-3.81386900
C	3.76981500	-0.99329100	0.03211300
H	4.27882000	-1.82145900	-0.47414300
C	4.27153600	-1.00818600	1.46947700
C	4.61683000	0.12756900	2.15218000
C	4.38262600	-2.26044900	2.15667400
C	5.08262800	0.03220900	3.48786800
C	4.86957500	-2.24243800	3.49387500
N	5.21741500	-1.09224400	4.14417600
H	4.51357500	1.10806000	1.70709600
H	5.35230400	0.94366300	4.01768700
C	4.03608000	-3.51293100	1.58167900
H	3.63740500	-3.55359900	0.57423900
C	5.00839300	-3.46188500	4.20610900
C	4.17803800	-4.67686200	2.29517300
H	5.38585200	-3.40621100	5.22163300
H	3.90599800	-5.62343000	1.84006900
C	4.67155800	-4.65451000	3.62091400
H	4.77795100	-5.58358600	4.17094700
N	2.35159000	-1.33928600	-0.01145800
H	2.02846600	-1.87613100	-0.82245900
C	1.39937900	-0.75772300	0.71128900
C	0.00965300	-0.97481800	0.75123000
N	-0.76742200	-1.80788900	0.02579100
H	-0.32784900	-2.21143800	-0.81252100
C	-2.13849800	-2.00396800	0.18022100
C	-2.79212400	-1.79490400	1.39872900
C	-2.86323400	-2.46424300	-0.92761800
C	-4.15203900	-2.06960500	1.49167600
C	-4.21365400	-2.75042700	-0.79360100
C	-4.87630200	-2.56865000	0.41608000
H	-2.25323400	-1.41461800	2.25851900
H	-2.36115700	-2.55362500	-1.88639000
H	-5.93579600	-2.77726300	0.50731600
C	-4.88255400	-1.71165200	2.75171700
F	-4.09762000	-1.77318400	3.83368100
F	-5.35891400	-0.44891100	2.68815700
F	-5.93908700	-2.50704400	2.96666700
C	-4.96257300	-3.29993700	-1.97439700
F	-4.85028500	-4.63706500	-2.04974000
F	-6.27489300	-3.02609800	-1.90648900

F	-4.50448200	-2.80664000	-3.13265900
C	1.27318900	0.41901100	1.61815000
O	1.98219300	1.32047100	2.00899000
C	-0.21098000	0.14423400	1.65789300
O	-1.18582200	0.74031200	2.10567600
C	0.39790900	-1.15860200	-2.76107600
O	0.84750100	-2.18123800	-2.19575800
O	1.21977000	-0.25179400	-3.28016100
H	2.08059000	-0.24675600	-2.74907100
O	-0.85587100	-0.90584600	-2.86641200
C	-2.29566600	1.28358500	-0.97937000
C	-1.30563700	1.52126000	-1.98812800
C	0.01340500	3.50130700	-0.98446000
C	-0.78118300	3.63872500	0.33282800
H	-1.94312600	0.90150400	-0.01868800
H	-1.07969000	0.29299400	-2.48613000
H	-1.66557800	2.12849300	-2.82307300
H	1.04946300	3.78242800	-0.78040400
H	-0.40004700	4.15629500	-1.75804600
H	-0.50422500	2.81443600	1.00109700
C	-0.02415100	2.03619600	-1.38594000
O	0.89263500	1.30639400	-1.06592900
S	-2.57091600	3.40518300	-0.03804200
C	-3.67871500	0.88352900	-1.30200200
C	-4.58627000	0.68743400	-0.25335900
C	-4.08306200	0.64074100	-2.61881100
C	-5.89183600	0.29776400	-0.51784900
C	-5.38424500	0.22268300	-2.87840200
C	-6.29261600	0.06494100	-1.83324100
H	-4.25420500	0.81808300	0.77359600
H	-3.37272700	0.74047500	-3.43325300
H	-6.58255000	0.14424300	0.30498400
H	-5.68737600	0.01139000	-3.89813400
H	-7.30580800	-0.26193900	-2.04179100
C	-0.53889000	4.94276600	1.05009800
C	0.03445100	4.91714600	2.32261800
C	-0.85975300	6.16956600	0.46193000
C	0.29041100	6.10781000	2.99980600
C	-0.60415400	7.35677400	1.13949600
C	-0.02848000	7.32754900	2.40968300
H	0.28853900	3.96043500	2.77261800
H	-1.31668900	6.19385600	-0.52422600
H	0.73736300	6.07992300	3.98801300
H	-0.85536100	8.30607900	0.67798600
H	0.16888000	8.25495700	2.93714200
C	-3.20444000	3.24786400	1.65067500

H	-2.72322400	2.40089900	2.14378500
H	-2.99476200	4.17622700	2.18543900
H	-4.28234700	3.10186500	1.58097800

(II')

N	2.74951000	0.86529700	-1.86334200
C	2.93254600	2.34777300	-2.00226000
H	2.14939300	2.68822300	-2.68276400
H	2.74906400	2.78071400	-1.01828500
C	4.35629200	2.65030700	-2.51087100
H	4.84236800	3.35522700	-1.83191700
H	4.32190700	3.10998400	-3.50213200
C	5.14235300	1.33569400	-2.56743700
C	3.00018700	0.21307100	-3.18743700
H	2.29863600	0.67738900	-3.88353200
H	2.71569100	-0.83785200	-3.11293000
C	4.47488000	0.42133900	-3.61911000
H	4.48109900	0.96133500	-4.57201700
C	5.11149700	0.68314000	-1.17826000
H	5.55203600	1.36962700	-0.44886000
H	5.71884600	-0.22657500	-1.16419200
C	3.65267300	0.35450100	-0.77415900
H	3.34367100	0.90242500	0.12400400
H	6.17774000	1.52249400	-2.86215900
C	5.20561900	-0.87657300	-3.83155300
H	5.24477200	-1.57526500	-2.99538800
C	5.80707900	-1.20552400	-4.97046700
H	5.79029300	-0.54060700	-5.83023300
H	6.33450600	-2.14652200	-5.08302100
C	3.40058200	-1.14403800	-0.53356000
H	3.70168100	-1.67994300	-1.44097900
C	4.23581000	-1.73240300	0.59447200
C	4.91643700	-0.96317200	1.50030200
C	4.33290600	-3.15574600	0.71392200
C	5.68535300	-1.58735800	2.51481100
C	5.13674800	-3.67629300	1.76672300
N	5.80435700	-2.88279300	2.65577600
H	4.86784000	0.11895400	1.48361100
H	6.21654100	-0.96481500	3.23161800
C	3.68576900	-4.07092500	-0.15952800
H	3.05250900	-3.70091200	-0.95789400
C	5.27026000	-5.08088500	1.91745200
C	3.83510500	-5.42480600	0.00906500
H	5.89014700	-5.43856700	2.73263800
H	3.33258300	-6.10953200	-0.66592600
C	4.63408100	-5.93798800	1.05801800

H	4.73856000	-7.01105300	1.17833400
N	1.98496900	-1.43868100	-0.37050000
H	1.43521200	-1.57280100	-1.23362700
C	1.27167400	-1.13887600	0.72993200
C	-0.09294400	-1.28986000	1.00782500
N	-1.07272000	-1.75341700	0.20171200
H	-0.77919200	-2.00869100	-0.74035200
C	-2.45439200	-1.71354900	0.40247600
C	-3.02961300	-1.39054800	1.63776900
C	-3.27320000	-1.99837100	-0.69285200
C	-4.41209700	-1.37097300	1.74406800
C	-4.65608000	-1.96445300	-0.55132500
C	-5.24633400	-1.66674800	0.66694100
H	-2.40990700	-1.12652000	2.48872000
H	-2.82758700	-2.20240500	-1.66159500
H	-6.32489400	-1.64056800	0.77137200
C	-5.05864900	-0.91361900	3.01880300
F	-4.20520400	-0.87067900	4.04485000
F	-5.57172000	0.32429400	2.87109200
F	-6.08145200	-1.70891400	3.37014800
C	-5.49727000	-2.21723600	-1.77021100
F	-5.26527300	-3.43619600	-2.28559200
F	-6.80824500	-2.13815100	-1.50513100
F	-5.22739600	-1.33278200	-2.74143200
C	1.51960200	-0.50305300	2.03524500
O	2.43519200	0.08585200	2.57726600
C	0.07017800	-0.77492400	2.39004200
O	-0.60468000	-0.60802200	3.38038300
C	-0.36057900	-0.21911900	-2.23182300
O	0.18902400	-1.32355600	-2.44748100
O	0.17209000	0.82130000	-1.74790500
H	1.68896300	0.72561300	-1.65043300
O	-1.67935000	-0.15919000	-2.52517800
C	-3.73223800	1.55411800	-0.50751200
C	-2.60678000	2.27086700	-0.66950600
C	-0.42315200	3.19078300	0.27767900
C	0.57887400	3.05349900	1.42057200
H	-3.79049100	0.92821200	0.38434500
H	-2.02319500	0.67839100	-2.17655800
H	-2.48682500	2.99379900	-1.47427100
H	0.08111900	3.07528300	-0.69109900
H	-0.90264200	4.17788400	0.26481700
H	0.74236700	1.98450100	1.59423300
C	-1.50284800	2.12716800	0.31603700
O	-1.47235900	1.20324900	1.10684500
S	-0.20877300	3.68612900	2.95686100

C	-4.89811800	1.52775000	-1.39482200
C	-6.13593100	1.13486400	-0.86859600
C	-4.80630000	1.84032500	-2.75755100
C	-7.25868700	1.05870600	-1.68453600
C	-5.92764700	1.75828100	-3.57335700
C	-7.15470700	1.36594600	-3.03941100
H	-6.20563400	0.89755100	0.18948300
H	-3.84813000	2.12092500	-3.18668600
H	-8.21150100	0.75176000	-1.26617400
H	-5.84383100	1.98824100	-4.63033000
H	-8.02679600	1.29416600	-3.68104000
C	1.90747200	3.71831300	1.15083300
C	3.08913600	3.11042600	1.59156200
C	1.99573700	4.91863100	0.43676300
C	4.33131900	3.67640500	1.30076700
C	3.23470800	5.48007600	0.14092000
C	4.40821300	4.85906600	0.56895800
H	3.02953400	2.17704600	2.14680100
H	1.09013100	5.41361800	0.09773500
H	5.23853700	3.19265700	1.65147900
H	3.28527600	6.40565000	-0.42361200
H	5.37351900	5.29779600	0.33725100
C	0.81982700	2.83079200	4.18037300
H	0.77593400	1.75041700	4.02068800
H	1.85471500	3.17609700	4.13151700
H	0.41053700	3.06803100	5.16320000

Equilibrium

(B_{anion})

H	1.73995100	-0.09645500	-0.00009200
O	1.02372600	-0.74261600	-0.00004100
O	0.07495500	1.28814500	-0.00006300
C	-0.14159000	0.05815000	0.00017300
O	-1.20018000	-0.58636200	-0.00006900

($1a_{cation}$)

C	-1.40480000	0.61422900	-0.45511500
C	-1.31929700	2.04573800	0.09225600
C	1.28772900	2.04777400	0.19317000
C	1.41282500	0.64123900	-0.40825200
H	-1.23619700	0.61342800	-1.53768400
H	-2.12814600	2.62702300	-0.35520900
H	-1.47127900	2.04097400	1.17818100
H	2.12959100	2.64840100	-0.15680100

H	1.33490600	1.99694100	1.28856100
H	1.25534500	0.68002700	-1.49174700
C	-0.00305700	2.75966200	-0.19775900
O	0.01397900	3.86658600	-0.67760600
S	0.00238800	-0.34352400	0.26351800
C	-2.68800200	-0.10806600	-0.13173600
C	-3.44665500	-0.65347600	-1.16880000
C	-3.12149500	-0.24333200	1.19114500
C	-4.63853700	-1.31642800	-0.88746500
C	-4.30839400	-0.91184700	1.46891000
C	-5.06922400	-1.44612000	0.42989200
H	-3.11103500	-0.55060900	-2.19688900
H	-2.53458500	0.16936400	2.00774700
H	-5.22804100	-1.73057100	-1.69800100
H	-4.64071400	-1.01372500	2.49611300
H	-5.99684800	-1.96371600	0.64904000
C	2.69668800	-0.08629300	-0.10362200
C	3.40468000	-0.68101100	-1.15038800
C	3.17514000	-0.19339000	1.20597200
C	4.58952400	-1.36514400	-0.89338600
C	4.35681300	-0.88134100	1.45948900
C	5.06569400	-1.46553800	0.41092500
H	3.03397700	-0.60075100	-2.16855100
H	2.63006400	0.25569000	2.03165800
H	5.13845600	-1.81739000	-1.71195900
H	4.72532000	-0.95974400	2.47636600
H	5.98860200	-1.99868700	0.61193600
C	0.02330700	-1.83888200	-0.74127100
H	0.02794300	-1.58463500	-1.80221000
H	0.92151300	-2.39254100	-0.46572800
H	-0.86838500	-2.40847500	-0.47706700

(I_{eq})

N	-3.33816600	-0.92749000	1.36280600
C	-3.52473000	-1.73378200	2.58437200
H	-3.09514900	-2.72295200	2.38847000
H	-2.94720100	-1.27807500	3.39516400
C	-5.03041100	-1.84671400	2.94628300
H	-5.27975100	-1.18720500	3.78463700
H	-5.27905300	-2.86766000	3.24987300
C	-5.84255700	-1.44074700	1.70504800
C	-3.87281700	-1.70157000	0.22776400
H	-3.18797600	-2.53020800	0.02993400
H	-3.86364800	-1.07474800	-0.66998900
C	-5.29830500	-2.25487600	0.51230400
H	-5.21869300	-3.30399900	0.82674700

C	-5.61402300	0.05850900	1.44950800
H	-6.16083700	0.65777900	2.18230600
H	-5.99527800	0.34833400	0.46234200
C	-4.09028900	0.32831800	1.54568300
H	-3.83954600	0.67723600	2.55661800
H	-6.90673300	-1.64390300	1.85230700
C	-6.15573900	-2.20963300	-0.71717200
H	-6.39468500	-1.22017100	-1.10991700
C	-6.59951900	-3.28585500	-1.36111000
H	-6.37094300	-4.28758800	-1.00538200
H	-7.20235800	-3.20575100	-2.25959200
C	-3.60809900	1.39352100	0.55169800
H	-3.68097200	0.96826400	-0.45200900
C	-4.37738200	2.70305500	0.60142600
C	-4.93376800	3.16582500	1.76686500
C	-4.46068800	3.53190100	-0.56147600
C	-5.58502700	4.42498900	1.78217000
C	-5.13813100	4.77826400	-0.43342000
N	-5.69385800	5.20718100	0.73834600
H	-4.89336400	2.59022300	2.68540100
H	-6.02909700	4.78033800	2.70967900
C	-3.90400700	3.18829000	-1.82351800
H	-3.38107800	2.24662400	-1.95707100
C	-5.24902900	5.63358200	-1.55992000
C	-4.02390900	4.03884000	-2.89405900
H	-5.77580400	6.57205200	-1.42493800
H	-3.59489100	3.76145900	-3.85104400
C	-4.70353200	5.27310200	-2.76434600
H	-4.79032100	5.93102100	-3.62244100
N	-2.18805600	1.70591100	0.76518300
H	-1.95460700	2.22349500	1.60477100
C	-1.23006400	1.31822400	-0.05434100
C	0.17391800	1.41242800	-0.08253100
N	1.07050200	1.93763200	0.78337900
H	0.75146800	2.08090700	1.73423900
C	2.46328800	1.80928500	0.62136700
C	3.06444300	1.99995800	-0.62567400
C	3.24897300	1.49367200	1.73024100
C	4.44019500	1.84672200	-0.74528200
C	4.62269300	1.33222600	1.57581500
C	5.23701600	1.50937600	0.34385200
H	2.46477200	2.25919600	-1.49144000
H	2.77867200	1.33578800	2.69772700
H	6.30392200	1.36439100	0.22814200
C	5.05514300	2.04988300	-2.10353600
F	4.51924800	1.21595400	-3.01306600

F	4.84558900	3.29331600	-2.55413900
F	6.37563200	1.83558100	-2.09575500
C	5.42086400	0.91099100	2.77781400
F	6.70320800	0.68600700	2.48303700
F	5.37596200	1.83061000	3.74943900
F	4.92294500	-0.22680400	3.30543600
C	-1.24212800	0.56400900	-1.33190500
O	-2.09596100	0.06523100	-2.03386000
C	0.27029700	0.62069600	-1.30685400
O	1.17085100	0.10229400	-1.95653100
C	0.03913400	-2.57896600	-0.25149300
C	-0.24282400	-2.16344200	1.19972500
C	2.25522000	-1.71006700	1.73342000
C	2.64924300	-1.76803600	0.25164800
H	0.07707800	-1.70116200	-0.89924400
H	-1.19826800	-1.61947000	1.20970000
H	-0.35151700	-3.05824700	1.82626600
H	2.89264200	-0.99113600	2.24867600
H	2.39540400	-2.68705400	2.21258100
H	2.20709300	-0.93444700	-0.30776600
C	0.80474700	-1.28413900	1.86479800
O	0.48991200	-0.29214400	2.48582400
S	1.75814300	-3.25911300	-0.41227300
C	-0.96909600	-3.54604300	-0.81374600
C	-1.62499800	-3.20908400	-2.00065300
C	-1.31912900	-4.71541100	-0.13151600
C	-2.65189400	-4.01742700	-2.48168300
C	-2.34140200	-5.52284100	-0.61895400
C	-3.01548100	-5.16841500	-1.78779500
H	-1.37125400	-2.28575800	-2.51533700
H	-0.80329200	-4.99505700	0.78352500
H	-3.17703100	-3.73763500	-3.38832000
H	-2.61626400	-6.42538000	-0.08389400
H	-3.82335800	-5.79150700	-2.15659800
C	4.12224900	-1.80357100	-0.04659100
C	4.55496700	-1.30700700	-1.28074000
C	5.05971100	-2.23887100	0.89257200
C	5.91226000	-1.25811700	-1.58061700
C	6.41886100	-2.16802400	0.59836500
C	6.84539800	-1.68599000	-0.63785700
H	3.82753100	-0.90808300	-1.98376900
H	4.74087500	-2.60702300	1.86252100
H	6.23772100	-0.85696900	-2.53428400
H	7.14505400	-2.48707400	1.33801100
H	7.90565100	-1.63323200	-0.86131000
C	1.97602300	-3.12676700	-2.19850800

**H**	**1.71445900**	**-2.11472000**	**-2.51888200**
**H**	**3.01632400**	**-3.36565300**	**-2.41861000**
**H**	**1.31619100**	**-3.87025200**	**-2.64767300**

(II_{eq})

**N**	**2.75701700**	**-2.35241900**	**0.15046400**
**C**	**2.94243500**	**-3.52586600**	**-0.72972500**
**H**	**2.75392700**	**-4.41716300**	**-0.12344700**
**H**	**2.16923200**	**-3.48744300**	**-1.50271200**
**C**	**4.37181200**	**-3.53650100**	**-1.32764200**
**H**	**4.35897800**	**-3.21711100**	**-2.37529400**
**H**	**4.79371000**	**-4.54544900**	**-1.29922300**
**C**	**5.23339200**	**-2.56867700**	**-0.50045600**
**C**	**3.58702700**	**-2.52726800**	**1.35507300**
**H**	**3.11666600**	**-3.28235200**	**1.99211700**
**H**	**3.57910000**	**-1.58745600**	**1.91776000**
**C**	**5.04444400**	**-2.93219000**	**0.99100700**
**H**	**5.15489300**	**-4.01971600**	**1.09026000**
**C**	**4.72639200**	**-1.13638200**	**-0.73396800**
**H**	**5.01576400**	**-0.77211900**	**-1.72417300**
**H**	**5.17550800**	**-0.45244700**	**-0.00570400**
**C**	**3.18559000**	**-1.15551200**	**-0.60360200**
**H**	**2.74129900**	**-1.25940300**	**-1.60286000**
**H**	**6.28836500**	**-2.65197800**	**-0.77599400**
**C**	**6.04451600**	**-2.28171700**	**1.90099000**
**H**	**6.05612400**	**-1.19009200**	**1.90964500**
**C**	**6.88880000**	**-2.94245900**	**2.68832600**
**H**	**6.90127500**	**-4.02919700**	**2.71458300**
**H**	**7.59217500**	**-2.42501500**	**3.33228600**
**C**	**2.57856500**	**0.13233900**	**-0.01743500**
**H**	**2.89241000**	**0.25250000**	**1.02502900**
**C**	**3.04988500**	**1.32800300**	**-0.83440600**
**C**	**2.46403900**	**1.58175300**	**-2.04993000**
**C**	**4.12391900**	**2.17324700**	**-0.41787800**
**C**	**2.94754000**	**2.63716700**	**-2.85715800**
**C**	**4.53516500**	**3.20230100**	**-1.31379000**
**N**	**3.94634800**	**3.41924000**	**-2.52583100**
**H**	**1.62946000**	**0.97888500**	**-2.39386300**
**H**	**2.47927300**	**2.82562200**	**-3.82150900**
**C**	**4.79646000**	**2.06095600**	**0.83043200**
**H**	**4.48811900**	**1.30785200**	**1.54728100**
**C**	**5.60889800**	**4.05840800**	**-0.95148000**
**C**	**5.82859200**	**2.90397800**	**1.15461500**
**H**	**5.89387300**	**4.82642600**	**-1.66282100**
**H**	**6.32424000**	**2.80400500**	**2.11453000**
**C**	**6.24535300**	**3.91188500**	**0.25237300**

H	7.06338300	4.57124000	0.52321300
N	1.13540200	-0.02250900	-0.07011200
H	0.71604000	-0.92273700	-0.39032100
C	0.29574000	0.92345400	0.33089600
C	-1.10584800	1.01150500	0.34003800
N	-1.99145800	0.07526800	-0.06706800
H	-1.55934900	-0.80020400	-0.43388100
C	-3.37838900	0.12384800	-0.03582900
C	-4.09842600	1.28781300	0.25449700
C	-4.06365300	-1.06762400	-0.32409300
C	-5.48889100	1.24341800	0.23756300
C	-5.44970500	-1.06853700	-0.34218800
C	-6.18653400	0.08091500	-0.06579200
H	-3.58088000	2.21136400	0.49871000
H	-3.49393900	-1.98188000	-0.48705400
H	-7.26971700	0.06682100	-0.07952200
C	-6.25318300	2.47914300	0.61658600
F	-6.43267800	2.56504700	1.94574600
F	-5.62019400	3.59873800	0.23841900
F	-7.47585600	2.50209900	0.06062800
C	-6.17596000	-2.32630600	-0.72262400
F	-7.37763300	-2.40756900	-0.12680900
F	-6.40184600	-2.38480300	-2.04773200
F	-5.48878300	-3.42734900	-0.39657900
C	0.44531100	2.25907500	0.93576700
O	1.37978000	2.94152000	1.30258100
C	-1.07480700	2.37066700	0.92280700
O	-1.87808500	3.22235500	1.25631400
H	1.06798000	-2.83880900	0.64989000
O	0.26237700	-3.34471000	0.91290700
O	-0.45562400	-2.05856600	-0.77646600
C	-0.76430500	-2.92789200	0.12082500
O	-1.88287900	-3.39536600	0.33226800

15. Supplementary References

[1] For the synthesis of 9-amino(9-deoxy)*epi*cinchona alkaloids, see: Cassani, C., Martín-Rapún, R., Arceo, E., Bravo, F. & Melchiorre, P. Synthesis of 9-amino(9-deoxy)*epi* cinchona alkaloids, general chiral organocatalysts for the stereoselective functionalization of carbonyl compounds. *Nature Protocols,* 8, 325–344 (2013).

[2] For the synthesis of the squaramide catalysts, see: Yang, W. & Du, D. M. Highly Enantioselective Michael Addition of Nitroalkanes to Chalcones Using Chiral Squaramides as Hydrogen Bonding Organocatalysts. *Org. Lett.,* 12, 5450-5453 (2010).

[3] For the synthesis of the thiourea catalysts, see: Zhang, G., Zhu, C., Liu, D., Pan, J., Zhang, J., Hu, D. & Song, B. Solvent-free enantioselective conjugate addition and bioactivities of nitromethane to Chalcone containing pyridine. *Tetrahedron,* 73, 129-136 (2017).

[4] Mielby, J. & Kegnæs, S. Epoxidation of Alkenes with Aqueous Hydrogen Peroxide and Quaternary Ammonium Bicarbonate Catalysts. *Catal. Lett.,* 143, 1162–1165 (2013).

[5] Wang, Y. & Gu, M. The Concept of Spectral Accuracy for MS. *Anal. Chem.,* 82, 7055-7062 (2010).

[6] Wang, Y. Methods for Operating MS Instrument Systems, United States Patent No. 6, 983, 213 (2006).

[7] Ochiaia, N., Sasamoto, K. & MacNamara, K. Characterization of sulfur compounds in whisky by full evaporation dynamic headspace and selectable one-dimensional/two-dimensional retention time locked gas chromatography–mass spectrometry with simultaneous element-specific detection. *Journal of Chromatography A*, 1270, 296-304 (2012).

[8] Ho, H.-P., Lee, R.-Y., Chen, C.-Y., Wang, S.-R., Li, Z.-G. & Lee, M.-R. Identification of new minor metabolites of penicillin G inhuman serum by multiple-stage tandem mass spectrometry. *Rapid Commun. Mass Spectrom.,* 25, 25-32 (2011).

[9] Vander, J. et al. (2006). *Cancer treatment using curcumin derivatives* (United States of America Patent No. US 2006/0276536 A1).

[10] Sun, H. & Yu, P. et al. Synthesis of tetracyclic oxindoles and evaluation of their α-glucosidase inhibitory and glucose consumption-promoting activity. *Bioorganic & Medicinal Chemistry Letters,* 30, 127264-127268 (2020).

[11] After completion of the reaction, the crude was extracted with ethyl acetate (3x10 mL). The combination of all organic phases was washed with brine, dried over $MgSO_4$, filtered, and concentrated under reduced pressure.

[12] Gendron, T., Kessedjian, H., Davioud-Charvet, E. & Lanfranchi, D. A. Diastereoselective Synthesis of 2,6-Diaryltetrahydrothiopyran-4-ones by Phase-Transfer Catalysis. *Eur. J. Org. Chem.,* 1790–1796 (2015).

[13] Parr, A. J., Walton, N. J., Bensalem, S., Mccabe, P. H. & Routledge, W. 8-Thiabicyclo[3.2.1]octan-3-one as a biochemical tool in the study of tropane alkaloid biosynthesis. *Phytochemistry,* 30, 2607-2609 (1991).

[14] This step is used for the precipitation of the remaining sulfonium salt and sodium bicarbonate that might be in the reaction crude. In this manner, the ring opening of the sulfonium salt is avoided in the purification step (it was tested that the sulfonium salt transformed slightly to the final product **3** in the presence of silica) and therefore, the enantiomeric excess is not affected.

[15] A mixture 1:1 of chloroform (0.25 mL) and water (0.25 mL) at 0 ºC was used. After one night, the reaction was extracted with diethyl ether, dried over $MgSO_4$, filtered, and concentrated under reduced pressure.

[16] http://supramolecular.org

[17] Brynn Hibbert, D. & Thordarson, P. The death of the Job plot, transparency, open science and online tools, uncertainty estimation methods and other developments in supramolecular chemistry data analysis. *Chem. Commun.*, 52, 12792-12805 (2016).
